# Gold-Catalyzed Regioselective Synthesis of Crowded Cyclopentadienes by Migratory Cycloisomerization of Vinylallenes

**DOI:** 10.1021/acs.orglett.2c02035

**Published:** 2022-07-12

**Authors:** Olaya Bernardo, Javier González, Javier Borge, Luis A. López

**Affiliations:** † Departamento de Química Orgánica e Inorgánica, Instituto Universitario de Química Organometálica “Enrique Moles” and Centro de Innovación en Química Avanzada (ORFEO−CINQA), Universidad de Oviedo, Julián Clavería 8, 33006 Oviedo, Spain; ‡ Departamento de Química Orgánica e Inorgánica, Universidad de Oviedo, Julián Clavería 8, 33006 Oviedo, Spain; § Departamento de Química Física y Analítica, Universidad de Oviedo, Julián Clavería 8, 33006 Oviedo, Spain

## Abstract

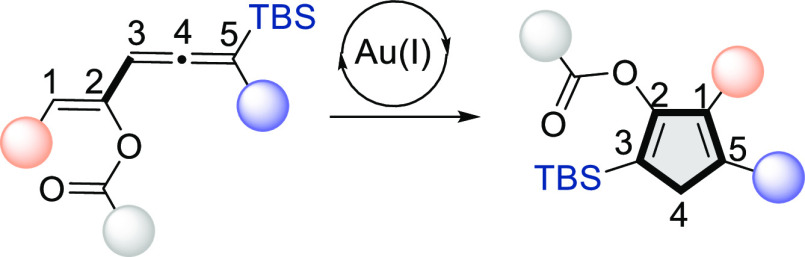

We report the regioselective synthesis of silyl-substituted cyclopentadienyl esters through gold-catalyzed migratory cycloisomerization of silyl-substituted vinylallenes. This transformation is proposed to proceed through a perfectly orchestrated sequence of events including Nazarov-like cyclization and several silyl and hydrogen rearrangements. Furthermore, exploiting the multifaceted nature of the gold catalyst, we have also identified suitable conditions for the synthesis of these cyclopentadienes in a more straightforward manner through gold-catalyzed reaction of propargyl esters and alkynylsilanes.

Cyclopentadiene derivatives are important substrates as reactive diene components in Diels–Alder reactions and as precursors of cyclopentadienyl anions, indispensable ligands in organometallic chemistry.^1^ Remarkably, the outstanding synthetic potential of the cyclopentadiene framework is not in accordance with its availability. In fact, despite significant advances in this field,^2^ straightforward and regioselective access to densely functionalized cyclopentadienes from readily available precursors remains a synthetic challenge, making the development of efficient routes to this valuable scaffold highly desirable. In this realm, gold catalysis has recently emerged as a valuable tool, providing a number of effective solutions to the regioselective synthesis of functionalized cyclopentadiene derivatives. Relevant examples include the gold-catalyzed cyclization of ynamides and gold carbenoid precursors such as propargyl esters^3^ and cyclopropenes.^4^ In this regard, the gold-catalyzed cycloisomerization of vinylallenes depicted in Scheme 1A deserves special mention, as it has evolved as one of the most reliable and useful methodologies for accessing highly substituted cyclopentadienes,^5,6^ even in the challenging scenarios of the total synthesis of complex natural products.^7^ Mechanistically, this transformation is proposed to proceed through initial coordination of the vinylallene to the cationic gold(I) catalyst through the central carbon of the allene moiety,^8^ leading to a pentadienyl cation intermediate, which then would undergo a Nazarov-like cyclization to give a cationic gold(I)-carbenoid intermediate. A final 1,2-hydrogen (or alkyl, in the case of 1,1-dialkyl-substituted substrates) shift would render the final cyclopentadiene derivative with regeneration of the cationic gold catalyst.

**Scheme 1:**
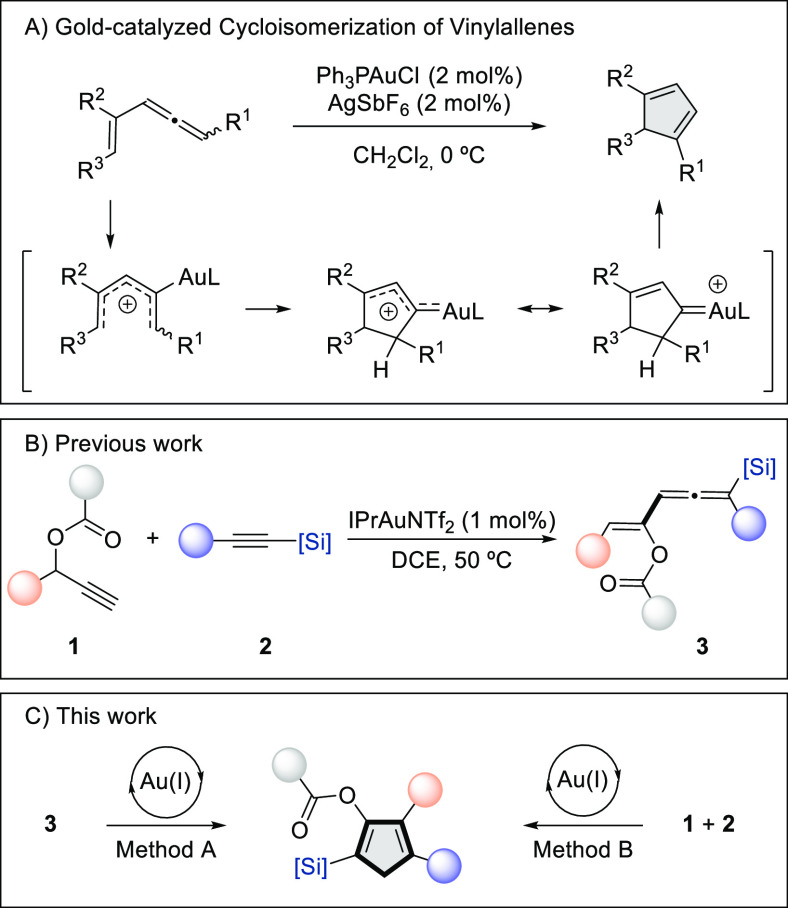
Background and Synopsis of the Present Study

We have recently reported the gold-catalyzed reaction of propargylic esters **1** with alkynylsilanes **2** to provide silyl-substituted vinylallene derivatives **3** resulting from consecutive [1,2]-acyloxy/[1,2]-silyl rearrangements (Scheme 1B).^9^ Notably, under the developed conditions, the formed vinylallenes did not evolve to the corresponding functionalized cyclopentadienes. We posited that under the appropriate reaction conditions these vinylallene derivatives could be converted into densely functionalized cyclopentadiene derivatives. Given the presence of both silyl and aryl (or alkyl) groups bonded to the C1 carbon atom of the allene moiety, the planed study could also add insight into the understudied topic of the relative migratory aptitudes in the proposed gold carbene intermediate.^10^ Herein, we report the gold-catalyzed migratory cycloisomerization of silyl-substituted vinylallene derivatives **3** as a straightforward and regioselective access to crowded silyl-substituted cyclopentadienyl esters (Scheme 1C, Method A). A detailed mechanism for this multistep transformation, substantiated by DFT calculations, is also proposed. These cyclopentadiene derivatives are also accessible directly from gold-catalyzed reaction of propargyl esters **1** and alkynylsilanes **2** (Scheme 1C, Method B).

Proof-of-concept for the formation of silicon-decorated cyclopentadiene derivatives by gold-catalyzed cycloisomerization was attained with vinylallene **3a** (eq 1). After some experimentation,^11^ this substrate was transformed into cyclopentadiene **4a** in excellent yield (91%) when heated in 1,2-dichloroethane (DCE) at 90 °C in the presence of 1.0 mol % of [(IPr)Au(CH_3_CN)]SbF_6_. Under these reaction conditions, no other isomers of **4a** were detected in the crude reaction mixture. The structure of compound **4a** was unambiguously established via single-crystal X-ray analysis of the related cyclopentadiene derivative **4l** (see Table 1).
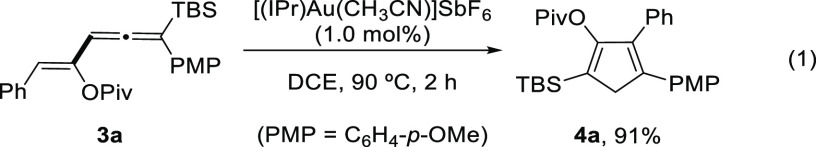
1

**Table 1:**
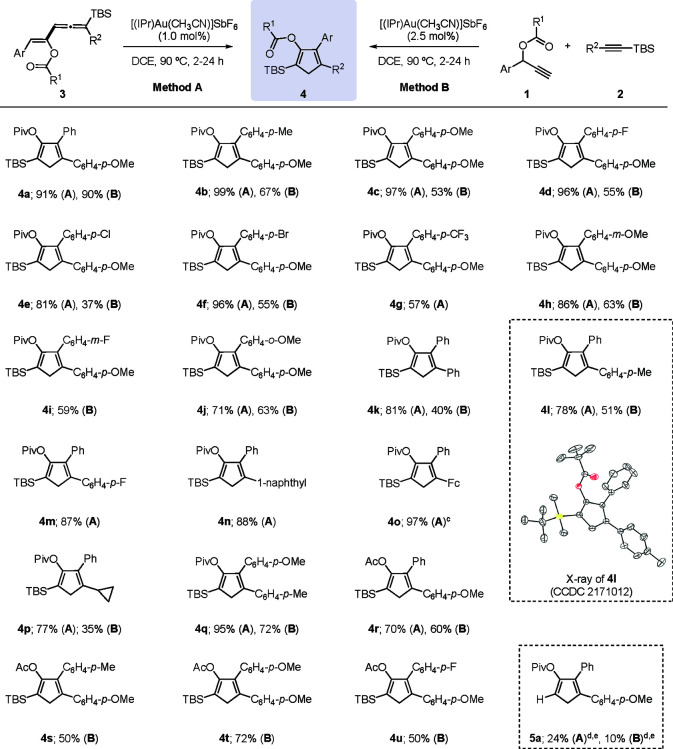
Cyclopentadiene Synthesis from Vinylallenes **3** (Method A) and from Propargyl Esters **1** and Alkynylsilanes **2** (Method B): Scope^a^^,^^b^

a Reaction conditions: Method A: **3** (0.2 mmol), [(IPr)Au(CH_3_CN)]SbF_6_ (1 mol %), DCE (1 mL), 90 °C. Method B: **1** (0.2 mmol), **2** (0.4 mmol, 2 equiv), [(IPr)Au(CH_3_CN)]SbF_6_ (2.5 mol %), DCE (1 mL), 90 °C.

b Yield of isolated products.

c Fc = ferrocenyl.

d The TBS group in **2a** and **3a** was replaced with a TMS group.

e NMR yield using CH_2_Br_2_ as the internal standard.

Having established suitable conditions for the synthesis of cyclopentadiene **4a**, we explored then the scope of this gold-catalyzed migratory cycloisomerization (Table 1, Method A). The reaction proved to be applicable to a wide range of 5-aryl-substituted vinylallenes, and the desired cyclopentadienes were obtained in moderate to good yields. Substrates **3b** and **3c** with electron-donating methyl and methoxy groups at the *para* position of the aromatic ring performed particularly well in this cycloisomerization, providing the corresponding cyclopentadienes **4b** and **4c** in nearly quantitative yields (99 and 97%, respectively). *p*-Halophenyl-substituted vinylallenes **3d**–**f** could also be successfully transformed into the desired cyclopentadienes **4d**–**f** in good to excellent yields (81–96%). A *para*-CF_3_-phenyl group was also compatible with the present conditions, although the corresponding cyclopentadiene derivative **4g** was isolated in moderate yield (57%). A methoxy group at the *meta* position was also well-tolerated as demonstrated by the formation of cyclopentadiene **4h** in good yield (86%). Even an *ortho*-substituted substrate could be engaged in this gold-catalyzed transformation as illustrated by the formation of cyclopentadiene **4j** in 71% yield. Interestingly, restricted rotation of the *ortho*-substituted aryl group was evidenced by the diastereotopicity of methylene protons and TBS methyl groups in the ^1^H NMR spectrum of compound **4j**.

Thereupon, variation of group R^2^ attached to the C1 carbon atom of the vinylallene was addressed. First, we found that an electron-rich aromatic group at this position is not essential. In fact, a phenyl-substituted substrate also performed well, furnishing the desired cyclopentadiene derivative **4k** in 81% isolated yield. Other *para*-substituted substrates bearing methyl and fluoro groups also undergo this transformation, providing the corresponding cyclopentadienes **4l** (78%) and **4m** (87%). A 1-(1-naphthyl)-substituted vinylallene was also an amenable substrate, delivering the corresponding product **4n** in 88% yield. Notably, a vinylallene derivative featuring a ferrocenyl group at the C1 position also performed well, affording the desired cyclopentadiene **4o** in almost quantitative yield. The present cycloisomerization is not restricted to the use of aryl-substituted substrates at the C1 position. In fact, a vinylallene featuring a cyclopropyl group could also be engaged as illustrated by the formation of cyclopentadiene **4p** in 77% yield. Attempted reactions with other alkyl groups such as hexyl and pentyl, however, basically met with failure.

Regarding the ester substituent R^1^, we found that acetate derivative **3q** (R^1^ = Me, Ar = Ph, R^2^ = C_6_H_4_-*p*-OMe) is also a suitable substrate, providing the expected cyclopentadiene **4r** in 70% yield. In contrast, when the TBS group was replaced with a trimethylsilyl (TMS) group, the reaction failed to provide the expected silyl-substituted cyclopentadiene. Instead, the desilylated cyclopentadiene **5a** was formed in low NMR yield (24%) along with other unidentified products.

After demonstrating the viability of the cycloisomerization of vinylallenes **3** into functionalized cyclopentadienes **4**, we decided to investigate the feasibility of preparing compounds **4** in a more straightforward manner starting from the corresponding propargyl esters **1** and alkynylsilanes **2**. To this end, we first studied the model reaction of 1-phenyl-prop-2-yn-1-yl pivalate (**1a**) with *tert*-butyl((4-methoxyphenyl)ethynyl)dimethylsilane (**2a**). To our delight, after a slight reoptimization of the reaction conditions,^11^ we found that stirring a mixture of propargyl ester **1a** and alkynylsilane **2a** (2 equiv) in the presence of 2.5 mol % of [(IPr)Au(MeCN)]SbF_6_ in DCE at 90 °C produced the desired cyclopentadiene **4a** in 90% yield.

Concerning the reaction scope of this cascade reaction (Table 1, Method B), we initially selected alkynylsilane **2a** as the reaction partner to evaluate the performance of a range of aryl-substituted propargyl pivalates (R^1^ = ^*t*^Bu) under the developed reaction conditions. Pleasingly, an array of propargyl esters performed well in this transformation, affording the desired cyclopentadienes **4** in moderate to good yields. These included electron-rich as well as electron-poor groups at the *para*, *meta*, and *ortho* positions of the aromatic ring. Representative propargyl acetates (R^1^ = Me) were screened next, and they provide the expected cyclopentadienes **4r**–**4u** in moderate to good yields (50–72%).

Compared to propargyl ester variations, this gold-catalyzed cascade showed higher sensitivity to changes to the structure of the alkynylsilane component. Thus, while aryl-substituted alkynylsilanes bearing electron-donating groups provided the expected cyclopentadienes in moderate to good yields, alkynylsilanes featuring aryl groups containing electron-withdrawing groups were found to be problematic, delivering the corresponding products in modest yields, even though these substituents were well-tolerated in Method A. The low performance of these alkynylsilanes in the cascade process is consistent with our previous study,^9^ which showed that these alkynylsilanes exhibit much reduced reactivity in the formation of the required vinylallene intermediate.

In agreement with the behavior observed in the gold-catalyzed transformation of TMS-substituted vinylallenes **3** (Method A), the reaction of 1-phenyl-prop-2-yn-1-yl pivalate (**1a**) with 1-(4-methoxyphenyl)-2-trimethylsilylacetylene (**2h**) provided the desilylated cyclopentadiene **5a** in low yield.

To gain further insight into the formation of cyclopentadiene derivatives **4**, we performed the labeling experiment depicted in eq 2.^12^ Subjecting labeled vinylallene [^13^C]**3a** to the previously developed conditions (Method A) delivered the corresponding cyclopentadiene derivative [^13^C]**4a** (87% isolated yield), in which the labeled carbon atom is found in the methylene group of the cyclopentadiene.^13^
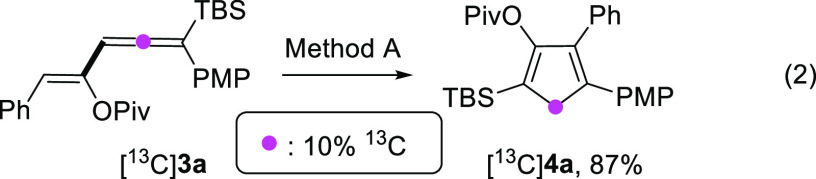
2

Building on this ^13^C labeling experiment and related literature precedents, a mechanism for the formation of cyclopentadiene derivatives **4** from vinylallenes **3** is depicted in Scheme 2. Initial coordination of the cationic gold complex to the vinylallene **3** would generate a pentadienyl cation intermediate **I** that would undergo an electrocyclization reaction, leading to carbene species **II**. Selective 1,2-migration of the silyl group would give rise to the cationic intermediate **III**,^14,15^ which upon demetalation would deliver cyclopentadiene intermediate **IV**. The formation of the final cyclopentadienes **4** could be explained through a series of thermally allowed suprafacial [1,5]-sigmatropic shifts of hydrogen and the ^*t*^BuMe_2_Si group.^16^ Thus, under the thermal conditions, cyclopentadiene **IV** could isomerize to cyclopentadiene **V**, which further isomerizes to intermediate **VI**.^17^ 1,5-Silyl migration in cyclopentadiene **VI** would generate cyclopentadiene **VII**,^18^ which by a subsequent [1,5]-sigmatropic hydrogen shift would render the final cyclopentadiene **4**.^19^

**Scheme 2:**
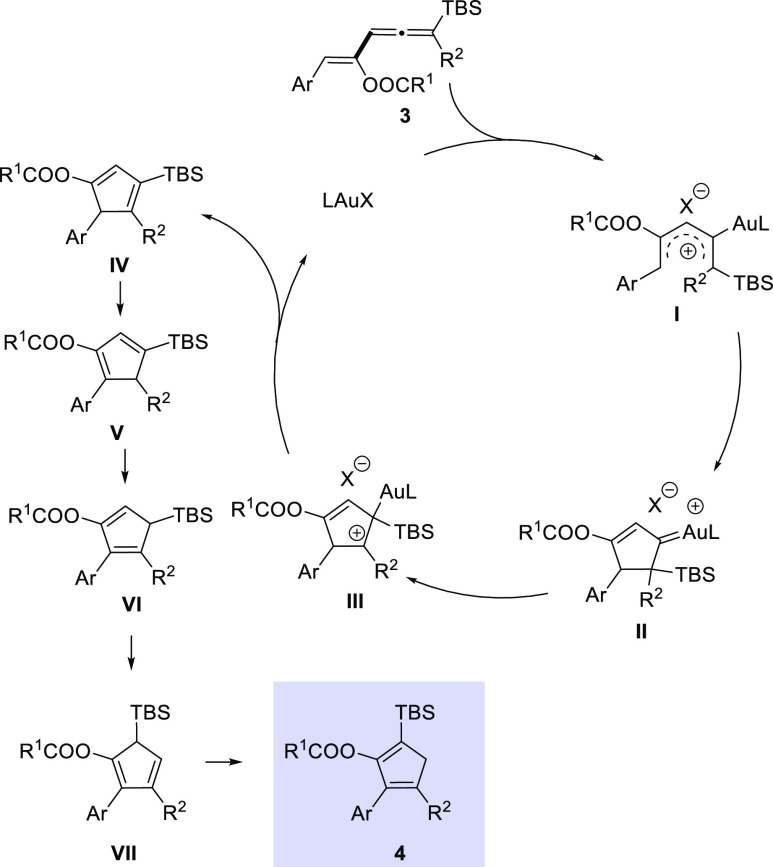
Proposed Reaction Mechanism for the Gold-Catalyzed Cycloisomerization of Vinylallenes **3**

To gain more insight into the mechanism of this multistep reaction, DFT calculations were carried out.^20^ To this end, the transformation of intermediate cyclopentadiene **IVk** (R^1^ = ^*t*^Bu, Ar = R^2^ = Ph) leading to the final product **4k** was computationally explored. According to our calculations, the overall outcome would reflect the thermodynamic preference for the formation of compound **4k**, since it was predicted to be more stable than the rest of the isomeric cyclopentadienes involved in the transformation. Our calculations also predict that the 1,5-silyl migration in intermediate **VIk** to generate cyclopentadiene **VIIk** occurs with a rather low barrier of 14.6 kcal/mol.

As shown in Scheme 3A, the cycloisomerization of vinylallene **3a** preserved its efficiency on a 2.50 mmol scale as illustrated by the formation of cyclopentadiene **4a** without any erosion of the yield. This easy scale-up allowed for follow-up transformations of compound **4a** to be explored (Scheme 3B). In particular, we were highly interested in transforming compound **4a** into 2,3-diaryl-substituted cyclopentenones since these compounds are valuable targets in medicinal chemistry.^21^ Compound **4a** underwent easy protodesilylation to the cyclopentadiene **5a** (86%) when subjected to tetrabutylammonium fluoride (TBAF, 1 equiv) in chloroform at room temperature. Unexpectedly, mere exposure of a solution of compound **5a** in chloroform to air for 3 days led to cyclopentenone **6a** in 61% yield. Next, we evaluated the stability of cyclopentadiene **4a** under acidic conditions. Pleasingly, we found that heating a solution of **4a** in toluene in the presence of 1 M HCl delivered the silyl-substituted cyclopentenone **7a** in excellent yield (97%). Besides, a one-pot protodesilylation/hydrolysis sequence provided cyclopentenone **8a** in good yield (72%).

**Scheme 3:**
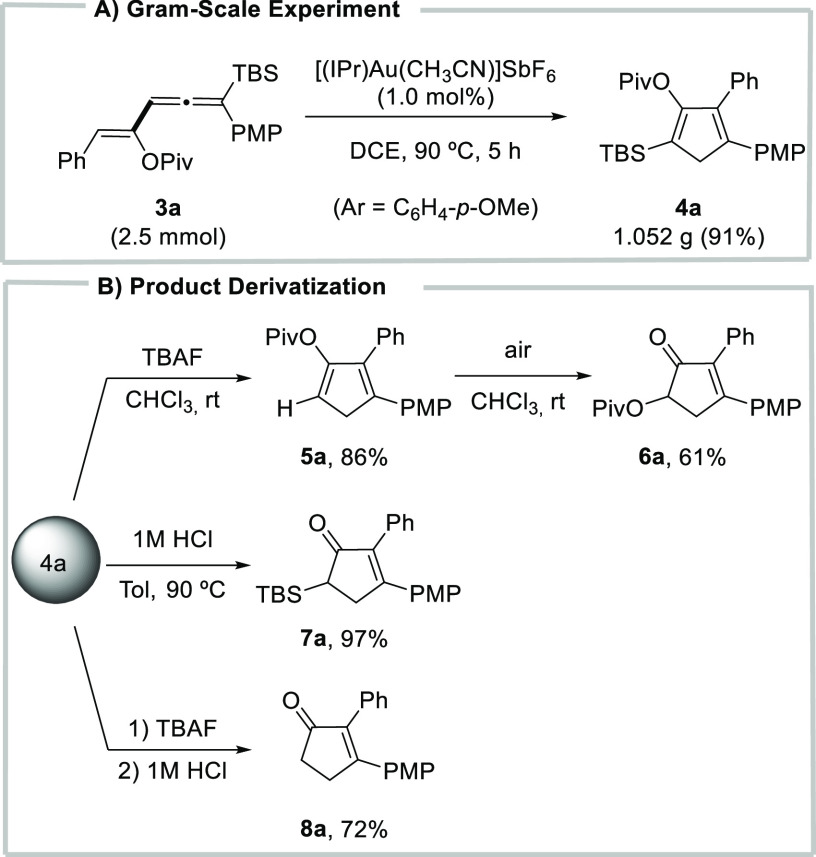
(A) Scale Up of Cyclopentadiene **4a** and (B) Product Derivatization Using **4a**

In conclusion, we have developed efficient syntheses of crowded cyclopentadienes from readily available substrates. It should be noted that the formation of highly substituted cyclopentadienes represents a notable synthetic challenge, and hence the procedures reported constitute an advance in this area. Mechanistically, this cyclization reaction takes place through an intricate, yet perfectly orchestrated, sequence of rearrangements. Very likely, the observed reactivity benefits from the extreme ability of silyl groups to engage in different types of rearrangements. A preliminary study on the reactivity of the reported cyclopentadienes seems to anticipate that they possess great promise as precursors for the synthesis of valuable cyclopentenone derivatives. Further efforts aimed at expanding the synthetic potential of this family of compounds are underway in our group.

## Supporting Information Available

Experimental procedures, computational data, and characterization data for all new compounds (PDF)

## Accession Codes

CCDC 2171012 contains the supplementary crystallographic data for this paper. These data can be obtained free of charge via www.ccdc.cam.ac.uk/data_request/cif, or by emailing data_request@ccdc.cam.ac.uk, or by contacting The Cambridge Crystallographic Data Centre, 12 Union Road, Cambridge CB2 1EZ, UK; fax: +44 1223 336033.
