# Supplementary material for: Gold-Catalyzed Regioselective Synthesis of Crowded Cyclopentadienes by Migratory Cycloisomerization of Vinylallenes
Source: Org Lett. 2022 Jul 12;24(28):5203–8. doi: 10.1021/acs.orglett.2c02035 (PMC9490818; doi:10.1021/acs.orglett.2c02035)

Supporting Information

Gold-Catalyzed Regioselective Synthesis of Crowded Cyclopentadienes by Migratory Cycloisomerization of Vinylallenes

Olaya Bernardo,[a] Javier González,[b] Javier Borge,[c] Luis A. López[a]*

[a] Departamento de Química Orgánica e Inorgánica, Instituto Universitario de Química Organometálica "Enrique Moles" and Centro de Innovación en Química Avanzada (ORFEO-CINQA), Universidad de Oviedo, Julián Clavería 8, 33006-Oviedo (Spain)

[b] Departamento de Química Orgánica e Inorgánica, Universidad de Oviedo, Julián Clavería 8, 33006-Oviedo (Spain)

[c] Departamento de Química Física y Analítica, Universidad de Oviedo, Julián Clavería 8, 33006-Oviedo (Spain)

lalg@uniovi.es

Table of Contents

**1.**	General Considerations	S-3
**2.**	Preparation of Vinyl Allenes **3**	S-5
**3.**	Synthesis of Cyclopentadienes **4** (Method A): Optimization	S-12
**4.**	General Procedure for the Synthesis of Cyclopentadienes **4** (Method A)	S-13
**5.**	Synthesis of Cyclopentadienes **4** (Method B): Optimization	S-14
**6.**	General Procedure for the Synthesis of Cyclopentadienes **4** (Method B)	S-15
**7.**	Characterization Data of Cyclopentadienes **4**	S-16
**8.**	^{13}C-Labelling Experiments	S-28
**9.**	Computational Details	S-35
**10.**	Experimental Procedure for the Synthesis of **4a** (2 mmol Scale)	S-54
**11.**	Product Derivatization	S-55
**12.**	References	S-58
**13.**	NMR spectra for new compounds	S-59

1. General Considerations

Reactions were performed in a RR9803012 place Carousel Reaction Station^{TM} from Radleys Discovery Technologies, equipped with gastight threaded caps with a valve, cooling reflux head system, and digital temperature controller. All reactions were carried under an atmosphere of nitrogen (99.99%). 1,2-Dichloroethane (DCE) was distilled from CaH_2 before use. The solvents used in column chromatography were obtained from commercial suppliers and used without further distillation. TLC was performed on aluminum-backed plates coated with silica gel 60 with F254 indicator (Merck), using UV light as a visualizing agent and phosphomolybdic acid in ethanol, potassium permanganate solution or *p*-anisaldehyde in ethanol, and heat as developing agent. Flash chromatography was performed on silica gel (40-60 um). ^{1}H NMR (300, 400 MHz) and ^{13}C NMR (75.5, 100 MHz) spectra were measured in $CDCl_3$ at room temperature on a Bruker DPX-300, Bruker AV-300 MHz and Bruker AV-400 instruments, with $CDCl_3$ (δ = 7.26, ^{1}H NMR; δ = 77.16, ^{13}C NMR) as internal standard. Carbon multiplicities were assigned by DEPT techniques.

High-resolution mass spectra (HRMS) were determined by Universidad de Oviedo with a Bruker Impact II, Q – TOF mass Spectrometer.

Melting points (m. p.) of solid samples were measured on a Buchi-Tottoli apparatus and are uncorrected.

This study was carried out using propargyl esters **1a**-**n**,[1] alkynylsilanes **2a**-**j**,[2] and vinyl allenes **3a-r**[3] (Figure S1), which were prepared according to procedures previously described in the literature. ^{13}C-labelled alkynylsilane [^{13}C]**2a** was prepared from labelled CBr_4 through a Corey-Fuchs protocol according to a procedure reported by Zhang and co-workers.[4] All other reagents used in this work were of the best commercial grade available and used without further purification.

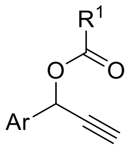

1a (Ar = Ph; R^1 = ^{t}Bu)
1b (Ar = C_6H_4-*p*-Me; R^1 = ^{t}Bu)
1c (Ar = C_6H_4-*p*-OMe; R^1 = ^{t}Bu)
1d (Ar = C_6H_4-*p*-F; R^1 = ^{t}Bu)
1e (Ar = C_6H_4-*p*-Cl; R^1 = ^{t}Bu)
1f (Ar = C_6H_4-*p*-Br; R^1 = ^{t}Bu)
1g (Ar = C_6H_4-*p*-CF_3; R^1 = ^{t}Bu)
1h (Ar = C_6H_4-*m*-OMe; R^1 = ^{t}Bu)
1i (Ar = C_6H_4-*m*-F; R^1 = ^{t}Bu)
1j (Ar = C_6H_4-*o*-OMe; R^1 = ^{t}Bu)
1k (Ar = Ph; R^1 = Me)
1l (Ar = C_6H_4-*p*-Me; R^1 = Me)
1m (Ar = C_6H_4-*p*-OMe; R^1 = Me)
1n (Ar = C_6H_4-*p*-F; R^1 = Me)

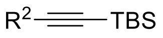

2a (R = C_6H_4-*p*-OMe)
2b (R = C_6H_4)
2c (R = C_6H_4-*p*-Me)
2d (R = C_6H_4-*p*-F)
2e (R = 1-naphthyl)
2f (R = ferrocenyl)
2g (R = cyclopropyl)

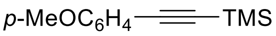

2h

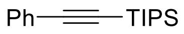

2i

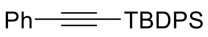

2j

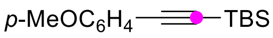

[^{13}C]**2a**

● : 10 % ^{13}C

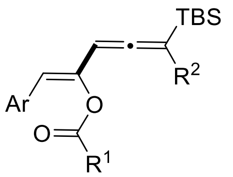

3a (Ar = Ph; R^1 = ^{t}Bu; R^2 = C_6H_4-*p*-OMe)
3b (Ar = C_6H_4-*p*-Me; R^1 = ^{t}Bu; R^2 = C_6H_4-*p*-OMe)
3c (Ar = C_6H_4-*p*-OMe; R^1 = ^{t}Bu; R^2 = C_6H_4-*p*-OMe)
3d (Ar = C_6H_4-*p*-F; R^1 = ^{t}Bu; R^2 = C_6H_4-*p*-OMe)
3e (Ar = C_6H_4-*p*-Cl; R^1 = ^{t}Bu; R^2 = C_6H_4-*p*-OMe)
3f (Ar = C_6H_4-*p*-Br; R^1 = ^{t}Bu; R^2 = C_6H_4-*p*-OMe)
3g (Ar = C_6H_4-*p*-CF_3; R^1 = ^{t}Bu; R^2 = C_6H_4-*p*-OMe)
3h (Ar = C_6H_4-*m*-OMe; R^1 = ^{t}Bu; R^2 = C_6H_4-*p*-OMe)
3i (Ar = C_6H_4-*o*-OMe; R^1 = ^{t}Bu; R^2 = C_6H_4-*p*-OMe)
3j (Ar = Ph; R^1 = ^{t}Bu; R^2 = Ph)
3k (Ar = Ph; R^1 = ^{t}Bu; R^2 = C_6H_4-*p*-Me)
3l (Ar = Ph; R^1 = ^{t}Bu; R^2 = C_6H_4-*p*-F)
3m (Ar = Ph; R^1 = ^{t}Bu; R^2 = 1-naphthyl)
3n (Ar = Ph; R^1 = ^{t}Bu; R^2 = ferrocenyl)
3o (Ar = Ph; R^1 = ^{t}Bu; R^2 = cyclopropyl)
3p (Ar = C_6H_4-*p*-OMe; R^1 = Me; R^2 = C_6H_4-*p*-Me)
3q (Ar = Ph; R^1 = Me; R^2 = C_6H_4-*p*-OMe)

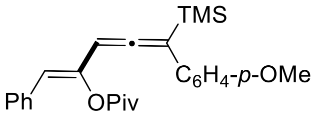

3r

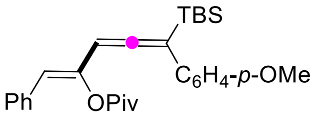

[^{13}C]**3a**

● : 10 % ^{13}C

Figure S1. Starting materials used in this work

2. Preparation of Vinyl Allenes 3

Vinyl allenes **3b**-**i** and **3l**-**n** were not previously reported and were prepared by gold-catalyzed reaction of propargyl esters **1** and alkynylsilanes **2** according to our previous report.[3]

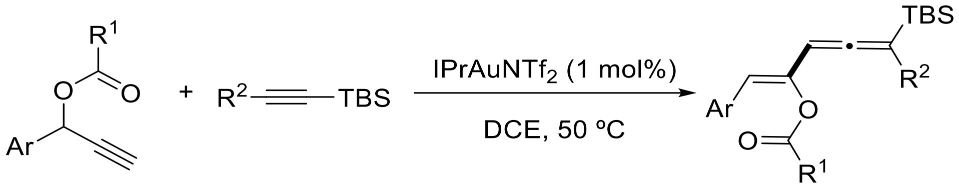

1 **2** **3**

$IPrAuNTf_2$ (3.4 mg, 0.004 mmol, 1.0 mol%) was added to a solution of the corresponding propargyl ester **1** (0.40 mmol) and alkynylsilane **2** (0.80 mmol, 2.0 equiv.) in 1,2-dichloroethane (DCE, 2 mL). The resulting mixture was stirred at 50 ºC until disappearance of **1** (checked by TLC). Then, the solvent was removed under reduced pressure and the resulting mixture was purified by flash chromatography (silica gel; hexanes/ethyl acetate 4:1) to yield allenylsilanes **3**.

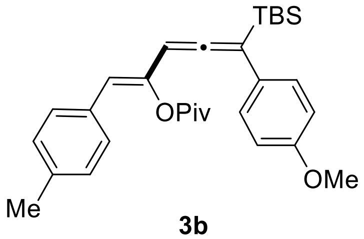

3b

5-(*tert*-butyldimethylsilyl)-5-(4-methoxyphenyl)-1-(*p*-tolyl)penta-1,3,4-trien-2-yl pivalate (3b)

Compound **3b** was obtained from propargyl ester **1b** (85.7 mg, 0.40 mmol) and alkynylsilane **2a** (197.1 mg, 0.80 mmol). Final purification by flash chromatography (silica gel; hexanes/ethyl acetate 4:1) provided compound **3b** (146.8 mg, 77% yield) as a colorless oil.

^{1}H NMR (300 MHz, $CDCl_3$): δ = 7.34 (d, *J* = 8.0 Hz, 2H), 7.30 (d, *J* = 8.8 Hz, 2H), 7.14 (d, *J* = 8.0 Hz, 2H), 6.88 (d, *J* = 8.8 Hz, 2H), 6.18 (s, 1H), 6.02 (s, 1H), 3.82 (s, 3H), 2.36 (s, 3H), 1.22 (s, 9H), 0.98 (s, 9H), 0.36 (s, 3H), 0.28 (s, 3H) ppm.

^{13}C NMR (75 MHz, $CDCl_3$): δ = 210.0 (C), 175.6 (C), 158.8 (C), 142.4 (C), 137.4 (C), 132.1 (C), 130.0 (CH), 129.4 (CH), 128.9 (CH), 128.7 (C), 117.0 (CH), 114.2 (CH), 103.7 (C), 90.7 (CH), 55.6 (CH_3), 39.3 (C), 27.7 (CH_3), 27.5 (CH_3), 21.7 (CH_3), 19.0 (C), -3.6 (CH_3), -3.7 (CH_3) ppm.

HRMS (EI) m/z: $[M + H]^+$ Calcd for $C_{30}H_{41}O_3Si$ 477.2819; Found 477.2821.

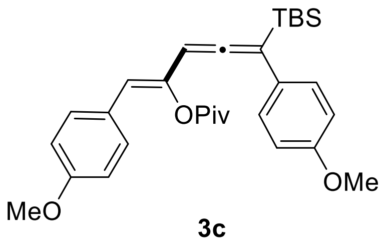

3c

5-(*tert*-butyldimethylsilyl)-1,5-bis(4-methoxyphenyl)penta-1,3,4-trien-2-yl pivalate (3c)

Compound **3c** was obtained from propargyl ester **1c** (92.1 mg, 0.40 mmol) and alkynylsilane **2a** (197.1 mg, 0.80 mmol). Final purification by flash chromatography (silica gel; hexanes/ethyl acetate 4:1) provided compound **3c** (139.9 mg, 71% yield) as a yellow oil.

^{1}H NMR (300 MHz, $CDCl_3$): δ = 7.37 (d, *J* = 8.8 Hz, 2H), 7.28 (d, *J* = 8.8 Hz, 2H), 6.86 (d, *J* = 8.8 Hz, 2H), 6.85 (d, *J* = 8.8 Hz, 2H), 6.14 (s, 1H), 6.00 (s, 1H), 3.85 (s, 6H), 1.21 (s, 9H), 0.96 (s, 9H), 0.34 (s, 3H), 0.26 (s, 3H) ppm.

^{13}C NMR (75 MHz, $CDCl_3$): δ = 209.9 (C), 175.6 (C), 159.0 (C), 158.8 (C), 141.5 (C), 130.2 (CH), 130.0 (CH), 128.7 (C), 127.7 (C), 116.5 (CH), 114.2 (CH), 103.7 (C), 90.7 (CH), 55.6 (CH_3), 39.3 (C), 27.7 (CH_3), 27.5 (CH_3), 19.0 (C), -3.6 (CH_3), -3.7 (CH_3) ppm.

HRMS (EI) m/z: $[M + H]^+$ Calcd for $C_{30}H_{41}O_4Si$ 493.2769; Found 493.2772.

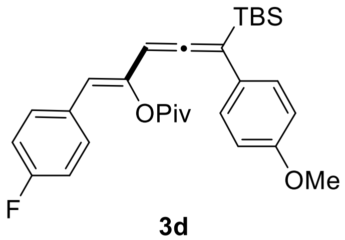

3d

5-(*tert*-butyldimethylsilyl)-1-(4-fluorophenyl)-5-(4-methoxyphenyl)penta-1,3,4-trien-2-yl pivalate (3d)

Compound **3d** was obtained from propargyl ester **1d** (87.3 mg, 0.40 mmol) and alkynylsilane **2a** (197.1 mg, 0.80 mmol). Final purification by flash chromatography (silica gel; hexanes/ethyl acetate 4:1) provided compound **3d** (186.5 mg, 97% yield) as a yellow oil.

^{1}H NMR (300 MHz, $CDCl_3$): δ = 7.42-7.37 (m, 2H), 7.29 (d, *J* = 8.8 Hz, 2H), 7.03-6.98 (m, 2H), 6.88 (d, *J* = 8.8 Hz, 2H), 6.16 (s, 1H), 6.00 (s, 1H), 3.81 (s, 3H), 1.20 (s, 9H), 0.97 (s, 9H), 0.36 (s, 3H), 0.28 (s, 3H) ppm.

^{13}C NMR (75 MHz, $CDCl_3$): δ = 209.9 (C), 175.5 (C), 162.1 (C, J_{C-F} = 245.7 Hz), 158.1 (C), 140.0 (C), 131.1 (C, J_{C-F} = 3.3 Hz), 130.6 (CH, J_{C-F} = 7.7 Hz), 130.0 (CH), 128.5 (C), 115.8 (CH), 115.6 (CH, J_{C-F} = 21.3 Hz), 114.2 (CH), 103.8 (C), 90.4 (CH), 55.6 (CH_3), 39.3 (C), 27.7 (CH_3), 27.4 (CH_3), 19.0 (C), -3.6 (CH_3), -3.7 (CH_3) ppm.

[19]**F NMR** (282 MHz, $CDCl_3$): δ = -114.0 ppm.

HRMS (EI) m/z: $[M + H]^+$ Calcd for $C_{29}H_{38}FO_3Si$ 481.2569; Found 481.2572.

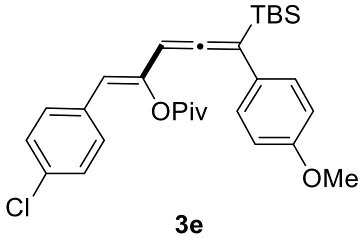

3e

5-(*tert*-butyldimethylsilyl)-1-(4-chlorophenyl)-5-(4-methoxyphenyl)penta-1,3,4-trien-2-yl pivalate (3e)

Compound **3e** was obtained from propargyl ester **1e** (93.9 mg, 0.40 mmol) and alkynylsilane **2a** (197.1 mg, 0.80 mmol). Final purification by flash chromatography (silica gel; hexanes/ethyl acetate 4:1) provided compound **3e** (127.3 mg, 64% yield) as a yellow oil.

[1]**H NMR** (300 MHz, $CDCl_3$): δ = 7.35 (d, *J* = 8.7 Hz, 2H), 7.29-7.27 (m, 4H), 6.87 (d, *J* = 8.8 Hz, 2H), 6.14 (s, 1H), 6.00 (s, 1H), 3.81 (s, 3H), 1.20 (s, 9H), 0.96 (s, 9H), 0.36 (s, 3H), 0.27 (s, 3H) ppm.

[13]**C NMR** (75 MHz, $CDCl_3$): δ = 209.9 (C), 175.5 (C), 158.9 (C), 143.8 (C), 133.5 (C), 133.1 (C), 130.2 (CH), 130.0 (CH), 128.8 (CH), 128.3 (C), 115.8 (CH), 114.3 (CH), 103.9 (C), 90.4 (CH), 55.6 (CH_3), 39.3 (C), 27.8 (CH_3), 27.4 (CH_3), 19.0 (C), -3.6 (CH_3), -3.7 (CH_3) ppm.

HRMS (EI) m/z: $[M + H]^+$ Calcd for $C_{29}H_{38}ClO_3Si$ 497.2273; Found 497.2276.

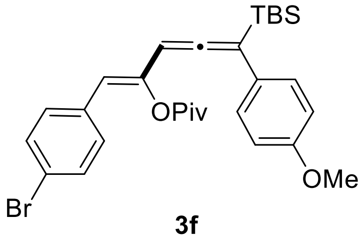

3f

1-(4-bromophenyl)-5-(*tert*-butyldimethylsilyl)-5-(4-methoxyphenyl)penta-1,3,4-trien-2-yl pivalate (3f)

Compound **3f** was obtained from propargyl ester **1f** (111.7 mg, 0.40 mmol) and alkynylsilane **2a** (197.1 mg, 0.80 mmol). Final purification by flash chromatography (silica gel; hexanes/ethyl acetate 4:1) provided compound **3f** (95.5 mg, 45% yield) as a colorless oil.

[1]**H NMR** (300 MHz, $CDCl_3$): δ = 7.42 (d, *J* = 8.3 Hz, 2H), 7.29-7.24 (m, 4H), 6.85 (d, *J* = 8.8 Hz, 2H), 6.11 (s, 1H), 5.98 (s, 1H), 3.81 (s, 3H), 1.18 (s, 9H), 0.95 (s, 9H), 0.34 (s, 3H), 0.25 (s, 3H) ppm.

^{13}**C NMR** (75 MHz, $CDCl_3$): δ = 209.9 (C), 175.4 (C), 158.9 (C), 143.9 (C), 139.9 (C), 131.8 (CH), 130.4 (CH), 130.0 (CH), 128.3 (C), 121.3 (C), 115.8 (CH), 114.2 (CH), 103.9 (C), 90.4 (CH), 55.6 (CH_3), 39.3 (C), 27.7 (CH_3), 27.4 (CH_3), 19.0 (C), -3.6 (CH_3), -3.7 (CH_3) ppm.

HRMS (EI) m/z: $[M + Na]^+$ Calcd for $C_{29}H_{37}BrNaO_3Si$ 563.1588; Found 563.1593.

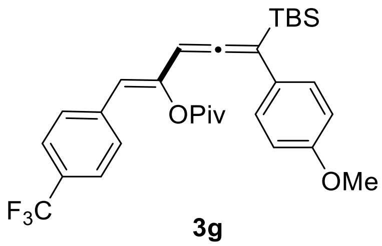

3g

5-(*tert*-butyldimethylsilyl)-5-(4-methoxyphenyl)-1-(4-(trifluoromethyl)phenyl)penta-1,3,4-trien-2-yl pivalate (3g)

Compound **3g** was obtaiened from propargyl ester **1g** (107.3 mg, 0.40 mmol) and alkynylsilane **2a** (197.1 mg, 0.80 mmol). Final purification by flash chromatography (silica gel; hexanes/ethyl acetate 4:1) provided compound **3g** (57.3 mg, 27% yield) as a yellow oil.

^{1}**H NMR** (300 MHz, $CDCl_3$): δ = 7.55 (d, *J* = 8.5 Hz, 2H), 7.50 (d, *J* = 8.5 Hz, 2H), 7.26 (d, *J* = 8.8 Hz, 2H), 6.86 (d, *J* = 8.8 Hz, 2H), 6.21 (s, 1H), 5.99 (s, 1H), 3.82 (s, 3H), 1.16 (s, 9H), 0.95 (s, 9H), 0.35 (s, 3H), 0.26 (s, 3H) ppm.

^{13}**C NMR** (75 MHz, $CDCl_3$): δ = 209.5 (C), 175.0 (C), 158.6 (C), 144.9 (C), 138.2 (C), 129.6 (CH), 128.9 (C), 128.6 (CH), 127.8 (C), 125.1 (CH, q, $J_{C\text{-}F}$ = 4.0 Hz), 124.1 (C, q, $J_{C\text{-}F}$ = 268.6 Hz), 122.4 (C), 115.1 (CH), 113.9 (CH), 113.7 (C), 89.8 (CH), 55.2 (CH_3), 38.9 (C), 27.2 (CH_3), 27.0 (CH_3), 18.6 (C), - 4.1 (CH_3), - 4.2 (CH_3) ppm.

^{19}**F NMR** (282 MHz, $CDCl_3$) = δ = - 62.5 ppm.

HRMS (EI) m/z: $[M + H]^+$ Calcd for $C_{30}H_{38}F_3O_3Si$ 531.2537; Found 531.2541.

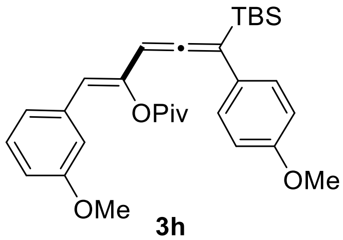

3h

5-(*tert*-butyldimethylsilyl)-1-(3-methoxyphenyl)-5-(4-methoxyphenyl)penta-1,3,4-trien-2-yl pivalate (3h)

Compound **3h** was obtained from propargyl ester **1h** (92.1 mg, 0.40 mmol) and alkynylsilane **2a** (197.1 mg, 0.80 mmol). Final purification by flash chromatography (silica gel; hexanes/ethyl acetate 4:1) provided compound **3h** (149.8 mg, 76% yield) as an orange oil.

^{1}**H NMR** (300 MHz, $CDCl_3$): δ = 7.30 (d, *J* = 8.8 Hz, 2H), 7.24 (t, *J* = 7.9 Hz, 1H), 7.05 (d, *J* = 7.9 Hz, 1H), 6.98 (br s, 1H), 6.88 (d, *J* = 8.8 Hz, 2H), 6.79 (dd, *J* = 7.9 and 2.6 Hz, 1H), 6.19 (s, 1H), 6.02 (s, 1H), 3.81 (s, 3H), 3.80 (s, 3H), 1.22 (s, 9H), 0.98 (s, 9H), 0.37 (s, 3H), 0.29 (s, 3H) ppm.

^{13}**C NMR** (75 MHz, $CDCl_3$): δ = 210.0 (C), 175.6 (C), 159.9 (C), 158.9 (C), 146.4 (C), 136.2 (C), 130.0 (CH), 129.6 (CH), 128.5 (C), 121.6 (CH), 117.0 (CH), 114.8 (CH), 114.2 (CH), 112.8 (CH), 103.8 (C), 90.6 (CH), 55.6 (CH_3), 39.3 (C), 27.7 (CH_3), 27.5 (CH_3), 19.0 (C), -3.6 (CH_3), -3.7 (CH_3) ppm.

HRMS (EI) m/z: $[M + H]^+$ Calcd for $C_{30}H_{41}O_4Si$ 493.2769; Found 493.2775.

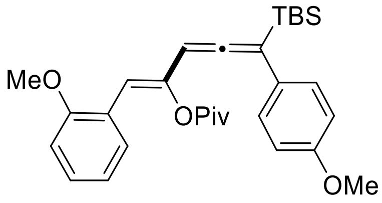

3i

5-(*tert*-butyldimethylsilyl)-1-(2-methoxyphenyl)-5-(4-methoxyphenyl)penta-1,3,4-trien-2-yl pivalate (3i)

Compound **3i** was obtained from propargyl ester **1j** (92.1 mg, 0.40 mmol) and alkynylsilane **2a** (197.1 mg, 0.80 mmol). Final purification by flash chromatography (silica gel; hexanes/ethyl acetate 4:1) provided compound **3i** (130.1 mg, 66% yield) as an orange oil.

^{1}**H NMR** (300 MHz, $CDCl_3$): δ = 7.55 (d, *J* = 7.7 Hz, 1H), 7.29 (d, *J* = 8.3 Hz, 2H), 7.24-7.19 (m, 2H), 6.92-6.86 (m, 3H), 6.53 (s, 1H), 6.07 (s, 1H), 3.85 (s, 3H), 3.82 (s, 3H), 1.18 (s, 9H), 0.98 (s, 9H), 0.33 (s, 3H), 0.28 (s, 3H) ppm.

^{13}**C NMR** (75 MHz, $CDCl_3$): δ = 210.0 (C), 175.6 (C), 158.8 (C), 157.1 (C), 142.9 (C), 130.0 (CH), 129.6 (CH), 128.9 (C), 128.8 (CH), 123.8 (C), 120.4 (CH), 114.2 (CH), 111.6 (CH), 110.8 (CH), 103.6 (C), 90.9 (CH), 55.8 (CH_3), 55.6 (CH_3), 39.3 (C), 27.7 (CH_3), 27.5 (CH_3), 19.0 (C), -3.6 (CH_3), -3.7 (CH_3) ppm.

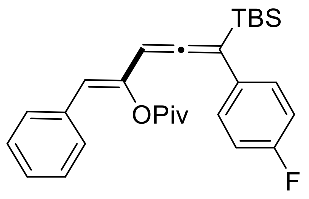

3l

5-(*tert*-butyldimethylsilyl)-5-(4-fluorophenyl)-1-phenylpenta-1,3,4-trien-2-yl pivalate (3l)

Compound **3l** was obtained from propargyl ester **1a** (80.1 mg, 0.40 mmol) and alkynylsilane **2d** (187.5 mg, 0.80 mmol). Final purification by flash chromatography (silica gel; hexanes/ethyl acetate 4:1) provided compound **3l** (111.8 mg, 62% yield) as a yellow oil.

^{1}H NMR (300 MHz, $CDCl_3$): δ = 7.43 (d, *J* = 8.5 Hz, 2H), 7.34-7.23 (m, 5H), 7.02 (t, *J* = 8.7 Hz, 2H), 6.22 (s, 1H), 6.04 (s, 1H), 1.20 (s, 9H), 0.96 (s, 9H), 0.35 (s, 3H), 0.28 (s, 3H) ppm.

^{13}C NMR (75 MHz, $CDCl_3$): δ = 210.3 (C), 175.6 (C), 162.0 (C, $J_{C\text{-}F}$ = 244.7 Hz), 142.7 (C), 134.8 (C), 132.5 (C, $J_{C\text{-}F}$ = 3.4 Hz), 130.3 (CH, $J_{C\text{-}F}$ = 7.9 Hz), 129.0 (CH), 128.7 (CH), 127.7 (CH), 117.5 (CH), 115.7 (CH, $J_{C\text{-}F}$ = 21.1 Hz), 103.6 (C), 90.7 (CH), 39.3 (C), 27.7 (CH_3), 27.4 (CH_3), 19.0 (C), -3.7 (CH_3), -3.8 (CH_3) ppm.

^{19}F NMR (282 MHz, $CDCl_3$) = δ = -115.7 ppm.

HRMS (EI) m/z: $[M + H]^+$ Calcd for $C_{28}H_{36}FO_2Si$ 451.2463; Found 451.2463.

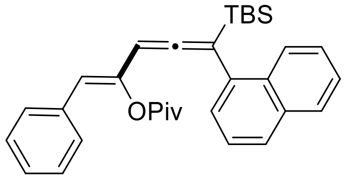

3m

5-(*tert*-butyldimethylsilyl)-5-(naphthalen-1-yl)-1-phenylpenta-1,3,4-trien-2-yl pivalate (3m)

Compound **3m** was obtained from propargyl ester **1a** (80.1 mg, 0.40 mmol) and alkynylsilane **2e** (213.2 mg, 0.80 mmol). Final purification by flash chromatography (silica gel; hexanes/ethyl acetate 4:1) provided compound **3m** (73.4 mg, 38% yield) as a yellow oil.

^{1}H NMR (300 MHz, $CDCl_3$): δ = 8.18-8.15 (m, 1H), 7.89-7.86 (m, 1H), 7.76 (d, *J* = 8.1 Hz, 2H), 7.54-7.31 (m, 8H), 6.21 (s, 1H), 5.89 (s, 1H), 1.30 (s, 9H), 0.89 (s, 9H), 0.27 (s, 3H), 0.22 (s, 3H) ppm.

^{13}C NMR (75 MHz, $CDCl_3$): δ = 208.4 (C), 175.4 (C), 142.9 (C), 134.9 (C), 134.6 (C), 131.6 (C), 128.9 (CH), 128.6 (CH), 127.5 (CH), 127.4 (CH), 126.3 (CH), 126.2 (CH), 125.8 (CH), 125.5 (CH), 117.0 (CH), 101.9 (C), 88.4 (CH), 39.4 (C), 27.9 (CH_3), 27.2 (CH_3), 19.2 (C), -4.2 (CH_3), -4.5 (CH_3) ppm.

HRMS (EI) m/z: $[M + H]^+$ Calcd for $C_{32}H_{39}O_2Si$ 483.2714; Found 483.2721.

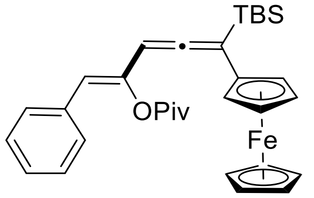

3n

5-(*tert*-butyldimethylsilyl)-5-(ferrocenyl)-1-phenylpenta-1,3,4-trien-2-yl pivalate (3n)

Compound **3n** was obtained from propargyl ester **1a** (80.1 mg, 0.40 mmol) and alkynylsilane **2f** (259.5 mg, 0.80 mmol). Final purification by flash chromatography (silica gel; hexanes/ethyl acetate 4:1) provided compound **3n** (108.1 mg, 50% yield) as orange solid (m.p.: 125.7-127.8 ºC).

^{1}H NMR (300 MHz, $CDCl_3$): δ = 7.46-7.44 (m, 2H), 7.36-7.31 (m, 2H), 7.26-7.23 (m, 1H), 6.24 (s, 1H), 5.86 (s, 1H), 4.47-4.46 (m, 1H), 4.22-4.17 (s + m, 8H), 1.24 (s, 9H), 1.06 (s, 9H), 0.26 (s, 6H) ppm.

^{13}C NMR (75 MHz, $CDCl_3$): δ = 208.9 (C), 175.8 (C), 143.2 (C), 135.0 (C), 129.0 (CH), 128.7 (CH), 127.5 (CH), 116.8 (CH), 101.0 (C), 90.3 (CH), 81.8 (C), 70.1 (CH), 69.8 (CH), 69.4 (CH), 68.3 (CH), 39.4 (C), 27.7 (CH_3), 27.3 (CH_3), 18.7 (C), -3.8 (CH_3), -4.2 (CH_3) ppm.

HRMS (EI) m/z: $[M + Na]^+$ Calcd for $C_{32}H_{40}FeNaO_2Si$ 563.2039; Found 563.2039.

3. Synthesis of Cyclopentadienes 4 (Method A): Optimization

Table S1. Optimization of reaction conditions[a]

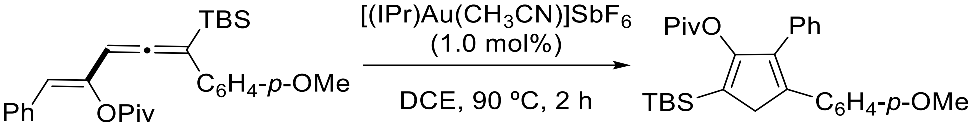

3a **4a**

Entry	Variation from optimal conditions	Yield (%)[b]
1	No changes	91
2	$[IPrAu][NTf_2]$ instead of $[(IPr)Au(CH_3CN)]SbF_6$	80
3	$PtCl_2$ instead of $[(IPr)Au(CH_3CN)]SbF_6$	18
4	DCM instead of DCE	50
5	Toluene instead of DCE	90
6	70 °C instead of 90 °C	n.d.
7	2 mol% instead of 1 mol%	90

[a] These experiments were performed on a 0.20 mmol scale. [b] Yield of the isolated product after chromatographic purification (silica gel; hexanes/ethyl acetate 40:1).

4. General Procedure for the Synthesis of Cyclopentadienes 4 (Method A)

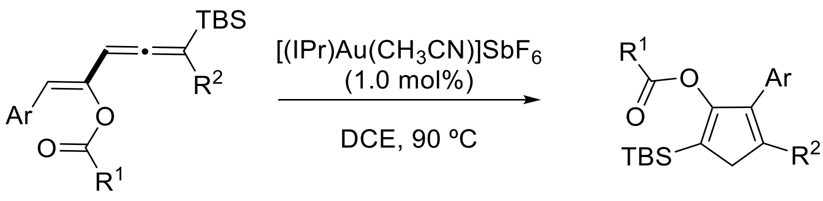

3 **4**

[(IPr)Au(CH_3CN)]SbF_6 (1.7 mg, 1.0 mol%) was added to a solution of the corresponding vinylallene **3** (0.20 mmol) in DCE (1 mL). The resulting mixture was stirred at 90 ºC until disappearance of the starting vinyl allene (checked by TLC, 2-24 h). Then, the solvent was removed under reduced pressure and the resulting mixture was purified by flash chromatography (silica gel, hexanes/ethyl acetate 40:1) to yield cyclopentadienes **4**.

5. Synthesis of Cyclopentadienes 4 (Method B): Optimization

Table S2. Optimization of reaction conditions[a]

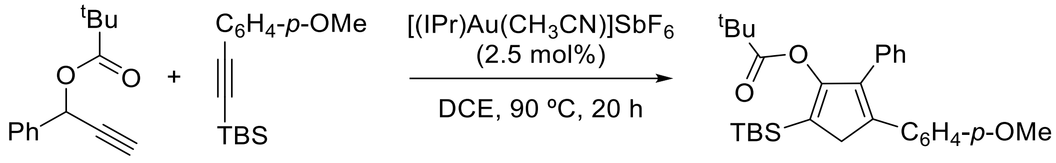

1a **2a** (2 equiv) **4a**

Entry	Variation from optimal conditions	Yield (%)[b]
1	No changes	91
2	[IPrAu][NTf_2] used instead	78
3	IPrAuOTf used instead	n.d.
4	JohnPhosAuNTf_2 used instead	n.d.
5	[(JohnPhos)Au(CH_3CN)]SbF_6 used instead	35%
6	[Cu(CH_3CN)$_4$]PF_6 used instead	n.d.
7	Ph_3AuCl/AgSbF_6 used instead	50
8	DCM used instead	65
9	Acetonitrile used instead	23
10	THF used instead	n.d.
11	Toluene used instead	n.d.
12	1 equiv used instead	82
13	70 ºC instead of 90 ºC	25
14	1 mol% used instead	69

[a] These experiments were performed on a 0.20 mmol scale. [b] Yield of the isolated product after chromatographic purification (silica gel; hexanes/ethyl acetate 40:1).

6. General Procedure for the Synthesis of Cyclopentadienes 4 (Method B)

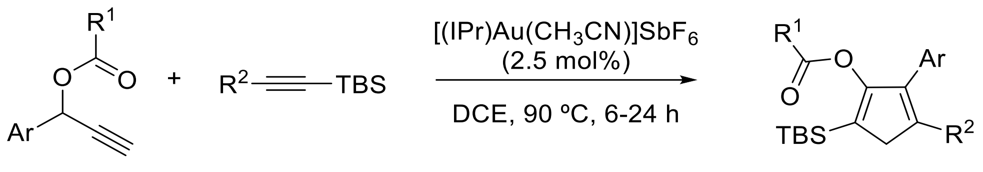

1 **2** **4**

$[(IPr)Au(CH_3CN)]SbF_6$ (4.3 mg, 2.5 mol%) was added to a solution of propargyl ester **1** (0.20 mmol) and alkynylsilane **2** (0.40 mmol, 2 equiv) in DCE (1 mL). The resulting mixture was stirred at 90 ºC until disappearance of the starting propargyl ester (checked by TLC, 2-24 h). Then, the solvent was removed under reduced pressure and the resulting mixture was purified by flash chromatography (silica gel, hexanes/ethyl acetate 40:1) to yield cyclopentadienes **4**.

7. Characterization Data of Cyclopentadienes 4

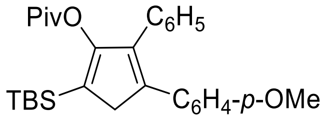

4a

2-(*tert*-butyldimethylsilyl)-4-(4-methoxyphenyl)-5-phenylcyclopenta-1,4-dien-1-yl pivalate (**4a**).

Method A: Cyclopentadiene **4a** was obtained from vinyl allene **3a** (92.5 mg, 0.20 mmol). After 2 hours, purification by flash chromatography (silica gel, hexanes/ethyl acetate 40:1) afforded compound **4a** (84.2 mg, 91% yield) as a yellow solid (m.p.: 113.9-115.2 ºC).

Method B: Alternatively, cyclopentadiene **4a** was obtained from propargyl ester **1a** (43.3 mg, 0.20 mmol) and alkynylsilane **2a** (98.6 mg, 0.40 mmol). After 20 hours, purification by flash chromatography (silica gel, hexanes/ethyl acetate 40:1) afforded compound **4a** (83.3 mg, 90% yield) as a yellow solid.

^{1}H NMR (300 MHz, $CDCl_3$): δ = 7.38-7.23 (m, 5H), 7.13 (d, *J* = 8.8 Hz, 2H), 6.72 (d, *J* = 8.8 Hz, 2H), 3.76 (s, 3H), 3.56 (s, 2H), 0.99 (s, 9H), 0.98 (s, 9H), 0.21 (s, 6H) ppm.

^{13}C NMR (75 MHz, $CDCl_3$): δ = 176.2 (C), 160.5 (C), 158.5 (C), 145.2 (C), 137.7 (C), 135.1 (C), 129.8 (CH), 128.9 (C), 128.4 (CH), 128.3 (CH), 127.2 (CH), 122.5 (C), 113.6 (CH), 55.1 (CH_3), 44.3 (CH_2), 38.6 (C), 27.1 (CH_3), 26.7 (CH_3), 17.7 (C), -5.3 (CH_3) ppm.

HRMS (EI) m/z: $[M + H]^+$ Calcd for $C_{29}H_{39}O_3Si$ 463.2663; Found 463.2658.

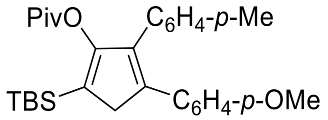

4b

2-(*tert*-butyldimethylsilyl)-4-(4-methoxyphenyl)-5-(*p*-tolyl)cyclopenta-1,4-dien-1-yl pivalate (**4b**).

Method A: Cyclopentadiene **4b** was obtained from vinyl allene **3b** (95.3 mg, 0.20 mmol). After 2 hours, purification by flash chromatography (silica gel, hexanes/ethyl acetate 40:1) afforded compound **4b** (94.4 mg, 99% yield) as a red solid (m.p.: 129.3-131.7 ºC).

Method B: Alternatively, cyclopentadiene **4b** was obtained from propargyl ester **1b** (46.1 mg, 0.20 mmol) and alkynylsilane **2a** (98.6 mg, 0.40 mmol). After 6 hours, purification by flash chromatography (silica gel, hexanes/ethyl acetate 40:1) afforded compound **4b** (63.9 mg, 67% yield) as a red solid.

^{1}H NMR (300 MHz, $CDCl_3$): δ = 7.17-7.14 (m, 6H), 6.73 (d, *J* = 8.8 Hz, 2H), 3.77 (s, 3H), 3.55 (s, 2H), 2.37 (s, 3H), 1.01 (s, 9H), 0.98 (s, 9H), 0.20 (s, 6H) ppm.

^{13}C NMR (75 MHz, $CDCl_3$): δ = 176.7 (C), 161.1 (C), 158.8 (C), 145.4 (C), 138.0 (C), 137.1 (C), 132.3 (C), 130.0 (CH), 129.5 (CH), 129.4 (C), 128.7 (CH), 122.8 (C), 113.9 (CH), 55.5 (CH_3), 44.6 (CH_2), 39.0 (C), 27.5 (CH_3), 27.1 (CH_3), 21.7 (CH_3), 18.1 (C), -4.9 (CH_3) ppm.

HRMS (EI) m/z: $[M + H]^+$ Calcd for $C_{30}H_{41}O_3Si$ 477.2819; Found 477.2819.

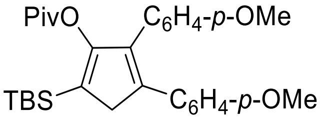

4c

2-(*tert*-butyldimethylsilyl)-4,5-bis(4-methoxyphenyl)cyclopenta-1,4-dien-1-yl pivalate (**4c**).

Method A: Cyclopentadiene **4c** was obtained) from vinyl allene **3c** (98.5 mg, 0.20 mmol). After 2 hours, purification by flash chromatography (silica gel, hexanes/ethyl acetate 40:1) afforded compound **4c** (95.6 mg, 97% yield) as a red oil.

Method B: Alternatively, cyclopentadiene **4c** was obtained from propargyl ester **1c** (49.3 mg, 0.20 mmol) and alkynylsilane **2a** (98.6 mg, 0.40 mmol). After 2 hours, purification by flash chromatography (silica gel, hexanes/ethyl acetate 40:1) afforded compound **4c** (52.2 mg, 53% yield) as a red oil.

^{1}H NMR (300 MHz, $CDCl_3$): δ = 7.15 (d, J = 8.8 Hz, 2H), 7.14 (d, J = 8.7 Hz, 2H), 6.89 (d, J = 8.8 Hz, 2H), 6.72 (d, J = 8.7 Hz, 2H), 3.83 (s, 3H), 3.76 (s, 3H), 3.52 (s, 2H), 1.02 (s, 9H), 0.96 (s, 9H), 0.16 (s, 6H) ppm.

^{13}C NMR (75 MHz, $CDCl_3$): δ = 176.7 (C), 161.1 (C), 159.1 (C), 158.7 (C), 145.5 (C), 137.6 (C), 131.3 (CH), 129.4 (C), 128.7 (CH), 127.6 (C), 122.8 (C), 114.2 (CH), 113.4 (CH), 55.6 (CH_3), 55.5 (CH_3), 44.6 (CH_2), 30.0 (C), 27.6 (CH_3), 27.1 (CH_3), 18.1 (C), -4.9 (CH_3) ppm.

HRMS (EI) m/z: $[M + H]^+$ Calcd for $C_{30}H_{41}O_4Si$ 493.2769; Found 493.2771.

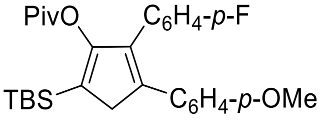

4d

2-(*tert*-butyldimethylsilyl)-5-(4-fluorophenyl)-4-(4-methoxyphenyl)cyclopenta-1,4-dien-1-yl pivalate (**4d**).

Method A: Cyclopentadiene **4d** was obtained from vinyl allene **3d** (96.1 mg, 0.20 mmol). After 2 hours, purification by flash chromatography (silica gel, hexanes/ethyl acetate 40:1) afforded compound **4d** (92.3 mg, 96% yield) as a yellow oil.

Method B: Alternatively, cyclopentadiene **4d** was obtained from propargyl ester **1d** (46.9 mg, 0.20 mmol) and alkynylsilane **2a** (98.6 mg, 0.40 mmol). After 24 hours, purification by flash chromatography (silica gel, hexanes/ethyl acetate 40:1) afforded compound **4d** (52.9 mg, 55% yield) as a yellow oil.

^{1}H NMR (300 MHz, $CDCl_3$): δ = 7.24-7.19 (m, 2H), 7.12-7.02 (m, 4H), 6.73 (d, *J* = 8.8 Hz, 2H), 3.77 (s, 3H), 3.54 (s, 2H), 1.02 (s, 9H), 0.97 (s, 9H), 0.20 (s, 6H) ppm.

^{13}C NMR (75 MHz, $CDCl_3$): δ = 176.7 (C), 162.5 (C, J_{C-F} = 244.6 Hz), 160.6 (C), 158.9 (C), 146.2 (C), 136.9 (C), 131.9 (CH, J_{C-F} = 7.9 Hz), 131.4 (C, J_{C-F} = 3.2 Hz), 129.1 (C), 128.8 (CH), 123.2 (C), 115. (CH, J_{C-F} = 20.9 Hz), 114.0 (CH), 55.6 (CH_3), 44.8 (CH_2), 39.0 (C), 27.5 (CH_3), 27.1 (CH_3), 18.1 (C), -4.9 (CH_3) ppm.

^{19}F NMR (282 MHz, $CDCl_3$): δ = -114.9 ppm.

HRMS (EI) m/z: $[M + Na]^+$ Calcd for $C_{29}H_{37}FNaO_3Si$ 503.2388; Found 503.2391.

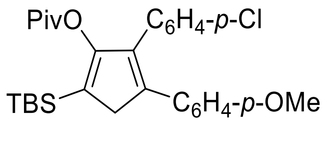

4e

2-(*tert*-butyldimethylsilyl)-5-(4-chlorophenyl)-4-(4-methoxyphenyl)cyclopenta-1,4-dien-1-yl pivalate (**4e**).

Method A: Cyclopentadiene **4e** was obtained from vinyl allene **3e** (99.4 mg, 0.20 mmol). After 2 hours, purification by flash chromatography (silica gel, hexanes/ethyl acetate 40:1) afforded compound **4e** (80.5 mg, 81% yield) as an orange yellow oil.

Method B: Alternatively, cyclopentadiene **4e** was obtained from propargyl ester **1e** (50.1 mg, 0.20 mmol) and alkynylsilane **2a** (98.6 mg, 0.40 mmol). After 24 hours, purification by flash chromatography (silica gel, hexanes/ethyl acetate 40:1) afforded compound **4e** (36.8 mg, 37% yield) as an orange oil.

^{1}H NMR (300 MHz, $CDCl_3$): δ = 7.33 (d, *J* = 8.4 Hz, 2H), 7.18 (d, *J* = 8.4 Hz, 2H), 7.10 (d, *J* = 8.9 Hz, 2H), 6.73 (d, *J* = 8.9 Hz, 2H), 3.77 (s, 3H), 3.53 (s, 2H), 1.02 (s, 9H), 0.95 (s, 9H), 0.19 (s, 6H) ppm.

^{13}C NMR (75 MHz, $CDCl_3$): δ = 176.7 (C), 160.3 (C), 159.0 (C), 146.4 (C), 136.7 (C), 133.9 (C), 133.5 (C), 131.6 (CH), 129.1 (CH), 128.9 (C), 128.8 (CH), 123.4 (C), 114.1 (CH), 55.6 (CH_3), 44.7 (CH_2), 39.0 (C), 27.7 (CH_3), 27.1 (CH_3), 18.1 (C), -4.9 (CH_3) ppm.

HRMS (EI) m/z: $[M + H]^+$ Calcd for $C_{29}H_{38}ClO_3Si$ 497.2273; Found 497.2277.

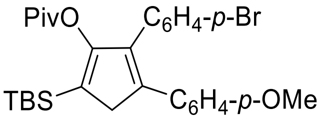

4f

5-(4-bromophenyl)-2-(*tert*-butyldimethylsilyl)-4-(4-methoxyphenyl)cyclopenta-1,4-dien-1-yl pivalate (**4f**).

Method A: Cyclopentadiene **4f** was obtained from vinyl allene **3f** (108.3 mg, 0.20 mmol). After 2 hours, purification by flash chromatography (silica gel, hexanes/ethyl acetate 40:1) afforded compound **4f** (104. mg, 96% yield) as an orange solid (m.p.: 125.2-127.1 ºC).

Method B: Alternatively, cyclopentadiene **4f** was obtained from propargyl ester **1f** (59.0 mg, 0.20 mmol) and alkynylsilane **2a** (98.6 mg, 0.40 mmol). After 24 hours, purification by flash chromatography (silica gel, hexanes/ethyl acetate 40:1) afforded compound **4f** (59.6 mg, 55% yield) as an orange solid.

^{1}H NMR (300 MHz, $CDCl_3$): δ = 7.48 (d, *J* = 8.4 Hz, 2H), 7.13-7.09 (m, 4H), 6.74 (d, *J* = 8.9 Hz, 2H), 3.77 (s, 3H), 3.55 (s, 2H), 1.03 (s, 9H), 0.96 (s, 9H), 0.18 (s, 6H) ppm.

^{13}C NMR (75 MHz, $CDCl_3$): δ = 176.7 (C), 160.2 (C), 159.0 (C), 146.4 (C), 136.7 (C), 134.4 (C), 132.0 (CH), 131.9 (CH), 128.9 (C), 128.8 (CH), 123.5 (C), 121.7 (C), 114.1 (CH), 55.6 (CH_3), 44.9 (CH_2), 39.0 (C), 27.6 (CH_3), 27.1 (CH_3), 18.1 (C), -4.9 (CH_3) ppm.

HRMS (EI) m/z: $[M + H]^+$ Calcd for $C_{29}H_{38}BrO_3Si$ 541.1768; Found 541.1770.

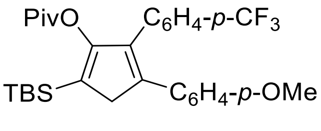

4g

2-(*tert*-butyldimethylsilyl)-4-(4-methoxyphenyl)-5-(4-(trifluoromethyl)phenyl)cyclopenta-1,4-dien-1-yl pivalate (**4g**).

Method A: Cyclopentadiene **4g** was obtained from vinyl allene **3g** (106.1 mg, 0.20 mmol). After 24 hours, purification by flash chromatography (silica gel, hexanes/ethyl acetate 40:1) afforded compound **4g** (60.5. mg, 57% yield) as an orange oil.

^{1}H NMR (300 MHz, $CDCl_3$): δ = 7.61 (d, *J* = 7.9 Hz, 2H), 7.37 (d, *J* = 7.9 Hz, 2H), 7.08 (d, *J* = 8.8 Hz, 2H), 6.74 (d, *J* = 8.8 Hz, 2H), 3.78 (s, 3H), 3.57 (s, 2H), 0.99 (s, 9H), 0.97 (s, 9H), 0.20 (s, 6H) ppm.

^{13}C NMR (75 MHz, $CDCl_3$): δ = 176.3 (C), 159.6 (C), 158.8 (C), 146.5 (C), 139.1, 136.2, 130.2 (CH), 128.5 (CH), 128.3 (C), 124.2 (C, q, J_{C-F} = 273.0 Hz), 125.3 (CH, J_{C-F} = 4.0 Hz), 123.4 (C), 113.8 (CH), 55.2 (CH_3), 44.6 (CH_2), 38.6 (C), 27.0 (CH_3), 26.7 (CH_3), 17.7 (C), -5.3 (CH_3) ppm.

^{19}F NMR (282 MHz, $CDCl_3$): δ = - 62.5 ppm.

HRMS (EI) m/z: $[M + H]^+$ Calcd for $C_{30}H_{38}F_3O_3Si$ 531.2537; Found 531.2540.

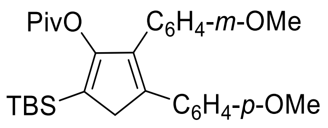

4h

2-(*tert*-butyldimethylsilyl)-5-(3-methoxyphenyl)-4-(4-methoxyphenyl)cyclopenta-1,4-dien-1-yl pivalate (**4h**).

Method A: Cyclopentadiene **4h** was obtained from vinyl allene **3h** (98.5 mg, 0.20 mmol). After 2 hours, purification by flash chromatography (silica gel, hexanes/ethyl acetate 40:1) afforded compound **4h** (84.7. mg, 86% yield) as an orange oil.

Method B: Alternatively, cyclopentadiene **4h** was obtained from propargyl ester **1h** (49.3 mg, 0.20 mmol) and alkynylsilane **2a** (98.6 mg, 0.40 mmol). After 4 hours, purification by flash chromatography (silica gel, hexanes/ethyl acetate 40:1) afforded compound **4h** (62.1 mg, 63% yield) as an orange solid.

^{1}H NMR (300 MHz, $CDCl_3$): δ = 7.29-7.23 (m, 1H), 7.16 (d, J = 8.8 Hz, 2H), 6.86-6.79 (m, 3H), 6.72 (d, J = 8.8 Hz, 2H), 3.77 (s, 3H), 3.76 (s, 3H), 3.55 (s, 2H), 1.01 (s, 9H), 0.98 (s, 9H), 0.20 (s, 6H) ppm.

^{13}C NMR (75 MHz, $CDCl_3$): δ = 176.7 (C), 160.8 (C), 159.9 (C), 158.9 (C), 145.7 (C), 137.8 (C), 136.7 (C), 129.9 (CH), 129.2 (C), 128.7 (CH), 123.0 (C), 122.6 (CH), 115.2 (CH), 114.0 (CH), 55.6 (CH_3), 55.1 (CH_3), 44.6 (CH_2), 39.0 (C), 27.5 (CH_3), 27.1 (CH_3), 18.1 (C), -4.9 (CH_3) ppm.

HRMS (EI) m/z: $[M + Na]^+$ Calcd for $C_{30}H_{40}NaO_4Si$ 515.2588; Found 515.2583.

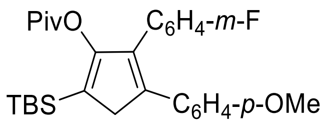

4i

2-(*tert*-butyldimethylsilyl)-5-(3-fluorophenyl)-4-(4-methoxyphenyl)cyclopenta-1,4-dien-1-yl pivalate (**4i**).

Method B: Cyclopentadiene **4i** was obtained from propargyl ester **1i** (46.9 mg, 0.20 mmol) and alkynylsilane **2a** (98.6 mg, 0.40 mmol). After 20 hours, purification by flash chromatography (silica gel, hexanes/ethyl acetate 40:1) afforded compound **4i** (56.7 mg, 59% yield) as a red oil.

^{1}H NMR (300 MHz, $CDCl_3$): δ = 7.34-7.27 (m, 1H), 7.12 (d, J = 8.8 Hz, 2H), 7.04-6.95 (m, 3H), 6.74 (d, J = 8.8 Hz, 2H), 3.78 (s, 3H), 3.55 (s, 2H), 1.03 (s, 9H), 0.97 (s, 9H), 0.21 (s, 6H) ppm.

^{13}C NMR (75 MHz, $CDCl_3$): δ = 176.3 (C), 162.8 (C, $J_{C\text{-}F}$ = 244.4 Hz), 159.9 (C), 158.7 (C), 146.1 (C), 137.3 (C, $J_{C\text{-}F}$ = 7.0 Hz), 136.3 (C), 129.9 (CH, $J_{C\text{-}F}$ = 33.3 Hz), 128.5 (C), 128.4 (CH), 125.7 (CH), 123.1 (C), 116.6 (CH, $J_{C\text{-}F}$ = 20.8 Hz), 114.1 (CH, $J_{C\text{-}F}$ = 83.0 Hz), 113.7 (CH), 113.5 (CH), 55.2 (CH_3), 44.5 (CH_2), 38.6 (C), 27.1 (CH_3), 26.7 (CH_3), 17.7 (C), -5.3 (CH_3) ppm.

^{19}F NMR (282 MHz, $CDCl_3$): δ = -113.3 ppm.

HRMS (EI) m/z: $[M + Na]^+$ Calcd for $C_{29}H_{37}FNaO_3Si$ 503.2388; Found 503.2392.

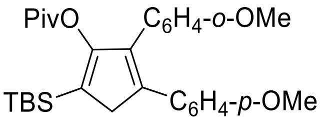

4j

2-(*tert*-butyldimethylsilyl)-5-(2-methoxyphenyl)-4-(4-methoxyphenyl)cyclopenta-1,4-dien-1-yl pivalate (**4j**).

Method A: Cyclopentadiene **4j** was obtained from vinyl allene **3i** (98.5 mg, 0.20 mmol). After 2 hours, purification by flash chromatography (silica gel, hexanes/ethyl acetate 40:1) afforded compound **4j** (70.0. mg, 71% yield) as a red oil.

Method B: Alternatively, cyclopentadiene **4j** was obtained from propargyl ester **1j** (49.3 mg, 0.20 mmol) and alkynylsilane **2a** (98.6 mg, 0.40 mmol). After 4 hours, purification by flash chromatography (silica gel, hexanes/ethyl acetate 40:1) afforded compound **4j** (62.1 mg, 63% yield) as a red solid.

^{1}H NMR (300 MHz, $CDCl_3$): δ = 7.32-7.26 (m, 1H), 7.16 (d, J = 8.9 Hz, 2H), 7.12-7.09 (m, 1H), 6.95-6.90 (m, 2H), 6.70 (d, J = 8.9 Hz, 2H), 3.75 (s, 3H), 3.72 (s, 3H), 6.62 (s, 1H), 3.55 (s, 1H), 0.96 (s, 9H), 0.94 (s, 9H), 0.20 (s, 3H), 0.17 (s, 3H) ppm.

^{13}C NMR (75 MHz, $CDCl_3$): δ = 176.3 (C), 161.4 (C), 158.7 (C), 158.0 (C), 145.7 (C), 134.7 (C), 132.1 (CH), 129.7 (C), 129.3 (CH), 128.1 (CH), 124.3 (C), 122.1 (C), 121.1 (CH), 113.9 (CH), 111.3 (CH), 55.9 (CH_3), 55.5 (CH_3), 44.6 (CH_2), 38.9 (C), 27.4 (CH_3), 27.2 (CH_3), 18.2 (C), -5.0 (CH_3) ppm.

HRMS (EI) m/z: $[M + H]^+$ Calcd for $C_{30}H_{41}O_4Si$ 493.2769; Found 493.2770.

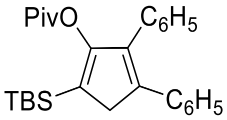

4k

2-(*tert*-butyldimethylsilyl)-4,5-diphenylcyclopenta-1,4-dien-1-yl pivalate (**4k**).

Method A: Cyclopentadiene **4k** was obtained from vinyl allene **3j** (86.5 mg, 0.20 mmol). After 8 hours, purification by flash chromatography (silica gel, hexanes/ethyl acetate 40:1) afforded compound **4k** (70.1. mg, 81% yield) as a colorless oil.

Method B: Alternatively, cyclopentadiene **4k** was obtained from propargyl ester **1a** (43.3 mg, 0.20 mmol) and alkynylsilane **2b** (86.6 mg, 0.40 mmol). After 24 hours, purification by flash chromatography (silica gel, hexanes/ethyl acetate 40:1) afforded compound **4k** (34.6 mg, 40% yield) as a colorless oil.

^{1}H NMR (300 MHz, $CDCl_3$): δ = 7.37-7.16 (m, 10H), 3.62 (s, 2H), 1.09 (s, 9H), 1.01 (s, 9H), 0.24 (s, 6H) ppm.

^{13}C NMR (75 MHz, $CDCl_3$): δ = 176.7 (C), 160.8 (C), 146.0 (C), 139.6 (C), 136.5 (C), 135.2 (C), 130.1 (CH), 128.8 (CH), 128.6 (CH), 127.7 (CH), 127.6 (CH), 127.2 (CH), 124.2 (C), 44.8 (CH_2), 39.0 (C), 27.5 (CH_3), 27.2 (CH_3), 18.1 (C), -4.9 (CH_3) ppm.

HRMS (EI) m/z: $[M + H]^+$ Calcd for $C_{28}H_{37}O_2Si$ 433.2557; Found 433.2557.

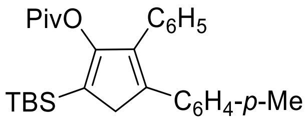

4l

2-(*tert*-butyldimethylsilyl)-5-phenyl-4-(*p*-tolyl)cyclopenta-1,4-dien-1-yl pivalate (**4l**).

Method A: Cyclopentadiene **4l** was obtained from vinyl allene **3k** (89.3 mg, 0.20 mmol). After 2 hours, purification by flash chromatography (silica gel, hexanes/ethyl acetate 40:1) afforded compound **4l** (69.7 mg, 78% yield) as an orange solid (m.p.: 126.4-128.3 ºC).

Method B: Alternatively, cyclopentadiene **4l** was obtained from propargyl ester **1a** (43.3 mg, 0.20 mmol) and alkynylsilane **2c** (92.2 mg, 0.40 mmol). After 17 hours, purification by flash chromatography (silica gel, hexanes/ethyl acetate 40:1) afforded compound **4l** (45.6 mg, 51% yield) as an orange solid.

Crystals of compound **4l** suitable for X-ray analysis were obtained by crystallization from pentane at -20 ºC.

^{1}H NMR (300 MHz, $CDCl_3$): δ = 7.35-7.23 (m, 5H), 7.09 (d, J = 8.0 Hz, 2H), 6.99 (d, J = 8.0 Hz, 2H), 3.57 (s, 2H), 2.29 (s, 3H), 0.99 (s, 9H), 0.98 (s, 9H), 0.21 (s, 6H) ppm.

^{13}C NMR (75 MHz, $CDCl_3$): δ = 176.7 (C), 160.8 (C), 146.0 (C), 138.9 (C), 137.0(C), 135.4 (C), 133.7 (C), 130.1 (CH), 129.3 (CH), 128.8 (CH), 127.6 (CH), 127.5 (CH), 123.6 (C), 44.7 (CH_2), 39.0 (C), 27.5 (CH_3), 27.1 (CH_3), 21.6 (CH_3), 18.1 (C), -4.9 (CH_3) ppm.

HRMS (EI) m/z: $[M + H]^+$ Calcd for $C_{29}H_{39}O_2Si$ 447.2714; Found 447.2714.

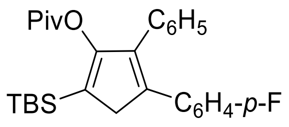

4m

2-(*tert*-butyldimethylsilyl)-4-(4-fluorophenyl)-5-phenylcyclopenta-1,4-dien-1-yl pivalate (**4m**).

Method A: Cyclopentadiene **4m** was obtained from vinyl allene **3l** (90.1 mg, 0.20 mmol). After 3 hours, purification by flash chromatography (silica gel, hexanes/ethyl acetate 40:1) afforded compound **4m** (78.4 mg, 87% yield) as an orange solid (m.p.: 117.7-119.1 ºC).

^{1}H NMR (300 MHz, $CDCl_3$): δ = 7.37-7.13 (m, 7H), 6.89-6.83 (m, 2H), 3.55 (s, 2H), 0.99 (s, 9H), 0.97 (s, 9H), 0.21 (s, 6H) ppm.

^{13}C NMR (75 MHz, $CDCl_3$): δ = 177.7 (C), 162.0 (C, $J_{C\text{-}F}$ = 245.0 Hz),, 160.6 (C), 144.8 (C), 139.4 (C), 135.5 (C), 132.6 (C), 130.0 (CH), 129.2 (CH, $J_{C\text{-}F}$ = 7.7 Hz),, 128.9 (CH), 127.8 (CH), 124.1 (C), 115.5 (CH, $J_{C\text{-}F}$ = 21.2 Hz), 44.8 (CH_2), 39.0 (C), 27.5 (CH_3), 27.1 (CH_3), 18.1 (C), -4.0 (CH_3) ppm.

^{19}F NMR (282 MHz, $CDCl_3$): δ = -115.0 ppm.

HRMS (EI) m/z: $[M + H]^+$ Calcd for $C_{28}H_{36}FO_2Si$ 451.2463; Found 451.2468.

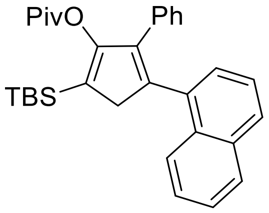

4n

2-(*tert*-butyldimethylsilyl)-4-(naphthalen-1-yl)-5-phenylcyclopenta-1,4-dien-1-yl pivalate (**4n**).

Method A: Cyclopentadiene **4n** was obtained from vinyl allene **3m** (96.5 mg, 0.20 mmol). After 3 hours, purification by flash chromatography (silica gel, hexanes/ethyl acetate 40:1) afforded compound **4n** (85.0 mg, 88% yield) as an orange oil.

^{1}H NMR (300 MHz, $CDCl_3$): δ = 7.81-7.72 (m, 3H), 7.43-7.31 (m, 5H), 7.11-7.04 (m, 4H), 3.59 (s, 2H), 1.11 (s, 9H), 1.00 (s, 9H), 0.23 (s, 6H) ppm.

^{13}C NMR (75 MHz, $CDCl_3$): δ = 176.3 (C), 159.5 (C), 146.3 (C), 141.0 (C), 135.7 (C), 134.0 (C), 133.5 (C), 131.7 (C), 128.8 (CH), 128.1 (CH), 127.7 (CH), 127.5 (CH), 126.9 (CH), 126.8 (CH), 126.1 (CH), 125.7 (CH), 125.6 (CH), 125.1 (CH), 124.6 (C), 48.0 (CH_2), 38.7 (C), 27.3 (CH_3), 26.8 (CH_3), 17.7 (C), -5.3 (CH_3) ppm.

HRMS (EI) m/z: $[M + Na]^+$ Calcd for $C_{32}H_{38}NaO_2Si$ 505.2533; Found 505.2532.

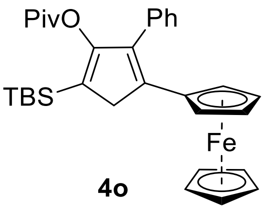

4o

2-(*tert*-butyldimethylsilyl)-4-(ferrocenyl)-5-phenylcyclopenta-1,4-dien-1-yl pivalate (**4o**).

Method A: Cyclopentadiene **4o** was obtained from vinyl allene **3n** (108.1 mg, 0.20 mmol). After 24 hours, purification by flash chromatography (silica gel, hexanes/ethyl acetate 40:1) afforded compound **4o** (104.9 mg, 97% yield) as a brown solid (m.p.: 126.3-128.2 ºC).

^{1}H NMR (300 MHz, $CDCl_3$): δ = 7.47-7.28 (m, 5H), 4.15-4.13 (m, 2H), 4.13-4.11 (m, 2H), 4.06 (s, 5H), 3.42 (s, 2H), 0.99 (s, 9H), 0.95 (s, 9H), 0.22 (s, 6H) ppm.

^{13}C NMR (75 MHz, $CDCl_3$): δ = 176.7 (C), 161.1 (C), 145.8 (C), 137.0 (C), 135.6 (C), 130.3 (CH), 128.5 (CH), 127.7 (CH), 121.2 (C), 80.6 (C), 69.9 (CH), 69.2 (CH), 67.3 (CH), 44.3 (CH_2), 39.0 (C), 27.4 (CH_3), 27.2 (CH_3), 18.3 (C), -4.9 (CH_3) ppm.

HRMS (EI) m/z: $[M]^+$ Calcd for $C_{32}H_{40}FeO_2Si$ 540.2147; Found 540.2156.

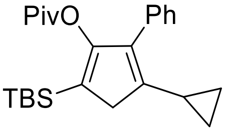

4p

2-(tert-butyldimethylsilyl)-4-cyclopropyl-5-phenylcyclopenta-1,4-dien-1-yl pivalate (**4p**).

Method A: Cyclopentadiene **4p** was obtained from vinyl allene **3o** (79.3 mg, 0.20 mmol). After 2 hours, purification by flash chromatography (silica gel, hexanes/ethyl acetate 40:1) afforded compound **4p** (61.1 mg, 77% yield) as an orange oil.

Method B: Alternatively, cyclopentadiene **4p** was obtained from propargyl ester **1a** (43.3 mg, 0.20 mmol) and alkynylsilane **2g** (72.1 mg, 0.40 mmol). After 4 hours, purification by flash chromatography (silica gel, hexanes/ethyl acetate 40:1) afforded compound **4p** (27.8 mg, 35% yield) as an orange oil.

^{1}H NMR (300 MHz, $CDCl_3$): δ = 7.39-7.26 (m, 5H), 2.80 (s, 2H), 1.83-1.80 (m, 1H), 1.02 (s, 9H), 0.92 (s, 9H), 0.86-0.79 (m, 2H), 0.68-0.62 (m, 2H), 0.13 (s, 6H) ppm.

^{13}C NMR (75 MHz, $CDCl_3$): δ = 176.6 (C), 160.2 (C), 151.3 (C), 138.4 (C), 134.7 (C), 129.9 (CH), 128.4 (CH), 127.2 (CH), 119.5 (C), 40.4 (C), 40.0 (CH_2), 27.6 (CH_3), 27.1 (CH_3), 18.1 (C), 12.1 (CH), 9.5 (CH_2), -4.9 (CH_3) ppm.

HRMS (EI) m/z: $[M + Na]^+$ Calcd for $C_{25}H_{36}NaO_2Si$ 419.2377; Found 419.2376.

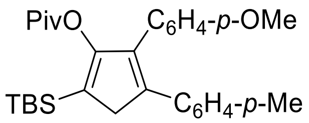

4q

2-(*tert*-butyldimethylsilyl)-5-(4-methoxyphenyl)-4-(*p*-tolyl)cyclopenta-1,4-dien-1-yl pivalate (**4q**).

Method A: Cyclopentadiene **4q** was obtained from vinyl allene **3p** (95.3 mg, 0.20 mmol). After 2 hours, purification by flash chromatography (silica gel, hexanes/ethyl acetate 40:1) afforded compound **4q** (90.6 mg, 95% yield) as an orange solid (m.p.: 102.5-104.1 ºC).

Method B: Alternatively, cyclopentadiene **4q** was obtained from propargyl ester **1c** (49.3 mg, 0.20 mmol) and alkynylsilane **2c** (92.2 mg, 0.40 mmol). After 4 hours, purification by flash chromatography (silica gel, hexanes/ethyl acetate 40:1) afforded compound **4q** (68.6 mg, 72% yield) as an orange solid.

^{1}H NMR (300 MHz, $CDCl_3$): δ = 7.17 (d, *J* = 8.3 Hz, 2H), 7.11 (d, *J* = 8.0 Hz, 2H), 7.00 (d, *J* = 8.0 Hz, 2H), 6.90 (d, *J* = 8.3 Hz, 2H), 3.84 (s, 3H), 3.56 (s, 2H), 2.30 (s, 3H), 1.05 (s, 9H), 0.98 (s, 9H), 0.22 (s, 6H) ppm.

^{13}C NMR (75 MHz, $CDCl_3$): δ = 176.7 (C), 161.1 (C), 159.2 (C), 145.9 (C), 138.5 (C), 136.9 (C), 133.8 (C), 131.2 (CH), 129.3 (CH), 127.5 (C), 127.4 (CH), 123.4 (C), 114.2 (CH), 55.6 (CH_3), 44.7 (CH_2), 39.1 (C), 27.6 (CH_3), 27.1 (CH_3), 21.6 (CH_3), 18.1 (C), -4.9 (CH_3) ppm.

HRMS (EI) m/z: $[M + H]^+$ Calcd for $C_{30}H_{41}O_3Si$ 477.2819; Found 477.2831.

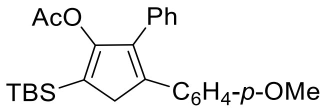

4r

2-(*tert*-butyldimethylsilyl)-4-(4-methoxyphenyl)-5-phenylcyclopenta-1,4-dien-1-yl acetate (**4r**).

Method A: Cyclopentadiene **4r** was obtained from vinyl allene **3q** (84.1 mg, 0.20 mmol). After 18 hours, purification by flash chromatography (silica gel, hexanes/ethyl acetate 40:1) afforded compound **4r** (58.9 mg, 70% yield) as a red solid (m.p.: 111.4-113.2 ºC).

Method B: Alternatively, cyclopentadiene **4r** was obtained from propargyl ester **1k** (34.8 mg, 0.20 mmol) and alkynylsilane **2a** (98.6 mg, 0.40 mmol). After 24 hours, purification by flash chromatography (silica gel, hexanes/ethyl acetate 40:1) afforded compound **4r** (50.5 mg, 60% yield) as an orange solid.

^{1}H NMR (300 MHz, $CDCl_3$): δ = 7.37-7.27 (m, 5H), 7.15 (d, *J* = 8.8 Hz, 2H), 6.74 (d, *J* = 8.8 Hz, 2H), 3.78 (s, 3H), 3.55 (s, 2H), 1.98 (s, 3H), 0.99 (s, 9H), 0.21 (s, 6H) ppm.

^{13}C NMR (75 MHz, $CDCl_3$): δ = 169.3 (C), 160.5 (C), 159.0 (C), 146.2 (C), 137.2 (C), 135.1 (C), 129.6 (CH), 129.3 (C), 129.1 (CH), 128.9 (CH), 127.6 (CH), 123.7 (C), 114.0 (CH), 55.6 (CH_3), 44.8 (CH_2), 27.1 (CH_3), 20.9 (CH_3), 18.1 (C), -5.1 (CH_3) ppm.

HRMS (EI) m/z: $[M + H]^+$ Calcd for $C_{26}H_{33}O_3Si$ 421.2193; Found 421.2193.

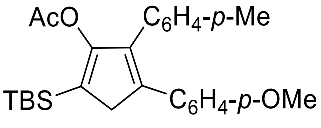

4s

2-(*tert*-butyldimethylsilyl)-4-(4-methoxyphenyl)-5-(p-tolyl)cyclopenta-1,4-dien-1-yl acetate (**4s**).

Method B: Cyclopentadiene **4s** was obtained from propargyl ester **1l** (37.6 mg, 0.20 mmol) and alkynylsilane **2a** (98.6 mg, 0.40 mmol). After 6 hours, purification by flash chromatography (silica gel, hexanes/ethyl acetate 40:1) afforded compound **4s** (43.5 mg, 50% yield) as an orange solid (m.p.: 94.3-96.6 ºC).

^{1}H NMR (300 MHz, $CDCl_3$): δ = 7.19-7.15 (m, 6H), 6.74 (d, *J* = 8.8 Hz, 2H), 3.78 (s, 3H), 3.54 (s, 2H), 2.38 (s, 3H), 1.98 (s, 3H), 0.99 (s, 9H), 0.21 (s, 6H) ppm.

^{13}C NMR (75 MHz, $CDCl_3$): δ = 169.4 (C), 160.7 (C), 158.9 (C), 145.8 (C), 137.2 (C), 137.1 (C), 132.0 (C), 129.6 (CH), 129.5 (C), 129.4 (CH), 129.0 (CH), 123.6 (C), 114.0 (CH), 55.6 (CH_3), 44.7 (CH_2), 27.1 (CH_3), 21.7 (CH_3), 21.0 (CH_3), 18.1 (C), -5.1 (CH_3) ppm.

HRMS (EI) m/z: $[M + H]^+$ Calcd for $C_{27}H_{35}O_3Si$ 435.235; Found 435.2351.

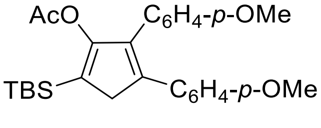

4t

2-(*tert*-butyldimethylsilyl)-4,5-bis(4-methoxyphenyl)cyclopenta-1,4-dien-1-yl acetate (**4t**).

Method B: Cyclopentadiene **4t** was obtained from propargyl ester **1m** (40.8 mg, 0.20 mmol) and alkynylsilane **2a** (98.6 mg, 0.40 mmol). After 3 hours, purification by flash chromatography (silica gel, hexanes/ethyl acetate 40:1) afforded compound **4t** (64.9 mg, 72% yield) as a yellow oil.

^{1}H NMR (300 MHz, $CDCl_3$): δ = 7.20 (d, *J* = 8.6 Hz, 2H), 7.17 (d, *J* = 8.6 Hz, 2H), 6.89 (d, *J* = 8.8 Hz, 2H), 6.75 (d, *J* = 8.8 Hz, 2H), 3.84 (s, 3H), 3.78 (s, 3H), 3.53 (s, 2H), 1.98 (s, 3H), 0.98 (s, 9H), 0.20 (s, 6H) ppm.

^{13}C NMR (75 MHz, $CDCl_3$): δ = 169.4 (C), 160.8 (C), 159.1 (C), 158.9 (C), 145.6 (C), 136.8 (C), 130.7 (CH), 129.5 (C), 129.0 (CH), 127.2 (C), 123.5 (C), 114.3 (CH), 114.0 (CH), 55.6 (CH_3), 55.5 (CH_3), 44.7 (CH_2), 27.1 (CH_3), 21.1 (CH_3), 18.1 (C), -5.1 (CH_3) ppm.

HRMS (EI) m/z: $[M + H]^+$ Calcd for $C_{27}H_{35}O_4Si$ 451.2299; Found 451.2297.

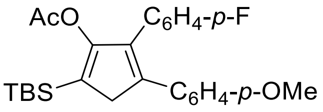

4u

2-(*tert*-butyldimethylsilyl)-5-(4-fluorophenyl)-4-(4-methoxyphenyl)cyclopenta-1,4-dien-1-yl acetate (**4u**).

Method B: Cyclopentadiene **4u** was obtained from propargyl ester **1n** (38.4 mg, 0.20 mmol) and alkynylsilane **2a** (98.6 mg, 0.40 mmol). After 24 hours, purification by flash chromatography (silica gel, hexanes/ethyl acetate 40:1) afforded compound **4u** (43.9 mg, 50% yield) as a red oil.

^{1}H NMR (300 MHz, $CDCl_3$): δ = 7.28-7.22 (m, 2H), 7.13 (d, *J* = 8.9 Hz, 2H), 7.07-7.01 (m, 2H), 6.75 (d, *J* = 8.9 Hz, 2H), 3.79 (s, 3H), 3.54 (s, 2H), 1.98 (s, 3H), 0.98 (s, 9H), 0.21 (s, 6H) ppm.

^{13}C NMR (75 MHz, $CDCl_3$): δ = 168.9 (C), 162.1 (C, $J_{C\text{-}F}$ = 244.9 Hz), 159.9 (C), 158.7 (C), 146.1 (C), 135.8 (C), 130.9 (CH, $J_{C\text{-}F}$ = 8.0 Hz), 130.6(C, $J_{C\text{-}F}$ = 3.5 Hz), 128.7 (CH), 128.6 (C), 123.5 (C), 115.5 (CH, $J_{C\text{-}F}$ = 21.2 Hz), 113.7 (CH), 55.2 (CH_3), 44.4 (CH_2), 26.7 (CH_3), 20.5 (CH_3), 17.7 (C), -5.5 (CH_3) ppm.

^{19}F NMR (282 MHz, $CDCl_3$): δ = - 114.7 ppm.

HRMS (EI) m/z: $[M + H]^+$ Calcd for $C_{26}H_{32}FO_2Si$ 439.2099; Found 439.2098.

8. ^{13}C Labelling Experiments

Preparation of [^{13}C]2a

Labelled alkynylsilane [^{13}C]**2a** was prepared according to a previous report by Zhang and coworkers.[4] CBr_4 (10% ^{13}C) was prepared by mixing 99.5 mg of CBr_4 (99% ^{13}C) with 895.4 mg of normal CBr_4 (1.1% ^{13}C). The required sequence is depicted in the following Scheme.

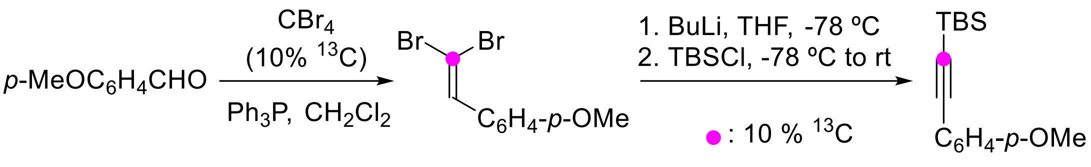

[^{13}C]**2a**

Synthesis of ^{13}C-labelled 1-(2,2-dibromovinyl)-4-methoxybenzene

CBr_4 (10% ^{13}C, 994.9 mg, 1equiv) and Ph_3P (1.5 g, 2 equiv) were combined in a flask, which was evacuated and refilled with nitrogen for three tiem. Then, CH_2Cl_2 (6 mL) was added and the resulting solution stirred for 15 minutes at room temperature. A solution of *p*-methoxybenzaldehyde (0.4 mL, 1 equiv) in CH_2Cl_2 (3 mL) was added at 0 ºC and the resulting mixture was stirred and allowed to slowly reach room temperature. The solvent was removed under reduced pressure and the resulting residue was purified by flash chromatography to give 1-(2,2-dibromovinyl)-4-methoxybenzene (377 mg, 45%) as a yellow oil which was used in the next step. Figures S2 and S3 show the ^{1}H and ^{13}C NMR spectra of ^{13}C-labelled 1-(2,2-dibromovinyl)-4-methoxybenzene.

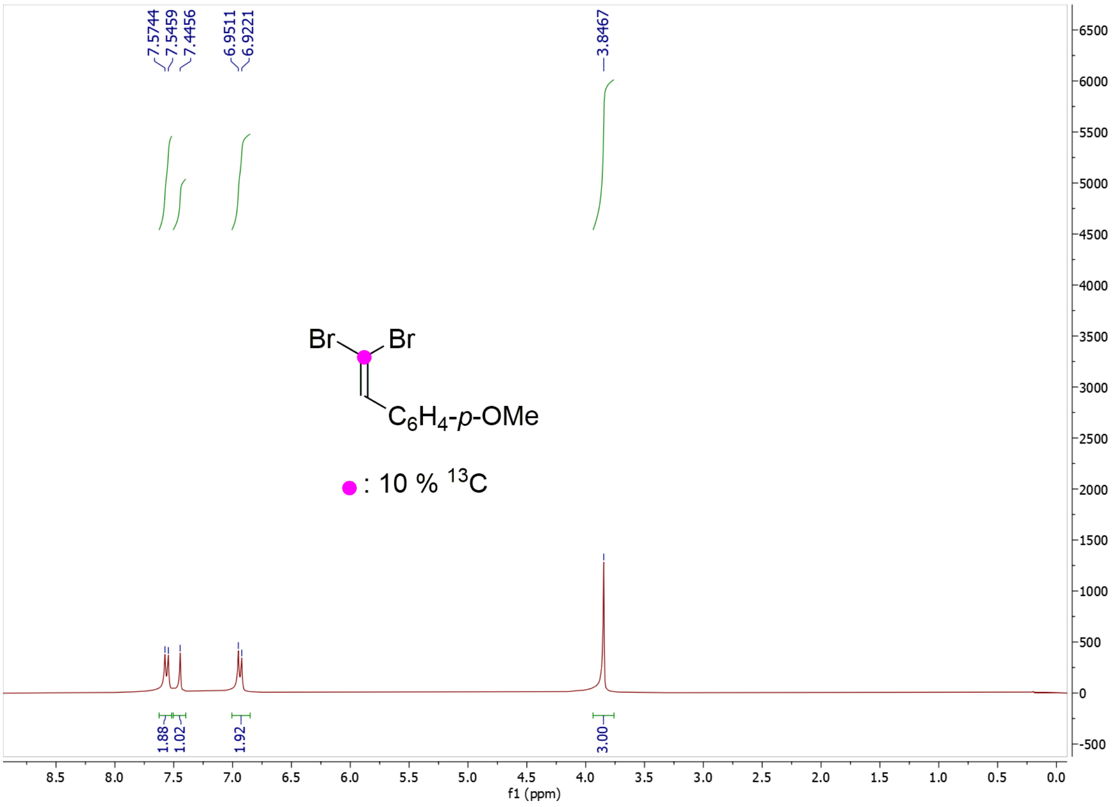

Figure S2. ^{1}H NMR spectrum ($CDCl_3$, 300 MHz) of ^{13}C-labelled 1-(2,2-dibromovinyl)-4-methoxybenzene.

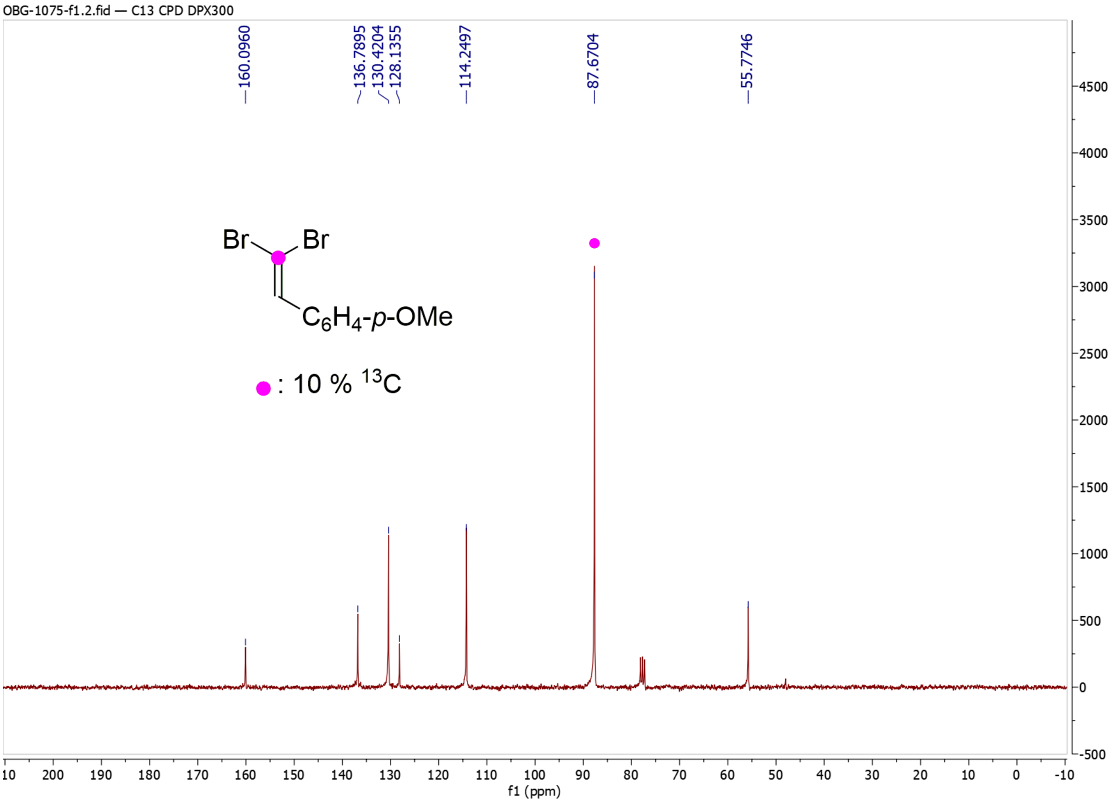

Figure S3. ^{13}C NMR spectrum ($CDCl_3$, 75 MHz) of ^{13}C-labelled 1-(2,2-dibromovinyl)-4-methoxybenzene.

*Synthesis of ^{13}C-labelled alkynylsilane [^{13}C]**2a***

To a solution of the 1-(2,2-dibromovinyl)-4-methoxybenzene (377 mg, 1.0 equiv) prepared in the previous step was added slowly BuLi (1.6 M in hexanes, 1.7 mL, 2.05 equiv) at -78 ºC. After stirring for 1 hour at this temperature, *tert*-butyldimethylsilyl chloride (215.5 mg, 1.1 equiv.) was added. The resulting mixture was stirred at -78 ºC for 1 hour, allowed to warm to room temperature for 1 additional hour. Then, saturated aqueous NH_4Cl (10 mL) was added, the layers were separated and the aqueous one extracted with diethylether (3 x 15 mL). The combined organic layers were washed with brine (15 mL), dried over Na_2SO_4, filtered, and concentrated under reduced pressure. The resulting residue was purified by column chromatography to give labelled alkynylsilane [^{13}C]**2a** as a yellow oil (125.8 mg, 40% yield). Figures S4 and S5 show the ^{1}H and ^{13}C NMR spectra of [^{13}C]**2a**.

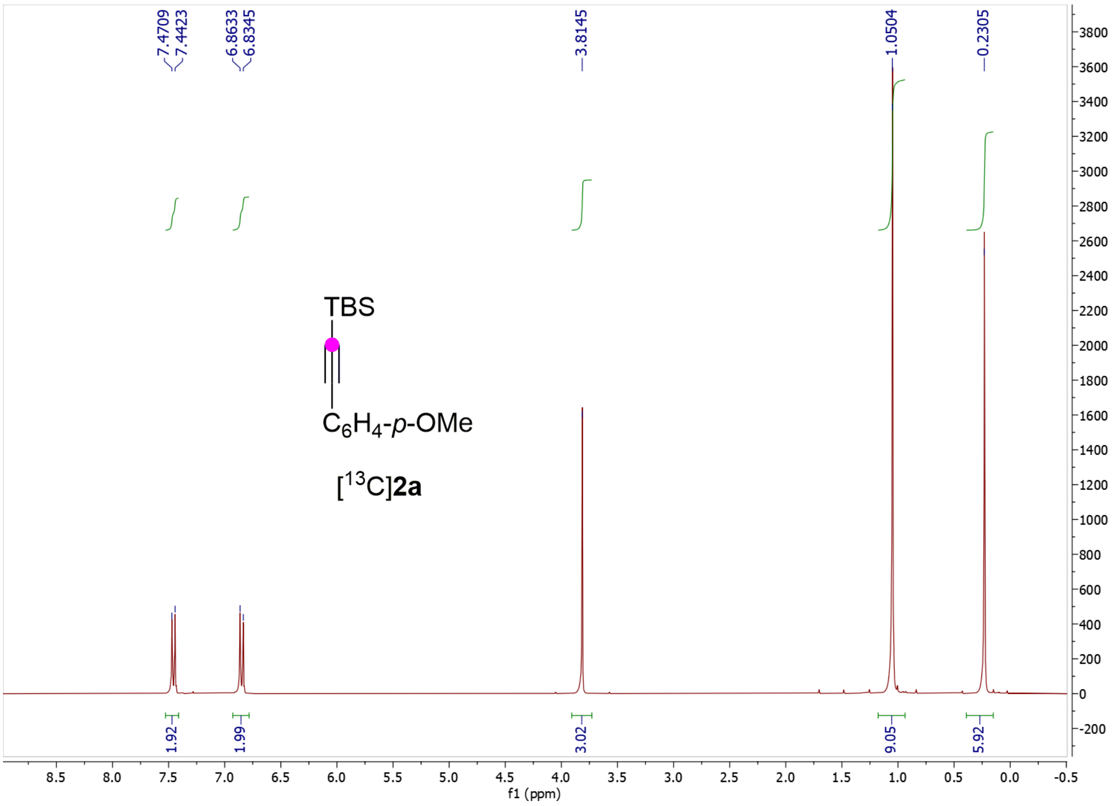

Figure S4. ^{1}H NMR spectrum ($CDCl_3$, 300 MHz) of ^{13}C-labelled [^{13}C]**2a**.

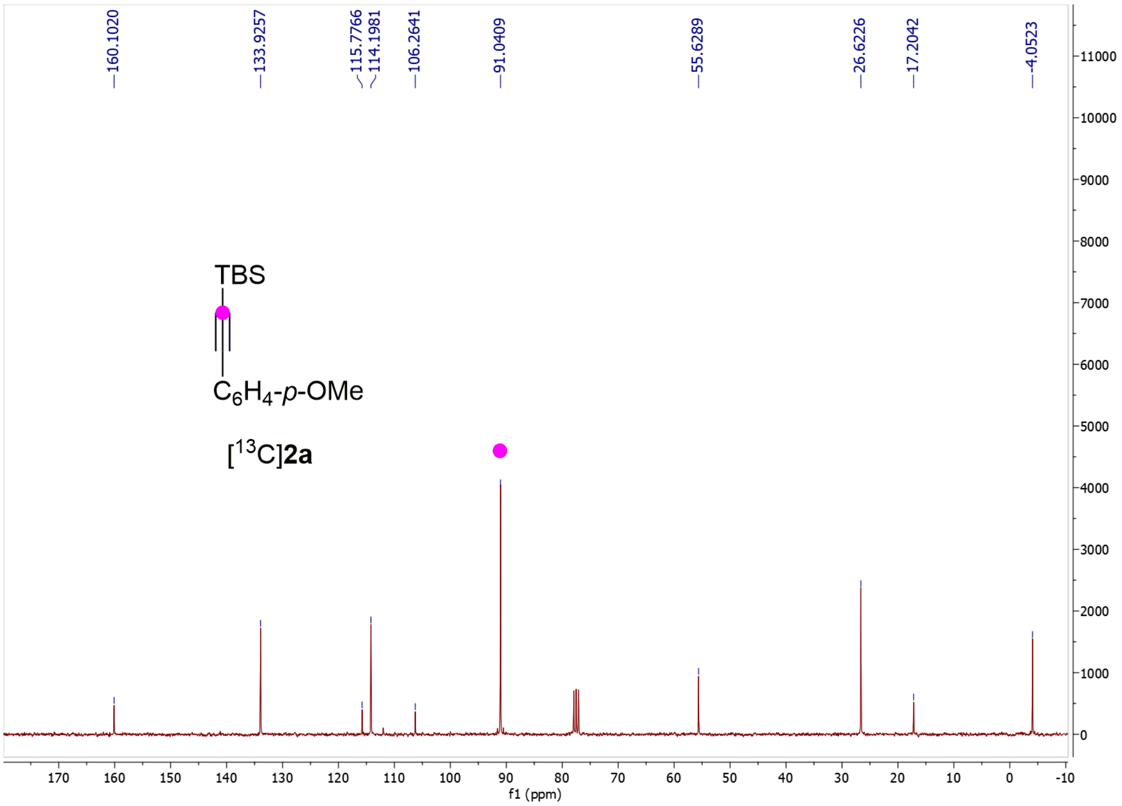

Figure S5. ^{13}C NMR spectrum ($CDCl_3$, 75 MHz) of ^{13}C-labelled [^{13}C]**2a**.

Synthesis of labelled vinylallene [^{13}C]3a

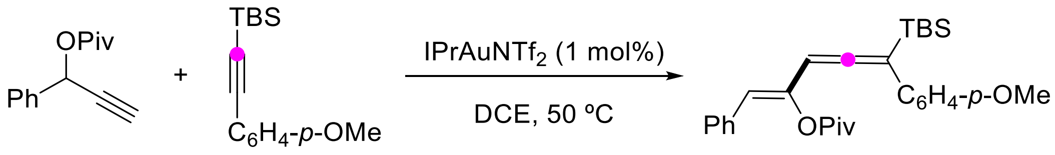

IPrAuNTf$_2$ (1.7 mg, 0.002 mmol, 1.0 mol%) was added to a solution of propargyl ester **1a** (43.3mg, 0.20 mmol) and alkynylsilane [^{13}C]**2a** (98.5 mg, 0.40 mmol, 2.0 equiv.) in 1,2-dichloroethane (DCE, 1 mL). The resulting mixture was stirred at 50 ºC until disappearance of **1** (checked by TLC). Then, the solvent was removed under reduced pressure and the resulting mixture was purified by flash chromatography (silica gel; hexanes/ethyl acetate 40:1) to yield allenylsilane [^{13}C]**3a** as a yellow oil (49.5 mg, 70% yield). The spectroscopic data of compound [^{13}C]**3a** match with those previously reported.[3] Figures S6 and S7 show the ^{1}H and ^{13}C NMR spectra of [^{13}C]**3a**.

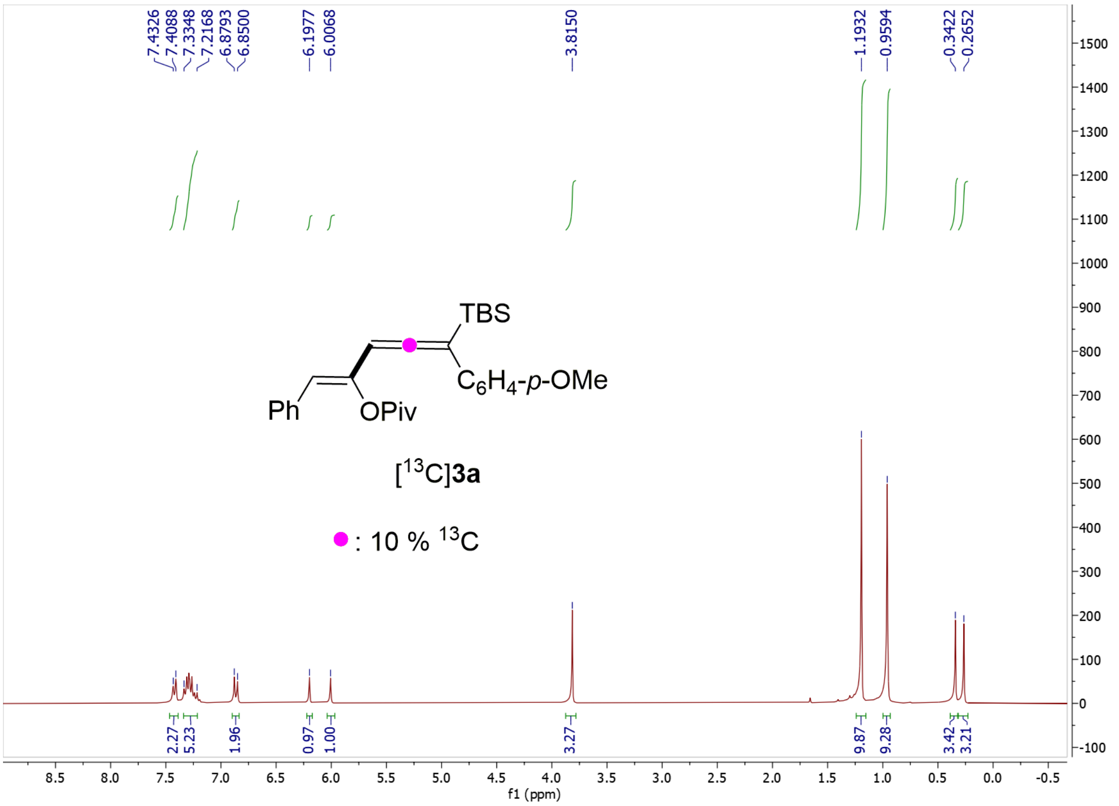

Figure S6. ^{1}H NMR spectrum ($CDCl_3$, 300 MHz) of [^{13}C]**3a**

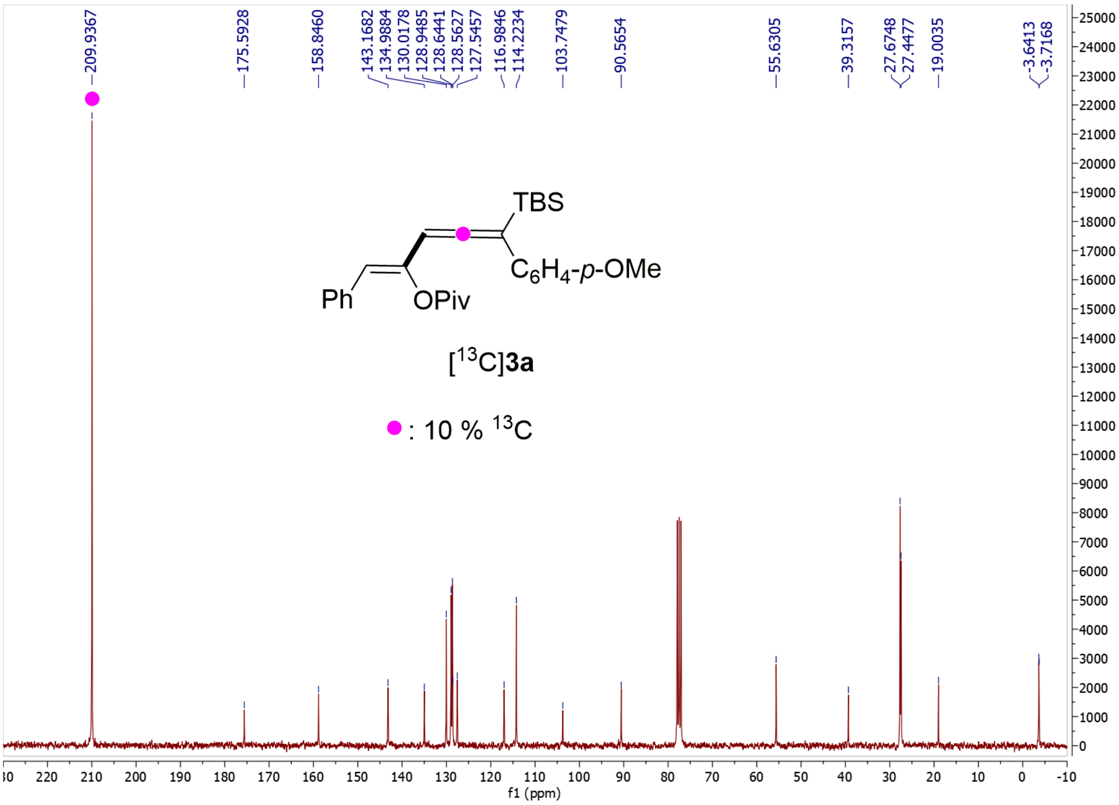

Figure S7. ^{13}C NMR spectrum ($CDCl_3$, 75 MHz) of [^{13}C]**3a**.

Synthesis of labelled cyclopentadiene [^{13}C]4a (Method A)

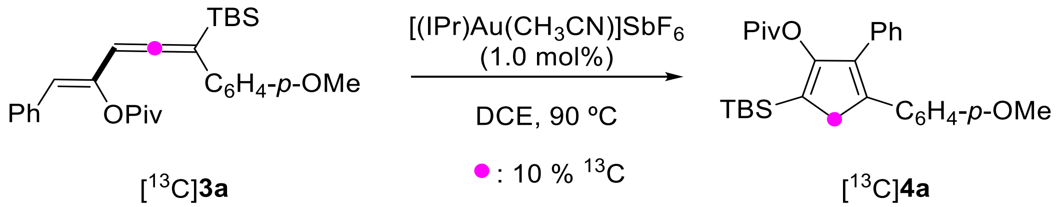

[(IPr)Au(CH_3CN)]SbF_6 (1.0 mg, 1.0 mol%) was added to a solution of vinyl allene [^{13}C]**3a** (49.5 mg, 0.1 mmol) in DCE (1 mL). The resulting mixture was stirred at 90 ºC until disappearance of the starting vinyl allene (checked by TLC, 2 h). Then, the solvent was removed under reduced pressure and the resulting mixture was purified by flash chromatography (silica gel, hexanes/ethyl acetate 40:1) to yield cyclopentadiene [^{13}C]**4a** as a yellow oil (40.3 mg, 87% yield). The spectroscopic data of cyclopentadiene [^{13}C]**4a** match with those reported for the unlabeled analogue **4a** (see page 15). Figures S8 and S9 show the ^{1}H and ^{13}C NMR spectra of [^{13}C]**4a**.

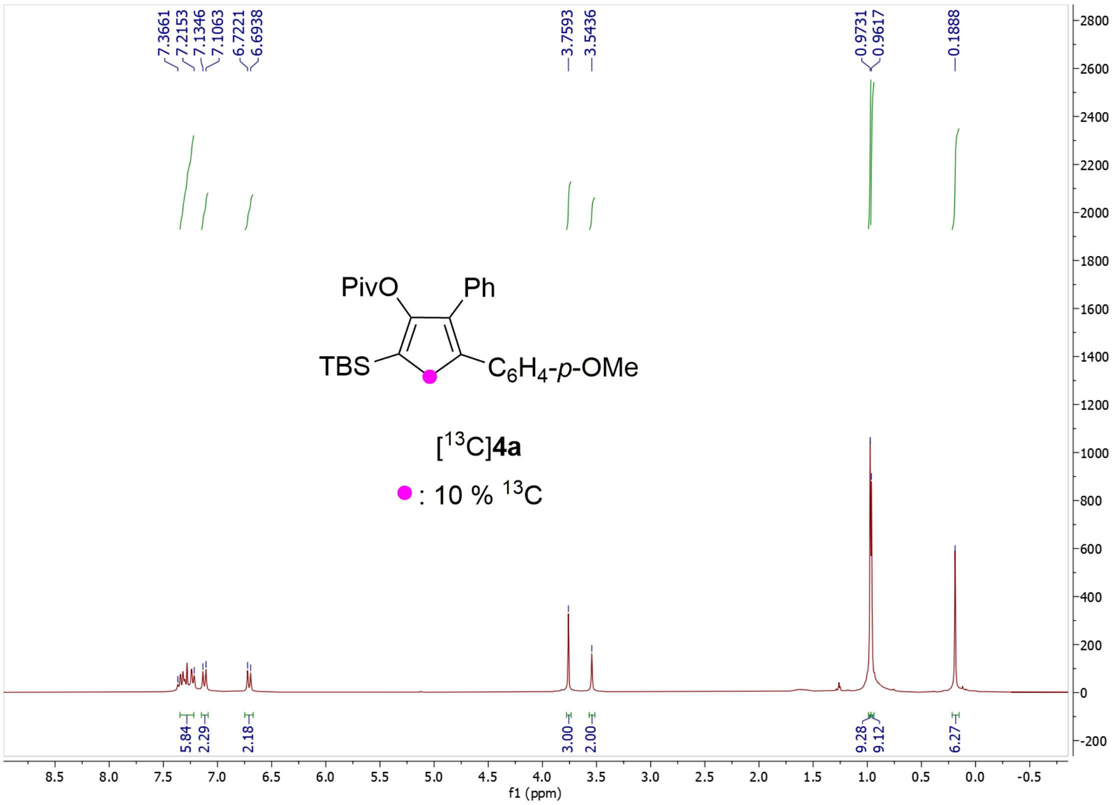

Figure S8. ^{1}H NMR spectrum ($CDCl_3$, 300 MHz) of [^{13}C]**4a**.

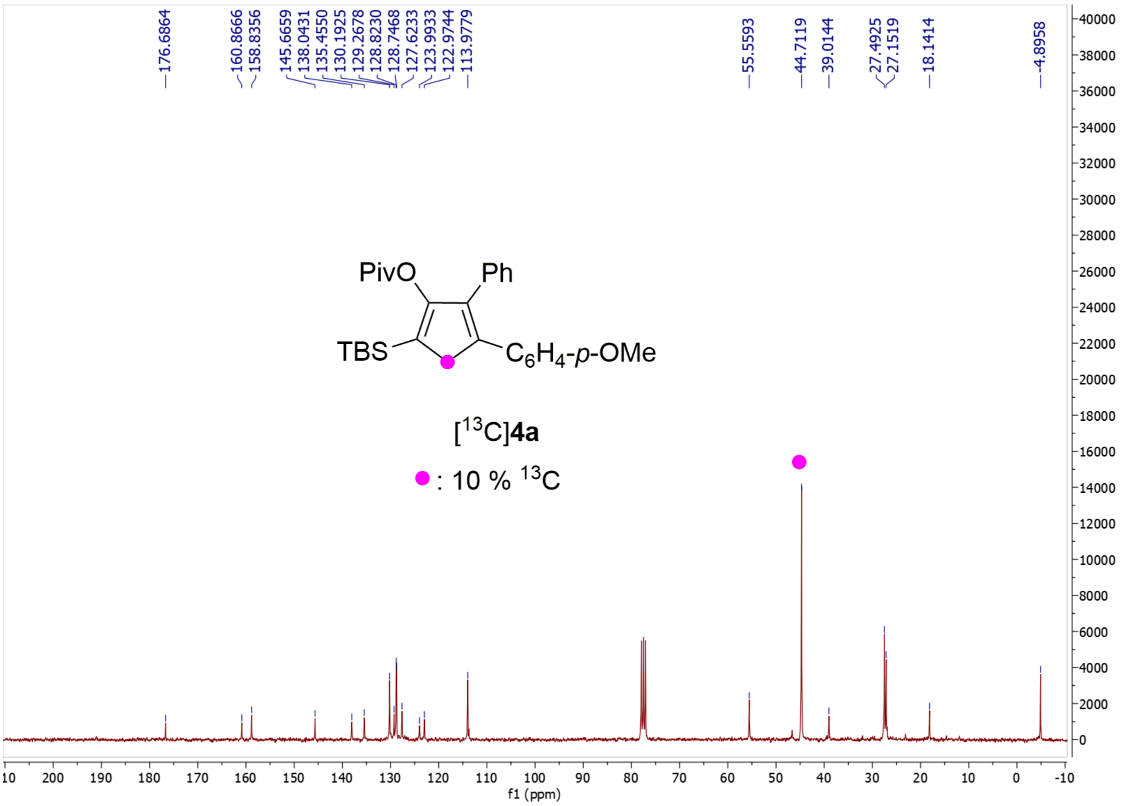

Figure S9. ^{13}C NMR spectrum ($CDCl_3$, 75 MHz) of [^{13}C]**4a**.

Synthesis of labelled cyclopentadiene [^{13}C]4a (Method B)

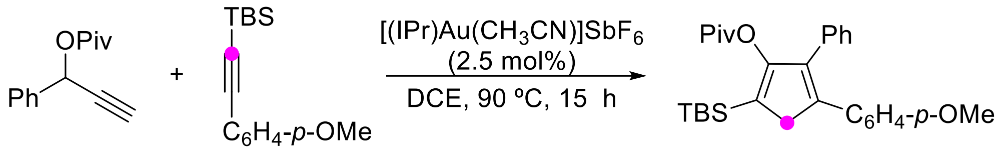

1a [^{13}C]**2a** : 10 % ^{13}C [^{13}C]**4a**

[(IPr)Au(CH_3CN)]SbF_6 (4.3 mg, 2.5 mol%) was added to a solution of propargyl ester **1a** (43.3 mg, 0.20 mmol) and alkynylsilane [^{13}C]**2a** (99.0 mg, 0.40 mmol, 2 equiv) in DCE (1 mL). The resulting mixture was stirred at 90 ºC until disappearance of the starting propargyl ester (checked by TLC, 15 h). Then, the solvent was removed under reduced pressure and the resulting mixture was purified by flash chromatography (silica gel, hexanes/ethyl acetate 40:1) to yield labelled cyclopentadiene [^{13}C]**4a** as a yellow oil (76.8 mg, 83% yield). The spectroscopic data of [^{13}C]**4a** match with those found when prepared according to Method A (see Figures S8 and S9).

9. Computational Details

The proposed mechanism for the gold-catalyzed cycloisomerization of vinylallenes **3** is depicted in Scheme S1. Coordination of the cationic gold complex to the vinylallene **3** would generate a pentadienyl cation intermediate **I**, that would undergo an electrocyclization reaction leading to carbene species **II**. Selective 1,2-migration of the silyl group would give rise to the intermediate **III**, which upon demetalation would deliver cyclopentadiene intermediate **IV**. The formation of the final cyclopentadienes **4** could be explained through a series of thermally allowed suprafacial [1,5]-sigmatropic shifts of hydrogen and ^{t}BuMe$_2$Si group.

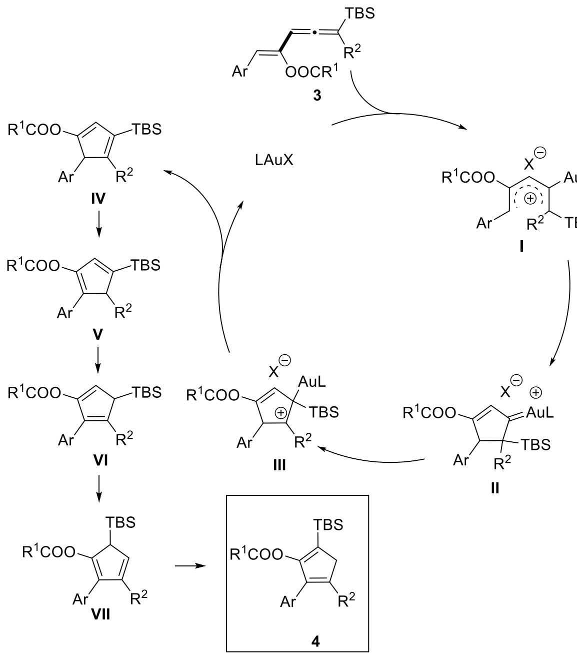

Scheme S1. Proposed reaction mechanism for the gold-catalyzed cycloisomerization of vinylalenes **3**

To gain more insight into the mechanism of the isomerization reactions of these cyclopentadiene derivatives we carried out density-functional theory (DFT) calculations on the potential-energy surface corresponding to the transformation of intermediate **IVk** (R^1 = ^{t}Bu, Ar = R^2 = Ph) into the final cyclopentadiene derivative **4k**.

Computational Methodology

The potential energy surfaces of the reactions considered in this work were explored with the density-functional theory (DFT), using the hybrid functional B3LYP,[5] which has been employed previously to study pericyclic reactions,[6] including several kinds of [1,5] sigmatropic shifts.[7] The 6-31G(d) basis set was employed, the use of a larger basis being precluded due to the size of the systems considered.

The geometry of each stationary point located was fully optimized in all cases and each stationary point was characterized to be a minimum or a first-order saddle point (transition structure) by computing the harmonic vibrational frequencies at 298.150 ºK and 1.0 atm. The connection of either, the reactants or products with the corresponding transition structure was established by computation of the intrinsic reaction coordinate (IRC). The Cartesian coordinates and energies of the stationary points located are collected at the end of this computational section.

All the calculations described in this paper work were carried out with the *Gaussian09* suite of programs.[8]

Reaction Path for the Formation of Cyclopentadiene derivative 4k.

The computational study of the reactions leading from the cyclopentadiene intermediate **IVk** to the derivative **4k**, has allowed to understand the mechanistic details of these transformations. The reaction intermediates and the transition states located for this reaction path are outlined in Scheme S2.

According to the computational results, eleven possible cyclopentadiene derivatives (**IVk** to **XIIIk**, see Scheme S2), and the corresponding transition states for their isomerization reactions were found.

The cyclopentadiene intermediates found, can be collected in two groups, as it is shown in Scheme S2: the first group, involves the intermediates **IVk**, **Vk**, **VIk**, **XIk** and **XIIk**, while the second one includes the intermediates **VIIk** to **XIIIk** and the reaction product **4k**. These intermediates could be interconverted by a thermally-allowed, suprafacial [1,5]-hydrogen sigmatropic shift.

On the other hand, intermediate **VIk** could experience a [1,5]-^{t}butyldimethylsilyl sigmatropic shift, leading to two different cyclopentadiene intermediates, **VIIk** and **XIIIk**. This suprafacial [1,5]-silyl shift connects the two previously described groups of cyclopentadiene intermediates.

The formation of the product **4k** (the experimentally observed cyclopentadiene derivative), can be explained by a reaction pathway involving the intermediates **IVk** to **VIIk**, and the transition states **TS1** to **TS4**. This mechanistic pathway is highlighted (blue color) in Scheme S1.

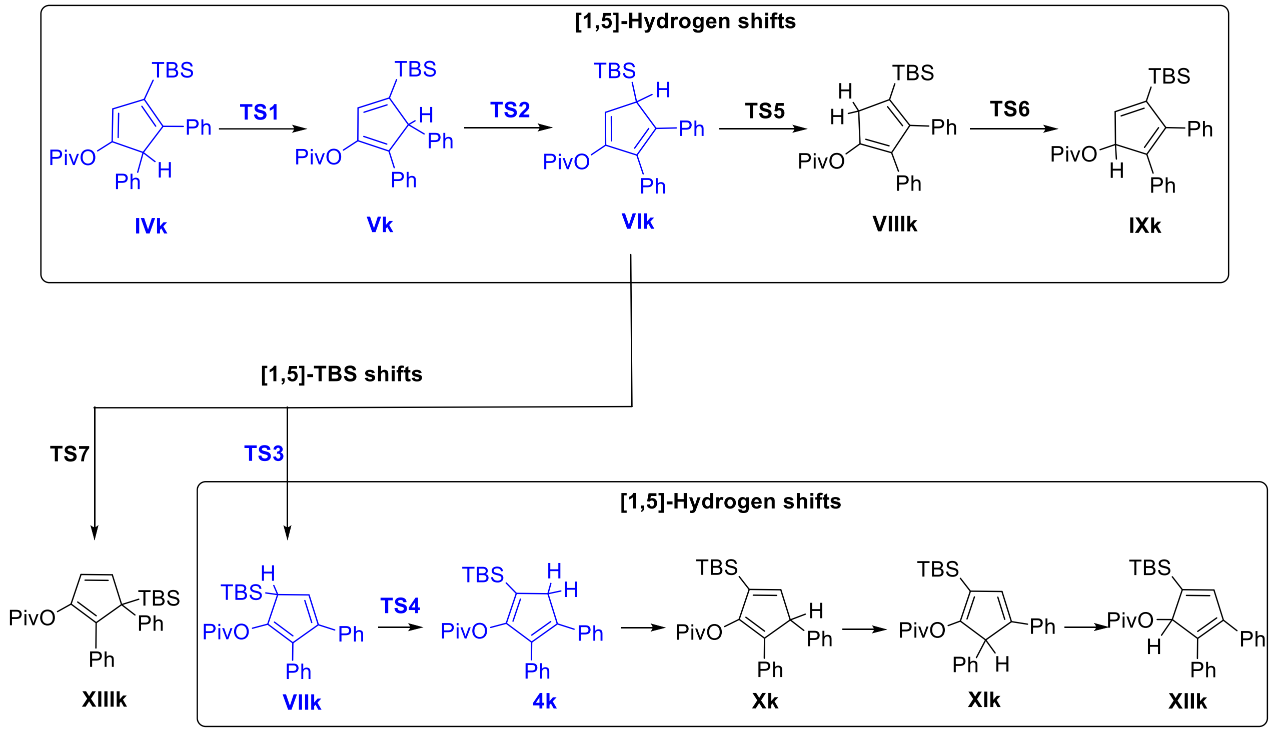

Scheme S2. Computational study of the formation of cyclopentadiene derivative **4k**. The proposed reaction path for the transformation of intermediate **IVk** into **4k** is shown in blue color.

Structures and Relative Energies of Isomeric Cyclopenta-1,3-dienes.

The structures of all the cyclopentadiene derivatives considered in this work, and previously shown in Scheme S2, are also collected in Figure S10 for an easy reference.

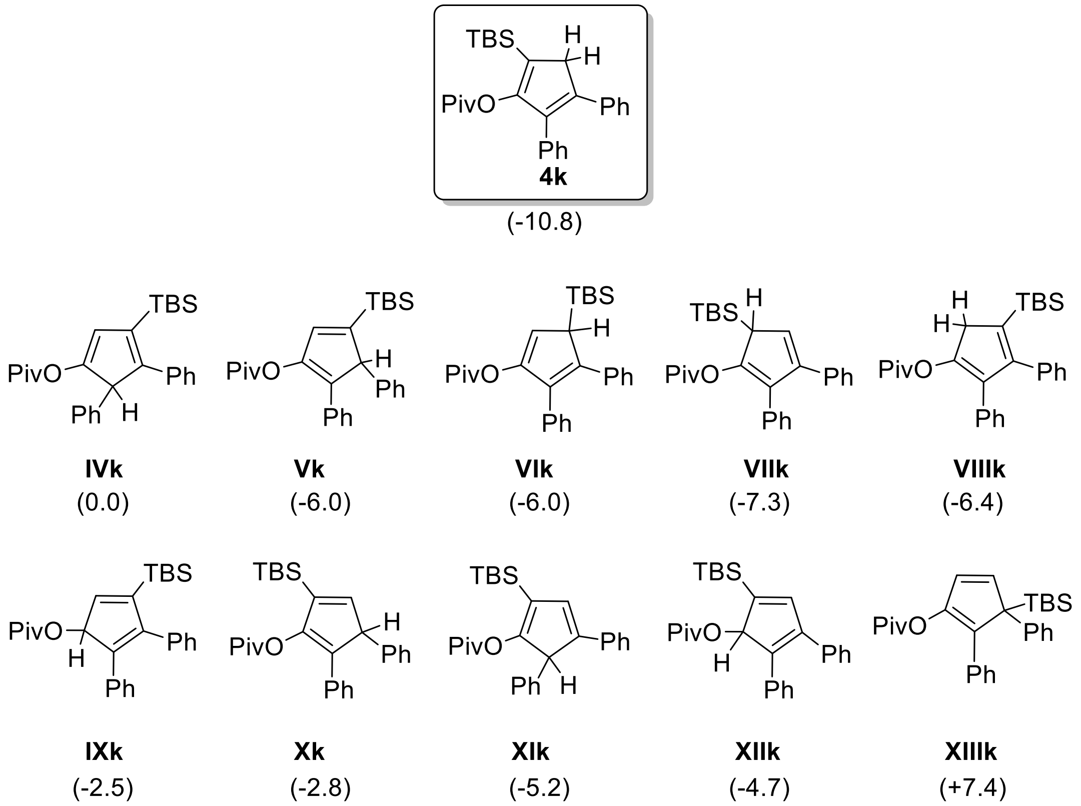

Figure S10. Structure and relative free-energies (in parenthesis, kcal mol^{-1}) of cyclopenta-1,3-diene derivatives considered in this work. The final product **4k**, is drawn in the square.

The optimized geometries and the most salient geometrical features of the cyclopentadiene derivatives, are presented in Figures S11 and S12.

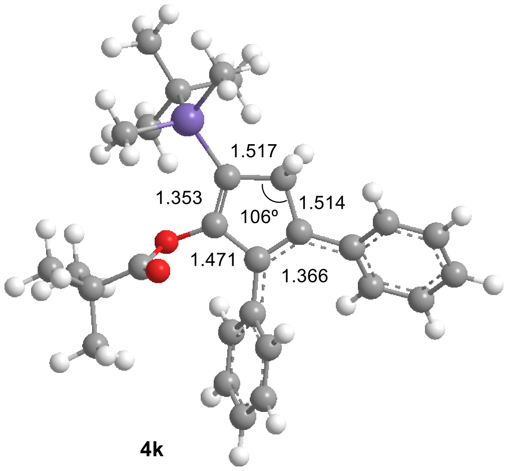

4k

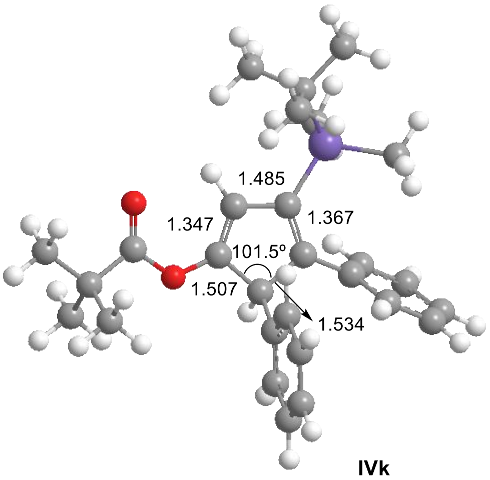

IVk

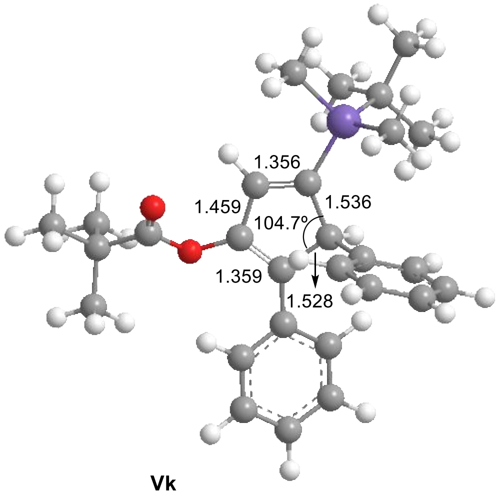

Vk

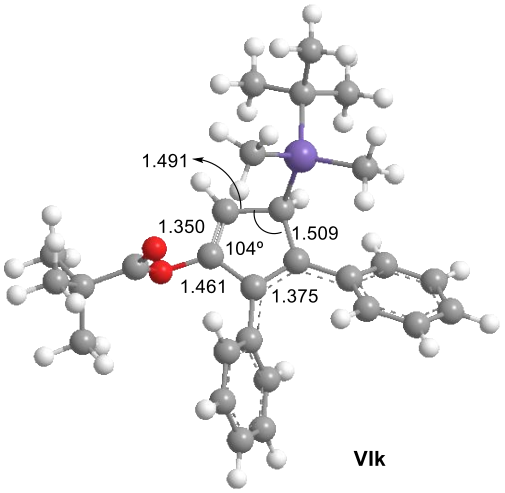

VIk

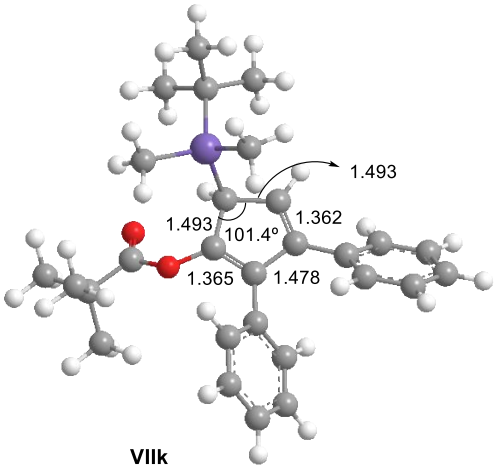

VIIk

Figure S11. Selected geometrical parameters of cyclopentadienes **4k** and **IVk**-**VIIk**. Lengths are in Å.

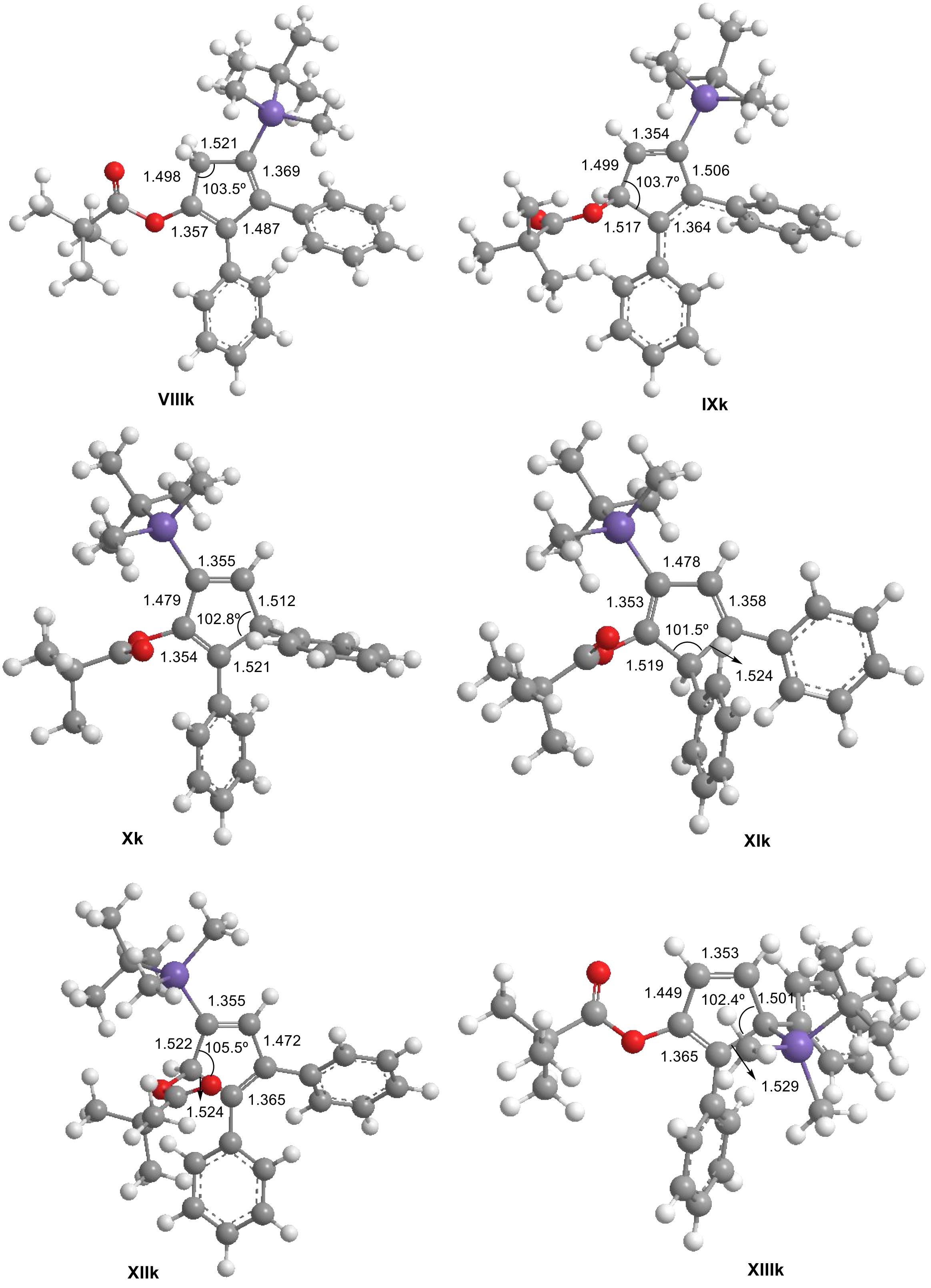

Figure S12. Selected geometrical parameters of cyclopentadienes **VIIIk**-**XIIIk**. Lengths are in Å.

The carbon atoms of the five-membered ring of the cyclopentadiene derivatives are lying in the same plane. In each structure, three different sets of C-C bond lengths can be found. One group includes the longest values (in the range of 1.5 Å), and corresponds to the two bonds between

the sp^3 and two sp^2 hybridized carbon atoms. There is another group, with the shortest bond lengths, corresponding to the formal C-C double bonds (lengths about 1.3 Å), and finally one group, which corresponds to the bond between *C3* and *C4*, sp^2- hybridized carbon atoms, having an intermediate value in the range of 1.4 Å.

From the relative values of the free-energies shown in Figure S13, it can be concluded that the cyclopentadiene intermediates **Vk**, **VIk** and **VIIIk** are significantly more stable than the demetalated intermediate **IVk**. According to these values, cyclopentadiene **4k** is predicted to be the thermodynamic control product. The less stable compound is the intermediate **XIIIk**, in which two bulky groups –Ph and TBS- are bonded to the sp^3 hybridized carbon atom.

Isomerization of Cyclopenta-1,3-dienes through Suprafacial [1,5]-hydrogen Shifts.

According to the mechanism proposed to account for the formation of **4k**, cyclopentadiene derivative **IVk**, first formed after the demetalation of intermediate **III** could undergo several suprafacial [1,5] sigmatropic shifts of hydrogen atom or ^{t}butyldimethylsilyl group (Scheme S1), which, according to the Woodward-Hoffmann rules, are thermally allowed.[9]

In the case of the migration of a hydrogen atom, as can be seen in Scheme S1, two groups of suprafacial [1,5] sigmatropic shifts were found. The potential-energy surface corresponding to the sequential isomerization reactions of cyclopentadiene intermediates **IVk**, **Vk**, **VIk**, **VIIIk**, **IXk**, of the first group, is shown in Figure S13.

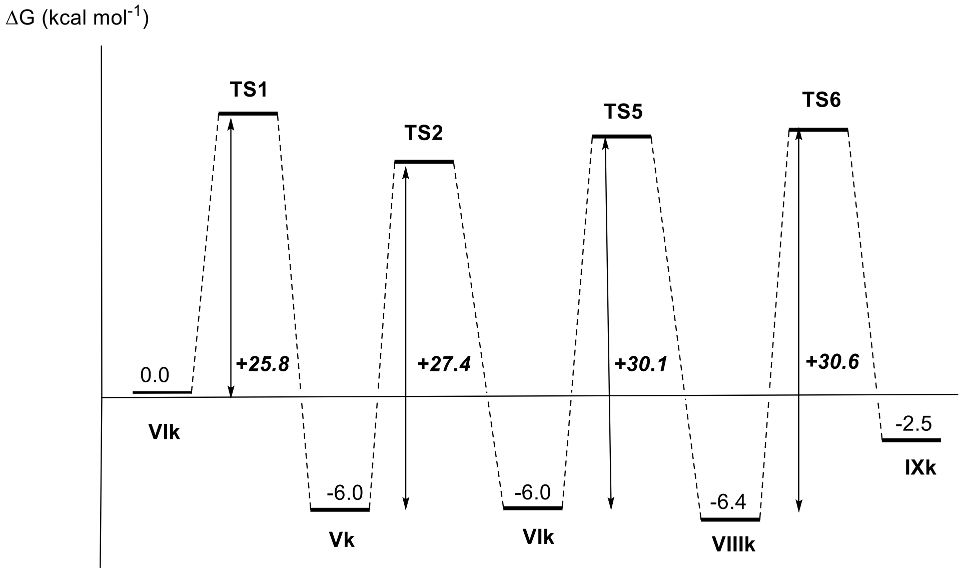

Figure S13. Reaction profile corresponding to the sequential isomerization of cyclopentadiene derivatives through [1,5]-hydrogen sigmatropic shifts

Four transition states, **TS1**, **TS2**, **TS5** and **TS6**, were located for these sigmatropic shifts (Figure S14). The activation free-energies are predicted to be in the range of 25.8 to 30.6 kcal mol^{-1}, values which are quite close to those found in previous studies for related reactions.[7] The imaginary vibrational normal mode of these transition states corresponds to the C-H bond breaking/bond forming process.

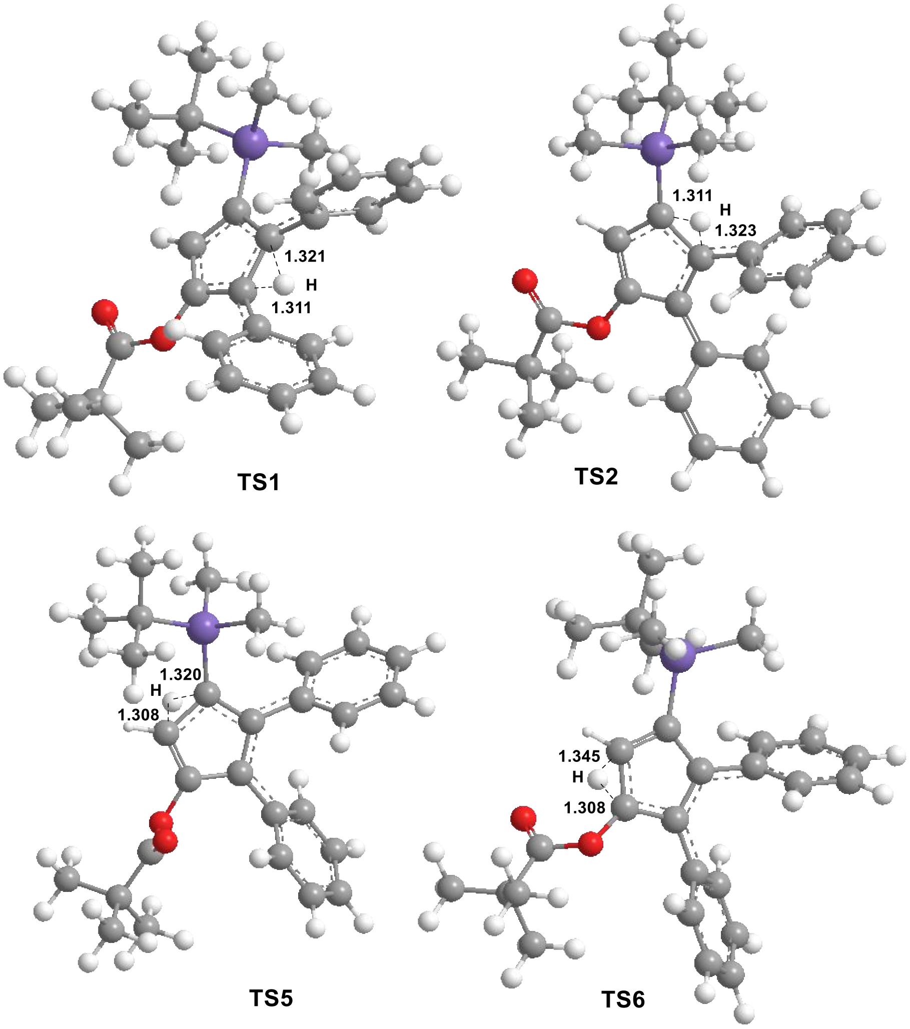

Figure S14. Transition states located for the suprafacial [1,5]-hydrogen shifts. Lengths are in Å.

In these transition states, all the carbon atoms of the five-membered ring lie in a plane and the C-C bond lengths are quite similar. The migrated hydrogen atom lies out of the plane formed by the carbon atoms, and it is simultaneously bound to a two adjacent carbon atoms, the asynchrony being small. As can be seen in Figure S14, all the CH bond lengths in the four transition states are quite close, with values about 1.3 Å. The geometries of these transition states are in good agreement with those reported earlier in simpler systems.[7]

On the other hand, as shown in Scheme S2, the second group of [1,5]-hydrogen shifts, involves the final product **4k** and the cyclopentadiene intermediates **VIIk**, **Xk**, **XIk** and **XIIk**. Transition state **TS4** (Figure S15), corresponding to the [1,5]-hydrogen shift leading from intermediate **VIIk** to the final product **4k**, was also located. The geometrical characteristics of **TS4** and the imaginary vibrational normal mode are similar to those of the transition states previously described. The value of the activation barrier for this reaction is predicted to be 28.3 kcal mol^{-1}.

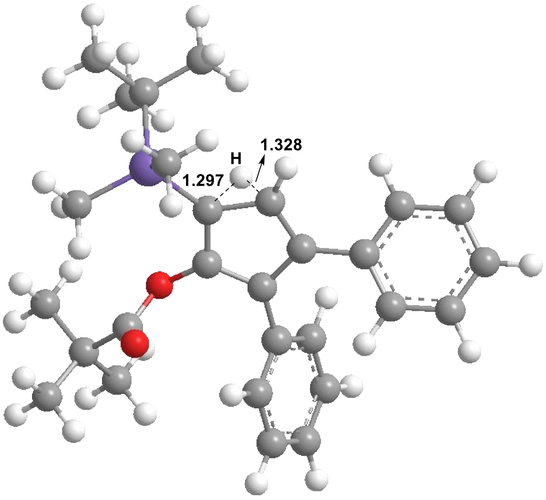

TS4

Figure S15. Transition state, **TS4**, corresponding to the suprafacial [1,5]-hydrogen shift leading to the final product **4k**. Lengths are in Å.

Suprafacial [1,5]-sylil Sigmatropic Shifts: Isomerization of Intermediate VIk.

The reaction paths proposed for the transformation of intermediate **IVk** into final product **4k**, involves a thermally allowed, sigmatropic [1,5]- ^{t}butyldimethylsilyl shifts, through transition state **TS3**, from intermediate **VIk** to give the cyclopentadiene derivative **VIIk**. Alternatively, intermediate **VIk** could undergo also a sigmatropic shift, through **TS7**, to give intermediate **XIIIk** (Scheme S2).

The salient geometry details of **TS3** and **TS7** and the corresponding potential energy surface for the isomerization of **VIk** to give both possible intermediates are shown in Figure S16. Transition states **TS3** and **TS7**, as in the previously reported cases of [1,5] sigmatropic shifts, are not very asynchronous, the two C-Si bond lengths being quite close. The imaginary vibrational normal mode in both transition states corresponds to the C-Si bond breaking/bond forming process.

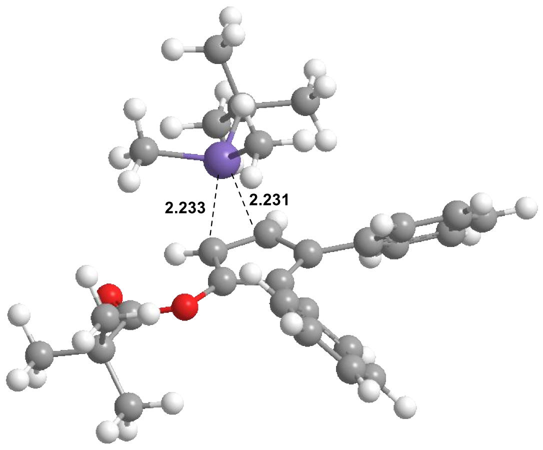

TS3

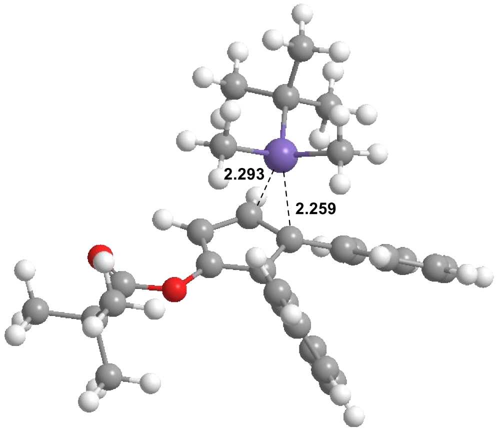

TS7

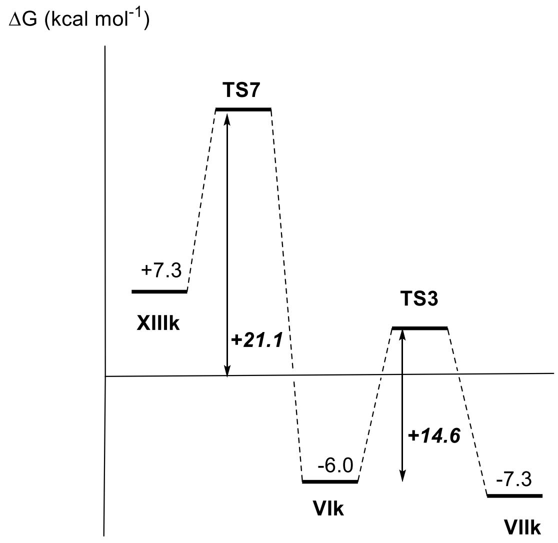

Figure S16. Transition states, **TS4** and **TS7** corresponding to the suprafacial [1,5]-TBS shift leading to the intermediates **VIIk** or **XIIIk**. Lengths are in Å.

As it has been previously reported,[10] the activation barrier of the thermally-allowed [1,5]-silyl sigmatropic shifts (**VIk** to **VIIk**, or **VIk** to **XIIIk**) is smaller than the barrier found for the analogous reaction involving a hydrogen atom.

The current computations indicate that the favored migration of the ^{t}butyldimethylsilyl group leads from intermediate **VIk**, through transition state **TS3**, to intermediate **VIIk**, which is both, the kinetic and thermodynamic product.

Cartesian Coordinates and Total and Free-Energies of the Stationary Points located at the B3LYP/6-31G(d) Level of Theory

**4k**	**IVk**
C,0,-1.1561786139,1.3589269416,-1.3150656353	C,0,-0.7439800661,-0.2285850131,-0.402761181
C,0,-0.4492495807,0.5692411193,-0.4748593534	C,0,0.1287497682,0.730311431,-0.7687070419
C,0,-0.1103032056,2.0977114851,-2.1281123923	C,0,-1.2214743431,0.04539109,0.9762314852
Si,0,-3.0117361186,1.616286521,-1.4879203108	Si,0,-2.4562247168,-1.0701915424,1.9070841229
O,0,-0.9976679218,-0.3227358961,0.4446433804	O,0,0.8546655802,0.9482528253,-1.9176997215
C,0,1.2307155511,1.5885076128,-1.6428289573	C,0,-0.6129608848,1.1854967348,1.4203454942
C,0,1.0075507268,0.6782223603,-0.6494569331	C,0,0.3251867635,1.7342323159,0.3373054529
C,0,2.4973543279,2.0234473767,-2.2519616106	C,0,-0.8493651009,1.9503147227,2.66918807
C,0,1.9837743837,-0.0772860318,0.169329972	C,0,1.779167153,1.9226592477,0.7507537667
C,0,2.6264862527,3.3262395506,-2.7710372067	C,0,-2.0891453547,2.5778662455,2.8846319131
C,0,3.8091891747,3.7481287111,-3.3754986495	C,0,-2.3306489446,3.317261853,4.0432591712
C,0,4.8935236936,2.8761444731,-3.4837384155	C,0,-1.3314998125,3.4520797494,5.0083016302
C,0,4.7794796325,1.5767838075,-2.9839478102	C,0,-0.0907224077,2.8455528008,4.8027362267
C,0,3.5988772942,1.1541845798,-2.3782153215	C,0,0.1510753149,2.1067138369,3.6446012983
C,0,1.9560336207,-1.481557968,0.1977478383	C,0,2.3472483144,3.1989987293,0.8102852989
C,0,2.8751663828,-2.1967454044,0.9676297988	C,0,3.6781956255,3.3715462872,1.1990632287
C,0,3.8286327424,-1.5194148392,1.7301732504	C,0,4.4584504613,2.2652907037,1.53269373
C,0,3.8575186712,-0.1229238056,1.7178176706	C,0,3.9002187902,0.9847286825,1.4741637584
C,0,2.9431908984,0.5920589548,0.9460510636	C,0,2.5724373364,0.8152052798,1.0861286053
C,0,-3.8735086165,1.5212915415,0.19774153	C,0,-2.1001743242,-1.0277532333,3.7686736935
C,0,-3.7596328208,0.3049144338,-2.6935067651	C,0,-2.3244726373,-2.9066446768,1.3011749432
C,0,-3.2618588624,3.3644329488,-2.1852717654	C,0,-4.2205864438,-0.4390266796,1.5968645326
C,0,-1.4553316204,-1.0675588812,2.7110909345	C,0,1.8015646082,0.5606924628,-4.0727326888
C,0,-3.5878687611,-1.1118086469,-2.1062347104	C,0,-0.8507314235,-3.3618577503,1.2260316975
C,0,-3.0450827384,0.3623867305,-4.0602837835	C,0,-2.9961574961,-3.1058339158,-0.0756804049
C,0,-5.2638339528,0.5826460429,-2.9037406025	C,0,-3.0574214292,-3.817574385,2.314369622
C,0,-0.960340156,0.0382481913,1.7708040463	C,0,0.8635929724,0.0665879965,-2.9672838199
O,0,-0.5695671506,1.1200135817,2.1387372803	O,0,0.2144369146,-0.9524111137,-2.9886701852
C,0,-0.2495815764,-1.4651364584,3.5955279452	C,0,1.7600456374,-0.4447199196,-5.23421219
C,0,-1.993805977,-2.300927844,1.9688718418	C,0,3.2358064943,0.6615502404,-3.5045013213
C,0,-2.5586221267,-0.4533652897,3.6004543996	C,0,1.3313593508,1.9517576668,-4.5544139028
H,0,-0.2209848884,1.9357925396,-3.2100481201	H,0,-1.0530087371,-1.0569226976,-1.0181792298
H,0,-0.207205857,3.1833385334,-1.9793893252	H,0,-0.0646617032,2.7114054947,0.0136616139
H,0,1.7966488623,4.0219326882,-2.6862115769	H,0,-2.8626890517,2.4905743914,2.1266193277
H,0,3.8833974961,4.7620067204,-3.7602753214	H,0,-3.297983511,3.7915894099,4.1880434785
H,0,5.8158714492,3.2035743156,-3.9558594757	H,0,-1.5167176168,4.027184549,5.9116913586
H,0,5.612333691,0.8840162528,-3.073168319	H,0,0.6933752372,2.9441428909,5.5491241778
H,0,3.5187144679,0.1363098504,-2.0129214402	H,0,1.116086191,1.631901415,3.5008933701
H,0,1.2180262372,-2.012395416,-0.3972260383	H,0,1.743411609,4.0660711799,0.5531658242
H,0,2.8473371381,-3.2834287816,0.9684403706	H,0,4.1018994486,4.3716281331,1.2400418156
H,0,4.5429008642,-2.075576918,2.3314776384	H,0,5.4941526215,2.3966639653,1.8344111041
H,0,4.5913256722,0.4119794851,2.3149331568	H,0,4.5016064569,0.1162550462,1.7303731081
H,0,2.9602896089,1.6775354799,0.9446963065	H,0,2.1413295781,-0.1818269869,1.0406886198
H,0,-3.3430755868,2.1329850402,0.9360993094	H,0,-2.140728716,-0.0106268945,4.1669946725
H,0,-3.9165104777,0.4973481808,0.5834223212	H,0,-1.1079801856,-1.4333275417,3.9994221365
H,0,-4.9027440316,1.893746925,0.1297811395	H,0,-2.8390396687,-1.628776064,4.3115016431
H,0,-4.3265999078,3.5898531345,-2.3157231866	H,0,-4.9689404614,-1.1206471673,2.0194554629
H,0,-2.7738081137,3.5024638612,-3.1569345785	H,0,-4.434454895,-0.3311013657,0.5274522782
H,0,-2.8509931056,4.1148570312,-1.4990144955	H,0,-4.3658508037,0.5405076825,2.0658735808
H,0,-4.1363840073,-1.234647935,-1.1645462485	H,0,-0.3408381018,-3.2493613569,2.1913703698
H,0,-2.5353967539,-1.3480844312,-1.9110358369	H,0,-0.2785068874,-2.799849196,0.4802237106
H,0,-3.9735553509,-1.8664528069,-2.8076013438	H,0,-0.7936639459,-4.4253875329,0.9507862717
H,0,-1.9756666813,0.1372708145,-3.9694488451	H,0,-2.5452841312,-2.4945512968,-0.864248142
H,0,-3.1439844716,1.3448331052,-4.5386777213	H,0,-4.0659642699,-2.8670258645,-0.0448522046
H,0,-3.4768851705,-0.3783911451,-4.749321804	H,0,-2.9063894805,-4.1554461875,-0.3922689951
H,0,-5.4424729916,1.5640619769,-3.3599811601	H,0,-4.1092796864,-3.5336009277,2.4469039829
H,0,-5.8257816256,0.5437585478,-1.9624495877	H,0,-2.5805740568,-3.8061795833,3.3006481456
H,0,-5.7018042448,-0.1712446613,-3.5745176955	H,0,-3.0462323255,-4.8584384472,1.9591034668
H,0,0.1480291799,-0.5925093586,4.1216883302	H,0,0.7496524211,-0.5371925352,-5.6441804542
H,0,0.5579551686,-1.90080224,2.9974495736	H,0,2.0769671854,-1.4401102148,-4.9088996467
H,0,-0.5663835829,-2.207373684,4.3374231492	H,0,2.4298142557,-0.1121946245,-6.0351509631
H,0,-1.2250519448,-2.7606771183,1.3410852266	H,0,3.5923663522,-0.3145077989,-3.1556863415
H,0,-2.8443078397,-2.0495984843,1.3272398059	H,0,3.2866709018,1.3637779578,-2.6679732299
H,0,-2.3289356432,-3.0469036127,2.6989483495	H,0,3.9180811571,1.007018988,-4.2898486375
H,0,-3.4385177102,-0.1743237738,3.0096959357	H,0,1.3624603507,2.6864334807,-3.7450984067
H,0,-2.1912711321,0.4419500099,4.1093052314	H,0,0.3079494949,1.9121711433,-4.9452469221
H,0,-2.8746682694,-1.1820030245,4.3555325472	H,0,1.9855352741,2.3006278836,-5.3617126305
**G(au)= -1528.145442**	**G(au)= -1528.128294**
**HF(au)= -1528.6489039**	**HF(au)= -1528.6320413**

Vk

C,0,0.0591606263,-1.4025343365,0.1950133075
C,0,1.1628178753,-0.4932863097,-0.0946870572
C,0,-1.1265288353,-0.755933698,0.0695924686
Si,0,-2.8018175454,-1.5925441266,0.3163098423
O,0,2.4904404607,-0.8794357098,-0.1891016754
C,0,-0.8124489326,0.6914266278,-0.3365944804
C,0,0.7121450192,0.7470996935,-0.4203993467
C,0,-1.410060724,1.7401767254,0.5966338215
C,0,1.4682257428,1.9398873247,-0.8136313598
C,0,-1.0071456386,1.8054848365,1.9380639705
C,0,-1.5553795387,2.7534374737,2.7993599234
C,0,-2.5176407724,3.6548943735,2.3346757877
C,0,-2.9238484805,3.5999484476,1.0023724823
C,0,-2.3710474682,2.6484153578,0.1404476007
C,0,0.8794574916,2.9255990793,-1.6296205745
C,0,1.5868442123,4.0633810188,-2.011761057
C,0,2.9023949652,4.2517847808,-1.5860725046
C,0,3.4994765644,3.2902306653,-0.7676995007
C,0,2.7941158781,2.1525313264,-0.3846580732
C,0,-2.4645143443,-3.2611460636,1.15502729
C,0,-3.6760073757,-1.8905415917,-1.3857625336
C,0,-3.9256081477,-0.576621374,1.4546309938
C,0,4.4351993184,-2.1825857862,0.3117334021
C,0,-2.7739205426,-2.7373356285,-2.3073622395
C,0,-3.9896593087,-0.5524845513,-2.0874608049
C,0,-5.0043376639,-2.6456318934,-1.1568425406
C,0,3.0220834345,-1.7588117373,0.723141101
O,0,2.4265461688,-2.1296533882,1.706275648
C,0,4.9864637548,-3.1431060233,1.3772262956
C,0,5.3370852384,-0.9327303481,0.2035683555
C,0,4.3624546592,-2.8911283868,-1.0603842377
H,0,0.2021616026,-2.4397866006,0.4683186298
H,0,-1.2293423573,0.8601114151,-1.3409173628
H,0,-0.2571431614,1.1081383743,2.3023912142
H,0,-1.2305666371,2.790526461,3.8360192122
H,0,-2.9438437345,4.3947570502,3.0069220057
H,0,-3.668573862,4.2978459709,0.6283506344
H,0,-2.6943366275,2.6117173095,-0.897773287
H,0,-0.140416005,2.7962362542,-1.9771470722
H,0,1.10715412,4.8045296709,-2.6458330303
H,0,3.4543255461,5.1391903874,-1.8838276991
H,0,4.5189960421,3.4306761359,-0.4174658294
H,0,3.2644167455,1.4270179979,0.2681381977
H,0,-1.9207322696,-3.1174602306,2.0959281865
H,0,-1.8704272243,-3.9366924938,0.5292879006
H,0,-3.4030817041,-3.7743671433,1.3944019881
H,0,-4.868678647,-1.1059316859,1.6376730692
H,0,-4.1644328076,0.4103027007,1.0463113537
H,0,-3.4416325308,-0.4151795382,2.4244519805
H,0,-2.5615940452,-3.723595336,-1.8771131683
H,0,-1.8131585924,-2.2463353249,-2.5024138397
H,0,-3.2642037367,-2.9041272558,-3.2778855781
H,0,-3.0776310166,0.0057899664,-2.3305716127
H,0,-4.6274400203,0.0964147671,-1.475292104
H,0,-4.5195454375,-0.7316983379,-3.034772706
H,0,-5.6989348651,-2.0773266238,-0.5264915142
H,0,-4.8483044055,-3.6236369956,-0.6860455793
H,0,-5.5102733652,-2.8248794016,-2.1171398453
H,0,4.3562410475,-4.0320941111,1.4749704977
H,0,5.0338146969,-2.6624785801,2.3591194936
H,0,5.9967942463,-3.4628666525,1.098779568
H,0,5.3932648142,-0.3999974368,1.1602939165
H,0,4.9718166793,-0.2388088926,-0.5583272677
H,0,6.3541487981,-1.2374255754,-0.0684653267
H,0,3.9793115125,-2.2215612331,-1.8354184382
H,0,3.7163482092,-3.7756557388,-1.0174970767
H,0,5.3647921615,-3.2223818295,-1.3551676315

G(au)= -1528.137862
HF(au)= -1528.6402879

VIk

C,0,-4.7844718984,2.8275426457,1.4018015467
C,0,-4.3752087527,4.0786796117,1.7007636774
C,0,-3.5732635566,1.9721002578,1.247886233
Si,0,-3.4570540958,1.3962716833,-0.6289842018
O,0,-5.2218918451,5.1543710358,1.9509281755
C,0,-2.4352509086,2.8755964305,1.6542020645
C,0,-2.9262232531,4.1407962135,1.8729779869
C,0,-1.0434231406,2.4121923858,1.8139404813
C,0,-2.1976644834,5.3515115866,2.3295542955
C,0,-0.7707506437,1.1505238332,2.3752610695
C,0,0.538857802,0.697667971,2.5310776534
C,0,1.6110929738,1.4955011794,2.1287366853
C,0,1.3586168986,2.750316942,1.5696194122
C,0,0.0503889275,3.2031929517,1.4133584325
C,0,-1.5504940092,5.3597171247,3.5759506112
C,0,-0.873787958,6.4945414312,4.0211526358
C,0,-0.8319890086,7.6436836395,3.2289295667
C,0,-1.4717948635,7.6484142986,1.9883155385
C,0,-2.1513030599,6.5133212728,1.5414913837
C,0,-4.1082893367,2.7783262234,-1.7449078761
C,0,-4.5259080193,-0.2065994855,-0.8307486833
C,0,-1.6714892094,1.0065358996,-1.1233937299
C,0,-6.4263508574,7.1457799937,1.4044816976
C,0,-5.9608253979,-0.0066412276,-0.3013979692
C,0,-3.8789716778,-1.3873415493,-0.0766963627
C,0,-4.6014749238,-0.5596415848,-2.3334728372
C,0,-5.4712549766,6.0424562082,0.9405060936
O,0,-4.9898496369,5.939924299,-0.1646833095
C,0,-6.6285435118,8.1387349528,0.2492742514
C,0,-5.8153427846,7.8650030691,2.6281082217
C,0,-7.7758819108,6.5023196958,1.7970946917
H,0,-5.8066292225,2.5133399456,1.2395769847
H,0,-3.6142033982,1.0433698926,1.8332610534
H,0,-1.5929272973,0.5246347945,2.7111569353
H,0,0.7207614434,-0.2786668873,2.9731286134
H,0,2.6321357169,1.1434941947,2.2488022986
H,0,2.1846128408,3.3786321559,1.2464090465
H,0,-0.1330043724,4.1737458841,0.9646476233
H,0,-1.5827969929,4.4681247137,4.1953746252
H,0,-0.3798708201,6.4814809421,4.9893568398
H,0,-0.3038574338,8.528074569,3.5756712273
H,0,-1.4390755408,8.5360063742,1.3613896466
H,0,-2.6435171688,6.5193790717,0.5739107929
H,0,-3.8222384862,3.7667117401,-1.3704432279
H,0,-5.2019859832,2.7742751308,-1.8114407369
H,0,-3.7122437029,2.668002243,-2.7616034154
H,0,-1.6505834449,0.5942623749,-2.1400038359
H,0,-1.1835089424,0.288335079,-0.4576862134
H,0,-1.0601012088,1.9147252222,-1.1236887641
H,0,-6.4708799109,0.8313288691,-0.7924055597
H,0,-5.9788304132,0.1742965142,0.7798339995
H,0,-6.5647843982,-0.9070708247,-0.4868745265
H,0,-3.8053600093,-1.1990292371,1.0021532404
H,0,-2.8706450376,-1.6085760612,-0.4453178228
H,0,-4.480658869,-2.2993976697,-0.202446304
H,0,-3.6101467254,-0.7412441322,-2.7661042109
H,0,-5.0809696868,0.2346688018,-2.9175007564
H,0,-5.1918154888,-1.4759172535,-2.4811192328
H,0,-7.0441067745,7.6430961801,-0.6331407234
H,0,-5.6814895749,8.60030799,-0.0467131603
H,0,-7.3178845615,8.9319878148,0.5596770508
H,0,-4.8383104805,8.2986237213,2.3881594718
H,0,-5.6833796169,7.1786104757,3.4686057867
H,0,-6.479668304,8.6775987888,2.9445102271
H,0,-7.653035615,5.7889216631,2.6168500915
H,0,-8.2279523097,5.9775564178,0.9472820939
H,0,-8.474932091,7.2822415116,2.1202804054

G(au)= -1528.137827
HF(au)= -1528.6431352|

VIIk

C,0,0.3672515211,-0.9837799491,-2.2931686263
C,0,1.0383840365,0.2244516971,-1.728510676

VIIIk

C,0,0.1823719427,-0.8951407071,1.3084711503
C,0,0.9628245221,0.304899639,0.8670411126

```
C,0,-0.9939741209,-0.4511295711,-2.5956103691
Si,0,0.2344499409,-2.4502196205,-1.0070747648
O,0,2.3674418577,0.3296955292,-1.3453891045
C,0,-1.1211813872,0.8325082644,-2.1591137849
C,0,0.1758456091,1.2752216283,-1.6054104098
C,0,-2.3748055357,1.6154389206,-2.2044587848
C,0,0.5194793236,2.6263440103,-1.1116946858
C,0,-3.20369499,1.5535432687,-3.3382934026
C,0,-4.416844489,2.2393572804,-3.378979549
C,0,-4.8248620131,3.0094013818,-2.2881938979
C,0,-4.0086164281,3.0859768181,-1.1578285719
C,0,-2.7961509139,2.3991028443,-1.1158539582
C,0,0.1692150124,3.7721645663,-1.8471226647
C,0,0.5166571538,5.0447035932,-1.3960637134
C,0,1.2186289421,5.2014474918,-0.1992410955
C,0,1.5694830526,4.0729880766,0.5440614241
C,0,1.2220875846,2.7993560672,0.0935659363
C,0,1.8888070221,-2.6640731465,-0.1073636169
C,0,-0.1877800771,-4.0814775884,-1.9478080742
C,0,-1.0947426781,-2.0273068563,0.2728474447
C,0,4.7478359759,0.222204294,-1.5437288679
C,0,1.0105495422,-4.5262539969,-2.8142248454
C,0,-1.4223940185,-3.9160886358,-2.8594110539
C,0,-0.4918087096,-5.1873407661,-0.9107375128
C,0,3.3828650304,-0.2304672843,-2.0743937379
O,0,3.2066088623,-0.9601914988,-3.023157894
C,0,5.8409696724,-0.6071211448,-2.2369340011
C,0,4.8276331345,0.0371989515,-0.0140013305
C,0,4.9142666295,1.7206525065,-1.8956358871
H,0,0.9048920196,-1.39186411,-3.1557351189
H,0,-1.7951161867,-1.0458454849,-3.0161322411
H,0,-2.879532249,0.9717972245,-4.1969641922
H,0,-5.0398453293,2.1788619749,-4.2677887305
H,0,-5.7680437658,3.5483934201,-2.3202569901
H,0,-4.3176241588,3.6812465707,-0.3023794461
H,0,-2.173974572,2.4596459759,-0.2284335919
H,0,-0.3725693184,3.6600815911,-2.781219651
H,0,0.2390937126,5.9162657178,-1.9832520765
H,0,1.4873660552,6.1941506261,0.1521266384
H,0,2.1094417773,4.1833823735,1.4811413344
H,0,1.4871250571,1.926591393,0.6826779738
H,0,2.1210711124,-1.7826997531,0.4996733454
H,0,2.7171547138,-2.8235204646,-0.8050534506
H,0,1.8488555416,-3.5274248726,0.567705846
H,0,-1.0235280953,-2.6921291245,1.1417315526
H,0,-2.1083495396,-2.1091491324,-0.1328812439
H,0,-0.967872519,-0.9984855877,0.6291485611
H,0,1.8980000882,-4.7421399237,-2.2084854676
H,0,1.2935748589,-3.7687621383,-3.5553850362
H,0,0.7604865588,-5.444262598,-3.365673295
H,0,-1.2361937162,-3.2126953309,-3.6792231555
H,0,-2.3044678198,-3.5671974641,-2.3083180616
H,0,-1.6865082861,-4.8807692853,-3.3168522657
H,0,-1.3639600968,-4.9424312269,-0.2928292974
H,0,0.3546589937,-5.3716960831,-0.2377883238
H,0,-0.7101397388,-6.1361245872,-1.4222636986
H,0,5.7948323045,-0.4953065945,-3.3236478893
H,0,5.7362325239,-1.6730000164,-2.0088381122
H,0,6.8278552487,-0.2776179504,-1.8934702671
H,0,4.6813605374,-1.0107898741,0.2706499029
H,0,4.0777409595,0.6418962754,0.5026789515
H,0,5.8184078472,0.3458975224,0.3389488351
H,0,4.1427074408,2.330788148,-1.4172677189
H,0,4.8596599733,1.8804361799,-2.9785072896
H,0,5.8949955246,2.0700784775,-1.5525952544
```

G(au)= -1528.140004
HF(au)= -1528.6443645

```
C,0,-0.5378872395,-0.4112032837,2.5576799899
Si,0,-1.5113336723,-1.6304669678,3.6249686317
O,0,1.6909289836,0.4329138669,-0.296628996
C,0,-0.1764494953,0.8930377367,2.76079462
C,0,0.7654941751,1.3534072938,1.7056297151
C,0,-0.5791820118,1.776734938,3.8866322487
C,0,1.3449337293,2.7069182955,1.5646327593
C,0,-1.9159025944,2.1617624366,4.0692531803
C,0,-2.2828383965,2.9957462825,5.1274160111
C,0,-1.3172910678,3.4618840773,6.0202310544
C,0,0.0188199883,3.0951299793,5.8430805155
C,0,0.3850138017,2.2678592228,4.7826758501
C,0,0.5406407141,3.8559341316,1.6522102147
C,0,1.0911556717,5.1270243311,1.4943728704
C,0,2.4574095591,5.2785719171,1.2508561728
C,0,3.2701604219,4.1464534469,1.1676323499
C,0,2.7197971649,2.8748097336,1.3243818235
C,0,-0.5095612548,-3.2471645109,3.6444434488
C,0,-3.2681294658,-1.9938512911,2.8890319861
C,0,-1.6746574727,-1.0570540121,5.423536962
C,0,2.8103782615,-0.1652019322,-2.3163478744
C,0,-3.161733588,-2.6251243824,1.4844029402
C,0,-4.0978565655,-0.6988025749,2.7796618869
C,0,-4.0113310786,-2.9814716309,3.8160957097
C,0,2.104927239,-0.639799836,-1.0418279346
O,0,1.9219363284,-1.7908739493,-0.7203213829
C,0,3.2789193526,-1.3994348957,-3.1030676997
C,0,4.021564417,0.7153196438,-1.9348001471
C,0,1.812215528,0.659407229,-3.1611184776
H,0,-0.5007705444,-1.2408390454,0.5207659585
H,0,0.8350665077,-1.7510043023,1.5137279482
H,0,-2.668846095,1.8094898997,3.3711125029
H,0,-3.3239626319,3.2834530852,5.2500267687
H,0,-1.6015777852,4.1106513286,6.844454913
H,0,0.7787075223,3.4563854648,6.5311515919
H,0,1.4270077599,1.9917278575,4.646878758
H,0,-0.5233173622,3.750494264,1.8391532054
H,0,0.4493205924,6.0016202654,1.5618298015
H,0,2.8856549519,6.2701959207,1.1307828878
H,0,4.3369343327,4.2526482109,0.9874004956
H,0,3.3591273999,1.9987672828,1.2727490526
H,0,0.4787783246,-3.0787232703,4.0893425023
H,0,-0.3526327428,-3.6651872776,2.6437998785
H,0,-1.0115940381,-4.0136875341,4.2468022785
H,0,-2.0254303805,-1.889283175,6.0460122494
H,0,-2.3693020677,-0.2220821862,5.550236897
H,0,-0.7046533069,-0.7338936616,5.8173894154
H,0,-2.596633863,-3.5648189696,1.4912008357
H,0,-2.6806150231,-1.9495151637,0.7670445934
H,0,-4.1642450433,-2.8511776562,1.0918819273
H,0,-3.6274400794,0.0250077165,2.1032274936
H,0,-4.2335026824,-0.2122327146,3.7532004875
H,0,-5.1000445778,-0.9166680441,2.3814705374
H,0,-4.164767039,-2.5681181009,4.8199406705
H,0,-3.4736353999,-3.9313794048,3.9235428752
H,0,-5.0044926644,-3.2160355487,3.4051809522
H,0,2.4357896983,-2.0416946748,-3.3744648277
H,0,3.9783792684,-2.0026855126,-2.516305308
H,0,3.7828094775,-1.0812275902,-4.0225753986
H,0,4.7382418891,0.1584566062,-1.3196159473
H,0,3.7109158601,1.6067434763,-1.3831079756
H,0,4.5412683013,1.0365104084,-2.8448437785
H,0,1.4702019565,1.5462663217,-2.620650306
H,0,0.9352484503,0.0613858276,-3.4351482702
H,0,2.2982321361,0.985446669,-4.0878573579
```

G(au)= -1528.138473
HF(au)= -1528.642378

IXk

```
C,0,-0.026667772,-1.6917167038,-0.0466062264
C,0,1.1412574896,-0.8399583575,-0.4451637018
C,0,-1.10792078,-0.9263771254,0.2339975381
```

Xk

```
C,0,0.7385553736,-1.9258973699,-0.0986049353
C,0,0.0669260199,-0.6293721672,-0.3352804574
C,0,-0.2275806348,-2.8657972791,-0.2399353479
```

Si,0,-2.7996011998,-1.7489556761,0.5267495437
O,0,2.3392621547,-1.1488193667,0.2998964663
C,0,-0.6683919575,0.5104223056,0.1362997749
C,0,0.6500690127,0.575841914,-0.2081224161
C,0,-1.5594734951,1.6797024623,0.3526159844
C,0,1.5396345856,1.7372172461,-0.3647892424
C,0,-2.1235710488,1.9364564851,1.6122029854
C,0,-2.9414884055,3.0489570801,1.8161672558
C,0,-3.2140414375,3.9224718134,0.7617304869
C,0,-2.6565789218,3.6803993493,-0.4952604714
C,0,-1.8345245725,2.5718452664,-0.6968336455
C,0,1.4114421637,2.8838672735,0.443602384
C,0,2.2745856624,3.9662884607,0.293930564
C,0,3.2923477786,3.9338585214,-0.6622601398
C,0,3.4415511317,2.8008138365,-1.4631669689
C,0,2.5799187029,1.7145452912,-1.3152360767
C,0,-2.4331168481,-3.5669290256,0.9412512969
C,0,-3.9038471862,-1.6681447014,-1.0601928477
C,0,-3.7403119073,-1.0342897499,2.0104739877
C,0,4.6154975552,-1.8271361841,0.5348763293
C,0,-3.1172586301,-2.1688955249,-2.2894045871
C,0,-4.3925166851,-0.2303657842,-1.3299409318
C,0,-5.137486702,-2.576390516,-0.8564605188
C,0,3.4302068032,-1.5555780262,-0.3982621256
O,0,3.444629494,-1.6802429103,-1.6056880051
C,0,5.8170718487,-2.2787463479,-0.3096609975
C,0,4.2218036311,-2.9353473652,1.5371774135
C,0,4.9560910452,-0.5271180676,1.2980389394
H,0,0.020123278,-2.7740147191,-0.074518697
H,0,1.3633792239,-1.0036187101,-1.5094500026
H,0,-1.9030110775,1.2684366386,2.4396531945
H,0,-3.3618728113,3.2347846985,2.8011940856
H,0,-3.8529166569,4.7872540198,0.9194486487
H,0,-2.860012394,4.3566968895,-1.3213290183
H,0,-1.3949115628,2.3904401216,-1.6734764305
H,0,0.6381386922,2.9176710423,1.2028566085
H,0,2.1557662633,4.836976658,0.9337727846
H,0,3.9646469318,4.7799357745,-0.7769691912
H,0,4.2303360856,2.7595401211,-2.2099358108
H,0,2.7146840656,0.8433526527,-1.9491355502
H,0,-1.7291768521,-3.64286774,1.7782036164
H,0,-2.0076511139,-4.1225011065,0.0979411173
H,0,-3.3539453444,-4.0810085997,1.2400049805
H,0,-4.6331507299,-1.6408732539,2.2058668761
H,0,-4.0623170047,-0.000710202,1.8575485734
H,0,-3.1234218155,-1.0638033742,2.9167980054
H,0,-2.7704269445,-3.2021198465,-2.1642718054
H,0,-2.2386786352,-1.5466617209,-2.4939586147
H,0,-3.7552337066,-2.1463808384,-3.1850290369
H,0,-3.5636306626,0.4626033469,-1.5080518883
H,0,-4.9809496167,0.1677584469,-0.4949290354
H,0,-5.0359223188,-0.2098935488,-2.2220997054
H,0,-5.7444111206,-2.2640046225,0.0024571459
H,0,-4.858360169,-3.6258717116,-0.7066618931
H,0,-5.7877992687,-2.5361203153,-1.7426726021
H,0,6.1037498587,-1.5104643981,-1.0340172656
H,0,5.5896550785,-3.1914627739,-0.8687895254
H,0,6.6744726562,-2.4764059158,0.3436028127
H,0,3.969252694,-3.8679213055,1.0187881183
H,0,3.3632701839,-2.6363115688,2.1448121669
H,0,5.0643792615,-3.1426354859,2.2070471285
H,0,4.1163801421,-0.1966324795,1.9150332414
H,0,5.2084155512,0.2842389204,0.6061913276
H,0,5.8209296933,-0.6992851398,1.9492305883

G(au)= -1528.132205
HF(au)= -1528.6373476

Si,0,2.5506681578,-2.3391712028,0.2675579998
O,0,0.7722924356,0.5744627858,-0.3728596978
C,0,-1.5645181403,-2.2490056138,-0.5833401056
C,0,-1.2475807153,-0.7621408258,-0.6330260097
C,0,-2.6612402048,-2.6285998726,0.407067321
C,0,-2.2473908849,0.2661241919,-0.9638094674
C,0,-2.6024445072,-2.1766535027,1.7333968459
C,0,-3.5856209079,-2.5459351849,2.648884406
C,0,-4.641631226,-3.3731716748,2.2550036334
C,0,-4.7072763428,-3.8256752988,0.9380866616
C,0,-3.7214760788,-3.4534539433,0.0202799076
C,0,-3.1060517903,0.0917510713,-2.0642962938
C,0,-4.0612201278,1.0533551899,-2.3880380768
C,0,-4.1850784412,2.2091759097,-1.6145572945
C,0,-3.350702385,2.3885900168,-0.5099691017
C,0,-2.3948804676,1.4265756134,-0.1841240113
C,0,3.3575388957,-1.0130294179,1.3533257286
C,0,3.5246721683,-2.534832039,-1.3891426449
C,0,2.5601868995,-3.9728282742,1.2311432352
C,0,1.6240615325,2.6306983238,0.5679437173
C,0,3.43733067,-1.2396449536,-2.2228289259
C,0,2.9406364546,-3.6990393458,-2.2167617995
C,0,5.0080399145,-2.8344694599,-1.0823433529
C,0,0.9020761041,1.2932849856,0.7837852618
O,0,0.443448789,0.9233368369,1.841384684
C,0,2.4914305122,2.9125948984,1.8105265018
C,0,0.5188427919,3.7111370634,0.4583498451
C,0,2.4868559911,2.6457490904,-0.7052048967
H,0,-0.1112136273,-3.9362270714,-0.111270804
H,0,-1.8706683706,-2.6006909446,-1.5812900699
H,0,-1.7857468546,-1.5272963991,2.0393408743
H,0,-3.5290359452,-2.1856907382,3.6728469578
H,0,-5.4083601051,-3.658809927,2.9703186463
H,0,-5.5261653046,-4.46576342,0.6197819729
H,0,-3.7797412846,-3.8079148896,-1.0066916096
H,0,-3.0113576945,-0.7977580137,-2.6812061956
H,0,-4.7089537594,0.9001943356,-3.2473894829
H,0,-4.9309710041,2.9585329556,-1.865682879
H,0,-3.452678364,3.2747700873,0.111288985
H,0,-1.7827718239,1.5497347912,0.7027350913
H,0,2.6963971272,-0.727329193,2.1783329559
H,0,3.5963413186,-0.1052665076,0.788731413
H,0,4.2931327279,-1.3878593113,1.784913875
H,0,3.5859720358,-4.2866352098,1.4588800309
H,0,2.0808774411,-4.791959001,0.6838830144
H,0,2.0308703862,-3.8574980736,2.1840468487
H,0,3.8800363131,-0.3834141121,-1.6999926809
H,0,2.4020818838,-0.9781266555,-2.4701598265
H,0,3.9830461527,-1.3576353614,-3.1707611456
H,0,1.882173107,-3.5396831619,-2.4541923504
H,0,3.0256920069,-4.658999415,-1.6929715845
H,0,3.4816089673,-3.799169133,-3.169146103
H,0,5.573784949,-2.962399599,-2.0168953361
H,0,5.1298584775,-3.7571675678,-0.5019329563
H,0,5.4853585776,-2.0211195166,-0.5225074335
H,0,3.3028097635,2.1823883139,1.9040297183
H,0,1.8914986431,2.8691259078,2.723021303
H,0,2.9399314685,3.9091987996,1.7307481133
H,0,0.983040294,4.6955858447,0.3292132838
H,0,-0.0938818605,3.737162568,1.3654526547
H,0,-0.1378038254,3.5269304907,-0.3982946664
H,0,2.9787855658,3.6208765522,-0.7984276855
H,0,1.8867262515,2.4765575271,-1.6028432542
H,0,3.2674505101,1.878413497,-0.6723930045

G(au)= -1528.132785
HF(au)= -1528.6377794

XIk

C,0,-0.8894795285,-1.7964951255,0.2104782103
C,0,-0.2264060529,-0.6320744385,0.3990568178
C,0,0.1065476093,-2.8561768311,0.4745324892
Si,0,-2.6945780784,-2.1735953034,-0.2170897793
O,0,-0.7204785314,0.6624044469,0.3576461171

XIIk

C,0,1.1389703505,-1.257176174,1.5884916544
C,0,0.8862560399,-0.3947684717,0.3598149139
C,0,-0.0761226716,-1.7351271442,1.9496515498
Si,0,2.7766535723,-1.7242382657,2.3914008394
O,0,1.5973396923,0.8570392896,0.2468408496

C,0,1.3206271255,-2.358868077,0.8243813035
C,0,1.2138114894,-0.8382648918,0.8351849025
C,0,2.5283026851,-3.1126485365,1.1771698562
C,0,2.2431601497,-0.0809787887,0.007056805
C,0,2.682155045,-4.4576505561,0.7848835429
C,0,3.811513629,-5.1874561645,1.1423633146
C,0,4.8286417271,-4.5922764491,1.8936558595
C,0,4.7002848757,-3.2574368444,2.2787362825
C,0,3.566829078,-2.5271805108,1.9251756516
C,0,2.9900467552,0.9579182346,0.5734994145
C,0,3.9300777005,1.6605795904,-0.1853133539
C,0,4.1314803308,1.3298295749,-1.5248063478
C,0,3.3870303288,0.2959430681,-2.1000228578
C,0,2.450508648,-0.4025107426,-1.341406015
C,0,-3.5536848372,-0.7172609412,-1.0653304562
C,0,-3.6296096262,-2.6451585771,1.4067944646
C,0,-2.6813600068,-3.6359706108,-1.4258360353
C,0,-1.1263951751,2.8052449074,-0.647229211
C,0,-3.6139577126,-1.4543635319,2.388063786
C,0,-2.9627689209,-3.8594248724,2.0869900867
C,0,-5.0947851792,-3.0032414614,1.0749974546
C,0,-0.7896103094,1.3247747939,-0.8521391485
O,0,-0.5964751947,0.7795751494,-1.9101384568
C,0,-1.2664678915,3.4673216213,-2.0274981685
C,0,0.0262810411,3.4663243102,0.1436270734
C,0,-2.4433241561,2.9432641143,0.1471722944
H,0,-0.1349822992,-3.9127916108,0.4230956544
H,0,1.2882545896,-0.4816993176,1.8749970192
H,0,1.9144494841,-4.9267596336,0.1761146615
H,0,3.9039287444,-6.222768901,0.8240961612
H,0,5.7128293439,-5.1609849259,2.1683416597
H,0,5.4853237887,-2.7795189113,2.8591457143
H,0,3.4861094696,-1.4908721334,2.2373670278
H,0,2.8378428204,1.2201064472,1.6185786385
H,0,4.503969869,2.4615483873,0.2736189755
H,0,4.8630424816,1.8720396114,-2.1180070764
H,0,3.5347186352,0.0348384235,-3.1445126991
H,0,1.8674125427,-1.1982621537,-1.7952199708
H,0,-2.9713000195,-0.3718717381,-1.9252714193
H,0,-3.693788558,0.1332673256,-0.3901612587
H,0,-4.5430239606,-1.0208115753,-1.427892856
H,0,-3.7012225514,-3.9222074061,-1.7095758234
H,0,-2.1931200189,-4.5263483271,-1.0138821809
H,0,-2.1484885424,-3.3624167191,-2.3442299479
H,0,-4.1167534243,-0.5731547869,1.9716872808
H,0,-2.5921711002,-1.1601735535,2.6557065944
H,0,-4.1367959658,-1.7178352867,3.3193408682
H,0,-1.9254011462,-3.6505334003,2.3732484424
H,0,-2.9612370145,-4.7451149351,1.4396193667
H,0,-3.5066857501,-4.1301354695,3.0040422981
H,0,-5.6416989764,-3.2630536974,1.9933069559
H,0,-5.1650454725,-3.8663050937,0.4015345056
H,0,-5.628084634,-2.1677541137,0.6055848345
H,0,-2.0687607293,3.0046058734,-2.6106157312
H,0,-0.342481809,3.3763956995,-2.6057967779
H,0,-1.4974324722,4.5315950427,-1.9056948141
H,0,-0.1799185633,4.5364983204,0.2616672709
H,0,0.9825188588,3.358982222,-0.3794512149
H,0,0.1297335426,3.0240108132,1.1384817937
H,0,-2.6690487418,4.0053363387,0.2975646852
H,0,-2.3711635225,2.4649984274,1.1279157652
H,0,-3.2840129158,2.4960962008,-0.3948212995

G(au)= -1528.136564
HF(au)= -1528.6395044

C,0,-1.1678050996,-1.1757181619,1.1351159479
C,0,-0.629246317,-0.3088993677,0.2288282057
C,0,-2.5753406389,-1.5876866309,1.3220934047
C,0,-1.2757332213,0.5728023566,-0.7513726539
C,0,-3.0975154439,-1.7319505326,2.6191458037
C,0,-4.4143487926,-2.145642532,2.8184122005
C,0,-5.2317638559,-2.4354158634,1.7248220228
C,0,-4.7218667541,-2.3069686579,0.4305148277
C,0,-3.4079226888,-1.8887397363,0.2304373466
C,0,-0.7022373548,0.789544284,-2.0194169809
C,0,-1.3023085006,1.6449999234,-2.9424808897
C,0,-2.4843978813,2.3126026902,-2.6179322293
C,0,-3.0581677138,2.1195079182,-1.3584765018
C,0,-2.4614669766,1.2644567806,-0.4354446741
C,0,3.6538976768,-0.1703150205,3.032592119
C,0,3.9232146029,-2.6689091949,1.1530639062
C,0,2.3684336878,-2.8341831541,3.8745012072
C,0,2.0110872441,3.1533438091,0.8213379967
C,0,4.4175705373,-1.7298440811,0.0326308043
C,0,3.1636760064,-3.8534816818,0.5195550523
C,0,5.1527459958,-3.21611786,1.9114838544
C,0,1.3402893461,1.8177197006,1.1756072833
O,0,0.6245090406,1.6348224909,2.134110453
C,0,2.1054500092,4.0044766159,2.0989556437
C,0,1.0916475312,3.8546004836,-0.2087728639
C,0,3.4104227675,2.949743908,0.2097893166
H,0,1.2675883122,-0.9513310832,-0.5092836941
H,0,-0.2585341988,-2.4659298541,2.7316085655
H,0,-2.4699904361,-1.4982056773,3.4751398571
H,0,-4.8013133253,-2.2414143079,3.8295946424
H,0,-6.2565321254,-2.7625660376,1.8791488991
H,0,-5.3479035419,-2.5395829109,-0.4270235892
H,0,-3.0144410369,-1.800654101,-0.7774499208
H,0,0.2153702916,0.2744870913,-2.2890881425
H,0,-0.8452245072,1.7878310813,-3.9183203459
H,0,-2.9514167309,2.9807292464,-3.3364836578
H,0,-3.9711402418,2.6444790225,-1.0895949814
H,0,-2.9015486874,1.1351972612,0.5476556497
H,0,2.9741171336,0.4141454554,3.6624056897
H,0,3.9863013937,0.4772735232,2.2143036426
H,0,4.534319622,-0.429179755,3.6328843766
H,0,3.2755789428,-3.0839428794,4.4370198586
H,0,1.8948596872,-3.7767071653,3.5763389131
H,0,1.6869480702,-2.322324912,4.5639353364
H,0,5.0225836214,-0.904865209,0.4265098836
H,0,3.5928377103,-1.2875769779,-0.5389971831
H,0,5.0463640802,-2.2838402158,-0.6800603221
H,0,2.2899812616,-3.5229052628,-0.0545665751
H,0,2.809713807,-4.5655028986,1.2752061541
H,0,3.8202128294,-4.4073357736,-0.1676657126
H,0,5.8248216927,-3.7448651553,1.2195702505
H,0,4.8683071123,-3.9279843629,2.6952849571
H,0,5.7374439322,-2.4156195882,2.3814851632
H,0,2.7530821036,3.5301247387,2.8444866222
H,0,1.1210456647,4.1431695976,2.5530941348
H,0,2.5242495768,4.988624991,1.8597704745
H,0,1.5083858266,4.8364817383,-0.4625371962
H,0,0.0860315294,4.0047550978,0.1982507204
H,0,1.0018085047,3.26892596,-1.1285849354
H,0,3.8440675892,3.9244910704,-0.0428192381
H,0,3.3684542332,2.3460497996,-0.7001835671
H,0,4.088520113,2.4574657331,0.9161448605

G(au)= -1528.135779
HF(au)= -1528.6412636

XIIIk

C,0,-0.3332214682,-1.1798146486,-2.047887149
C,0,0.3245978214,-0.2414043576,-1.1609745661
C,0,-1.6668124777,-1.0331701218,-1.8752496677
Si,0,-2.517279199,-1.2145935546,0.7169829004
O,0,1.6941999819,-0.1242118526,-0.9743961709
C,0,-1.9654442132,-0.0251104947,-0.8033759245
C,0,-0.572898917,0.4920080937,-0.4405825529
C,0,-2.9969633191,1.031081046,-1.2108694721

TS1

C,0,0.2021561006,-1.539058729,0.3771773386
C,0,1.1827945271,-0.5828960552,0.1151266955
C,0,-1.08661888,-0.9765064126,0.2213890469
Si,0,-2.7373233731,-1.8779354322,0.4580093063
O,0,2.5655180838,-0.6837387702,0.0601103883
C,0,-0.9111824004,0.3871638313,-0.165281099
C,0,0.5791261396,0.650935764,-0.2375893094
C,0,-1.9204072085,1.4836689878,-0.2109534075

```
C,0,-0.2108110267,1.555238392,0.5259412437
C,0,-3.245328059,1.2695080552,-2.5746521769
C,0,-4.163159638,2.2353336865,-2.9893879482
C,0,-4.8563830892,2.9987870047,-2.0503575456
C,0,-4.6093804414,2.7901019426,-0.6935297619
C,0,-3.6925566639,1.821788863,-0.2813062843
C,0,-0.67185884,2.8756720323,0.3788845885
C,0,-0.2903393359,3.8717406694,1.2782722997
C,0,0.5649295953,3.5757555115,2.3406552696
C,0,1.0434232746,2.2730912245,2.492988884
C,0,0.660876225,1.2762434519,1.5954686873
C,0,-1.0707041556,-2.3624679533,1.1480835069
C,0,-4.0853368476,-2.2987580314,0.3281752625
C,0,-2.8567293575,-0.2027839627,2.2933277336
C,0,4.0027376016,-0.023805102,-1.5803847959
C,0,-3.7295638312,-3.5412674998,-0.5176133155
C,0,-5.1951657411,-1.5038459076,-0.3898821884
C,0,-4.6484228421,-2.8042308791,1.6795593491
C,0,2.5505604661,-0.1731174524,-2.0447242334
O,0,2.1812850724,-0.3129098552,-3.1863229607
C,0,4.9207278984,-0.1124472931,-2.8096022895
C,0,4.3369141728,-1.1568156756,-0.5841307017
C,0,4.1698876306,1.3478025097,-0.8872232033
H,0,0.1732588028,-1.8555415647,-2.7224937119
H,0,-2.4400110867,-1.5603677378,-2.4196738721
H,0,-2.7047958016,0.6975127404,-3.3218386095
H,0,-4.3311517935,2.3908407253,-4.0520880922
H,0,-5.5742303165,3.7489780429,-2.3709493713
H,0,-5.1306415289,3.3828023229,0.0540129126
H,0,-3.5067350769,1.7000465764,0.7777119217
H,0,-1.3172152192,3.1254111899,-0.4552331684
H,0,-0.6567468191,4.8858158486,1.140244695
H,0,0.8608619127,4.3535834035,3.0396185976
H,0,1.714167288,2.0300564722,3.3132483956
H,0,1.0396408808,0.2661028888,1.7189361623
H,0,-0.2686498908,-1.8138423087,1.651811588
H,0,-0.6449051333,-2.8621879564,0.2742331058
H,0,-1.4195908525,-3.1378667349,1.8417577308
H,0,-2.7708974245,-0.8701491501,3.1596754269
H,0,-3.8573501175,0.2395509634,2.3236736854
H,0,-2.121874592,0.5975682943,2.4239248926
H,0,-2.9728973115,-4.1674306644,-0.0310278761
H,0,-3.3530930509,-3.2816205964,-1.5117685791
H,0,-4.6252563857,-4.1630425177,-0.6617702949
H,0,-4.8826102053,-1.1580400609,-1.3805924364
H,0,-5.5068914382,-0.6194801167,0.1777504133
H,0,-6.0841544179,-2.1375384549,-0.5253799148
H,0,-5.009687109,-1.9865282695,2.3121719113
H,0,-3.9064907724,-3.3715931588,2.255671515
H,0,-5.4991482721,-3.477243429,1.4989610787
H,0,4.6893723677,0.6738071119,-3.5342578126
H,0,4.8109377129,-1.074408856,-3.3197329422
H,0,5.9658178139,-0.0021987886,-2.4991377228
H,0,4.2083213233,-2.1430682789,-1.045308264
H,0,3.702469684,-1.1080874599,0.3051268648
H,0,5.3823394395,-1.0692700993,-0.2664612691
H,0,3.5171858479,1.4388146514,-0.0148102357
H,0,3.937998737,2.169092438,-1.5749886663
H,0,5.2089045288,1.4676706929,-0.5588208746
```

G(au)= -1528.116561
HF(au)= -1528.6223616

```
C,0,1.2299764278,1.9756612598,-0.3670697708
C,0,-2.7900860953,1.6402314475,-1.3000408973
C,0,-3.7444410144,2.6595067784,-1.3124580035
C,0,-3.8387706546,3.5414403469,-0.2354904929
C,0,-2.970637156,3.4028440158,0.8502905859
C,0,-2.016881232,2.3862311992,0.8601410443
C,0,0.9022731383,2.8704211508,-1.3982546596
C,0,1.5097675275,4.12254502,-1.4766926712
C,0,2.4658348458,4.5019199767,-0.5331798227
C,0,2.8040622635,3.6193349532,0.4939922297
C,0,2.1904967094,2.3700362584,0.5795389464
C,0,-3.8580213137,-0.8569922616,1.6012467024
C,0,-2.5038853472,-3.6366646184,1.2346548654
C,0,-3.6068752762,-2.0629384024,-1.2228490649
C,0,4.7470422176,-1.6396547668,0.0774392759
C,0,-1.7447065329,-3.5629080701,2.5772782571
C,0,-1.7652138511,-4.5932003548,0.2723376948
C,0,-3.9042550397,-4.2336924118,1.5101864292
C,0,3.2384666862,-1.8541741545,0.2544859358
O,0,2.7047437581,-2.9062047985,0.5227176504
C,0,5.4638100876,-2.9722621212,0.3459881525
C,0,5.2348428909,-0.5626671958,1.0716363964
C,0,5.0173955468,-1.1716731847,-1.3708637001
H,0,0.4175165463,-2.5709340603,0.5979799924
H,0,-0.185926101,0.3897845284,-1.2690587225
H,0,-2.7154350211,0.9574348128,-2.1422438886
H,0,-4.4107914351,2.7643767934,-2.1645426351
H,0,-4.5811329398,4.3350063348,-0.242744567
H,0,-3.0350031207,4.0894037036,1.690235023
H,0,-1.3397860527,2.2830645712,1.7032461419
H,0,0.1679094984,2.5844372777,-2.1464543429
H,0,1.2399123551,4.7992824469,-2.2830380974
H,0,2.9433858549,5.475751284,-0.5985285073
H,0,3.5439203482,3.904934814,1.2372431136
H,0,2.447524457,1.6941274613,1.3888232071
H,0,-4.0253168632,0.1473585077,1.1999624837
H,0,-3.419077676,-0.7429953159,2.599420993
H,0,-4.8380705226,-1.3340222433,1.7198433445
H,0,-4.5270716335,-2.6528988019,-1.1330376077
H,0,-2.9644228902,-2.5556381222,-1.9615246178
H,0,-3.8861403243,-1.0826767483,-1.6245026628
H,0,-2.2627613461,-2.9204224275,3.299917371
H,0,-0.7266993809,-3.1774860935,2.463271456
H,0,-1.6690563151,-4.5636289956,3.0276700538
H,0,-0.7536786414,-4.2535932141,0.0273610093
H,0,-2.3089979363,-4.7186712706,-0.6719009246
H,0,-1.6709800779,-5.5902743054,0.727393574
H,0,-4.5088421261,-4.3139116998,0.5984620749
H,0,-4.4703321696,-3.6408882658,2.2376393595
H,0,-3.8058102523,-5.2482445752,1.9232651727
H,0,5.1226263901,-3.7519146943,-0.3414397901
H,0,5.2763565211,-3.3254808796,1.3645877795
H,0,6.5444251077,-2.8437040902,0.2169333743
H,0,5.0338753261,-0.8583886097,2.1080775766
H,0,4.7507814704,0.4002469507,0.8863935034
H,0,6.3176805509,-0.4284273441,0.9658623651
H,0,4.515490437,-0.2236247917,-1.583495229
H,0,4.6726673691,-1.9158326427,-2.0982674311
H,0,6.0947460183,-1.0325875446,-1.5181279882
```

G(au)= -1528.087100
HF(au)= -1528.5868687|
$\bar{\nu}_i$(cm^{-1})= -1234.0914

TS2

```
C,0,-2.5511867595,-2.634007415,-0.5800300528
C,0,-1.4067720137,-1.9038436031,-0.2613273209
C,0,-3.6936959478,-1.9051612158,-0.1507961577
Si,0,-5.4303252674,-2.6861460119,-0.0709228357
O,0,-0.0803555398,-2.1700100014,-0.5669390016
C,0,-3.1502113613,-0.6141108097,0.427668587
C,0,-1.7323764298,-0.6712595613,0.3464787895
C,0,-3.9439333518,0.3646661494,1.2196913081
C,0,-0.7874873854,0.3844499614,0.7600956586
C,0,-3.7559684989,0.4392609938,2.6092377003
C,0,-4.4883141638,1.3412522016,3.3797462768
```

TS3

```
C,0,3.4925692127,-2.2480113099,-3.6644475914
C,0,4.5454486878,-1.348981766,-3.3958797648
C,0,2.2960875614,-1.4240950761,-3.7814901063
Si,0,2.229487624,-2.5290880306,-1.8448115762
O,0,5.8927025657,-1.6047506144,-3.1876449776
C,0,2.6671316412,-0.0638391172,-3.5982617985
C,0,4.0650843419,-0.0218144713,-3.3483271808
C,0,1.7065206323,1.0589104938,-3.6092920612
C,0,4.910524626,1.1747505167,-3.1271546065
C,0,0.6662063132,1.0888468426,-4.5568249946
C,0,-0.2751032474,2.1174803812,-4.5636066594
```

C,0,-5.4245470127,2.1846840153,2.7776201408
C,0,-5.6150464168,2.1261485053,1.3966600732
C,0,-4.8751225101,1.2288877328,0.6247404553
C,0,-1.0338114214,1.7374048356,0.4680142171
C,0,-0.1435501778,2.7319155662,0.8700827213
C,0,1.0173624207,2.3984945791,1.5699481931
C,0,1.2759433795,1.0591665919,1.8683034454
C,0,0.3834355342,0.0645131802,1.4711462219
C,0,-5.149982744,-4.5104557947,0.356741193
C,0,-6.3175607027,-2.530671182,-1.780662542
C,0,-6.4921603262,-1.9233707328,1.296993497
C,0,1.845294985,-3.2789110654,-1.4299536931
C,0,-5.501946847,-3.2466901243,-2.878831174
C,0,-6.48801909,-1.0500537699,-2.1778876992
C,0,-7.716228256,-3.1814052869,-1.6920449348
C,0,0.3404921926,-3.3369389276,-1.1380309709
O,0,-0.3845485632,-4.2734098708,-1.3824432131
C,0,2.2807707072,-4.6302069008,-2.0181180409
C,0,2.6172009227,-2.9937610414,-0.1228634389
C,0,2.1087812919,-2.1448119296,-2.4472268525
H,0,-2.5678539008,-3.5829774731,-1.0891430632
H,0,-3.6805605773,-0.7528493754,-0.7762161491
H,0,-3.0287176026,-0.215295997,3.0806935297
H,0,-4.327755187,1.3839904086,4.4537737968
H,0,-5.9949803501,2.8869010231,3.3794648528
H,0,-6.3311309622,2.7870094313,0.9152183057
H,0,-5.0159131721,1.2059083668,-0.4531634981
H,0,-1.9279619622,2.0071830755,-0.0854901475
H,0,-0.3560118551,3.7706795079,0.6301130495
H,0,1.7122703207,3.173957625,1.8811107436
H,0,2.171999605,0.7870951531,2.4206493463
H,0,0.5822853784,-0.9717391044,1.7252630386
H,0,-4.6429442629,-4.5981953435,1.3246281953
H,0,-4.5323550265,-5.0304854878,-0.3828958569
H,0,-6.1031214504,-5.0472522304,0.4329972852
H,0,-5.9549957341,-1.9231124807,2.2518434936
H,0,-7.4053844854,-2.5162288048,1.4299419418
H,0,-6.785780934,-0.8901299634,1.090827433
H,0,-5.3966348016,-4.3199975411,-2.6802714298
H,0,-4.4944061558,-2.826105662,-2.9812688099
H,0,-6.0019840862,-3.1440383224,-3.8530823742
H,0,-5.5203165684,-0.5488389428,-2.3092840888
H,0,-7.0633201532,-0.4848551797,-1.4345858652
H,0,-7.0221868999,-0.9690641469,-3.1358109865
H,0,-8.3592097488,-2.6774230442,-0.9605070211
H,0,-7.662364291,-4.2418441416,-1.4172871366
H,0,-8.2246000822,-3.1247806008,-2.6656421615
H,0,1.7364609135,-4.8561254676,-2.9398051451
H,0,2.0937023665,-5.4480044484,-1.3153245077
H,0,3.3527389143,-4.6060460556,-2.2443569113
H,0,2.4213254983,-3.7650163304,0.6314314948
H,0,2.3460054791,-2.0213427521,0.2966661485
H,0,3.694289172,-2.9912312352,-0.3266405411
H,0,1.8015276865,-1.1739303424,-2.0489071212
H,0,1.5690472036,-2.3206705466,-3.3850818903
H,0,3.1791930651,-2.1001506392,-2.6794205005

G(au)= -1528.094173
HF(au)= -1528.594727
$\bar{\nu}_i$(cm^{-1})= -1179.6105

C,0,-0.1958411668,3.1484648439,-3.6258749404
C,0,0.8334359702,3.1377370209,-2.6822017333
C,0,1.7717438539,2.1067100992,-2.6721014091
C,0,4.8456660069,2.2645582851,-4.0124963614
C,0,5.6491917835,3.3881219788,-3.8256785884
C,0,6.5386122961,3.4472564815,-2.750717969
C,0,6.6161899702,2.3720667151,-1.8640208192
C,0,5.811369137,1.2477460069,-2.0508697688
C,0,3.2024662743,-4.0822221216,-1.3168757777
C,0,0.3565911102,-3.0495445774,-1.9015319328
C,0,2.542957741,-1.1802191941,-0.5628058379
C,0,7.9828108966,-2.75047924,-3.2188889395
C,0,0.0854775181,-4.0400228988,-3.0539452475
C,0,-0.578947255,-1.8317608402,-2.0397686856
C,0,0.0213854295,-3.7595153498,-0.5665657058
C,0,6.4649238729,-2.8178113923,-3.425025862
O,0,5.8444883682,-3.8060104598,-3.7515365111
C,0,8.5714141348,-4.1489427624,-3.4629935387
C,0,8.286313209,-2.290650023,-1.7754801723
C,0,8.578805156,-1.7375812189,-4.2233844388
H,0,3.6102485294,-3.2589360454,-4.024059251
H,0,1.3862639266,-1.7506550159,-4.2695912381
H,0,0.6104865095,0.3056573283,-5.3083660291
H,0,-1.0662462668,2.1176933055,-5.3092186179
H,0,-0.9264643178,3.9529241277,-3.6318313156
H,0,0.904416209,3.9334786942,-1.9449394667
H,0,2.5607265715,2.1053740173,-1.9271212342
H,0,4.1613588764,2.2215725187,-4.8543308491
H,0,5.5834203101,4.2178056594,-4.5248919326
H,0,7.1659673617,4.322885664,-2.6061265536
H,0,7.3025714806,2.4083512864,-1.0217125204
H,0,5.8743776308,0.4183350974,-1.3527138208
H,0,4.2777090986,-3.8818379219,-1.3130697124
H,0,3.0360722963,-4.9271889045,-1.9945005283
H,0,2.9077938931,-4.3974625079,-0.3082305333
H,0,2.3974050027,-1.6037642307,0.4387067316
H,0,1.8689265385,-0.3273323617,-0.6793773614
H,0,3.570011539,-0.8074306114,-0.6155429084
H,0,0.7140342234,-4.9360356833,-2.9849125392
H,0,0.2510628712,-3.5924248397,-4.0412891517
H,0,-0.9615140563,-4.3748969211,-3.0270970072
H,0,-0.4146600577,-1.2706494767,-2.9648614465
H,0,-0.4592885075,-1.129052842,-1.2071971554
H,0,-1.6276823881,-2.1627792717,-2.0382971665
H,0,0.2118984471,-3.1189000285,0.3034313095
H,0,0.5885195883,-4.6868798963,-0.4349566133
H,0,-1.0456109901,-4.0240178531,-0.5446155881
H,0,8.3565340387,-4.4982599484,-4.4772316053
H,0,8.1553430318,-4.88285201,-2.7658899684
H,0,9.6585683868,-4.1209334424,-3.3285459575
H,0,7.851314243,-2.9766859182,-1.0390420161
H,0,7.8965224291,-1.2867614058,-1.5863566692
H,0,9.3707633776,-2.2728791726,-1.616511327
H,0,8.1753501244,-0.7339302195,-4.0641019281
H,0,8.3677835933,-2.0344360926,-5.2573058906
H,0,9.6674641907,-1.6969262798,-4.1015615814

G(au)= -1528.114586
HF(au)= -1528.6182745|
$\bar{\nu}_i$(cm^{-1})= -287.7207

TS4

C,0,0.787206232,-1.8821683342,-0.819253072
C,0,0.2088275813,-0.6867469835,-0.3107342631
C,0,0.3192570913,-1.8960759162,-2.2455423699
Si,0,1.5116168651,-3.4114336907,0.0514535543
O,0,0.5503179953,-0.1186541357,0.919636023
C,0,-0.4868162595,-0.7675367481,-2.486654329
C,0,-0.5529575302,-0.0198267858,-1.2788777229
C,0,-1.1112366405,-0.4705211567,-3.7898789801
C,0,-1.1982148739,1.3059034715,-1.097132741
C,0,-0.4333103197,-0.7733603484,-4.9848847542
C,0,-1.0232417664,-0.5413023309,-6.2260902303
C,0,-2.3047012198,0.0073399155,-6.3030588804
C,0,-2.9887285294,0.3164568646,-5.125588137
C,0,-2.4023679674,0.0790311051,-3.8836287376
C,0,-0.7584339613,2.4222048702,-1.8260950573

TS5

C,0,-0.1718650457,-1.4039277245,-0.6029503882
C,0,0.995572825,-0.6473196822,-0.4561919751
C,0,-1.3355688794,-0.4733112829,-0.4033428162
Si,0,-3.1342468698,-1.011437385,-0.0309027056
O,0,2.2722295216,-1.1339007763,-0.721313127
C,0,-0.7355988762,0.8123471775,-0.196007595
C,0,0.6763866802,0.6946439329,-0.203543522
C,0,-1.4691447431,2.0893038928,-0.0149963829
C,0,1.6728910114,1.7844556183,-0.0684778883
C,0,-2.3412859046,2.5752420964,-1.0035515272
C,0,-3.0284504602,3.7779205765,-0.8284455889
C,0,-2.8487159522,4.5252597822,0.3362388969
C,0,-1.9727809634,4.0627820915,1.3211885364
C,0,-1.2901021038,2.8596354374,1.1470880595
C,0,1.6293925059,2.9232640763,-0.8879484631

C,0,-1.3696409435,3.6649870792,-1.6640280679
C,0,-2.4357658544,3.8127937448,-0.774113508
C,0,-2.8852744088,2.7089374911,-0.0476963273
C,0,-2.2709568365,1.4646606253,-0.205230135
C,0,1.4589029491,-3.1927710794,1.9254786674
C,0,3.3305787428,-3.7064538658,-0.527578077
C,0,0.4134325802,-4.8833362993,-0.4139034497
C,0,0.1348651356,0.6322555661,3.1578810681
C,0,4.2094960934,-2.4916116262,-0.1612575862
C,0,3.4053779319,-3.9347196783,-2.0525151389
C,0,3.8904065663,-4.9598949973,0.1818839153
C,0,-0.308521887,-0.2435962983,1.9786272526
O,0,-1.2899939655,-0.9503712115,1.9538764436
C,0,-0.8142213842,0.3780501735,4.3397680342
C,0,0.0599402986,2.1149204526,2.7241566342
C,0,1.5864064041,0.2925503029,3.559942762
H,0,1.5272886215,-1.5368454746,-1.8263223985
H,0,0.4421929751,-2.7490701174,-2.900853548
H,0,0.5739265211,-1.1788926375,-4.9341770678
H,0,-0.476450581,-0.7812150108,-7.1345990347
H,0,-2.7647497487,0.1931088533,-7.2699255746
H,0,-3.9892659726,0.7388305019,-5.1723612515
H,0,-2.9524582525,0.3073335792,-2.9767639974
H,0,0.0666322853,2.309620171,-2.5238386374
H,0,-1.013687066,4.5186332336,-2.2350480185
H,0,-2.9144962856,4.780741676,-0.6509108388
H,0,-3.7201941212,2.8127543083,0.6408366251
H,0,-2.6237003621,0.6060705947,0.3581157463
H,0,0.4414804749,-2.965302548,2.2590908598
H,0,2.1175592231,-2.3895054465,2.2688500018
H,0,1.7729852003,-4.1194462622,2.4205190171
H,0,0.7285021565,-5.7952548545,0.1069717063
H,0,0.4220868349,-5.0938281251,-1.4891339654
H,0,-0.6246189563,-4.6791389888,-0.1265414845
H,0,4.2392990927,-2.3181310283,0.9209657035
H,0,3.8534744203,-1.5680962641,-0.6340622868
H,0,5.245060997,-2.6526920097,-0.4942505945
H,0,3.0641999671,-3.0611751916,-2.6224814982
H,0,2.8083102888,-4.797490437,-2.3721814844
H,0,4.4440402593,-4.126682873,-2.3583906669
H,0,3.3137395315,-5.861782481,-0.0571018086
H,0,3.8991366779,-4.8467688593,1.2724173832
H,0,4.9274853612,-5.1447704201,-0.1337099608
H,0,-0.7870892794,-0.6699835873,4.6544341027
H,0,-1.8485326936,0.6166181965,4.0756244742
H,0,-0.5199840476,1.0022797664,5.1910549347
H,0,-0.9545853318,2.3930541,2.4211092675
H,0,0.7307157074,2.3168693444,1.884679604
H,0,0.3528052643,2.7545849505,3.5649413797
H,0,2.278404436,0.4485776742,2.727609758
H,0,1.675432156,-0.7462046003,3.8959828139
H,0,1.8956221277,0.939736091,4.3889159497

G(au)= -1528.094900
HF(au)= =-1528.5969006
$\bar{\nu}_i$(cm^{-1})= -1241.1740

C,0,2.5839569078,3.9312819667,-0.7569042596
C,0,3.5977695864,3.820917266,0.1968709672
C,0,3.6487111356,2.6952895507,1.0209405678
C,0,2.6950670548,1.6851234283,0.8899913479
C,0,-3.6486996317,-0.1824579736,1.5910690817
C,0,-3.2807739137,-2.9323828977,0.1550904744
C,0,-4.2925938321,-0.446000002,-1.4235772608
C,0,4.3142124839,-2.2654046694,-0.201311936
C,0,-2.3825759723,-3.4590287051,1.295638412
C,0,-2.9428660597,-3.6664433485,-1.1618636675
C,0,-4.7483031974,-3.2697792633,0.5139637361
C,0,2.9573399746,-1.7547448478,0.2921718266
O,0,2.5165149632,-1.8715245019,1.4109097567
C,0,5.0718056952,-2.8741913004,0.9890644446
C,0,5.1179920769,-1.0924540431,-0.8050506958
C,0,4.0711493015,-3.3417586561,-1.2843596728
H,0,-0.2371572758,-2.4806100038,-0.6585643912
H,0,-0.9119300428,-0.9252812746,-1.5687969076
H,0,-2.4622699169,2.0125261888,-1.9248637043
H,0,-3.6962351972,4.1351332589,-1.6084234174
H,0,-3.381609353,5.462393751,0.4733699964
H,0,-1.8219957021,4.6393641254,2.230199524
H,0,-0.6130389621,2.50285732,1.917690976
H,0,0.8436483197,3.014710711,-1.631842288
H,0,2.5356931851,4.8045169537,-1.4024202491
H,0,4.3403096408,4.6080485583,0.2988738086
H,0,4.428444304,2.6050060926,1.7731373148
H,0,2.7255689806,0.8182816929,1.5440169023
H,0,-3.5522933958,0.9052865025,1.5213107324
H,0,-3.0267340767,-0.5153829862,2.4296578521
H,0,-4.6929823944,-0.4111489026,1.8348644632
H,0,-5.3119801519,-0.8167764636,-1.2636715951
H,0,-3.9604827382,-0.8003370895,-2.4066944655
H,0,-4.3431094553,0.6464878584,-1.4622811526
H,0,-2.6564252198,-3.0164535857,2.2605236817
H,0,-1.3200931534,-3.2515796246,1.1333372265
H,0,-2.4943149138,-4.5486005958,1.3951889048
H,0,-1.9064833263,-3.516140085,-1.4843220039
H,0,-3.5927727835,-3.346169112,-1.9851632663
H,0,-3.0848857271,-4.7501207669,-1.0392911711
H,0,-5.4508671691,-2.9522618423,-0.2653583072
H,0,-5.0602356328,-2.8071898205,1.4572931398
H,0,-4.8648213181,-4.3568706854,0.6316444583
H,0,4.5107622662,-3.6994840941,1.4374116511
H,0,5.2446503011,-2.1291303008,1.7719177127
H,0,6.0433021831,-3.2549342448,0.6542170652
H,0,5.2785684011,-0.2979382319,-0.0679129304
H,0,4.6023073842,-0.6559156308,-1.6641886493
H,0,6.0995975551,-1.4527337003,-1.1342130963
H,0,3.530311577,-2.9288667454,-2.1407117408
H,0,3.4951862651,-4.1847309755,-0.8846544533
H,0,5.0327883339,-3.7306059216,-1.6386220944

G(au)= -1528.089911
HF(au)= -1528.5906047
$\bar{\nu}_i$(cm^{-1})= -1251.7016

TS6

C,0,0.2041340679,-1.8075372384,-1.1160523131
C,0,-0.8486704138,-0.9654970873,-0.5354855975
C,0,1.3860578294,-1.0259570594,-1.184393414
Si,0,2.9716146857,-1.7327678204,-1.9273527715
O,0,-2.2041251044,-1.2816998763,-0.4966276615
C,0,1.051633628,0.2643989444,-0.6791818187
C,0,-0.3089631402,0.3237309476,-0.2893562622
C,0,1.9833166391,1.4197830848,-0.5579801242
C,0,-1.0523613985,1.4502118137,0.3166157316
C,0,3.0585240054,1.4082172003,0.3437004605
C,0,3.9175928554,2.5038499912,0.4482278581
C,0,3.7156127873,3.6324150681,-0.347878888
C,0,2.6457131874,3.659448339,-1.2446771732
C,0,1.7865252017,2.565452851,-1.3453137052
C,0,-0.5194565851,2.1873460199,1.3881727408
C,0,-1.2309983894,3.2438358886,1.9551080405
C,0,-2.4934644224,3.5866726058,1.4677955421
C,0,-3.0364658866,2.8649699525,0.4032029236
C,0,-2.3231156971,1.8108372996,-0.1673695196
C,0,2.4585242435,-2.7191646175,-3.4715508886

TS7

C,0,-2.6600885123,-0.5910334227,-3.4173616286
C,0,-1.5367041673,0.2472309596,-3.2834168357
C,0,-3.0401663537,-0.9589254965,-2.1203171632
Si,0,-1.5130559136,-2.4825970084,-1.3416910083
O,0,-0.7575993458,0.8490820729,-4.2599495145
C,0,-2.1412458553,-0.3214173955,-1.1515249294
C,0,-1.1948664978,0.4376193352,-1.9356199062
C,0,-2.5467293174,-0.0177744656,0.2533921213
C,0,-0.0912342196,1.3122185275,-1.4635504294
C,0,-3.9111869769,0.0633654349,0.5881514251
C,0,-4.3221911018,0.3648219742,1.8866942866
C,0,-3.3788188158,0.6047774775,2.8855714525
C,0,-2.0204846456,0.5436401243,2.5680031377
C,0,-1.609676747,0.2357231569,1.2722174447
C,0,-0.3666181044,2.5030953004,-0.7707439524
C,0,0.6648397718,3.3436797797,-0.3523135942
C,0,1.9945812683,3.0138374581,-0.6219804006
C,0,2.2845964807,1.8379725217,-1.3158794639
C,0,1.2511354193,0.9973832076,-1.7328989716
C,0,-0.4606702782,-2.7873610674,-2.8842410441

C,0,3.8649945523,-2.9347128292,-0.6977569536	C,0,-2.760954008,-3.9553070801,-1.0718445987
C,0,4.1562138944,-0.3729827454,-2.5064127113	C,0,-0.2918928879,-2.520569136,0.1190458305
C,0,-4.1339012246,-2.4148171327,0.3812629733	C,0,0.0435446556,1.518557367,-6.3986707689
C,0,2.9796794612,-4.1666389376,-0.4139060922	C,0,-3.5496172061,-4.2844204021,-2.3556698675
C,0,4.171627063,-2.2298888943,0.6389357427	C,0,-3.7488822572,-3.6736512358,0.079408284
C,0,5.1952817656,-3.4117606998,-1.3211443637	C,0,-1.929264912,-5.2068059152,-0.6946665183
C,0,-2.6119669483,-2.2944337834,0.3372059231	C,0,-0.9570558476,0.6760295671,-5.5975290917
O,0,-1.8234998722,-2.9739872511,0.9550136039	O,0,-1.8097032946,-0.0397188723,-6.071695198
C,0,-4.5003042977,-3.7363357277,1.0768929649	C,0,-0.1883707487,1.2611148788,-7.8957090876
C,0,-4.7163279846,-2.3819143146,-1.0474826454	C,0,1.4830428861,1.1187748021,-6.0047219178
C,0,-4.6739748522,-1.2168076264,1.1998785925	C,0,-0.1887269374,3.0116229172,-6.0720951184
H,0,-0.1909504433,-1.850058385,0.16923812	H,0,-3.0907850359,-0.9374574737,-4.342104675
H,0,0.0243663908,-2.7992782821,-1.5100849405	H,0,-3.9821346585,-1.4209563501,-1.8561629975
H,0,3.2151970394,0.5361760389,0.971414535	H,0,-4.6599773132,-0.0871431918,-0.1828258099
H,0,4.7429516182,2.4757992022,1.1552776917	H,0,-5.3840009475,0.4223558495,2.1121489288
H,0,4.3845381454,4.4852635645,-0.2680440424	H,0,-3.6968055101,0.8424840025,3.8971094784
H,0,2.4786363242,4.5345928293,-1.8673999141	H,0,-1.2725664138,0.7348254453,3.3332454375
H,0,0.9530454687,2.5909689651,-2.0416229202	H,0,-0.5507276582,0.1948716204,1.0482111396
H,0,0.4583890372,1.9255555738,1.7791472347	H,0,-1.399269025,2.7679496031,-0.5644632896
H,0,-0.79795681,3.7979391441,2.7839955461	H,0,0.4283376434,4.2607778288,0.1811886413
H,0,-3.0473907872,4.4099440965,1.9111487503	H,0,2.7980272157,3.6694432139,-0.2965457001
H,0,-4.0142859606,3.1289650049,0.0078946769	H,0,3.3162714943,1.5723879325,-1.5324585191
H,0,-2.7443850965,1.2663939583,-1.0069825054	H,0,1.4839456216,0.0842663774,-2.2738729197
H,0,2.0016810415,-2.0528209046,-4.2128650352	H,0,0.226443091,-1.9589166028,-3.0776309287
H,0,1.7329055649,-3.5101661821,-3.2492755725	H,0,-1.05517884,-2.946534821,-3.7867749242
H,0,3.3256583819,-3.1949408348,-3.9457389852	H,0,0.1486389483,-3.6828866761,-2.7070320827
H,0,4.9384687963,-0.8070273532,-3.1409879931	H,0,0.2608182213,-3.4671890418,0.0898085918
H,0,4.6440550862,0.1571385424,-1.6833565064	H,0,-0.771513139,-2.4391768688,1.0982517226
H,0,3.6229870147,0.3758347896,-3.1027296038	H,0,0.4392268722,-1.710052934,0.0320373133
H,0,2.7713451746,-4.7407825232,-1.3247826226	H,0,-2.8860125156,-4.5448325144,-3.1884667157
H,0,2.0188010431,-3.8869220642,0.0347874021	H,0,-4.1836897319,-3.4553433919,-2.6864076169
H,0,3.4823556489,-4.8458554778,0.2905299402	H,0,-4.2080384318,-5.1475824171,-2.1810919793
H,0,3.2545182236,-1.9034355471,1.1439752872	H,0,-4.4271113472,-2.842469877,-0.1424966909
H,0,4.813074233,-1.3507431038,0.5019083621	H,0,-3.2344980088,-3.4338600377,1.0174752412
H,0,4.6971320255,-2.9134410622,1.3223473904	H,0,-4.3735657756,-4.5597450731,0.2644829573
H,0,5.8875816745,-2.5796739118,-1.4963317429	H,0,-1.4043516283,-5.0850981882,0.2585169985
H,0,5.0429500327,-3.927324753,-2.2772960862	H,0,-1.1863448438,-5.4599101671,-1.4611408646
H,0,5.7007315786,-4.1201420255,-0.6480391518	H,0,-2.5947729332,-6.075920115,-0.5913114351
H,0,-4.0819260773,-3.7829283203,2.0859520113	H,0,-1.2078354568,1.5268648189,-8.190682575
H,0,-4.1203732621,-4.5983172848,0.5185959998	H,0,-0.0370055016,0.206488811,-8.1459337368
H,0,-5.5899305373,-3.8259708319,1.1463787191	H,0,0.5119149577,1.8613082573,-8.4875816982
H,0,-4.3254655973,-3.2048786089,-1.6568314711	H,0,1.6701990059,0.0576638589,-6.2081463928
H,0,-4.4866105563,-1.4418705405,-1.5552993499	H,0,1.6729585288,1.3066037748,-4.9444098838
H,0,-5.8056025834,-2.4899842335,-0.99758966	H,0,2.2002893774,1.7034415347,-6.5925592944
H,0,-4.3998454486,-0.2608024149,0.7447439697	H,0,-0.0269614571,3.2164488828,-5.0103025926
H,0,-4.2845421379,-1.2318877125,2.223858956	H,0,-1.2079581994,3.3202058095,-6.3322073375
H,0,-5.7670598962,-1.276133717,1.2527873161	H,0,0.5073248652,3.6274267552,-6.6535067636
**G(au)= -1528.089760**	**G(au)= -1528.104241**
**HF(au)= -1528.5895002**	**HF(au)= -1528.6091633**
**$\bar{\nu}_i$(cm^{-1})= -1257.9302**	**$\bar{\nu}_i$(cm^{-1})= -254.5925**

10. Experimental Procedure for the Synthesis of 4a (2.5 mmol Scale)

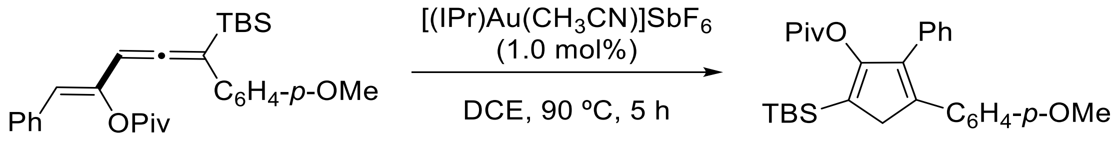

3a
(2.5 mmol)

4a
1.052 g (91%)

[(IPr)Au(CH_3CN)]SbF_6 (22.0 mg, 1.0 mol%) was added to a solution of vinylallene **3a** (1.156 g, 2.50 mmol) in DCE (2 mL). The resulting mixture was stirred at 90 ºC until disappearance of the starting vinyl allene (checked by TLC, 5 h). Then, the solvent was removed under reduced pressure and the resulting mixture was purified by flash chromatography (silica gel, hexanes/ethyl acetate 40:1) to yield cyclopentadiene **4a** (1.052 g, 91%). The spectroscopic data of compound **4a** match with those reported for the 0.20 mmol scale (see page S-16).

11. Product Derivatization

*Synthesis of 4-(4-methoxyphenyl)-5-phenylcyclopenta-1,4-dien-1-yl pivalate (**5a**)*

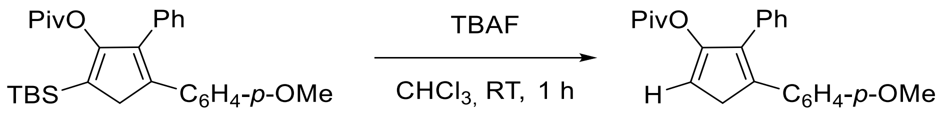

4a **5a**, 86%

To a solution of **4a** (138.8 mg, 0.3 mmol) in $CHCl_3$ (3 mL) was added a 1 M solution of TBAF in THF (0.9 mL, 0.9 mmol, 3 equiv). The resulting mixture was stirred for 1 h at room temperature and quenched with saturated aq. NH_4Cl. The aqueous layer was extracted with diethyl ether and the combined organic extracts were dried (Na_2SO_4), filtered and concentrated. The resulting residue was purified by flash chromatography (silica gel, hexanes/ethyl acetate 40:1) to afford 4-(4-methoxyphenyl)-5-phenylcyclopenta-1,4-dien-1-yl pivalate (**5a**, 89.9 mg, 86%) as a yellow oil.

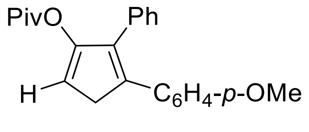

5a

^{1}H NMR (300 MHz, $CDCl_3$): δ = 7.40-7.31 (m, 5H), 7.18 (d, J = 8.9 Hz, 2H), 6.75 (d, J = 8.9 Hz, 2H), 6.17 (t, J = 2.0 Hz, 1H), 3.76 (s, 3H), 3.52 (d, J = 2.0 Hz, 2H),1.16 (s, 9H) ppm.

^{13}C NMR (75 MHz, $CDCl_3$): δ = 176.4 (C), 158.9 (C), 152.2 (C), 141.2 (C), 136.5 (C), 135.0 (C), 130.1 (CH), 129.3 (C), 129.0 (CH), 128.8 (CH), 127.7 (CH), 114.1 (CH), 112.7 (CH), 55.5 (CH_3), 39.6 (CH_2), 39.4 (C), 27.4 (CH_3) ppm.

Because of its low stability towards air and moisture, compound **5a** did not provide satisfactory high resolution mass spectra.

*Synthesis of 4-(4-methoxyphenyl)-2-oxo-3-phenylcyclopent-3-en-1-yl pivalate (**6a**)*

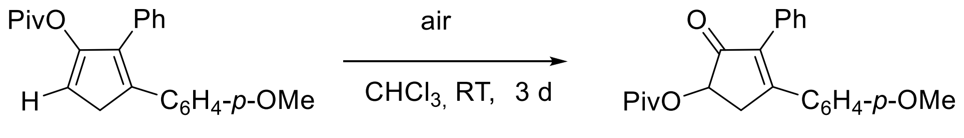

5a **6a**

When exposed to air a solution of cyclopentadiene **5a** (34.8 mg, 0.1 mmol) in $CHCl_3$ (2 mL), TLC analysis revealed the formation of a new product spot. After 3 days, the solvent was removed and the resulting residue was purified by flash chromatography (silica gel, hexanes/ethyl acetate 10:1) to afford cyclopentenone **6a** (22.2 mg, 61%) as a yellow oil.

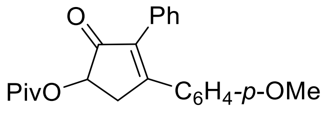

6a

^{1}H NMR (300 MHz, $CDCl_3$): δ = 7.37-7.28 (m, 7H), 6.81 (d, *J* = 8.9 Hz, 2H), 5.46 (dd, *J* = 7.2 and 3.4 Hz, 1H), 3.82 (s, 3H), 3.48 (dd, *J* = 17.4 and 7.2 Hz, 1H), 3.00 (dd, *J* = 17.4 and 3.4 Hz, 1H), 1.30 (s, 9H) ppm.

^{13}C NMR (75 MHz, $CDCl_3$): δ = 201.9 (C), 178.7 (C), 164.0 (C), 161.9 (C), 136.7 (C), 132.5 (C), 130.8 (CH), 129.9 (CH), 129.1 (CH), 128.5 (CH), 127.4 (C), 114.3 (CH), 72.5 (CH), 55.8 (CH_3), 39.3 (C), 36.9 (CH_2), 27.6 (CH_3) ppm.

HRMS (EI) m/z: $[M + K]^+$ Calcd for $C_{23}H_{24}KO_5$ 419.1255; found: 419.1266.

*Synthesis of 5-(tert-butyldimethylsilyl)-3-(4-methoxyphenyl)-2-phenylcyclopent-2-en-1-one (**7a**)*

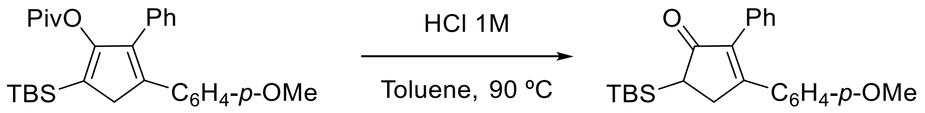

4a **7a**, 97%

To a solution of **4a** (92.5 mg, 0.2 mmol) in toluene (1 mL) was added 0.5 mL of a solution of 1M HCl. The resulting mixture was stirred for 1 h at 90 ºC. After removal of the solvent the resulting residue was purified by flash chromatography (silica gel, hexanes/ethyl acetate 10:1) to afford 4-(4-methoxyphenyl)-5-phenylcyclopenta-1,4-dien-1-yl pivalate **7a** (73.4 mg, 97%) as a yellow oil.

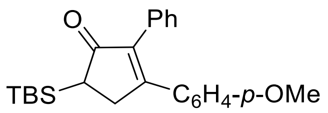

7a

^{1}H NMR (300 MHz, $CDCl_3$): δ = 7.39-7.21 (m, 7H), 6.81 (d, *J* = 8.7 Hz, 2H), 3.82 (s, 3H), 3.29 (dd, *J* = 18.5 and 6.5 Hz, 1H), 3.01 (d, *J* = 18.5 Hz, 1H), 2.60 (d, *J* = 6.5 Hz, 1H), 1.01 (s, 9H), 0.19 (s, 3H), 0.08 (s, 3H) ppm.

^{13}C NMR (75 MHz, $CDCl_3$): δ = 208.3 (C), 164.7 (C), 160.3 (C), 139.5 (C), 133.3 (C), 129.6 (CH), 129.5 (CH), 128.5 (CH), 128.2 (C), 127.6 (CH), 113.8 (CH), 55.3 (CH_3), 38.4 (CH), 33.4 (CH_2), 27.1 (CH_3), 18.0 (C), -6.5 (CH_3), -7.3 (CH_3) ppm.

HRMS (EI) m/z: $[M + H]^+$ Calcd for $C_{24}H_{31}O_2Si$ 379.2088; found: 379.2088.

*One-pot synthesis of 3-(4-methoxyphenyl)-2-phenylcyclopent-2-en-1-one (**8a**)*

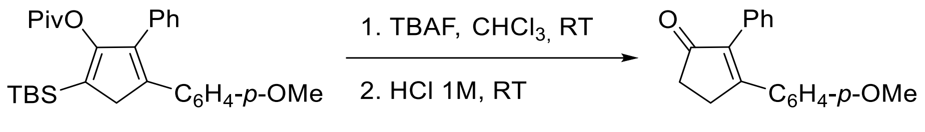

4a **8a**, 72%

To a solution of **4a** (46.3 mg, 0.1 mmol) in $CHCl_3$ (1 mL) was added a 1 M solution of TBAF in THF (0.3 mL, 0.3 mmol, 3 equiv). The resulting solution was stirred for 1 h at room temperature. To this solution was added 1mL of a solution of 1M HCl and the resulting mixture was stirred at RT overnight and quenched with saturated aq. NH_4Cl. The aqueous layer was extracted with diethyl ether and the combined organic extracts were dried (Na_2SO_4), filtered and concentrated. The resulting residue was purified by flash chromatography (silica gel, hexanes/ethyl acetate 10:1) to afford 4-(4-methoxyphenyl)-5-phenylcyclopenta-1,4-dien-1-yl pivalate 8**a**, (19.0 mg, 72%) as a yellow solid (m.p.: 131.3-132.5 ºC).

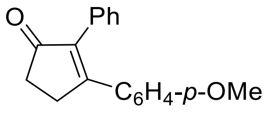

8a

^{1}H NMR (300 MHz, $CDCl_3$): δ = 7.41-7.22 (m, 7H), 6.80 (d, *J* = 9.0 Hz, 2H), 3.82 (s, 3H), 3.08-3.05 (m, 2H), 2.73-2.70 (m, 2H) ppm.

^{13}C NMR (75 MHz, $CDCl_3$): δ = 208.1 (C), 167.8 (C), 161.3 (C), 138.9 (C), 133.4 (C), 130.3 (CH), 129.9 (CH), 129.0 (CH), 128.3 (C), 128.2 (CH), 114.2 (CH), 55.7 (CH_3), 35.1 (CH_2), 29.6 (CH_2) ppm.

HRMS (EI) m/z: $[M + H]^+$ Calcd for $C_{18}H_{17}O_2$ 265,1223; found: 265,1219.

12. References

1. a) R. J. Detz, M. M. E. Delville, H. Hiemstra, J. H. van Maarseveen, *Angew. Chem. Int. Ed.* **2008**, *47*, 3777; b) E. Rettenmeier, A. M. Schuster, M. Rudolph, F. Rominger, C. A. Gade, A. S. K. Hashmi, *Angew. Chem. Int. Ed.* **2013**, *52*, 5880.
2. a) S. Chuprun, C. M. Acosta, L. Mathivathanan, K. V. Bukhryakov, *Organometallics* **2020**, *39*, 3453; b) A. A. Rajkiewicz, N. Wojciechowska, M. Kalek, *ACS Catal.* **2020**, *10*, 831.
3. O. Bernardo, S. González-Pelayo, I. Fernández, L. A. López, *Angew. Chem. Int. Ed.* **2021**, *60*, 25258.
4. Y. Zheng, J. Zhang, X. Cheng, X. Xu, L. Zhang, *Angew. Chem. Int. Ed.* **2019**, *58*, 5241.
5. For a good description of the computational methodology for the study of a chemical reaction, see: F. Jensen, *Introduction to Computational Chemistry*; Wiley, 2nd Ed., 2007.
6. O. Wiest, K. N. Houk, *Top. Curr. Chem.* **1996**, *183*, 1.
7. I. V. Alabugin, M. Manoharan, B. Breiner, F. D. Lewis, *J. Am. Chem. Soc.* **2003**, *125*, 9329, and references cited therein
8. *Gaussian 09*, Revision B.01, M. J. Frisch, G. W. Trucks, H. B. Schlegel, G. E. Scuseria, M. A. Robb, J. R. Cheeseman, G. Scalmani, V. Barone, B. Mennucci, G. A. Petersson, H. Nakatsuji, M. Caricato, X. Li, H. P. Hratchian, A. F. Izmaylov, J. Bloino, G. Zheng, J. L. Sonnenberg, M. Hada, M. Ehara, K. Toyota, R. Fukuda, J. Hasegawa, M. Ishida, T. Nakajima, Y. Honda, O. Kitao, H. Nakai, T. Vreven, J. A. Montgomery, Jr., J. E. Peralta, F. Ogliaro, M. Bearpark, J. J. Heyd, E. Brothers, K. N. Kudin, V. N. Staroverov, T. Keith, R. Kobayashi, J. Normand, K. Raghavachari, A. Rendell, J. C. Burant, S. S. Iyengar, J. Tomasi, M. Cossi, N. Rega, J. M. Millam, M. Klene, J. E. Knox, J. B. Cross, V. Bakken, C. Adamo, J. Jaramillo, R. Gomperts, R. E. Stratmann, O. Yazyev, A. J. Austin, R. Cammi, C. Pomelli, J. W. Ochterski, R. L. Martin, K. Morokuma, V. G. Zakrzewski, G. A. Voth, P. Salvador, J. J. Dannenberg, S. Dapprich, A. D. Daniels, O. Farkas, J. B. Foresman, J. V. Ortiz, J. Cioslowski, and D. J. Fox, Gaussian, Inc., Wallingford CT, 2010.
9. R. B. Woodward, R. Hoffmann, *The Conservation of Orbital Symmetry*; Academic Press, NY 1970.
10. J. Clarke, P. W. Fowler, S. Gronert, J. R. Keeffe *J. Org. Chem.* **2016**, *81*, 8777, and references cited therein.

14. NMR spectra for new compounds

^{1}H NMR spectrum ($CDCl_3$, 300 MHz) of compound **3b**

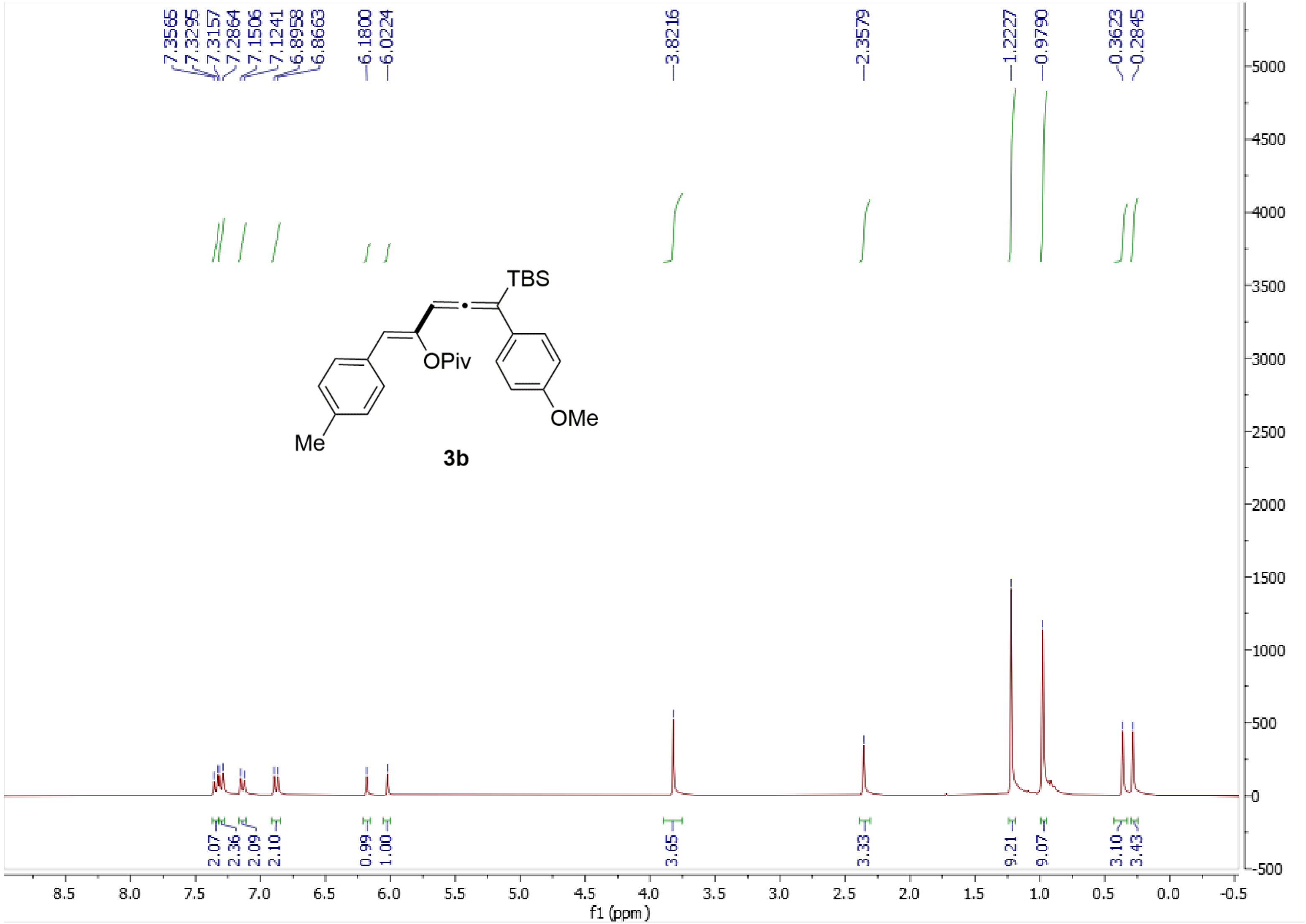

^{13}C NMR spectrum ($CDCl_3$, 75 MHz) of compound **3b**

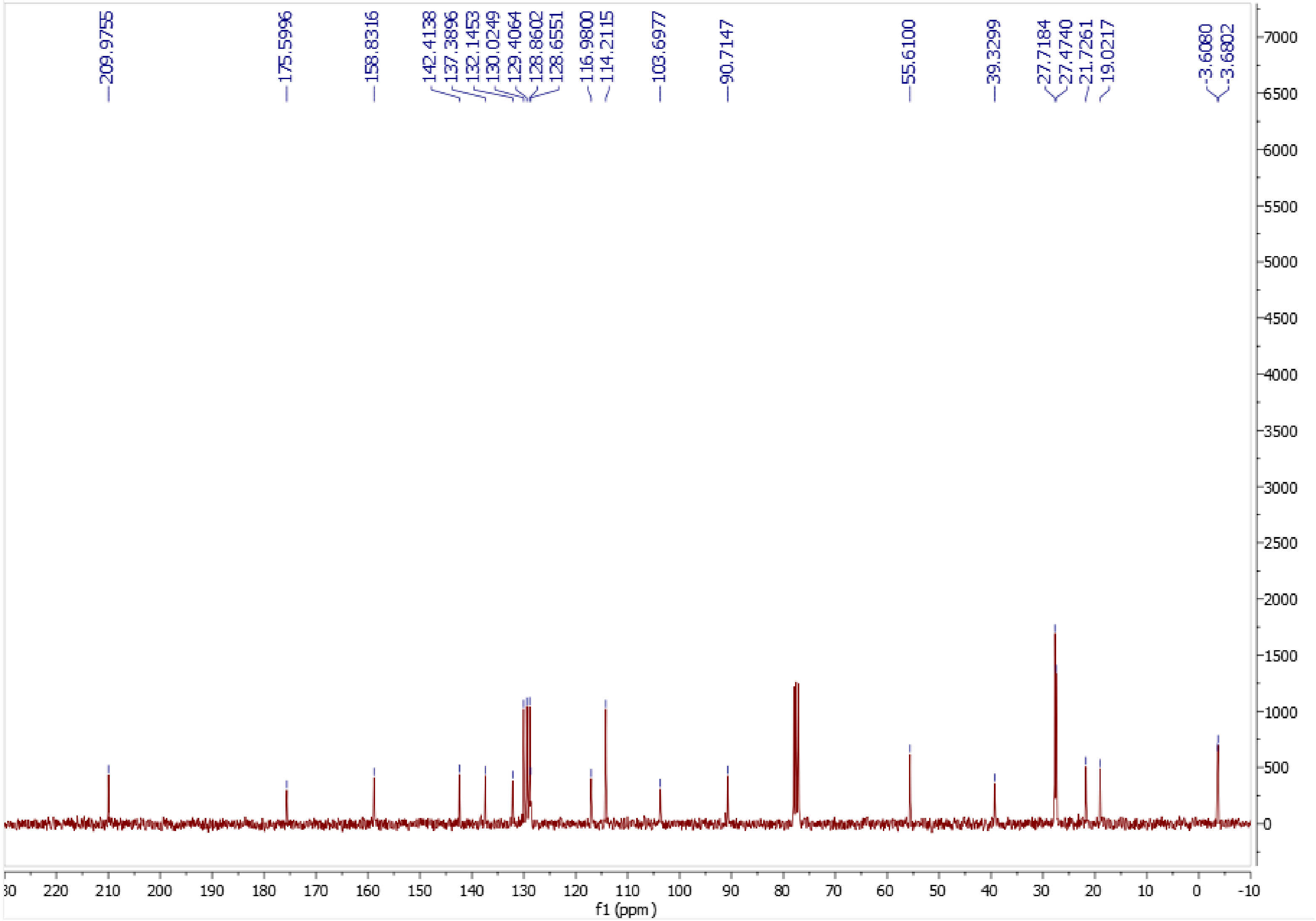

^{1}H NMR spectrum ($CDCl_3$, 300 MHz) of compound **3c**

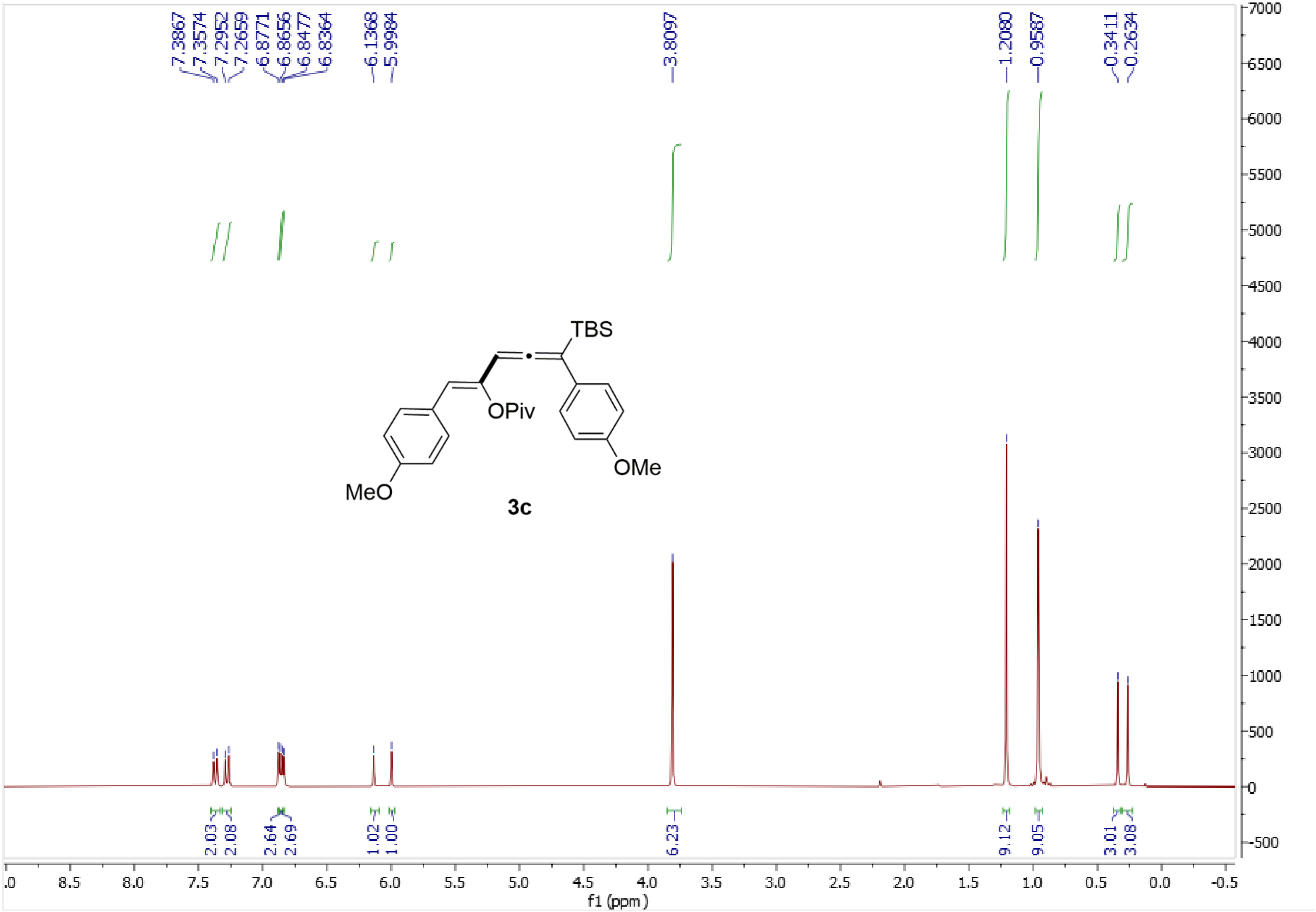

^{13}C NMR spectrum ($CDCl_3$, 75 MHz) of compound **3c**

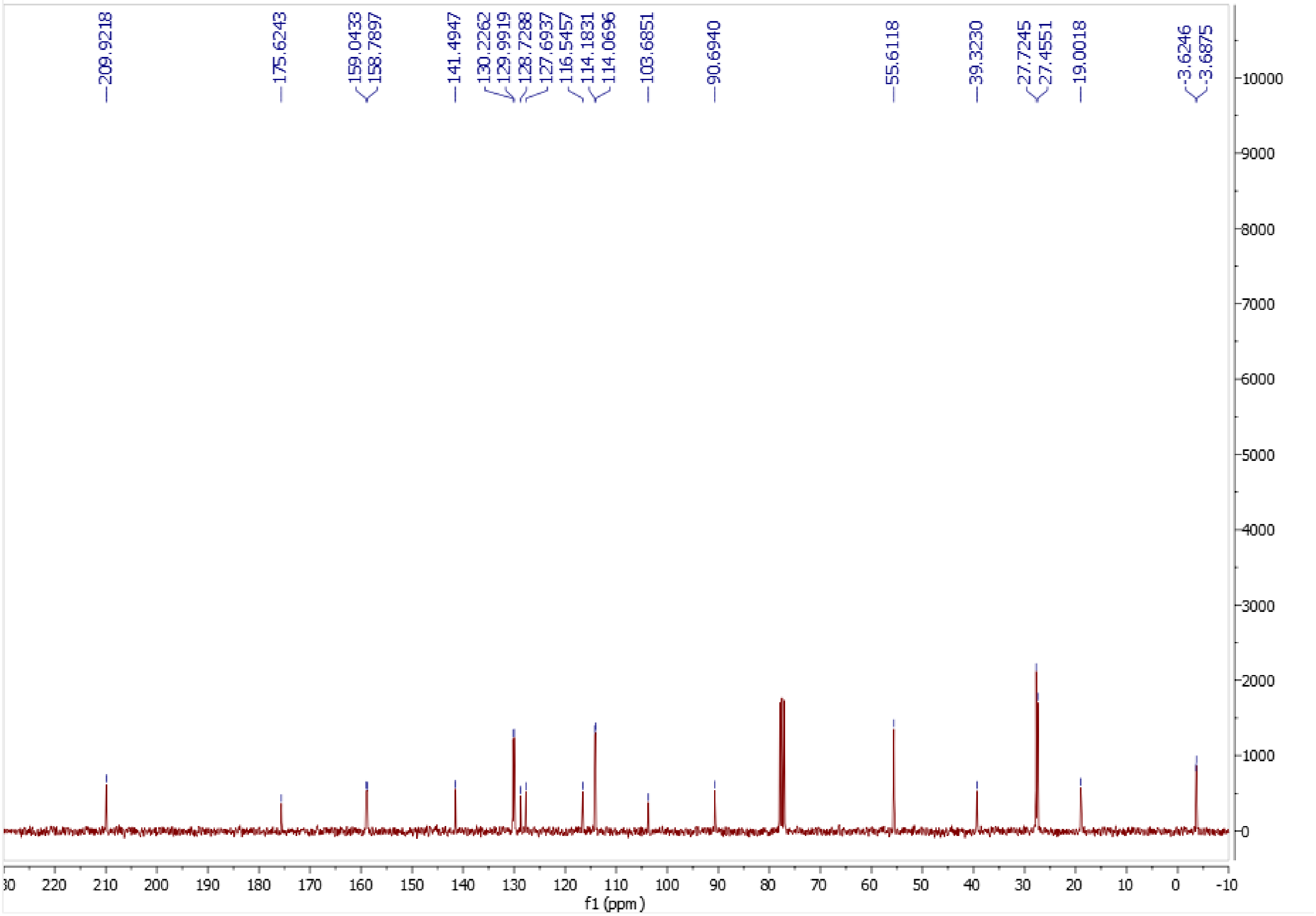

^{1}H NMR spectrum ($CDCl_3$, 300 MHz) of compound **3d**

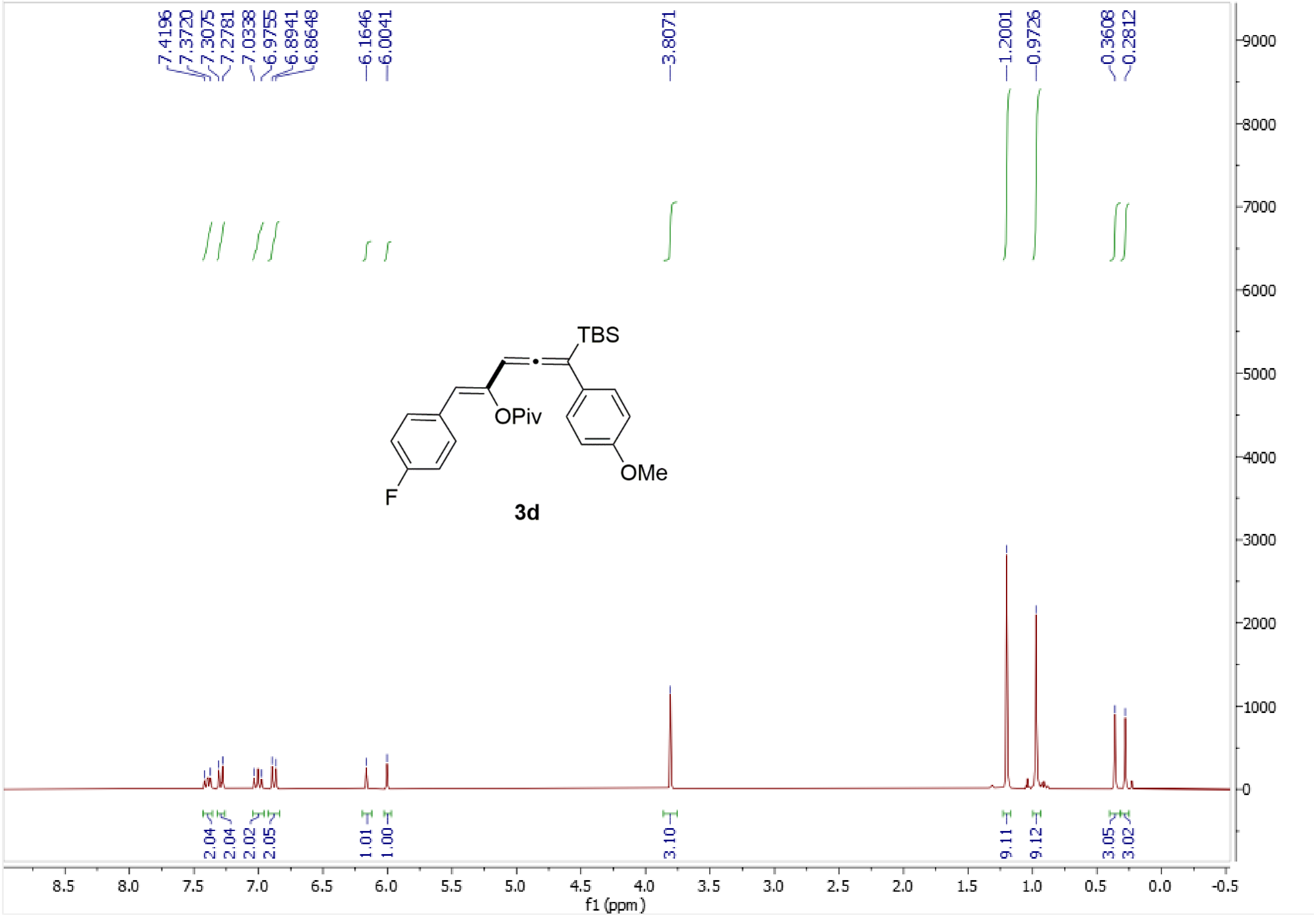

^{13}C NMR spectrum ($CDCl_3$, 75 MHz) of compound **3d**

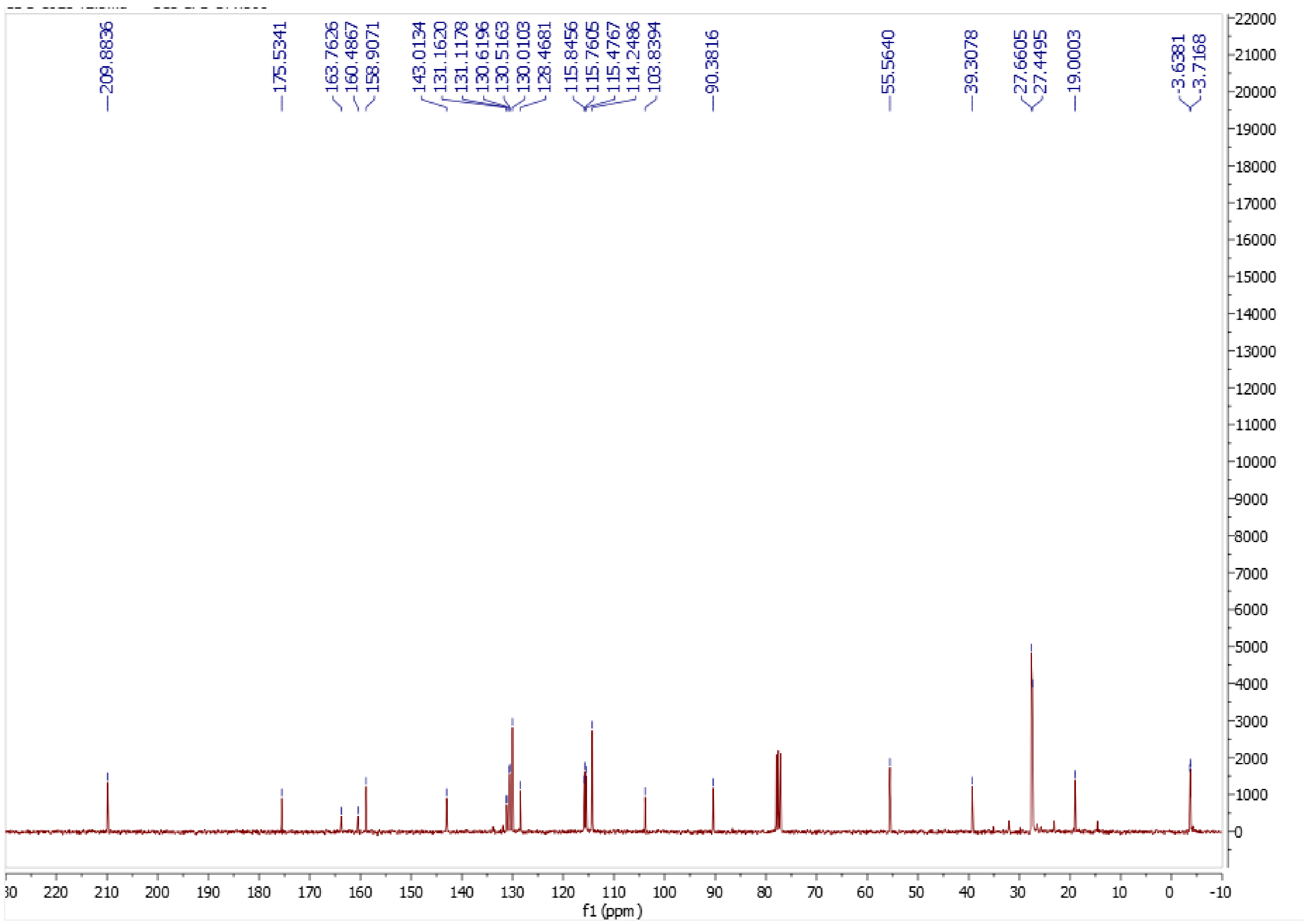

^{19}F NMR spectrum ($CDCl_{3,}$ 282 MHz) of compound **3d**

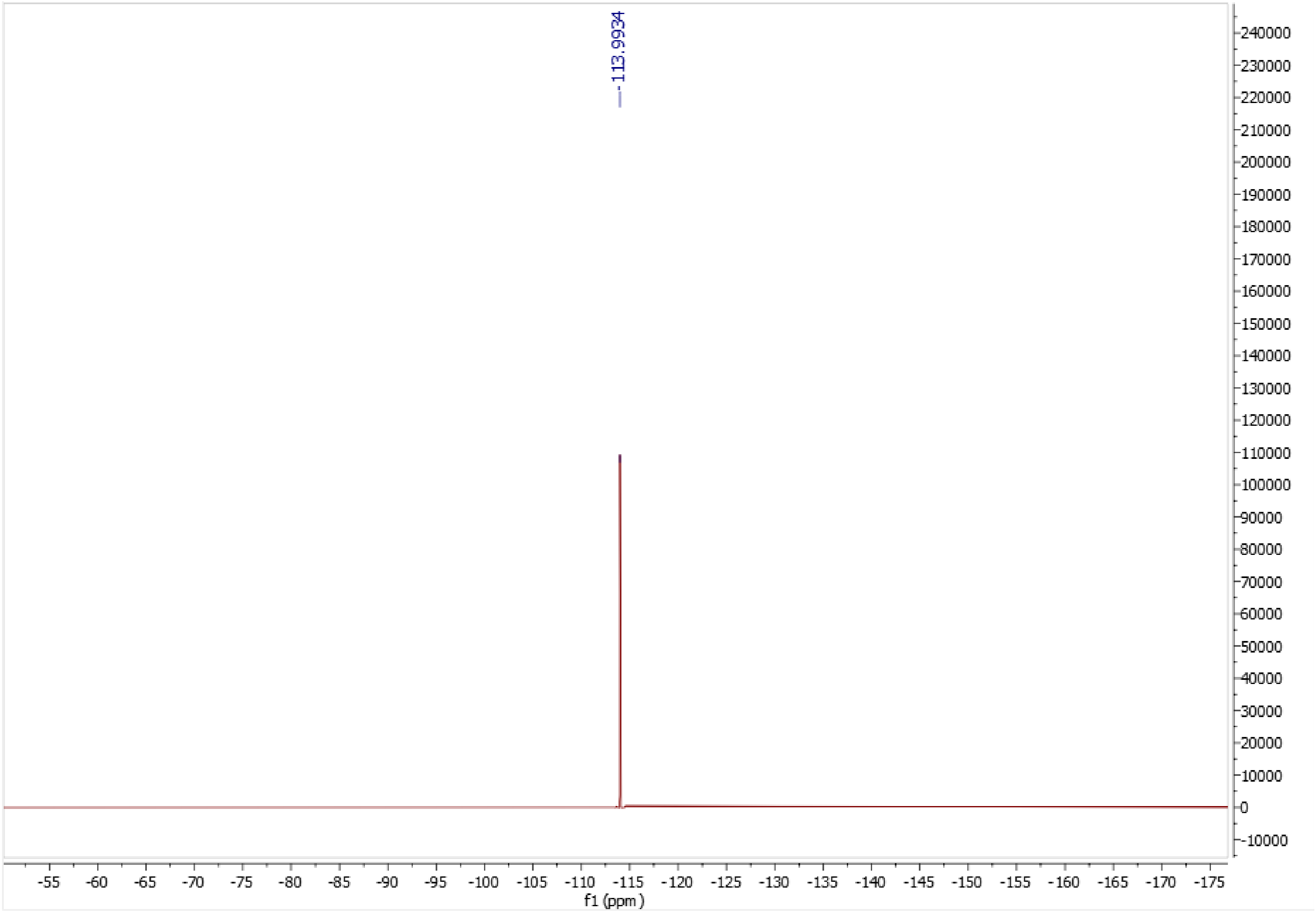

^{1}H NMR spectrum ($CDCl_3$, 300 MHz) of compound **3e**

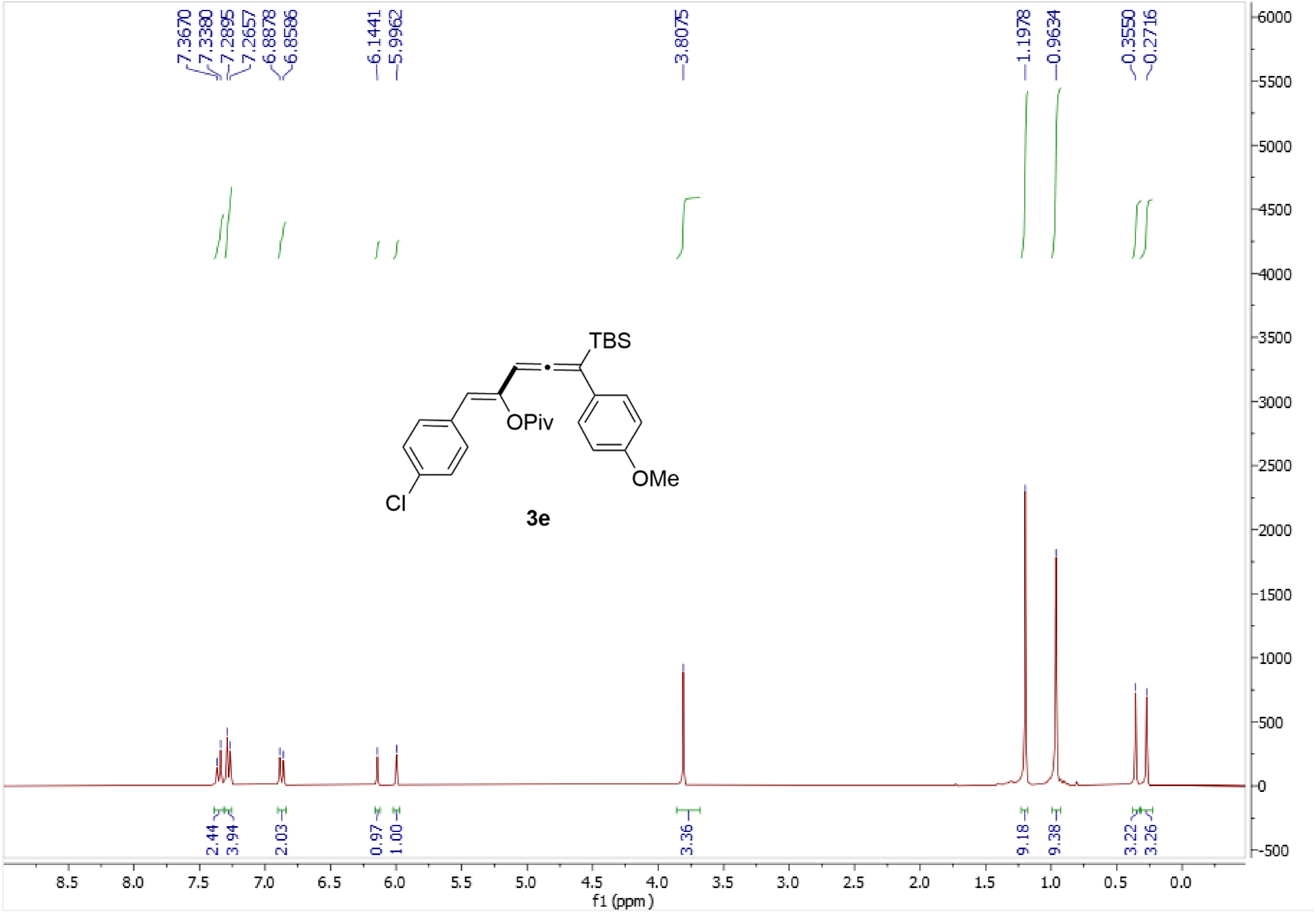

^{13}C NMR spectrum ($CDCl_3$, 75 MHz) of compound **3e**

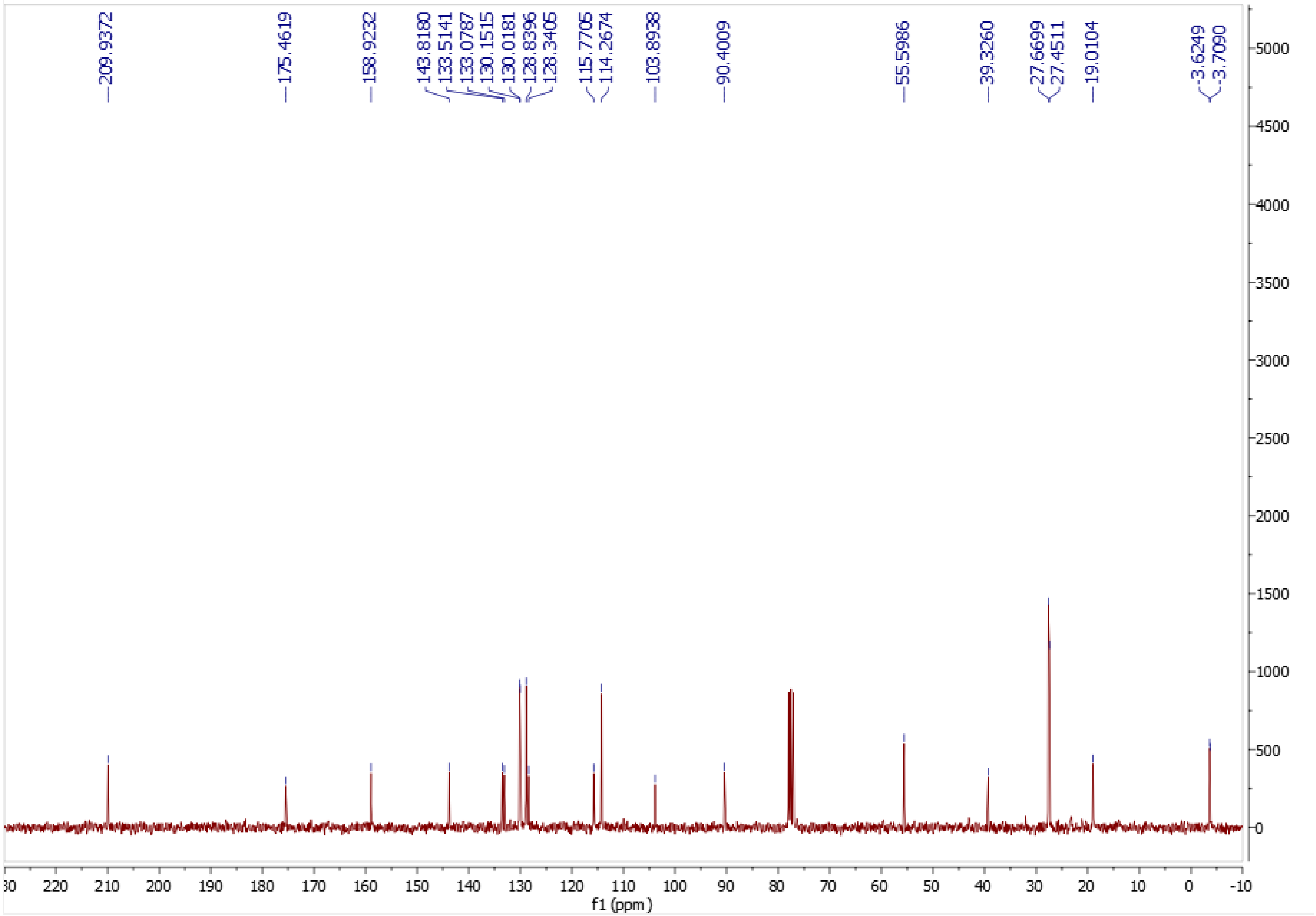

^{1}H NMR spectrum ($CDCl_3$, 300 MHz) of compound **3f**

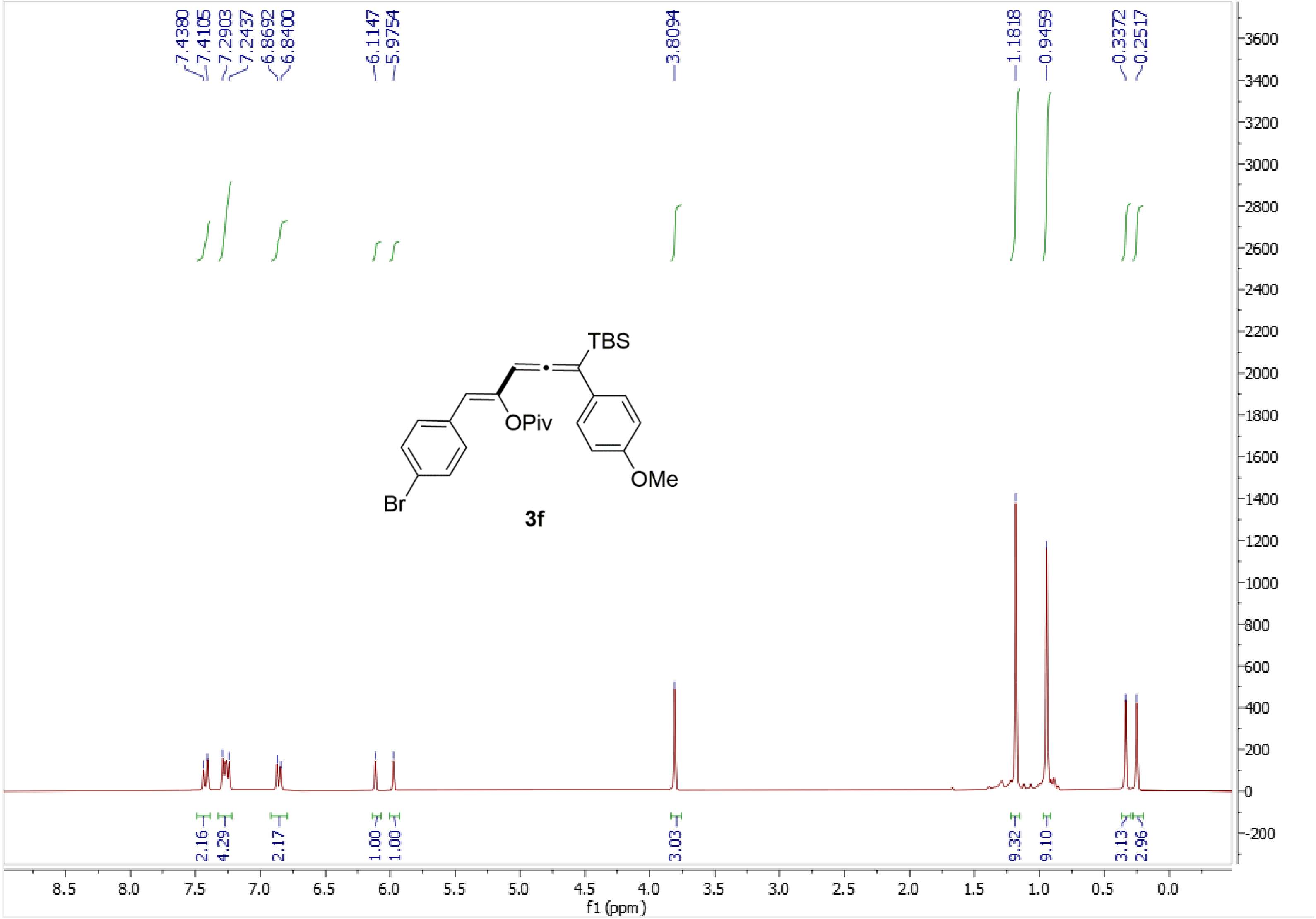

^{13}C NMR spectrum ($CDCl_3$, 75 MHz) of compound **3f**

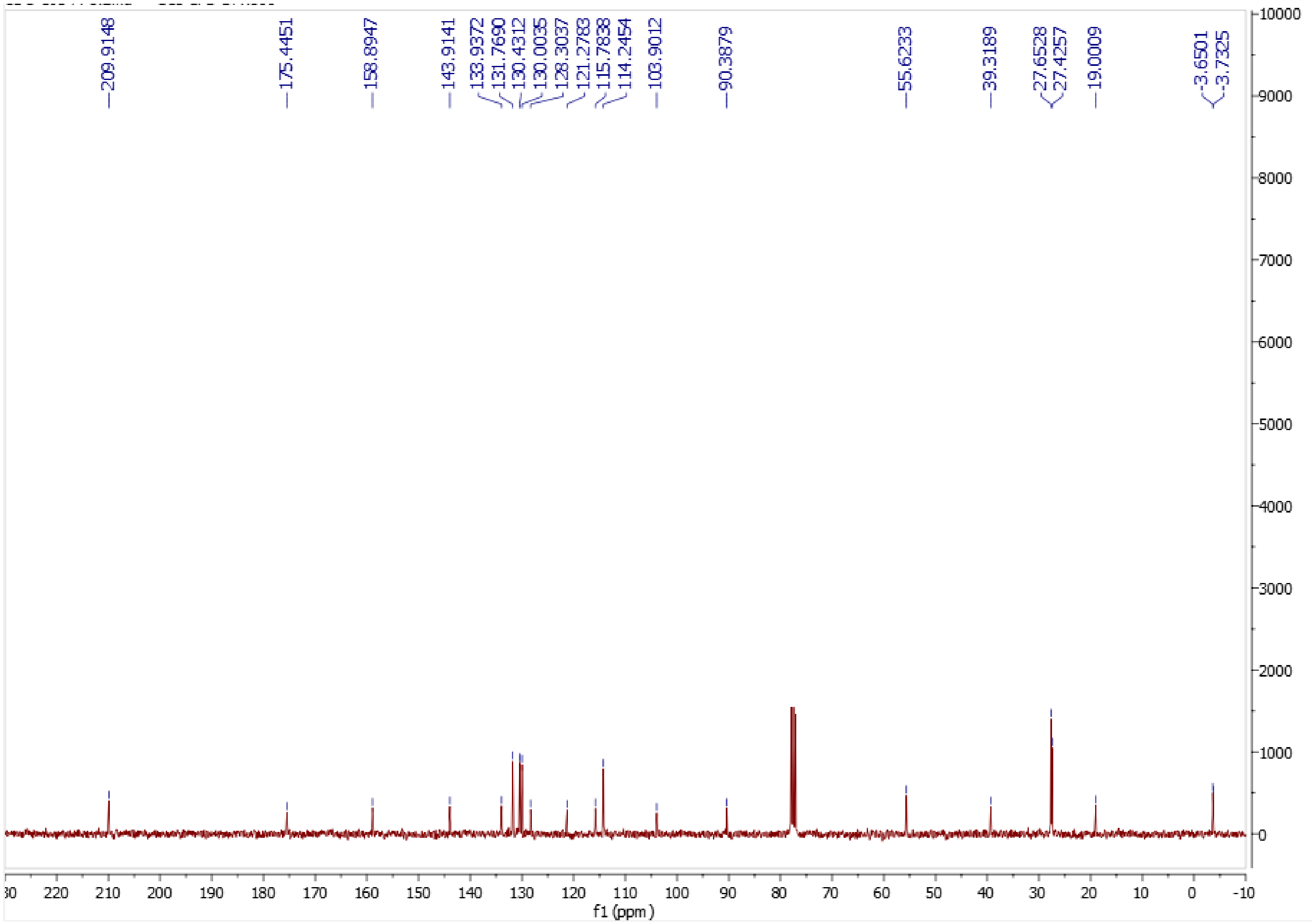

^{1}H NMR spectrum ($CDCl_3$, 300 MHz) of compound **3g**

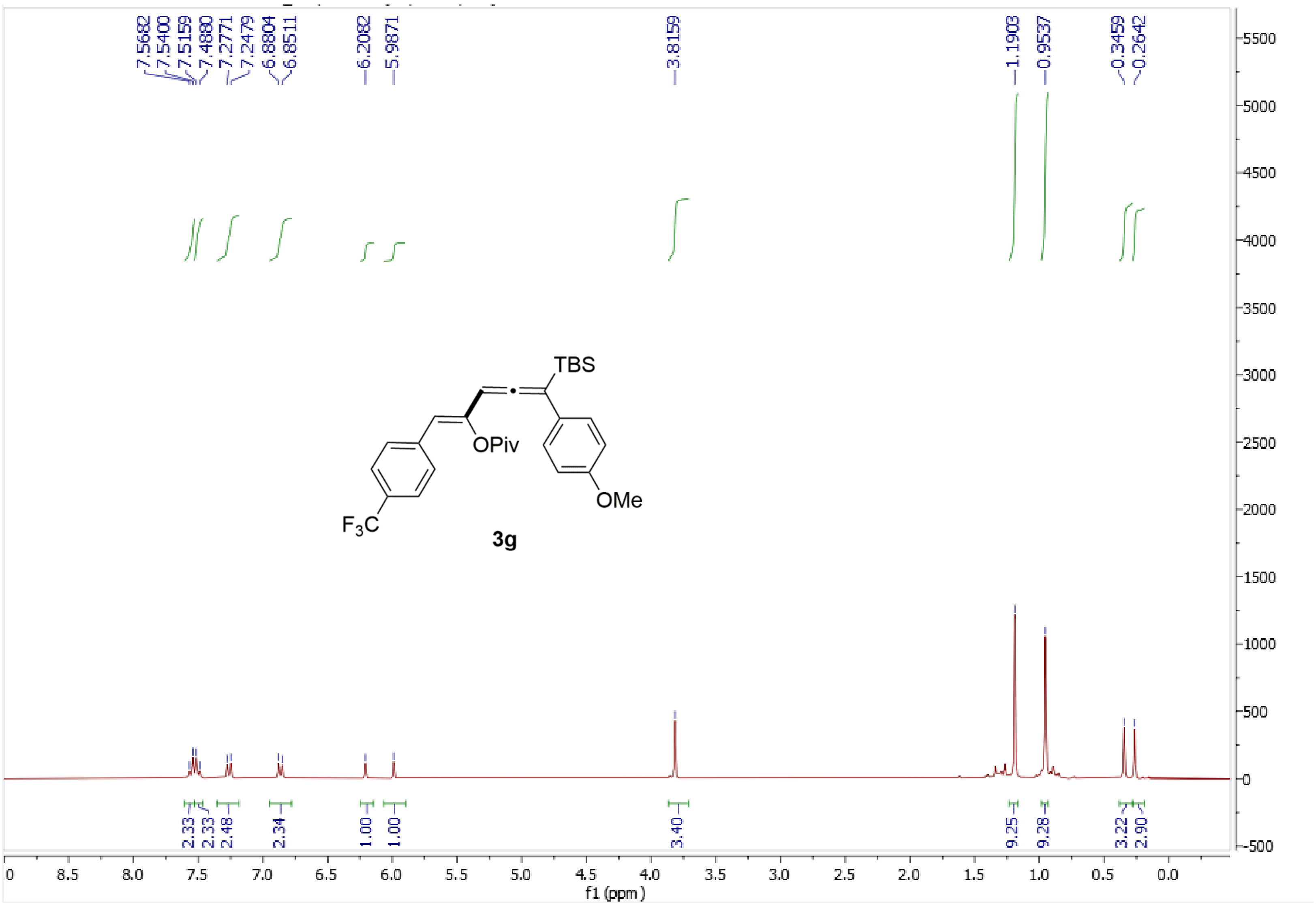

^{13}C NMR spectrum ($CDCl_3$, 75 MHz) of compound **3g**

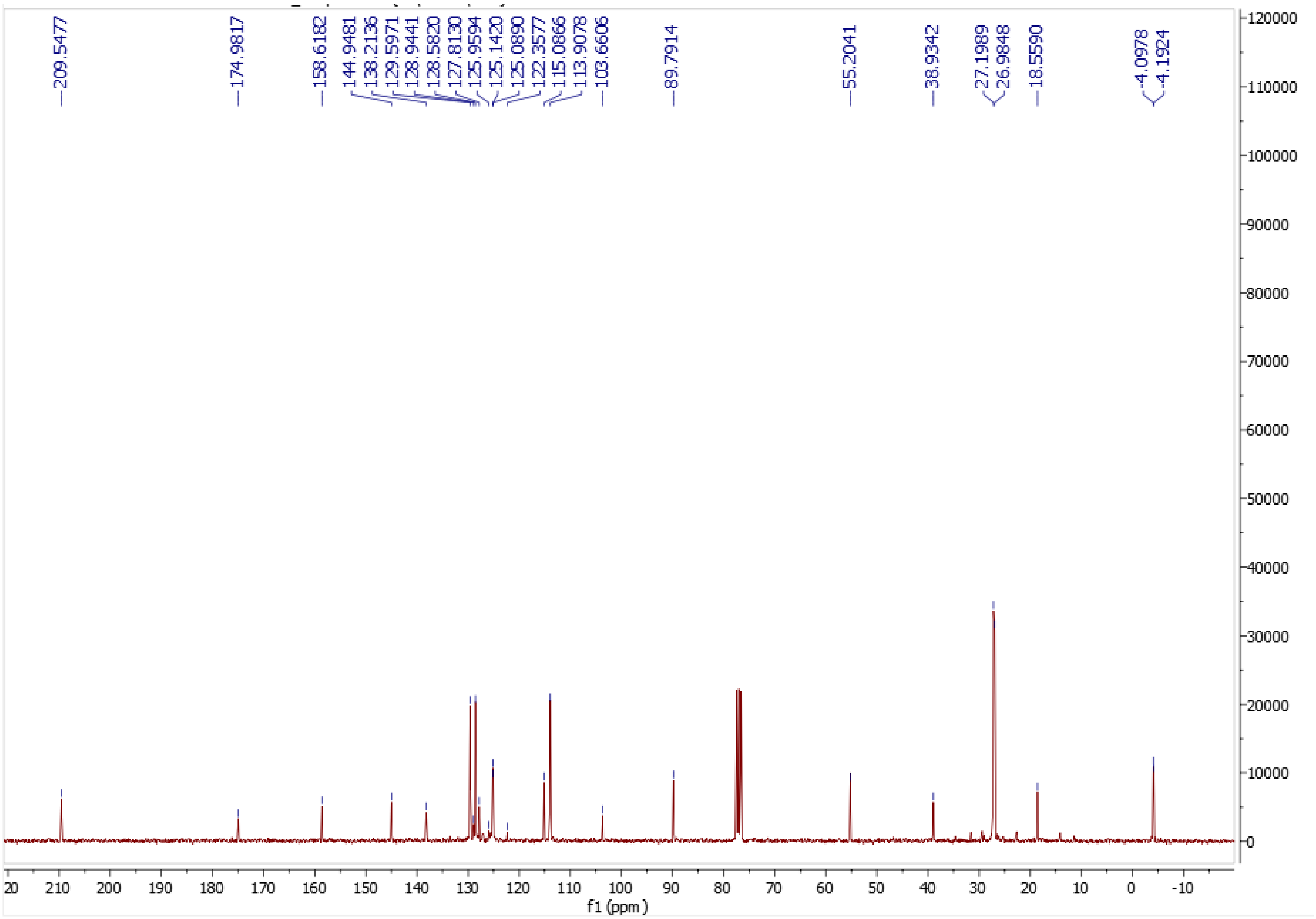

^{19}F NMR spectrum ($CDCl_3$, 282 MHz) of compound **3g**

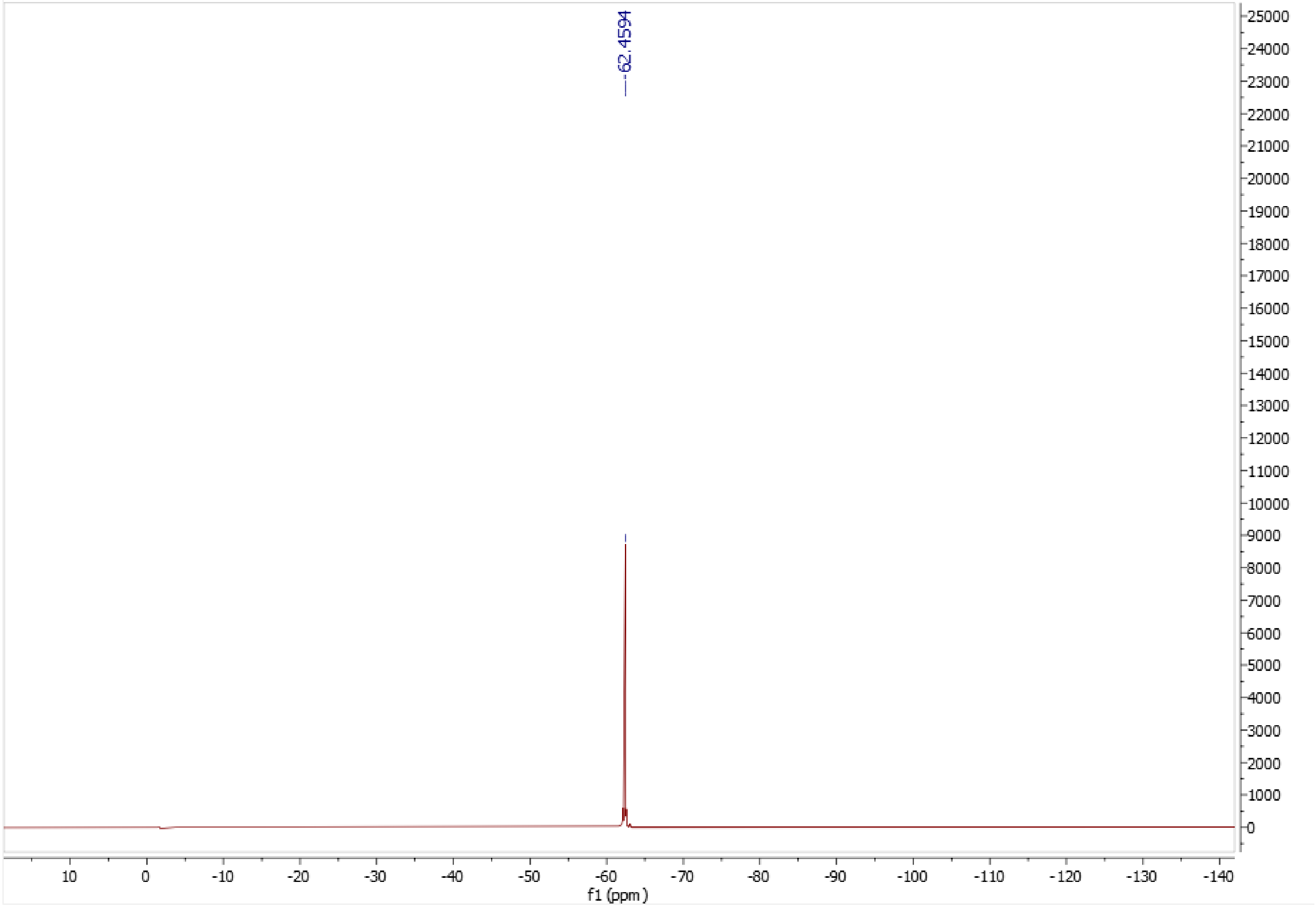

^{1}H NMR spectrum ($CDCl_3$, 300 MHz) of compound **3h**

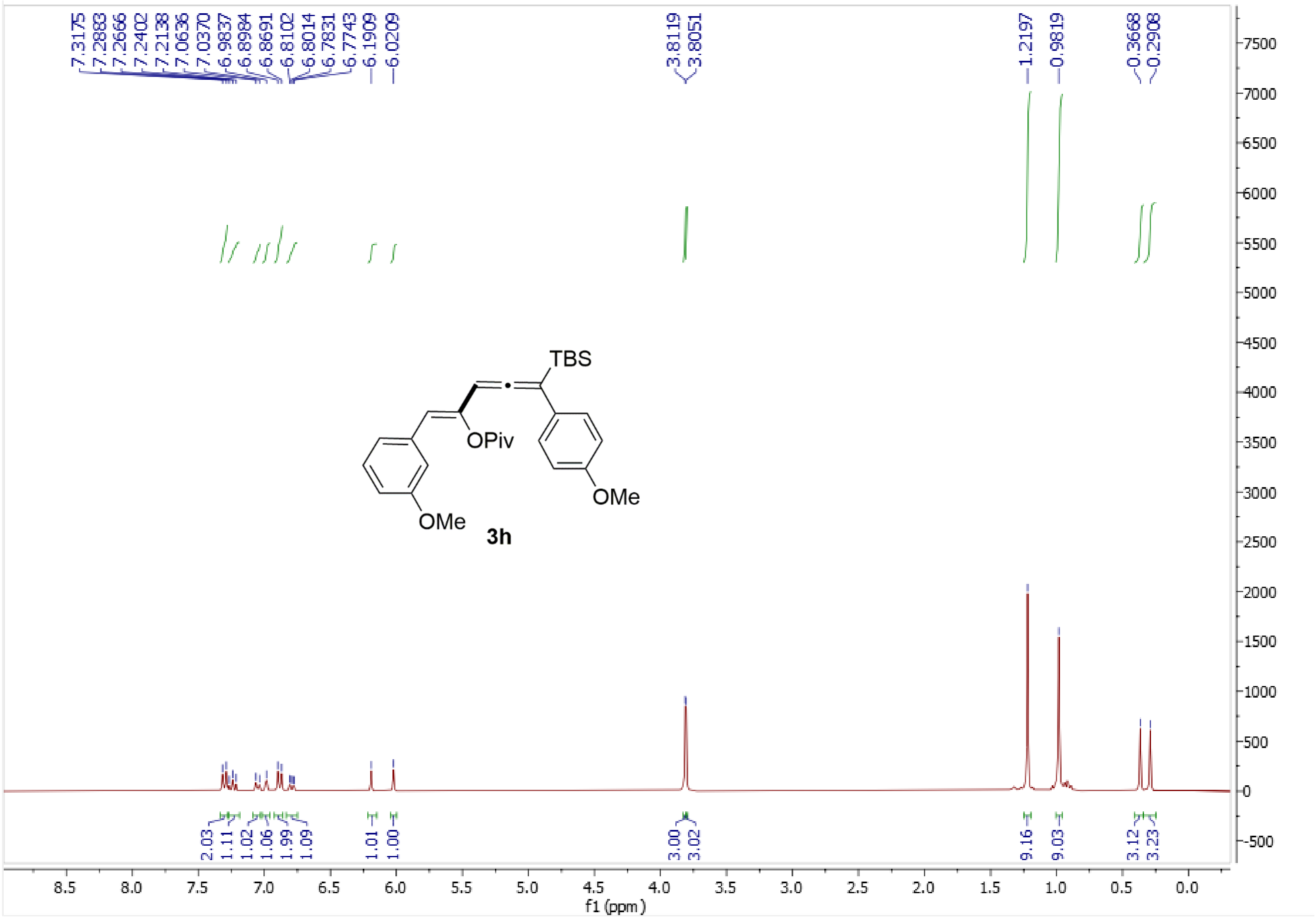

^{13}C NMR spectrum ($CDCl_3$, 75 MHz) of compound **3h**

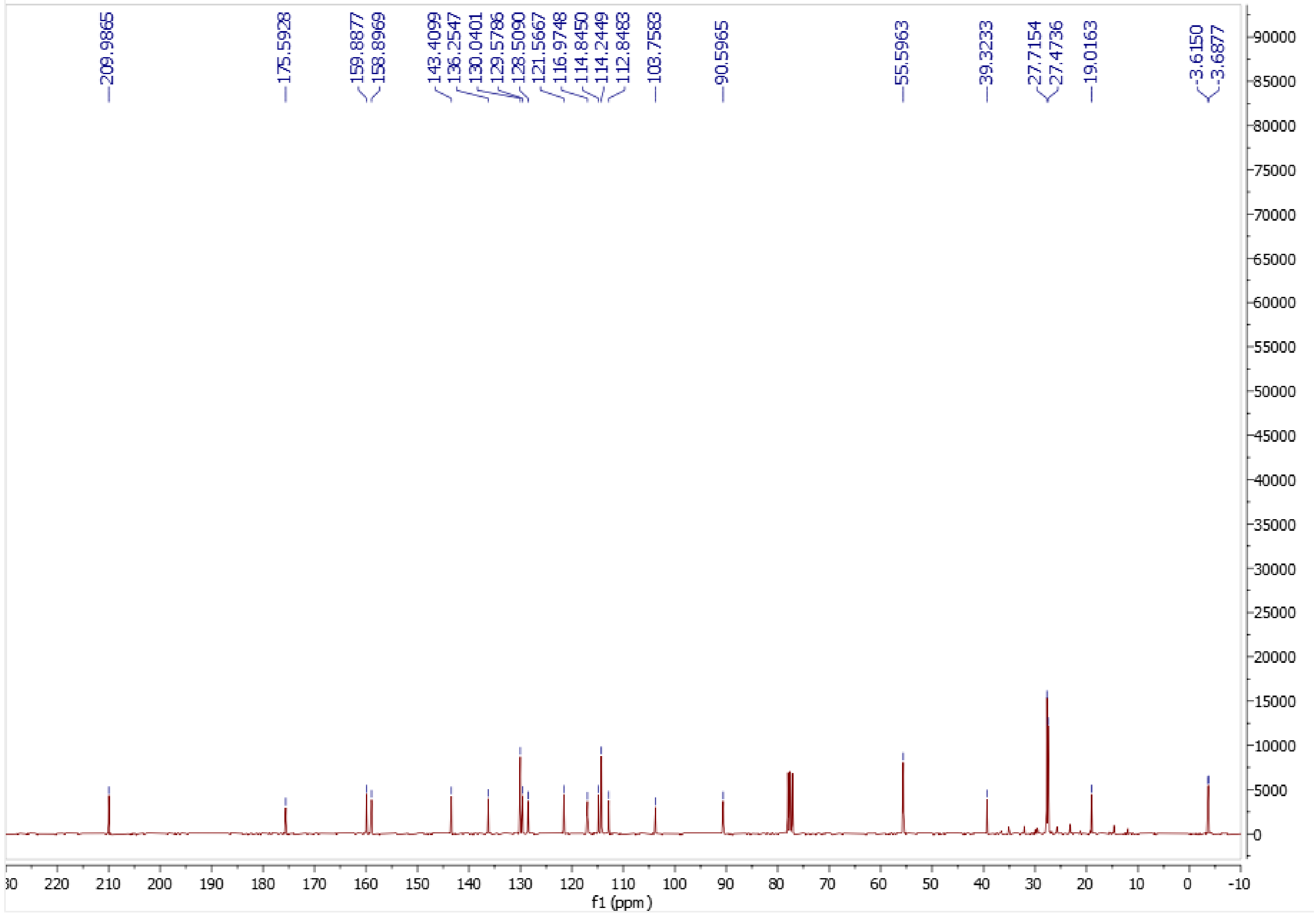

^{1}H NMR spectrum ($CDCl_3$, 300 MHz) of compound **3i**

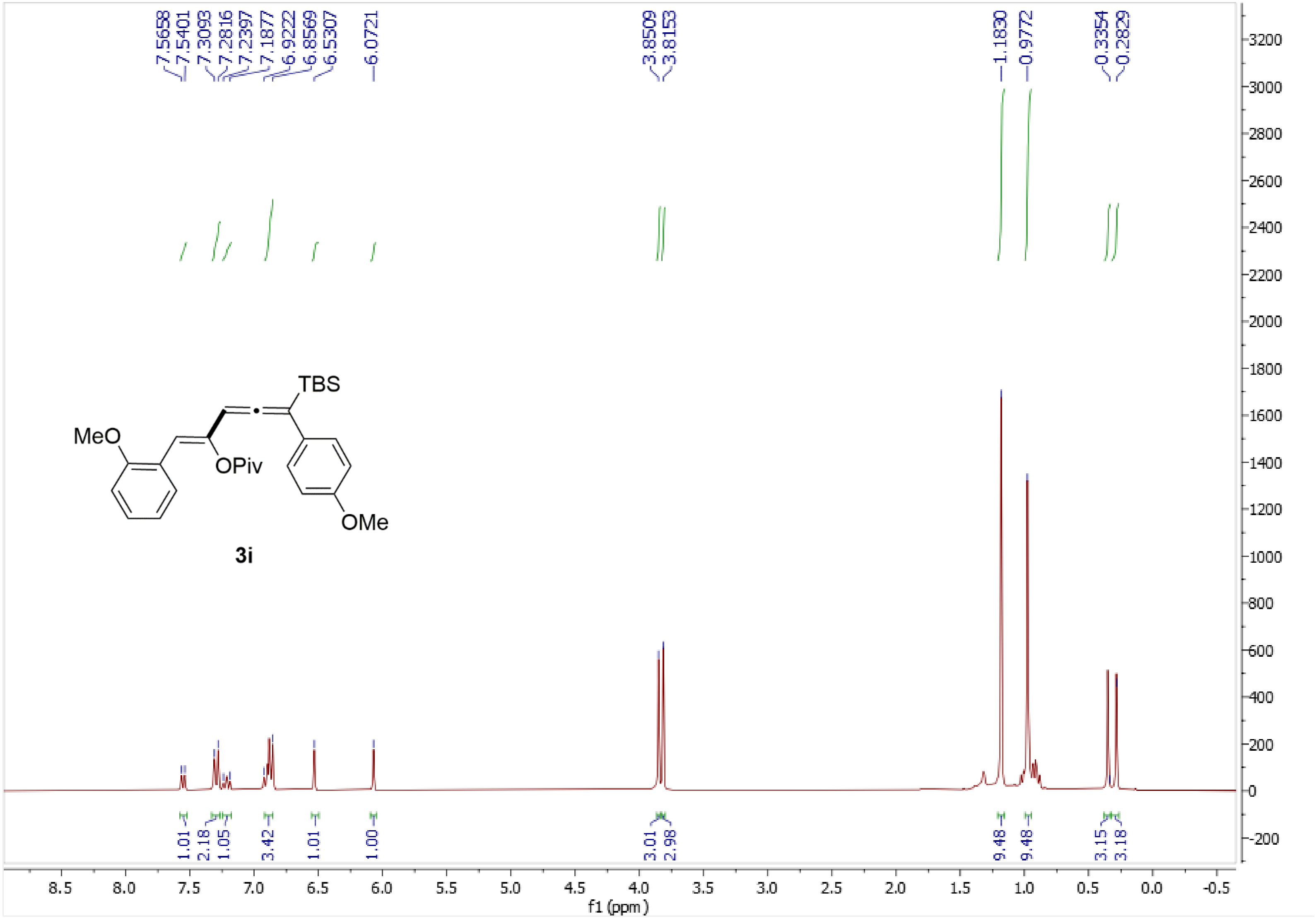

^{13}C NMR spectrum ($CDCl_3$, 75 MHz) of compound **3i**

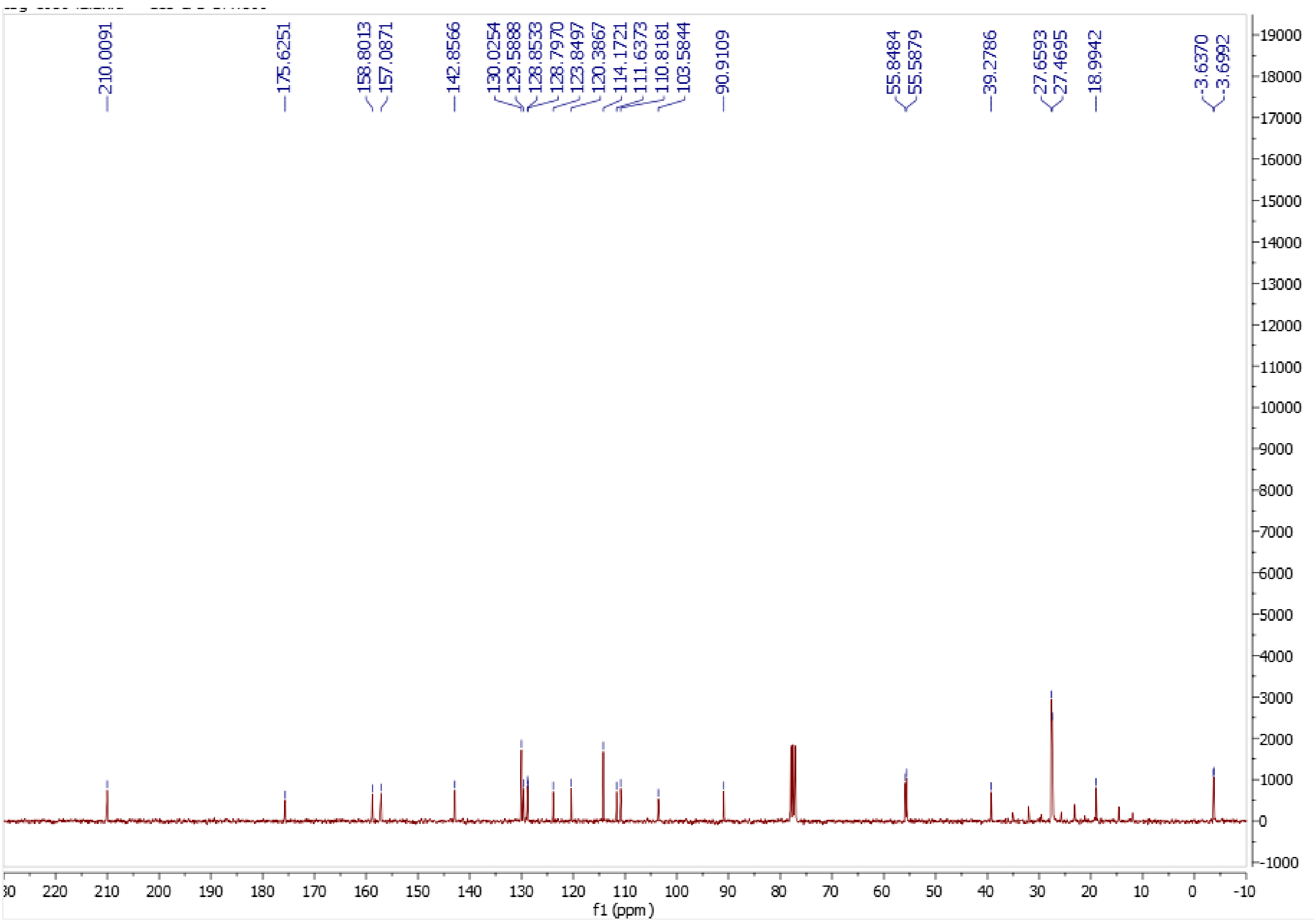

^{1}H NMR spectrum ($CDCl_3$, 300 MHz) of compound **3l**

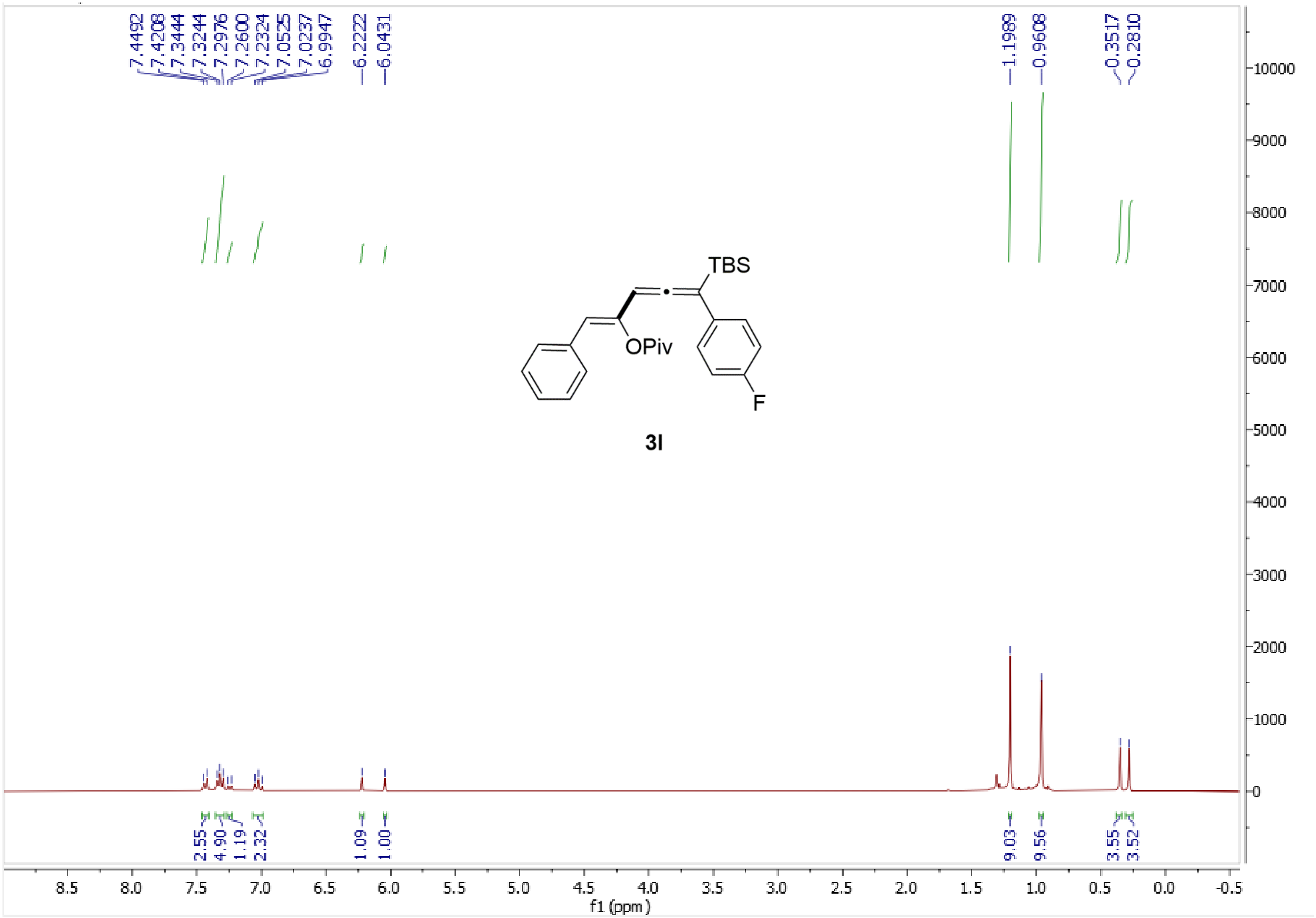

^{13}C NMR spectrum ($CDCl_3$, 75 MHz) of compound **3l**

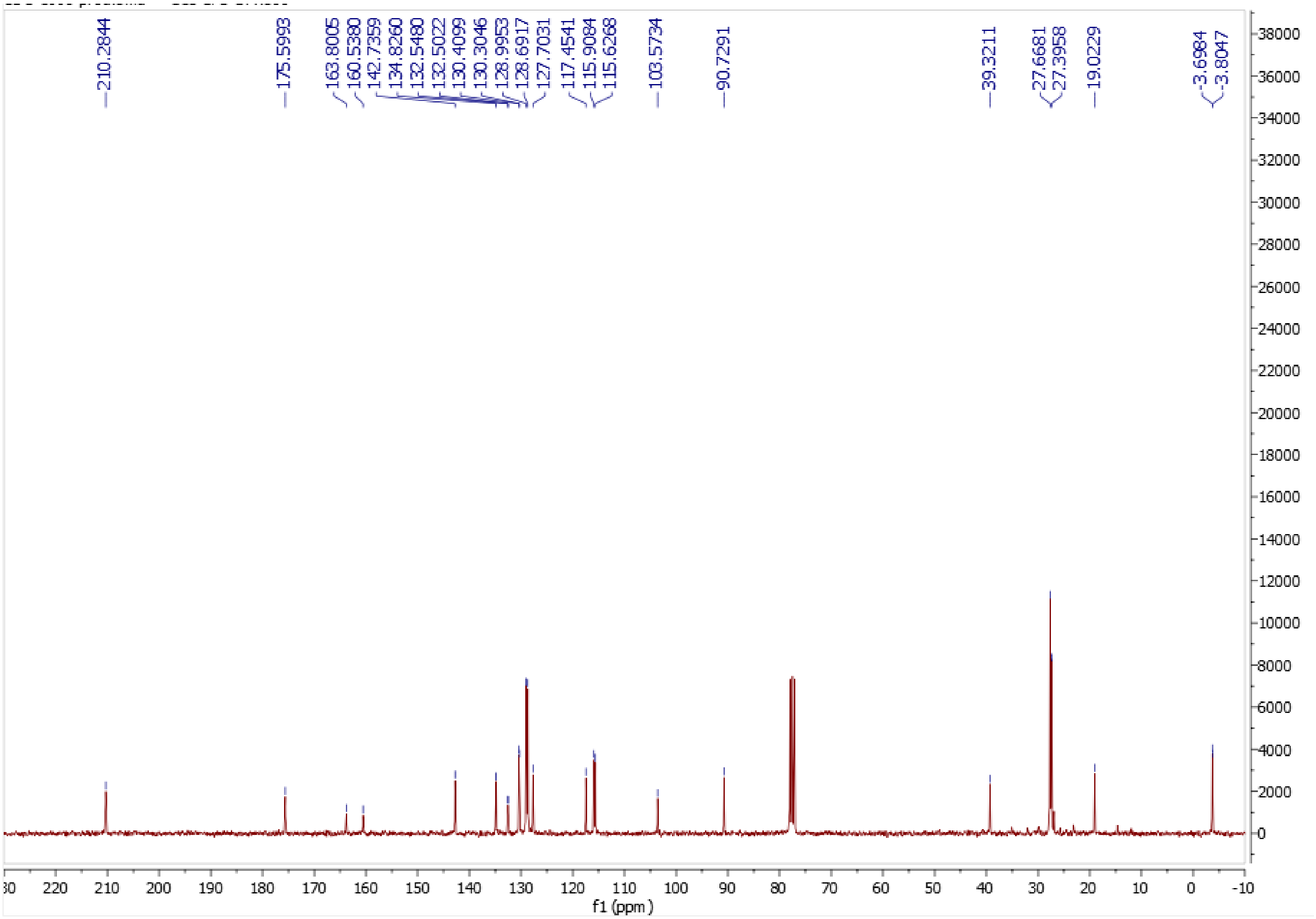

^{19}F NMR spectrum ($CDCl_3$, 282 MHz) of compound **3l**

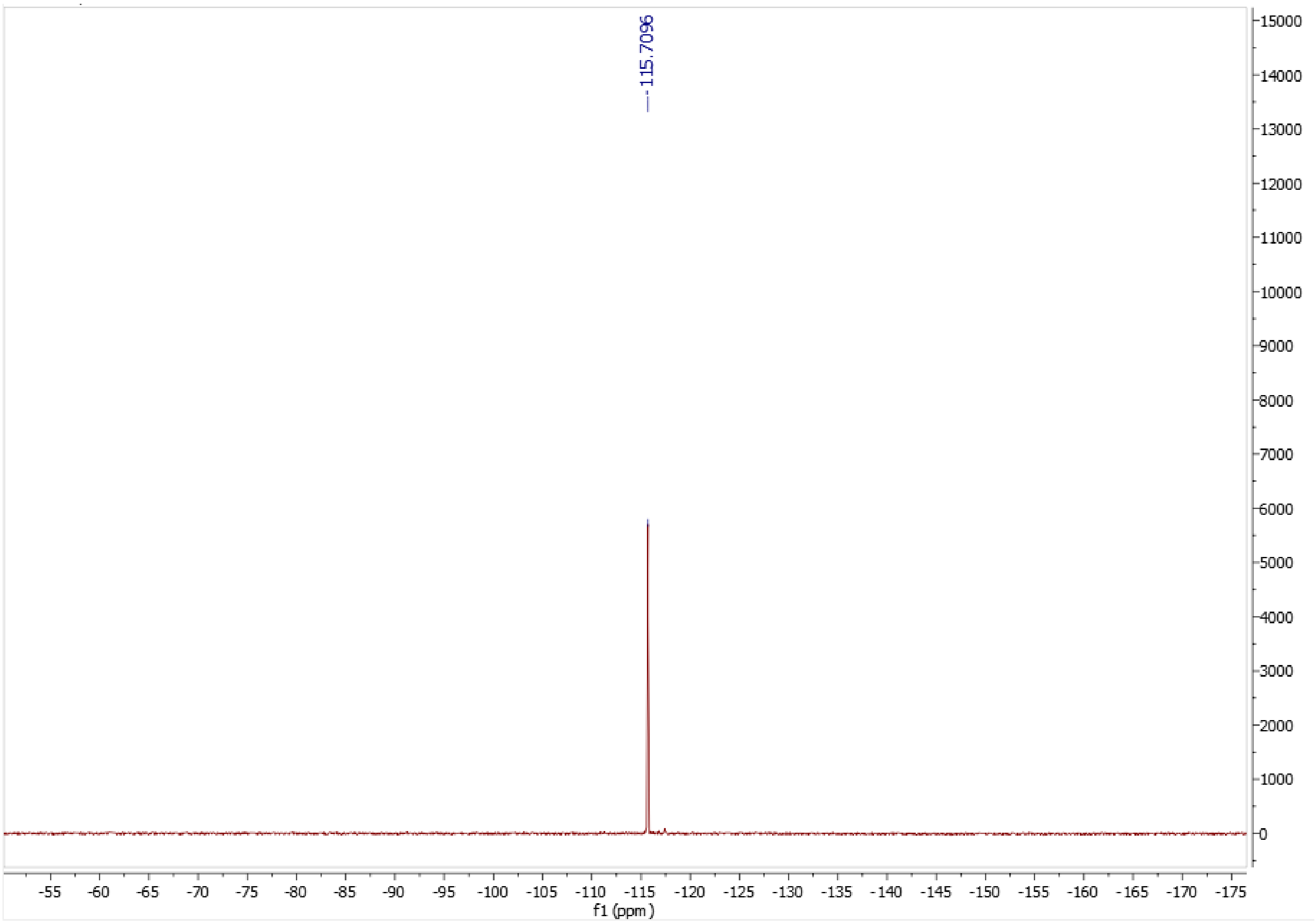

^{1}H NMR spectrum ($CDCl_3$, 300 MHz) of compound **3m**

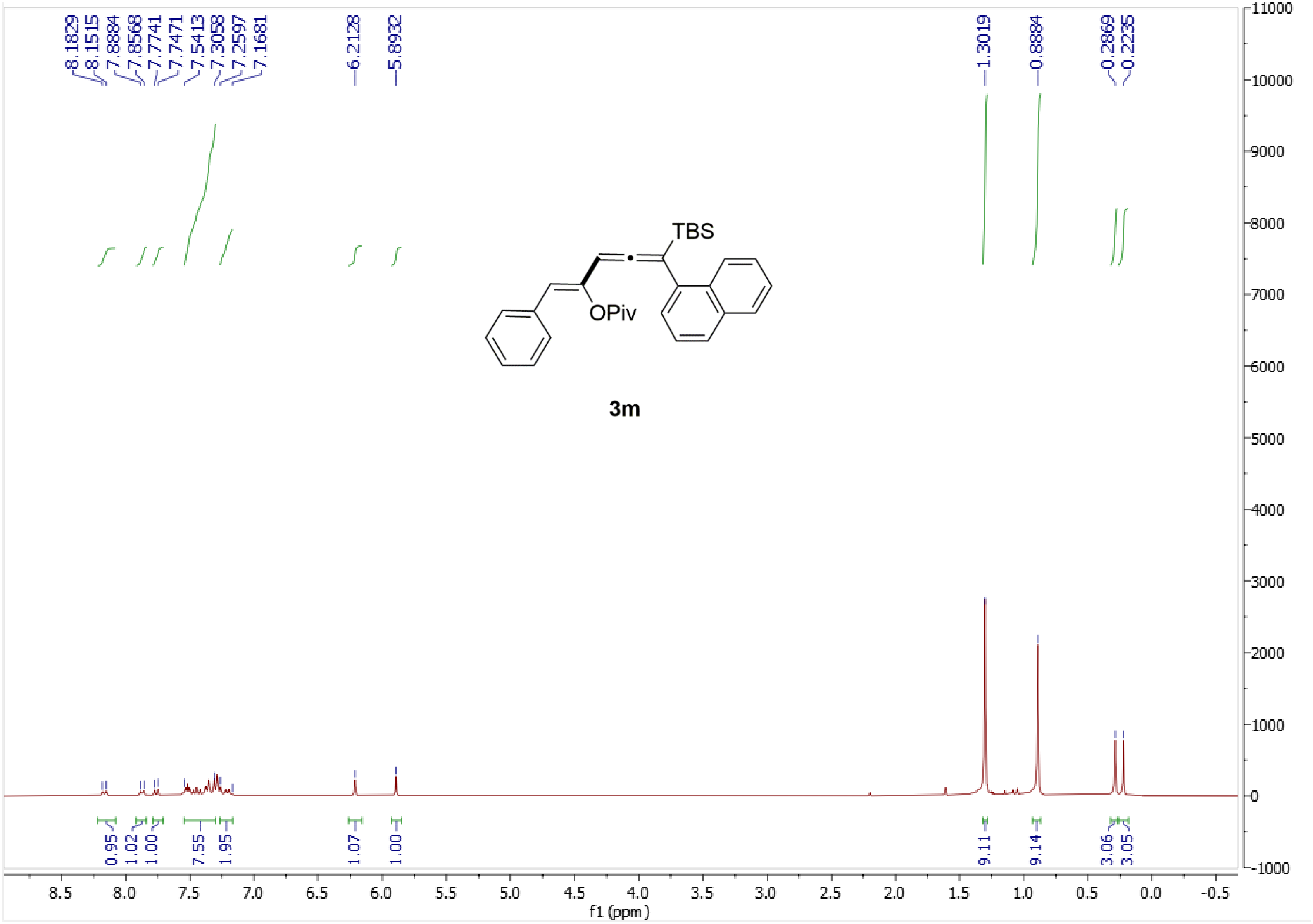

^{13}C NMR spectrum ($CDCl_3$, 75 MHz) of compound **3m**

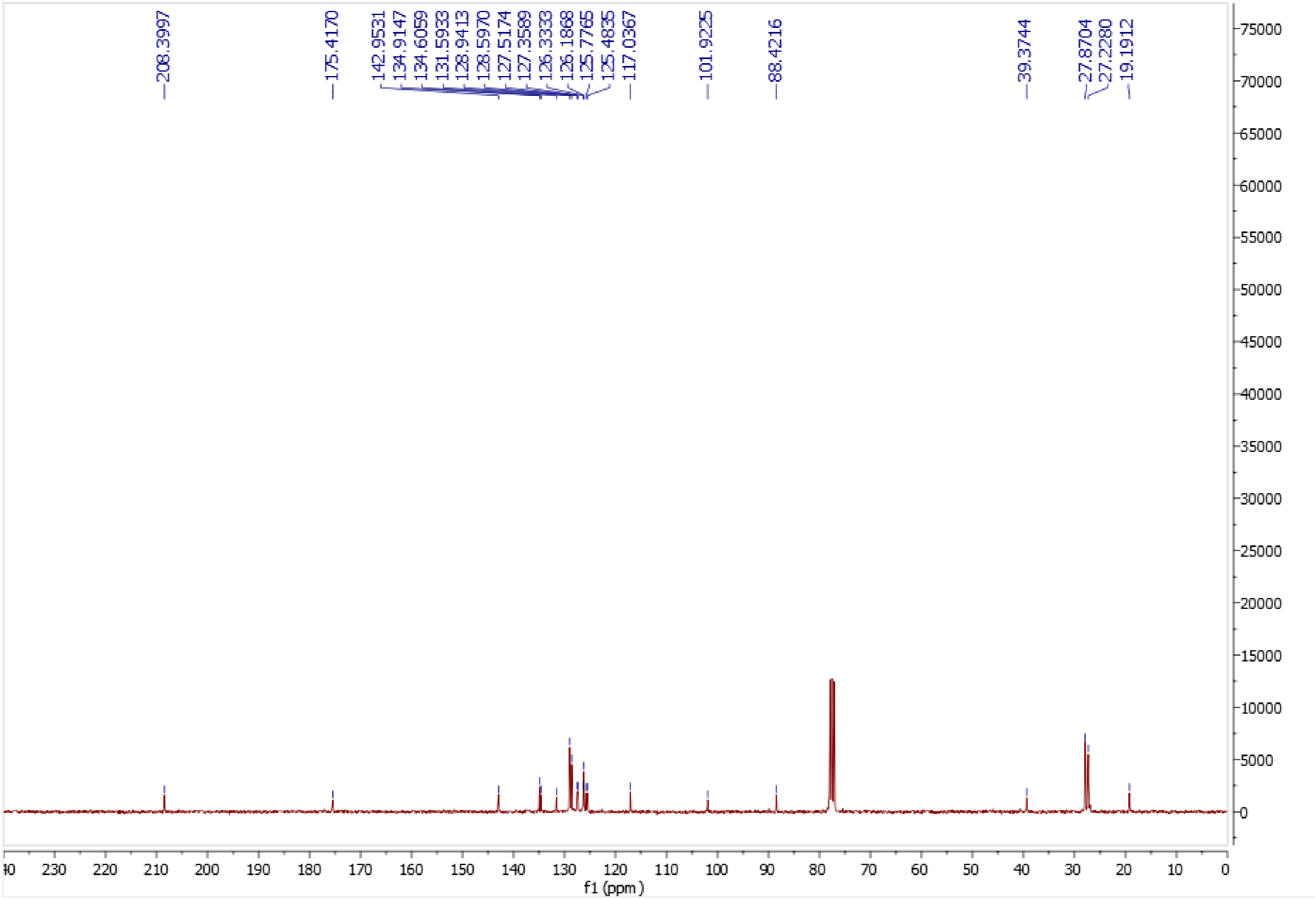

^{1}H NMR spectrum ($CDCl_3$, 300 MHz) of compound **3n**

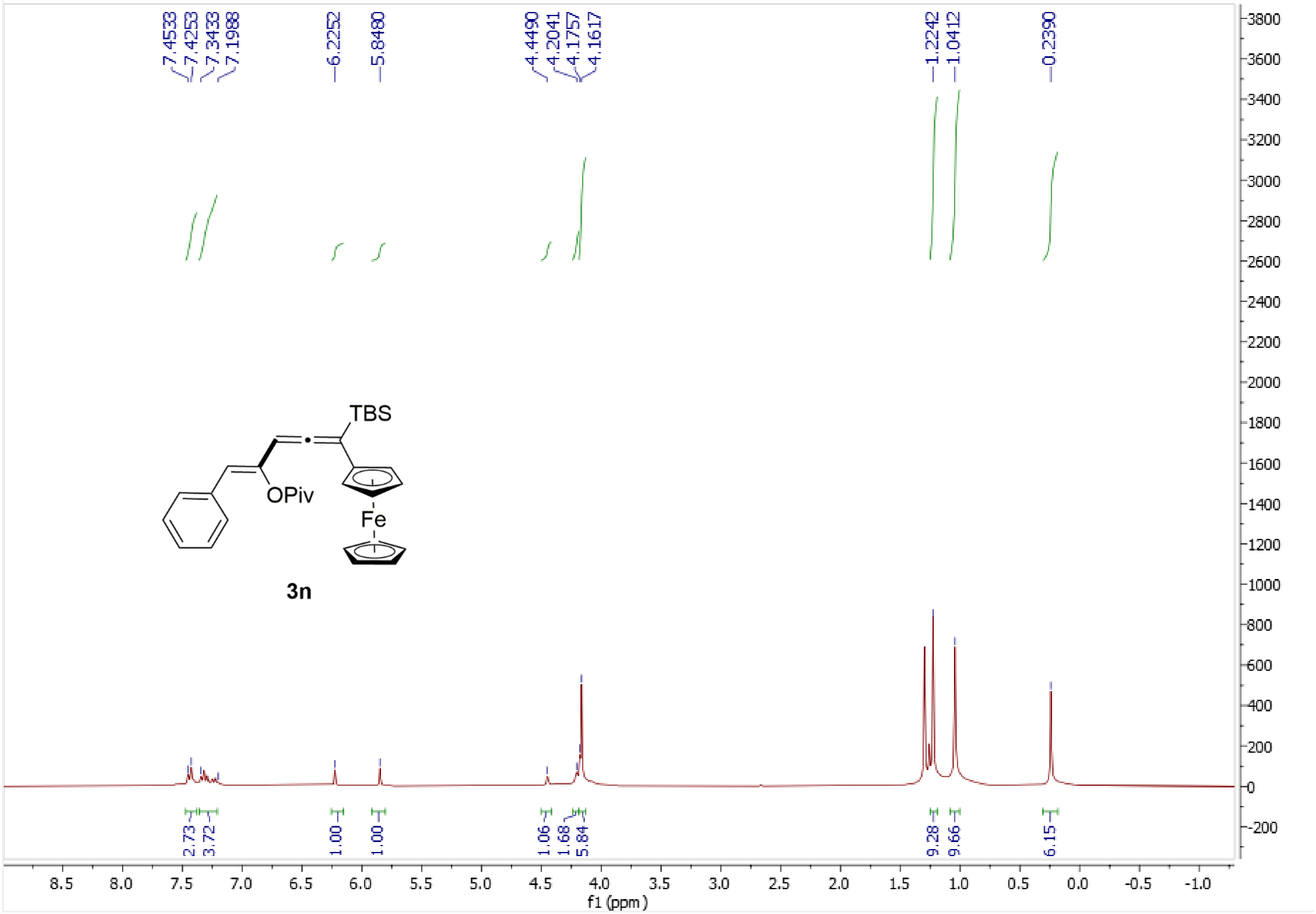

^{13}C NMR spectrum ($CDCl_3$, 75 MHz) of compound **3n**

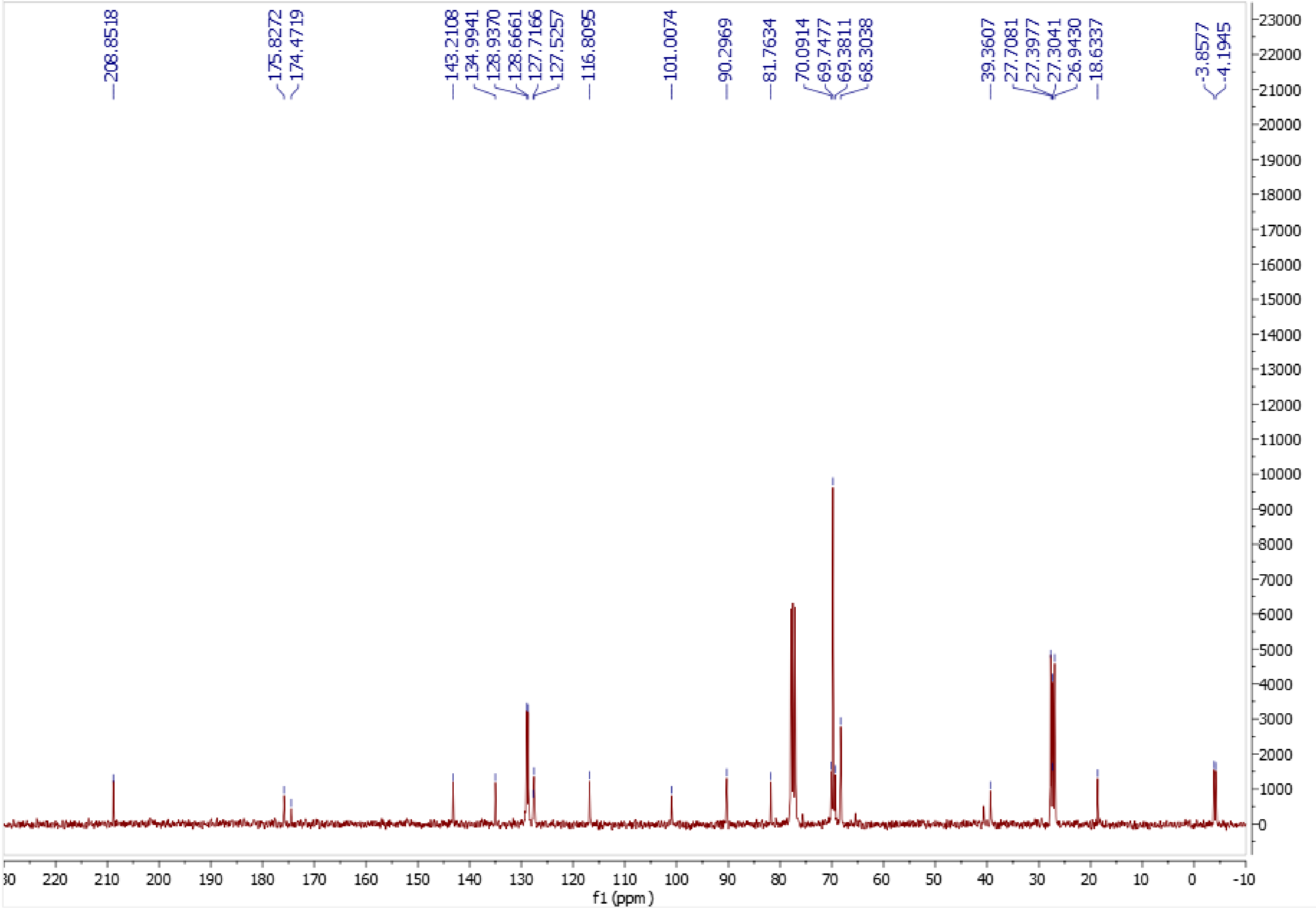

^{1}H NMR spectrum ($CDCl_3$, 300 MHz) of compound **4a** (obtained by Method A)

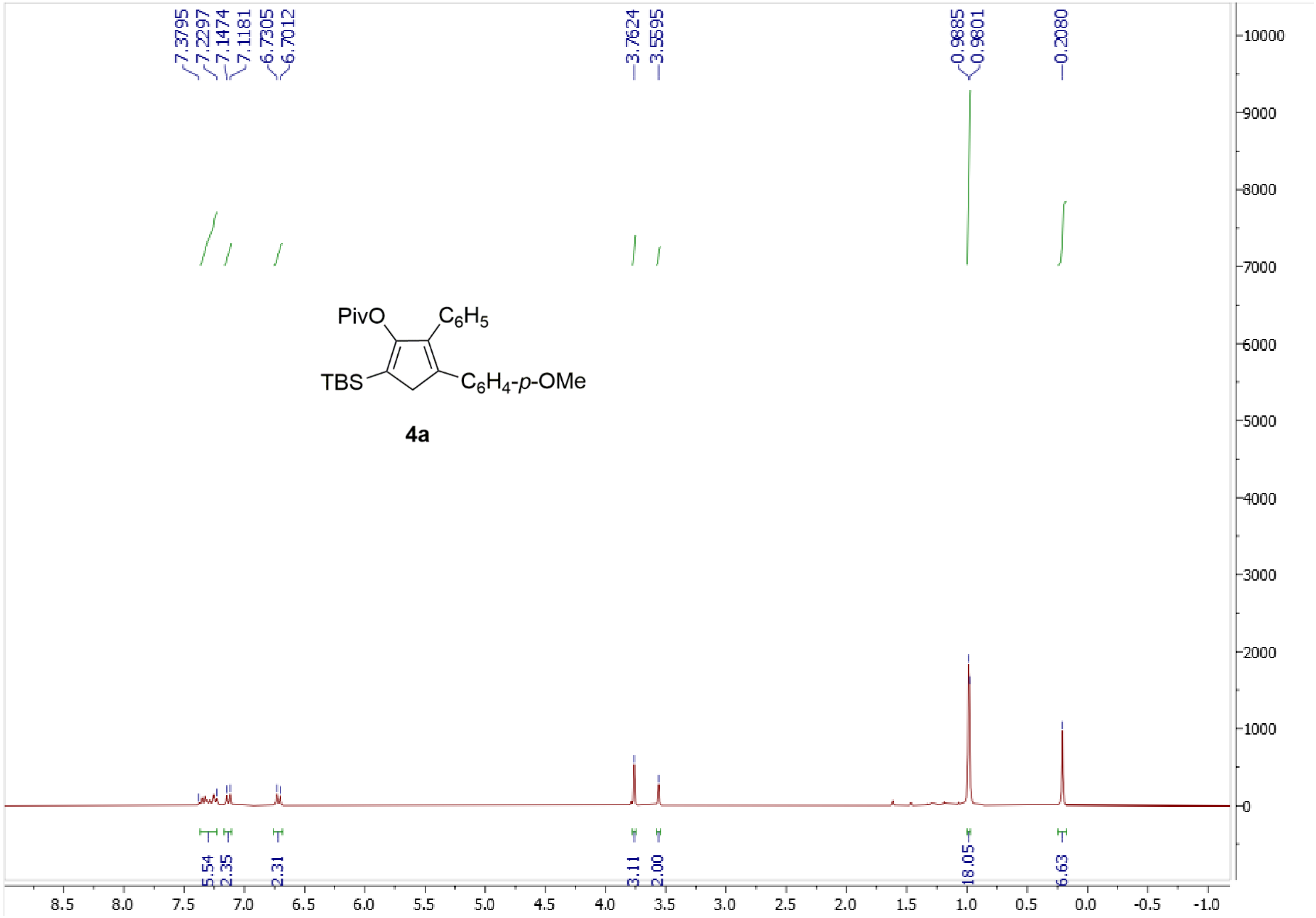

^{13}C NMR spectrum ($CDCl_3$, 75 MHz) of compound **4a** (obtained by Method A)

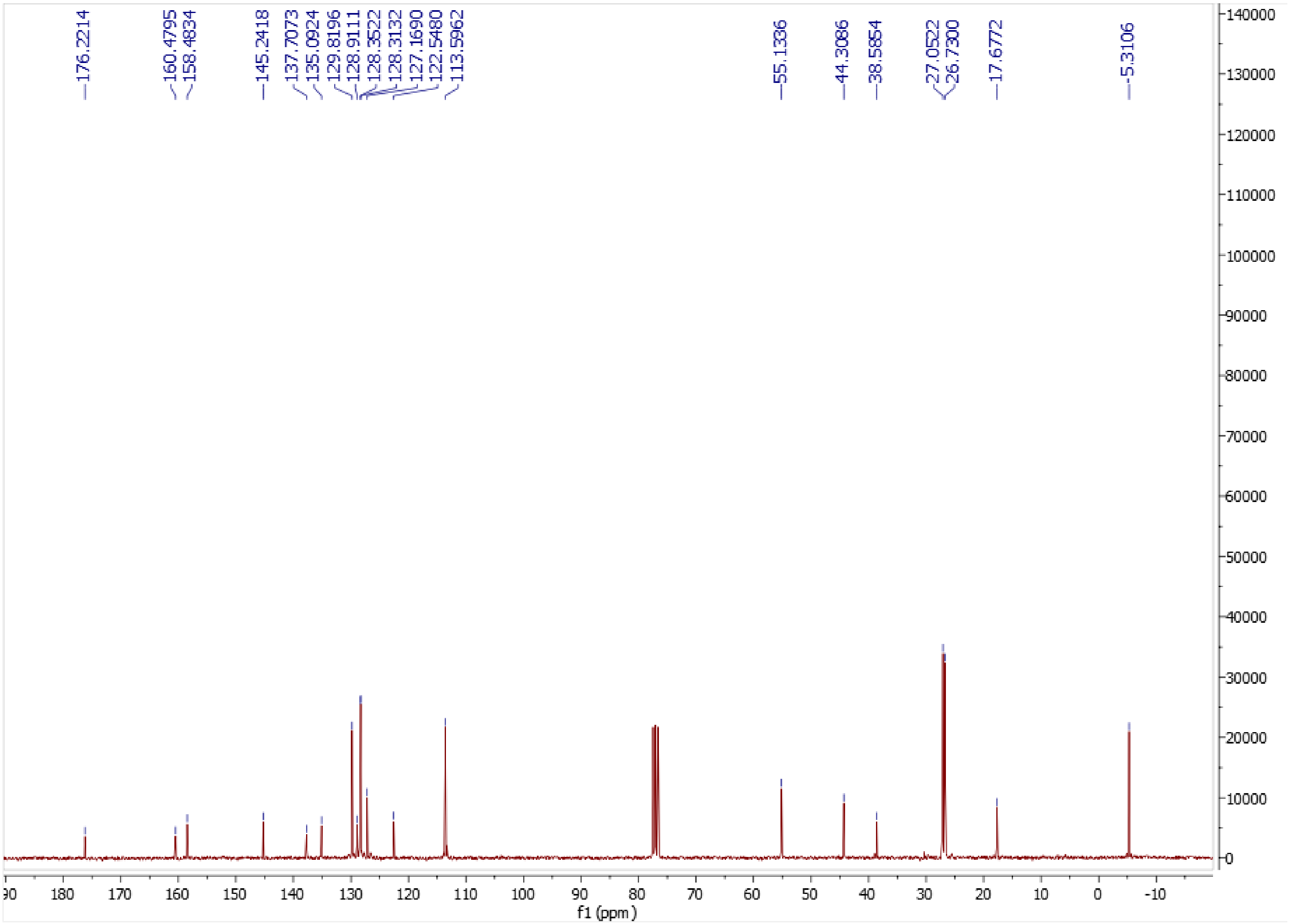

^{1}H NMR spectrum ($CDCl_3$, 300 MHz) of compound **4a** (obtained by Method B)

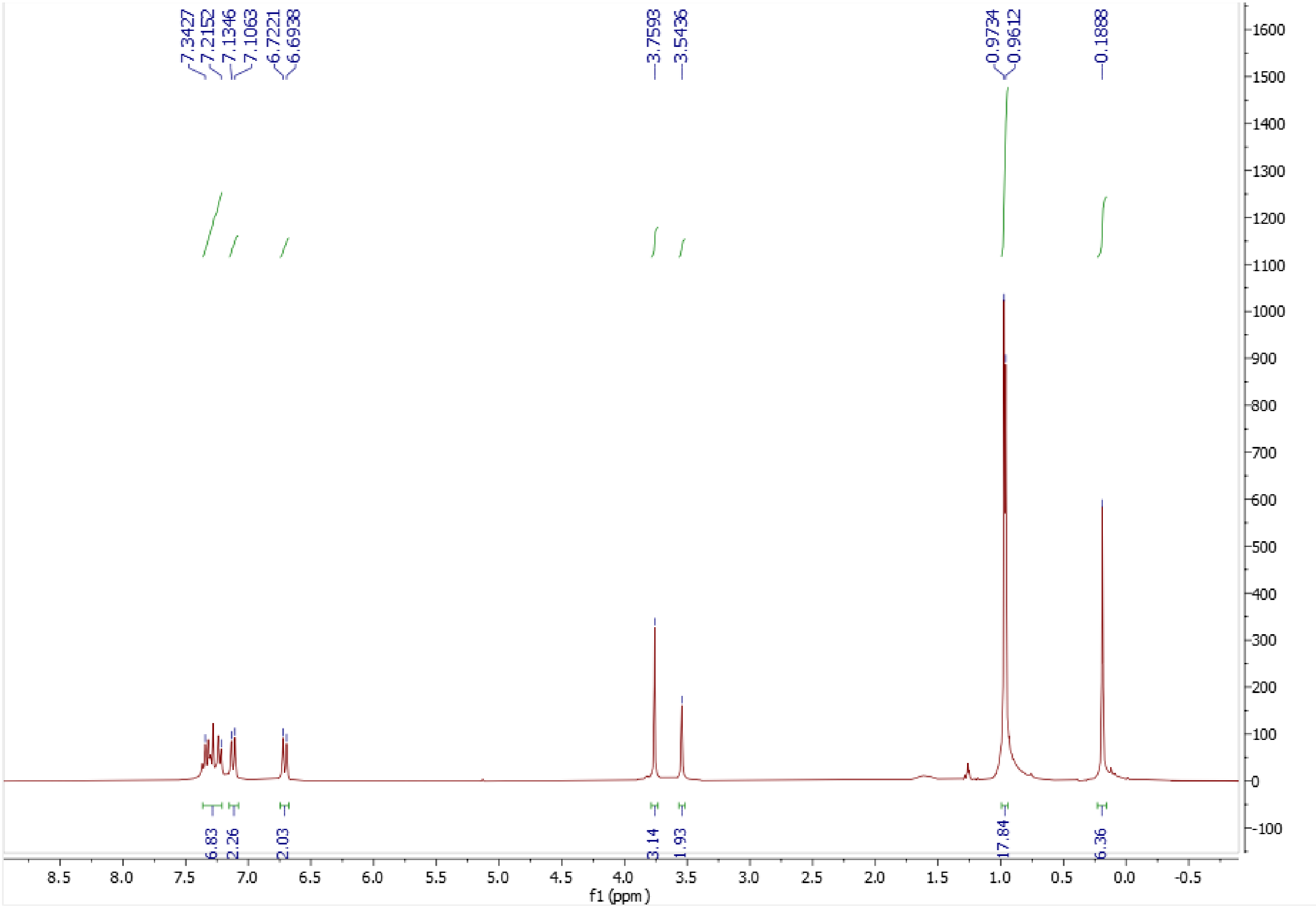

^{1}H NMR spectrum ($CDCl_3$, 300 MHz) of compound **4b** (obtained by Method A)

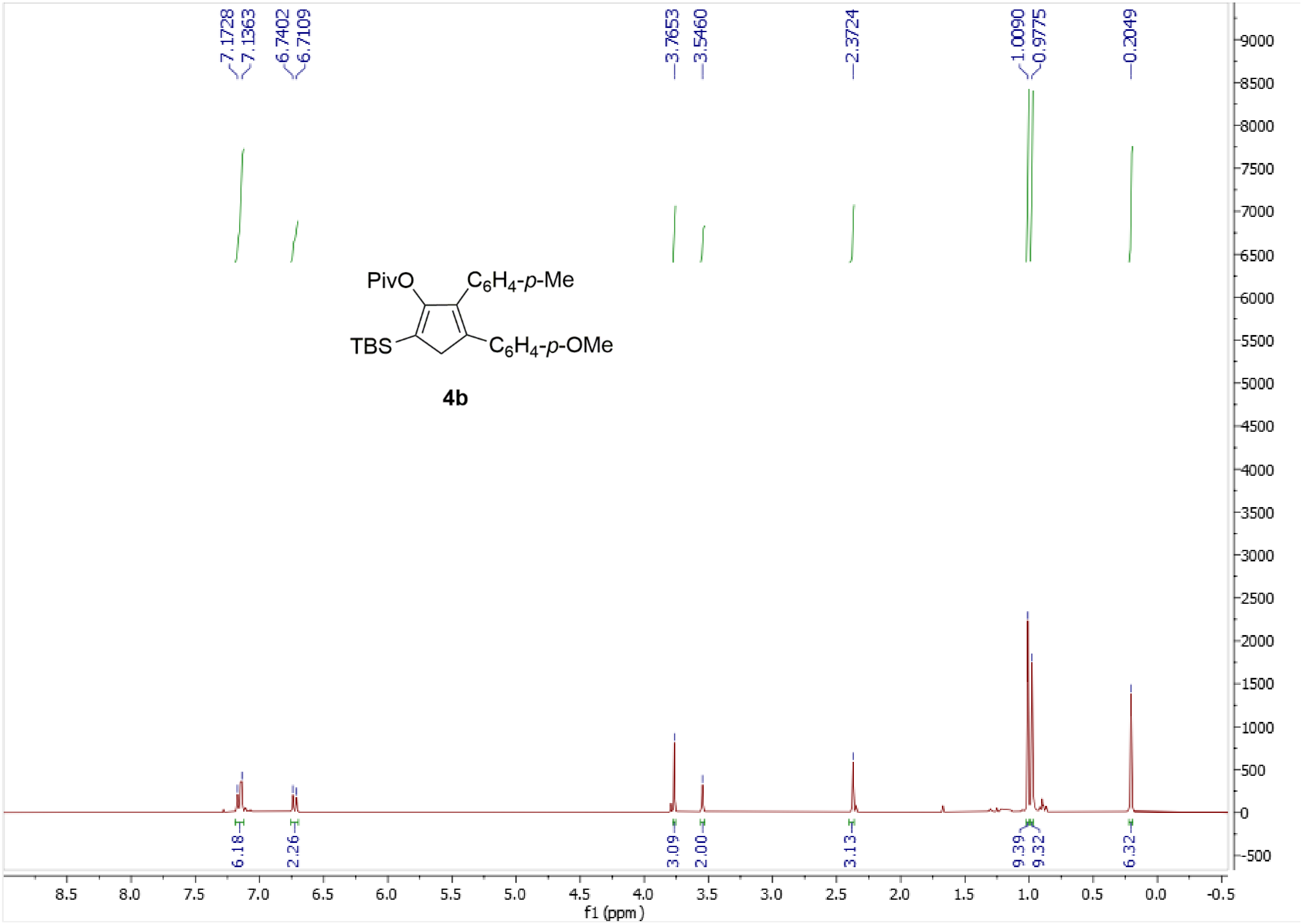

^{13}C NMR spectrum ($CDCl_3$, 75 MHz) of compound **4b** (obtained by Method A)

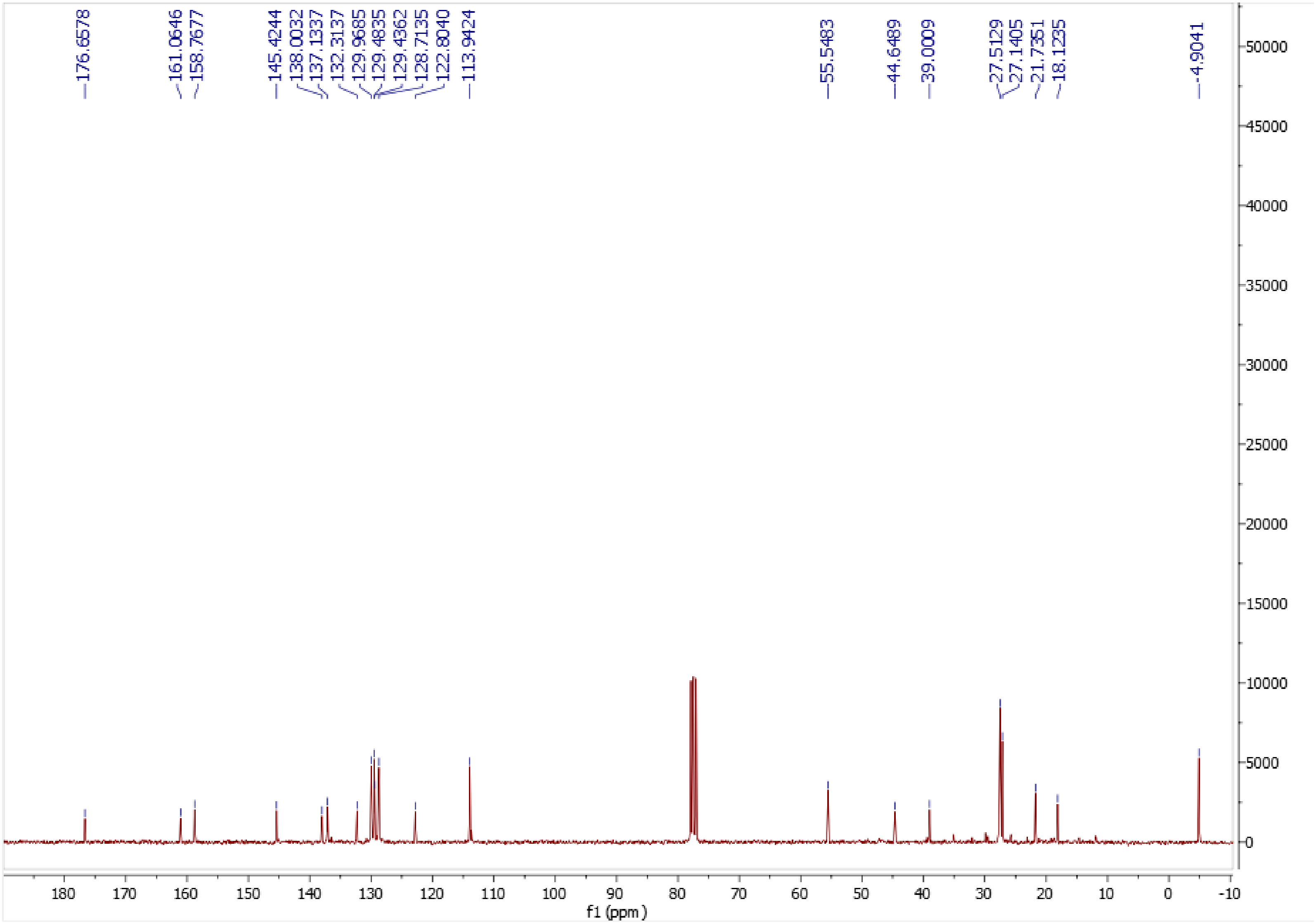

^{1}H NMR spectrum ($CDCl_3$, 300 MHz) of compound **4b** (obtained by Method B)

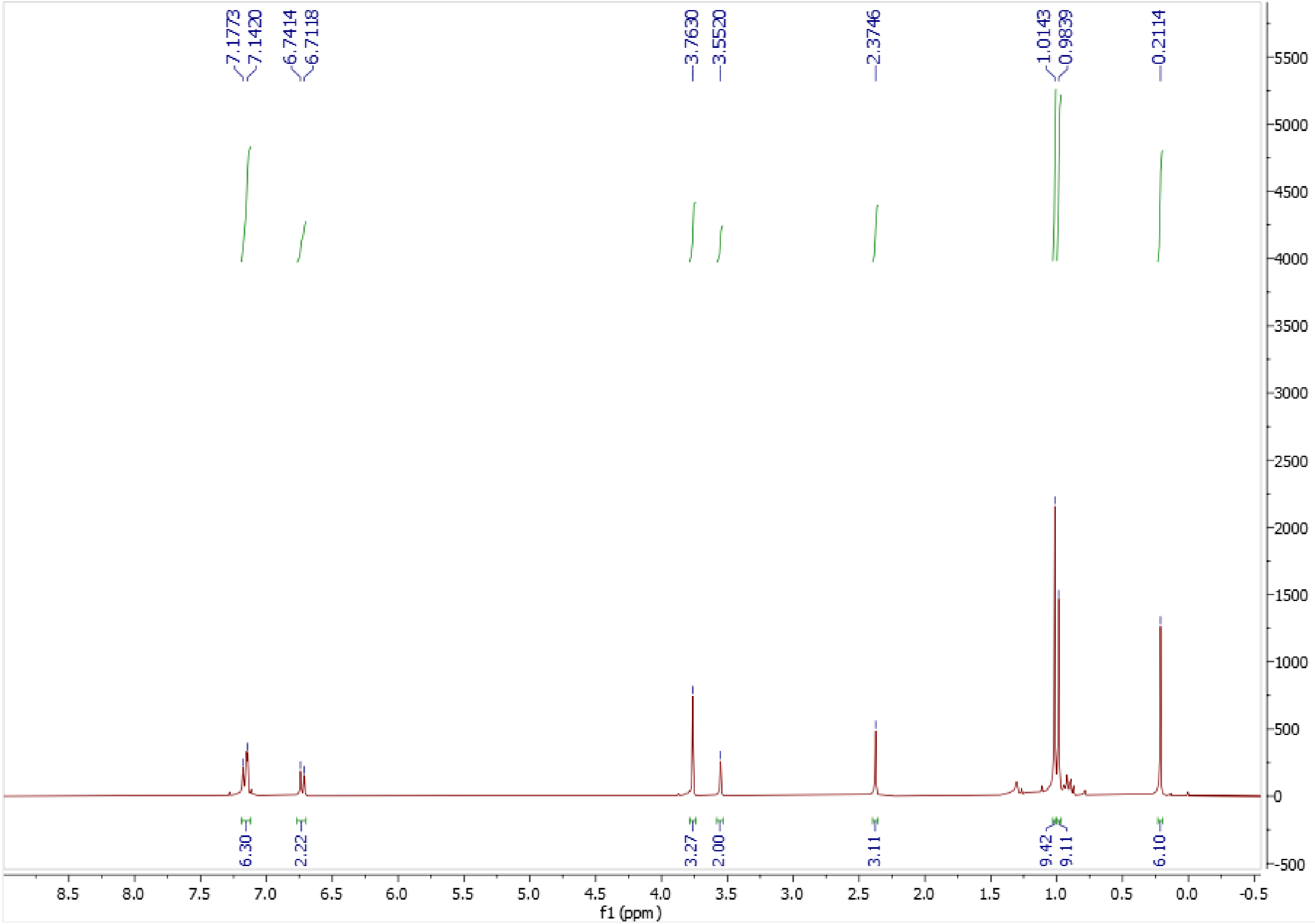

^{1}H NMR spectrum ($CDCl_3$, 300 MHz) of compound **4c** (obtained by Method A)

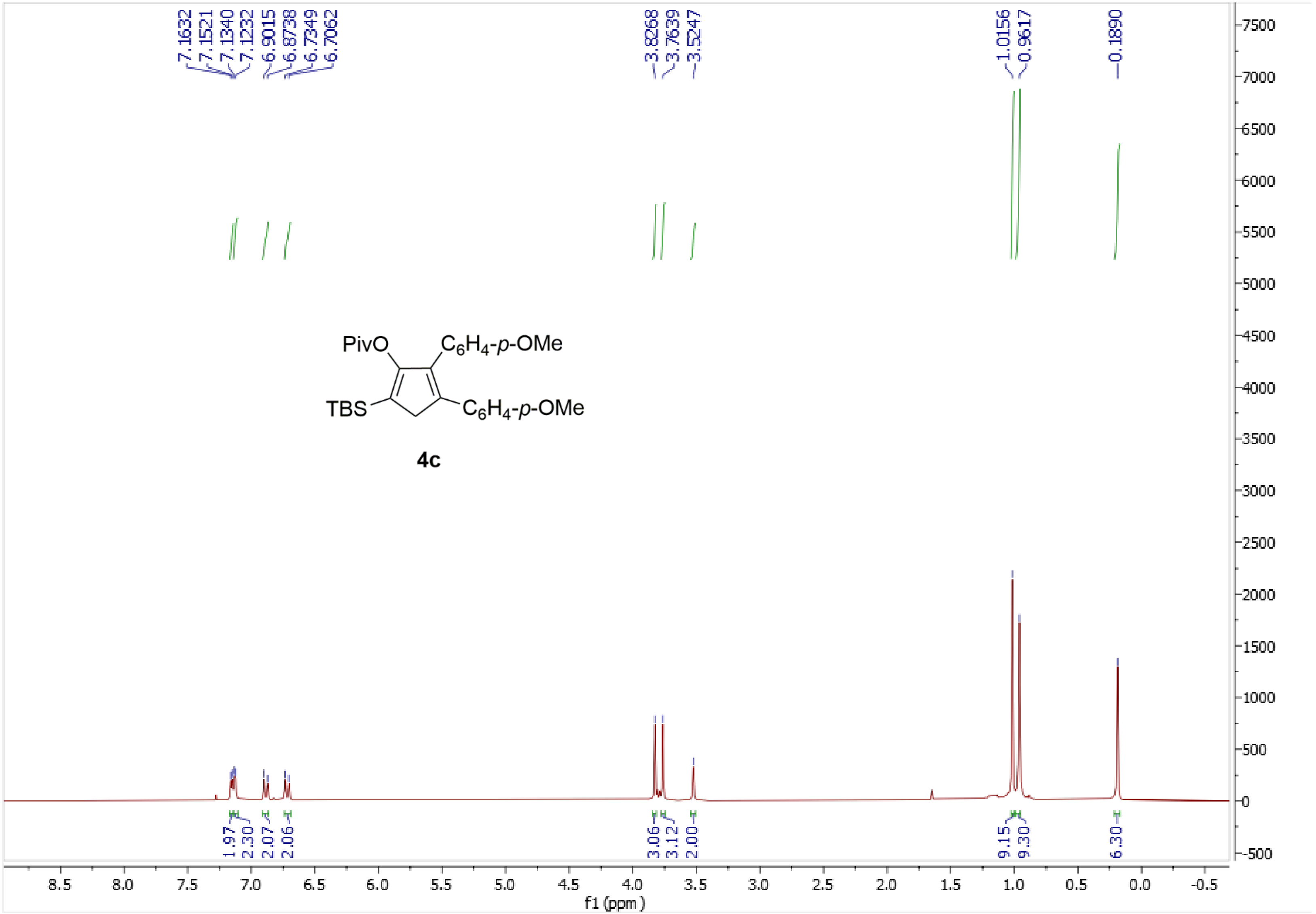

^{13}C NMR spectrum ($CDCl_3$, 75 MHz) of compound **4c** (obtained by Method A)

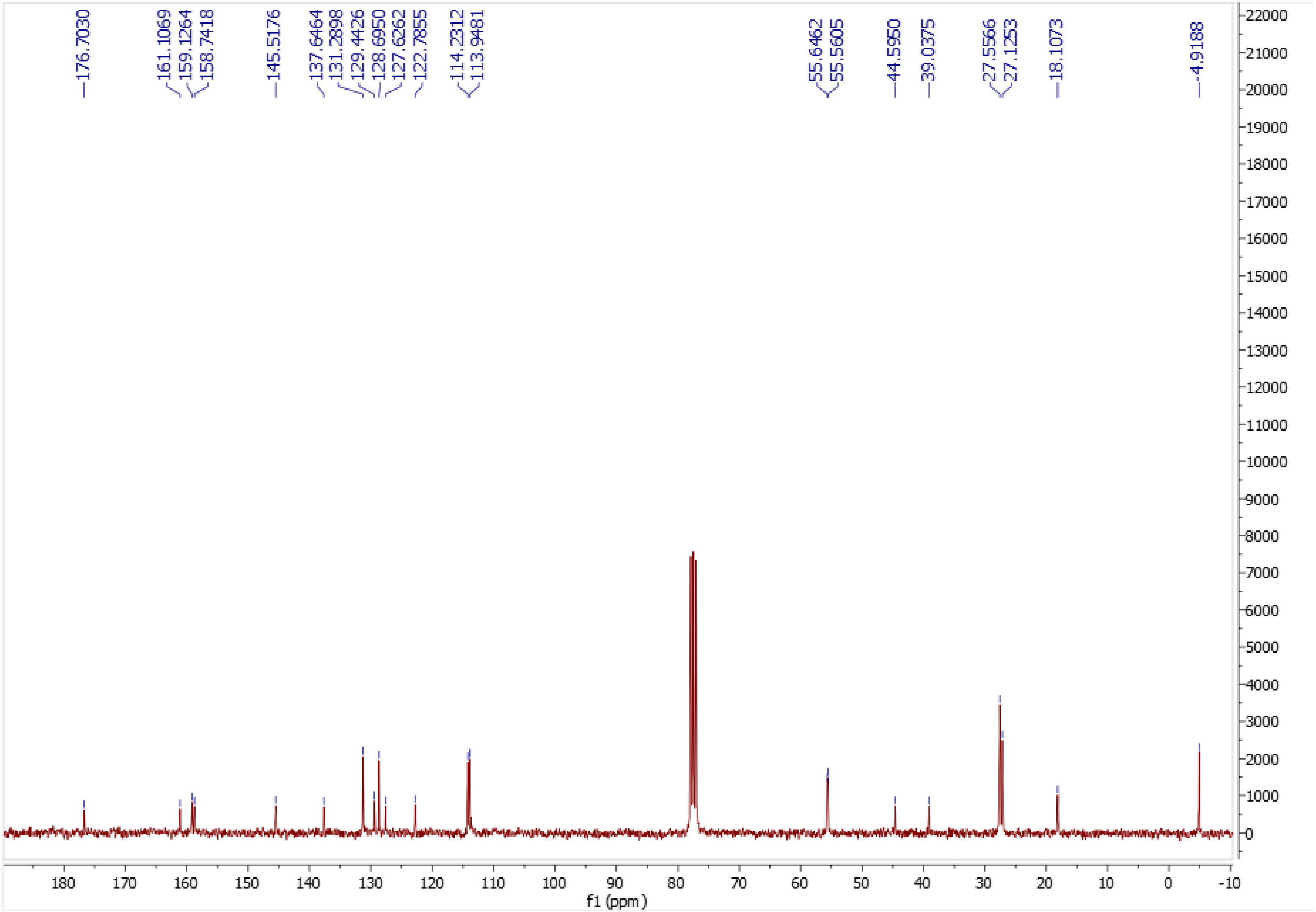

^{1}H NMR spectrum ($CDCl_3$, 300 MHz) of compound **4c** (obtained by Method B)

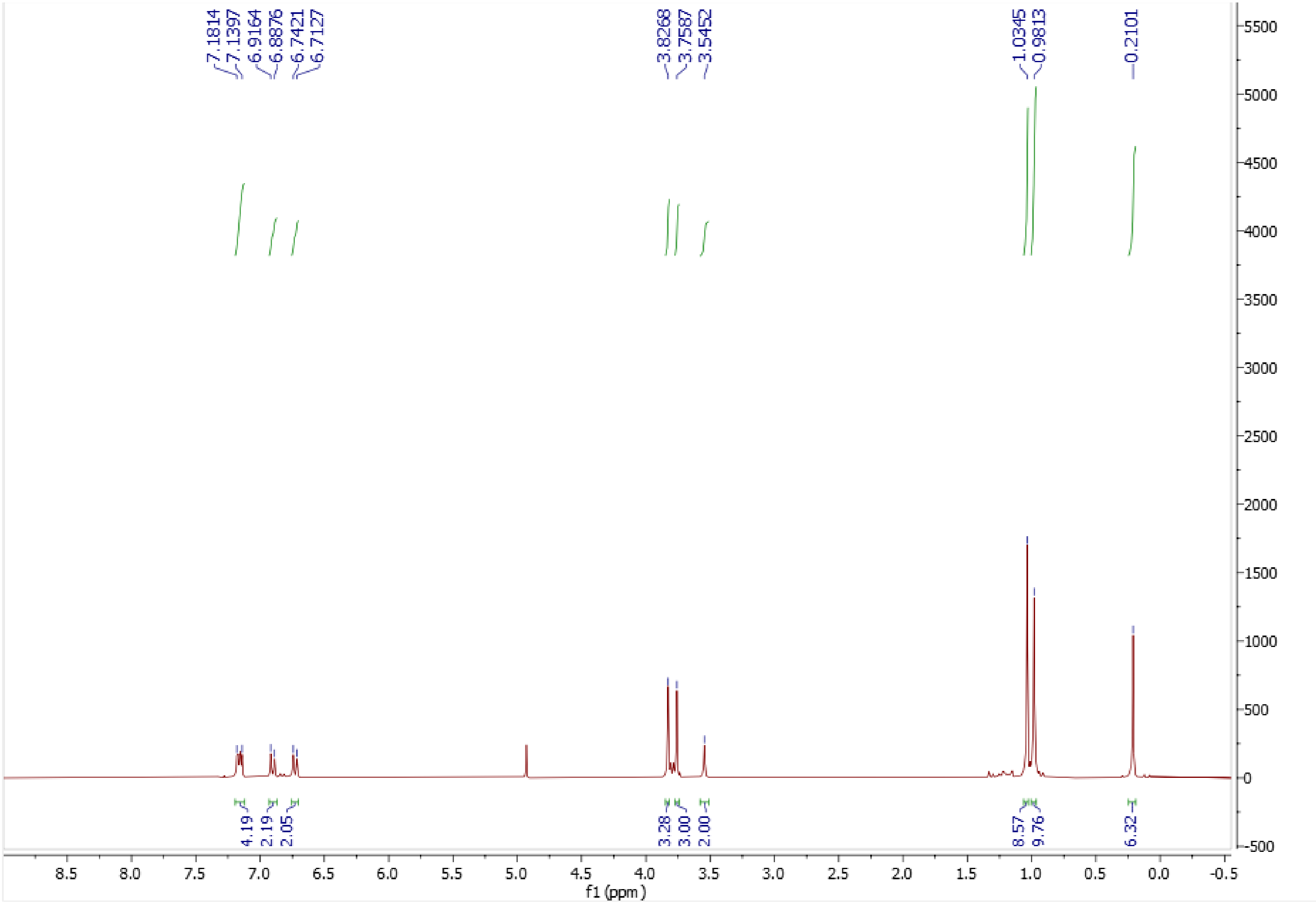

^{1}H NMR spectrum ($CDCl_3$, 300 MHz) of compound **4d** (obtained by Method A)

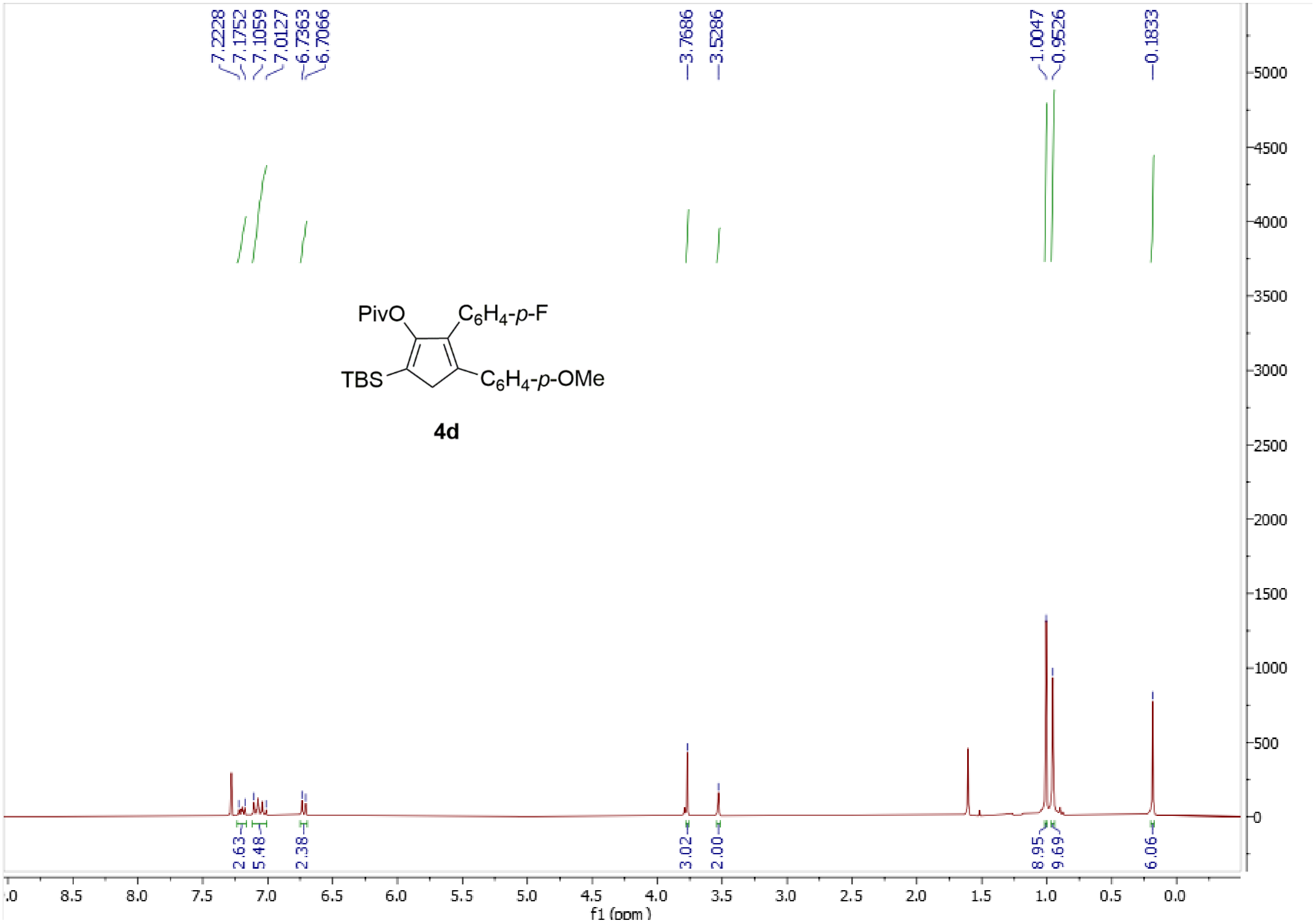

^{13}C NMR spectrum ($CDCl_3$, 75 MHz) of compound **4d** (obtained by Method A)

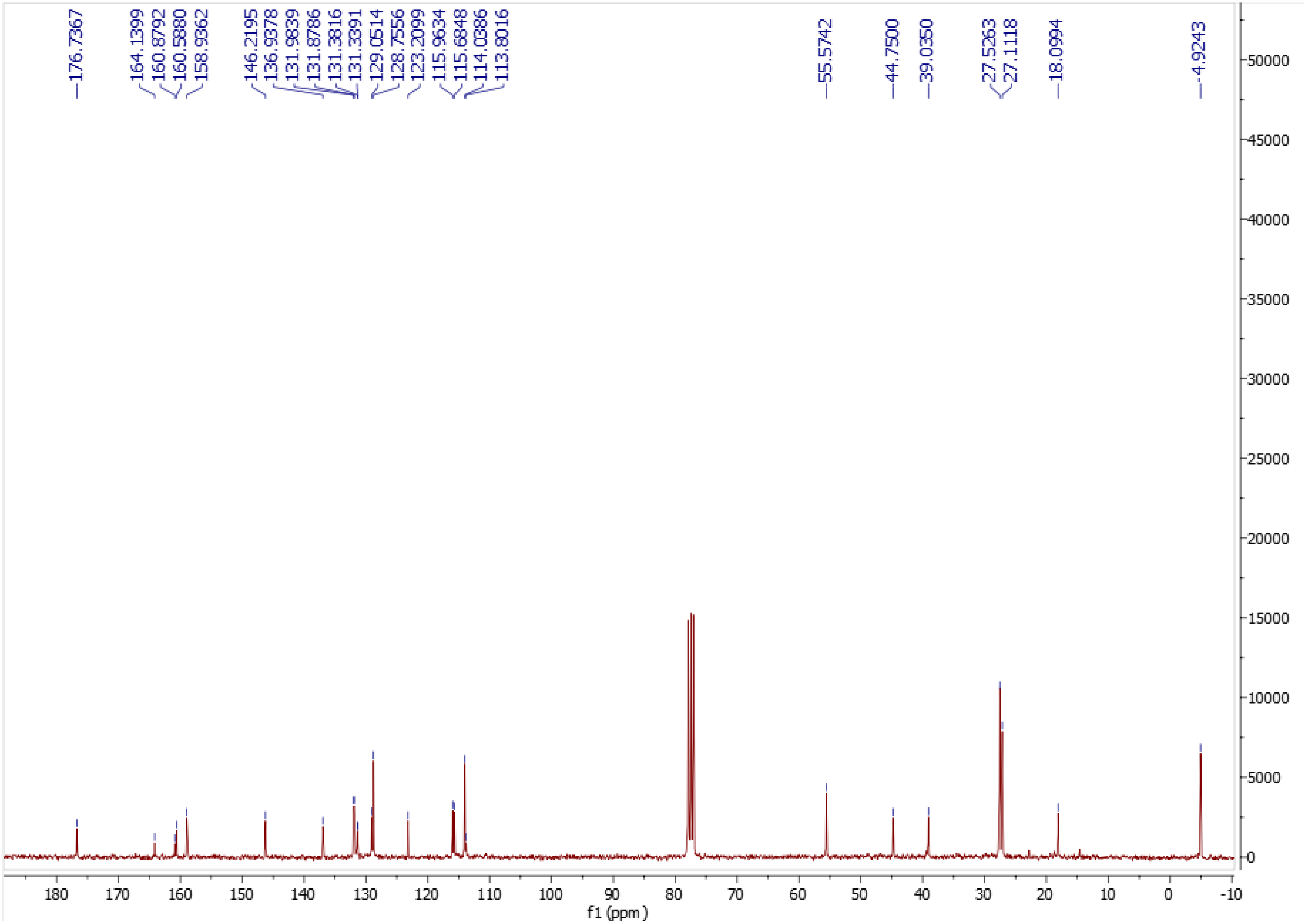

^{19}F NMR spectrum ($CDCl_3$, 282 MHz) of compound **4d** (obtained by Method A)

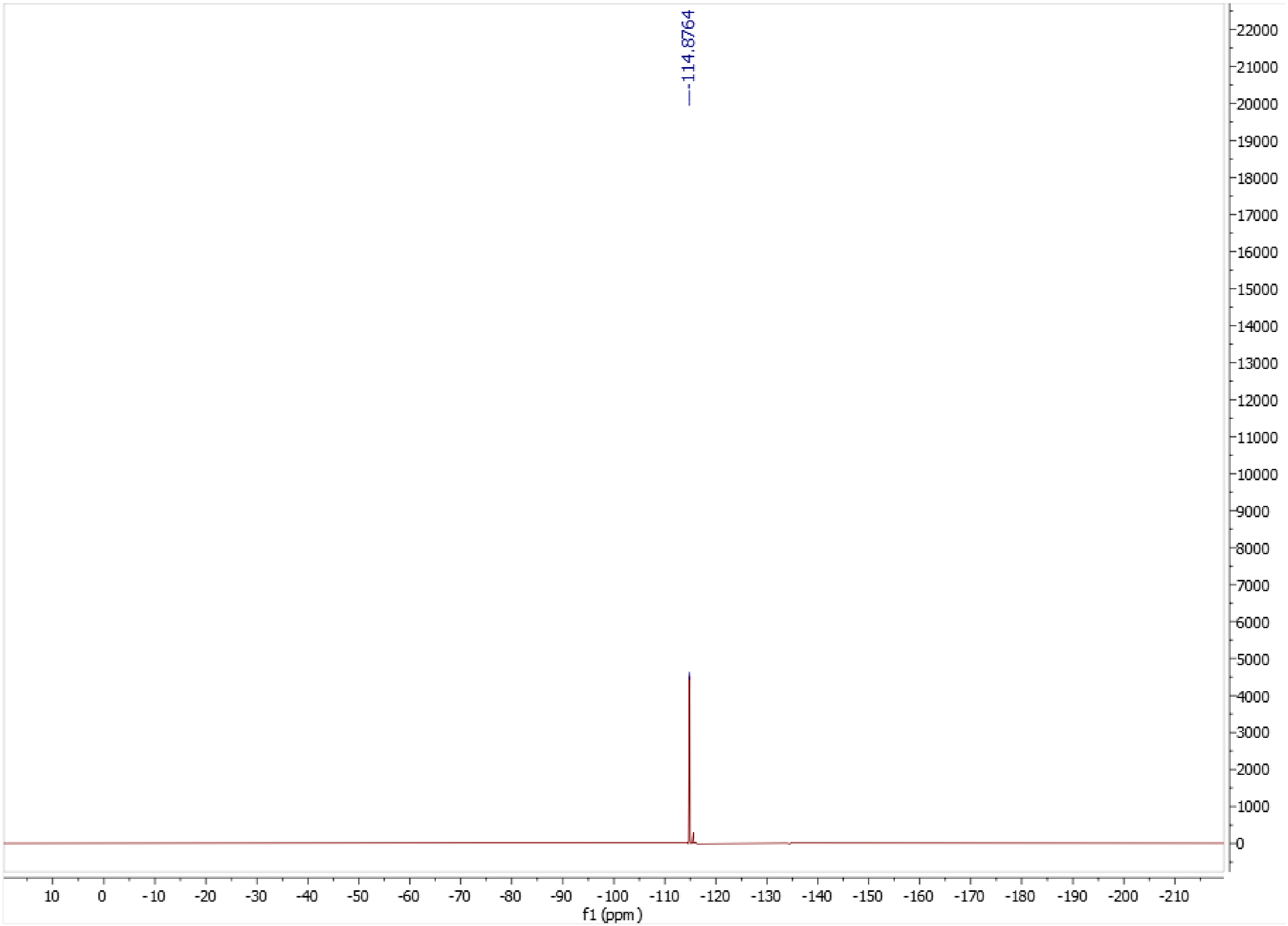

^{1}H NMR spectrum ($CDCl_3$, 300 MHz) of compound **4d** (obtained by Method B)

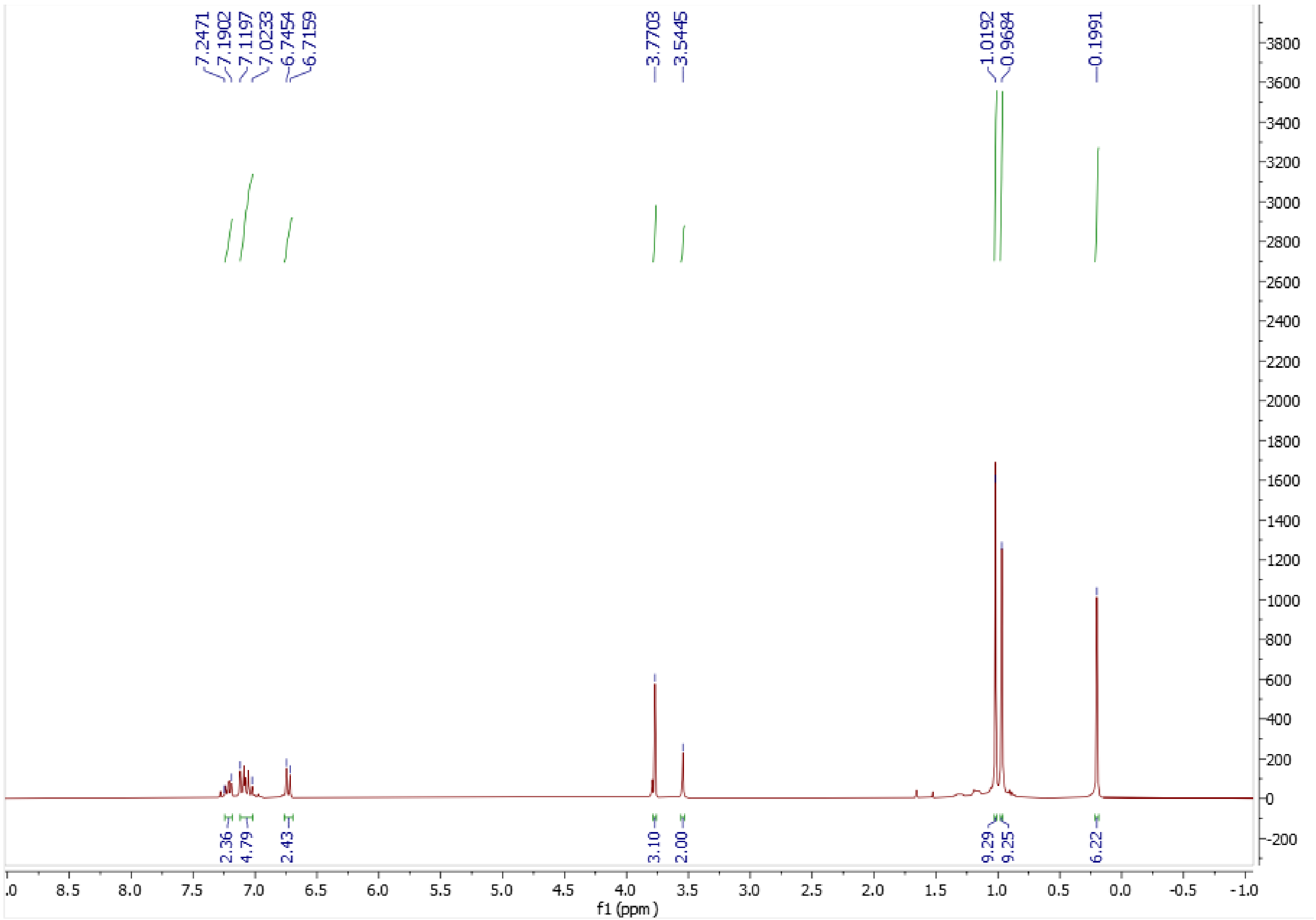

^{1}H NMR spectrum ($CDCl_3$, 300 MHz) of compound **4e** (obtained by Method A)

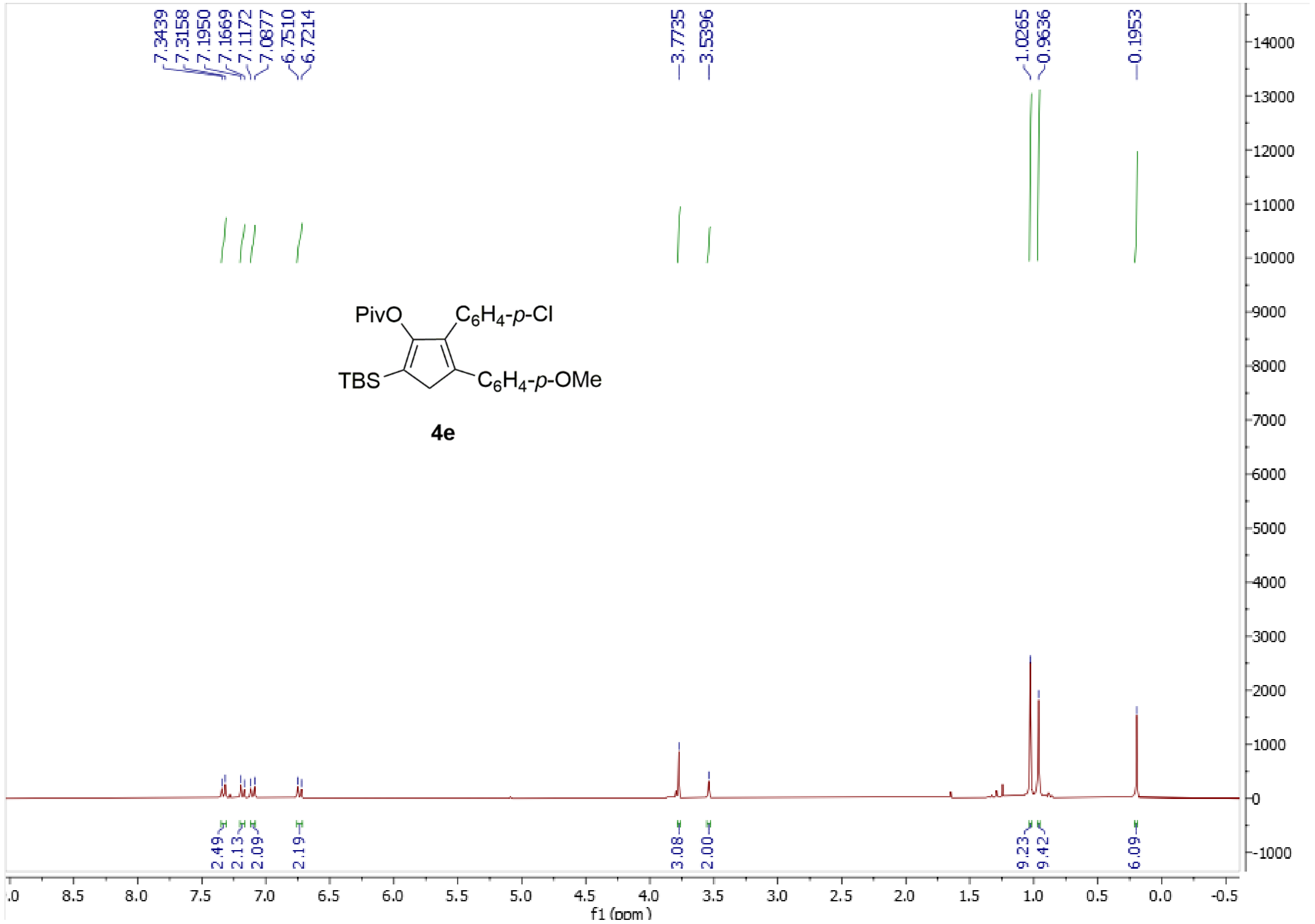

^{13}C NMR spectrum ($CDCl_3$, 75 MHz) of compound **4e** (obtained by Method A)

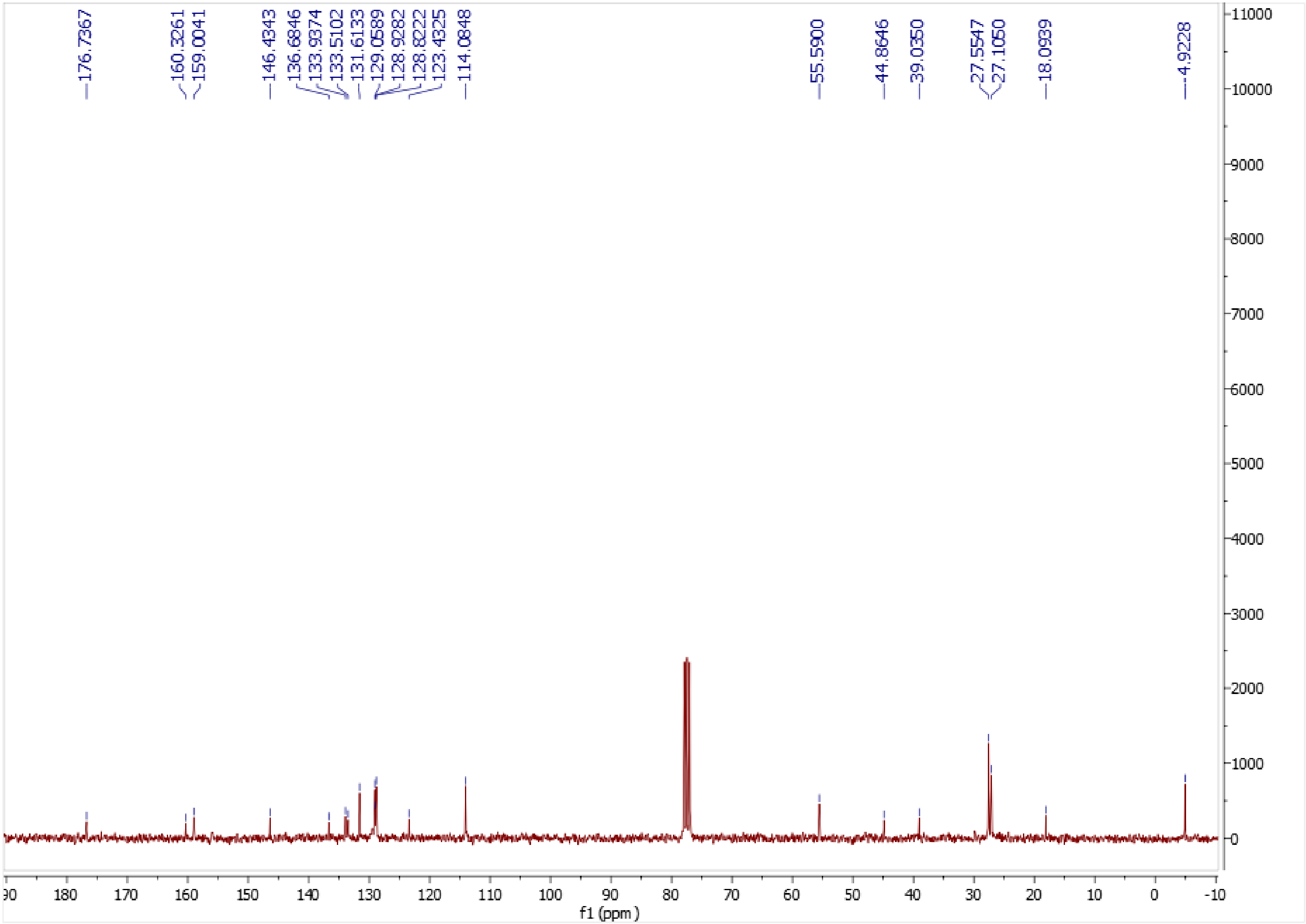

^{1}H NMR spectrum ($CDCl_3$, 300 MHz) of compound **4e** (obtained by Method B)

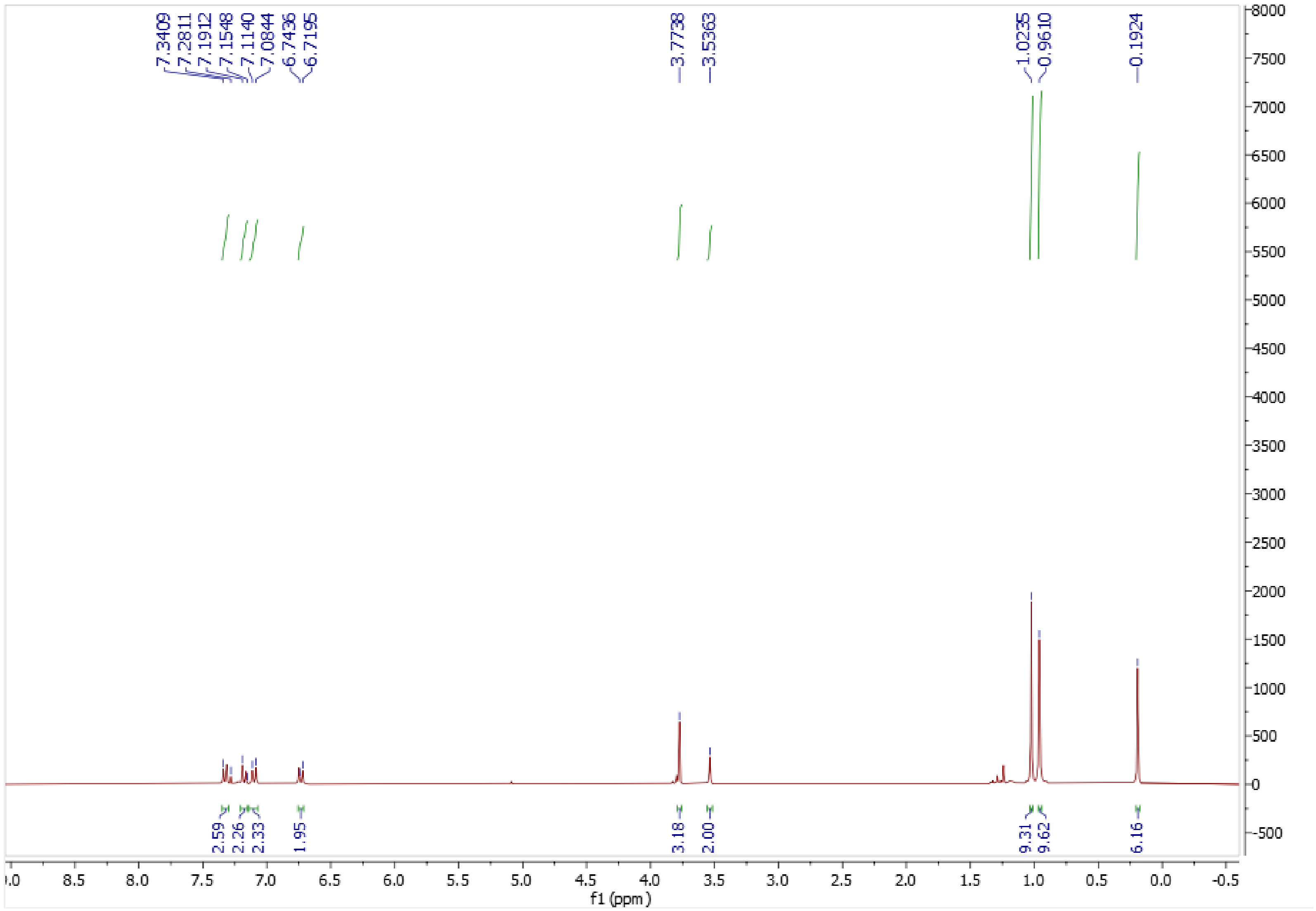

^{1}H NMR spectrum ($CDCl_3$, 300 MHz) of compound **4f** (obtained by Method A)

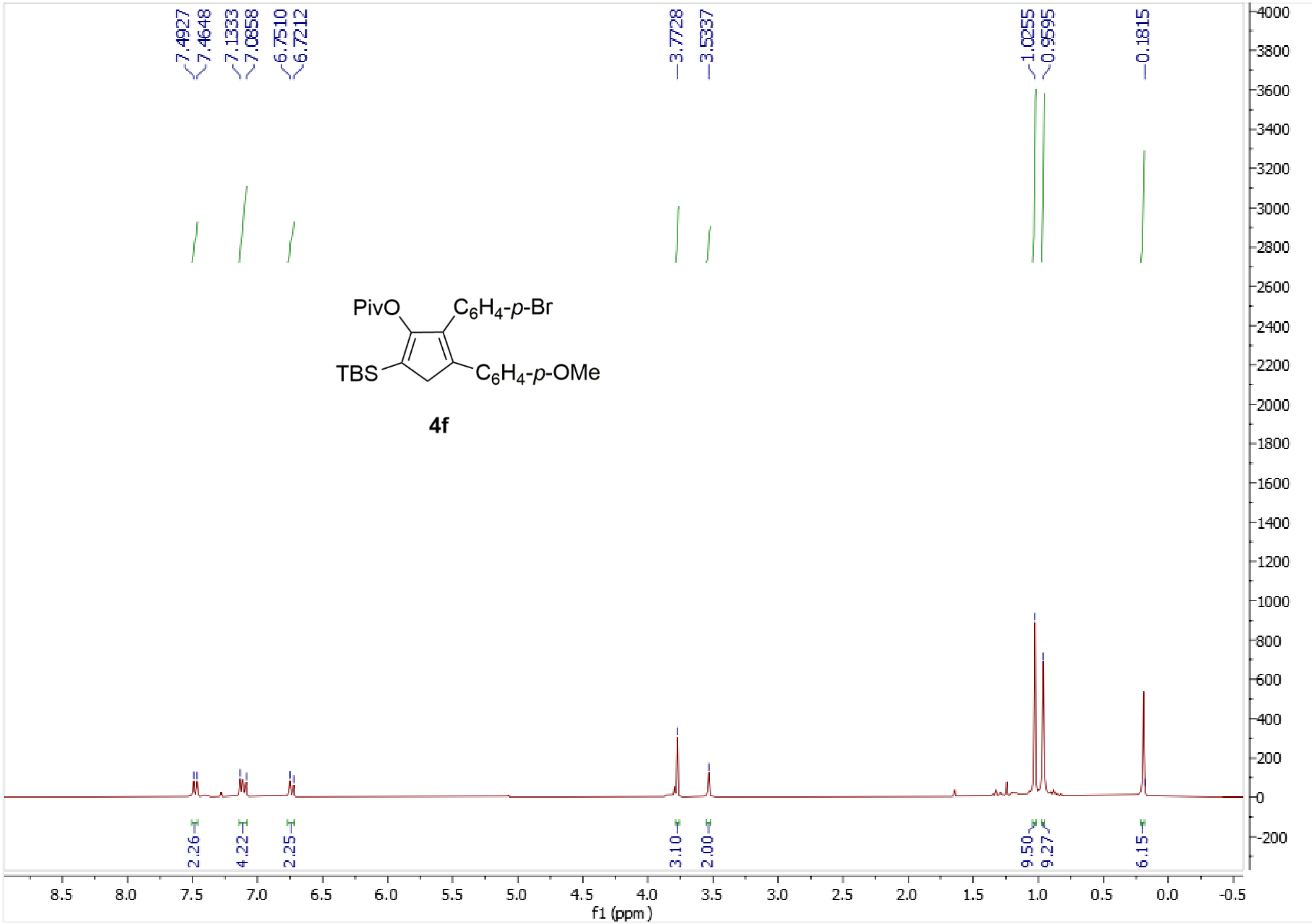

^{13}C NMR spectrum ($CDCl_3$, 75 MHz) of compound **4f** (obtained by Method A)

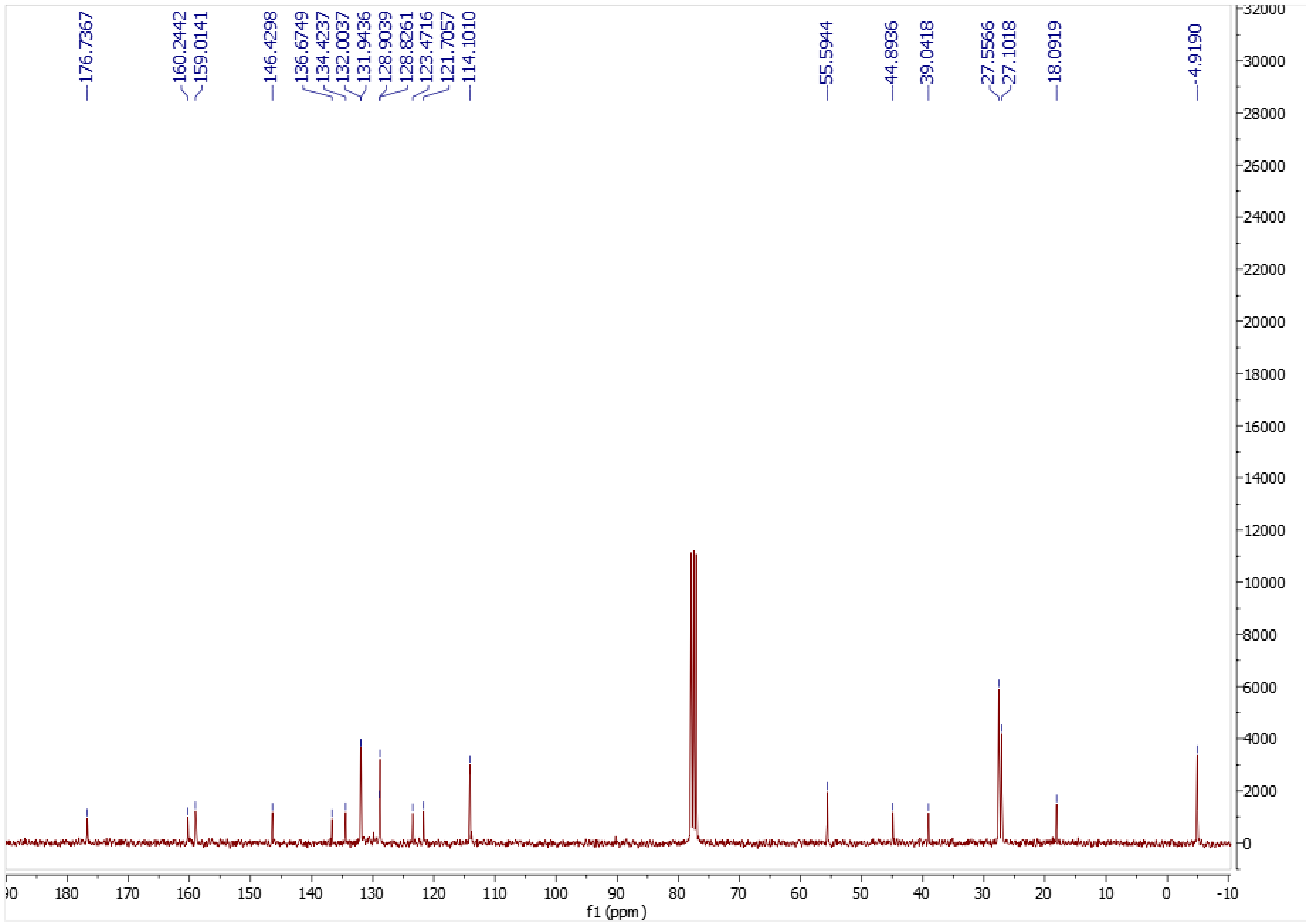

^{1}H NMR spectrum ($CDCl_3$, 300 MHz) of compound **4f** (obtained by Method B)

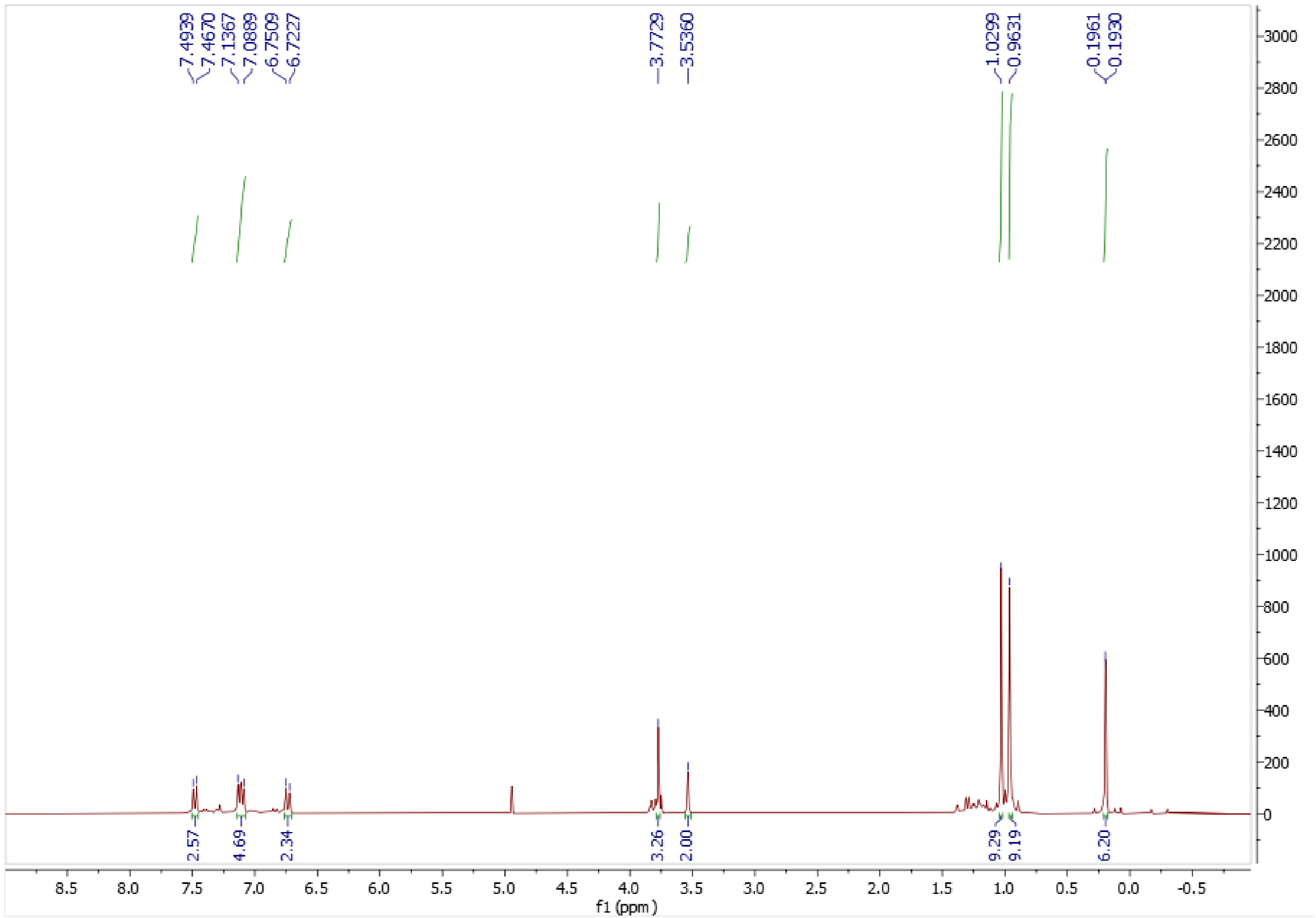

^{1}H NMR spectrum ($CDCl_3$, 300 MHz) of compound **4g**

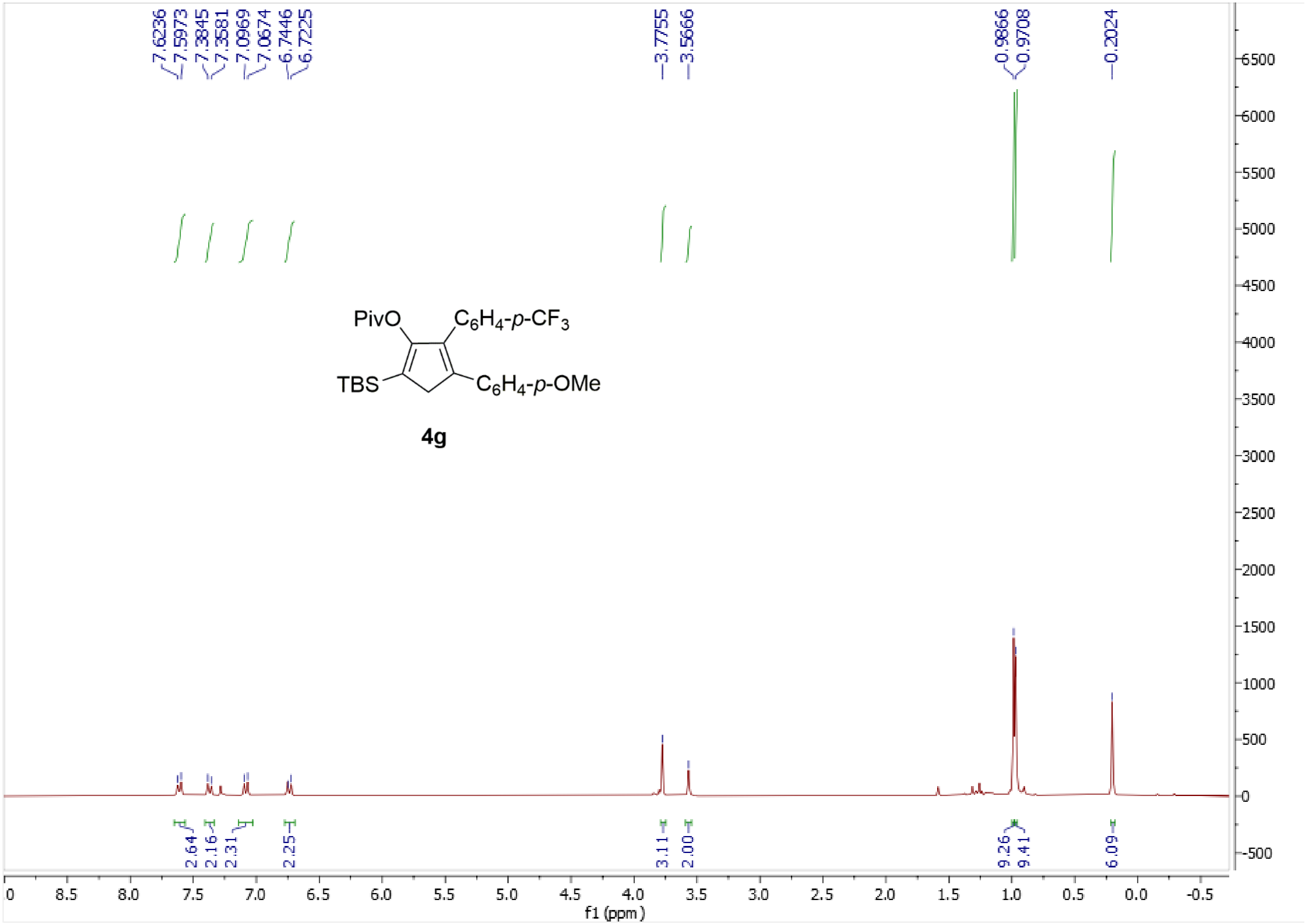

^{13}C NMR spectrum ($CDCl_3$, 75 MHz) of compound **4g**

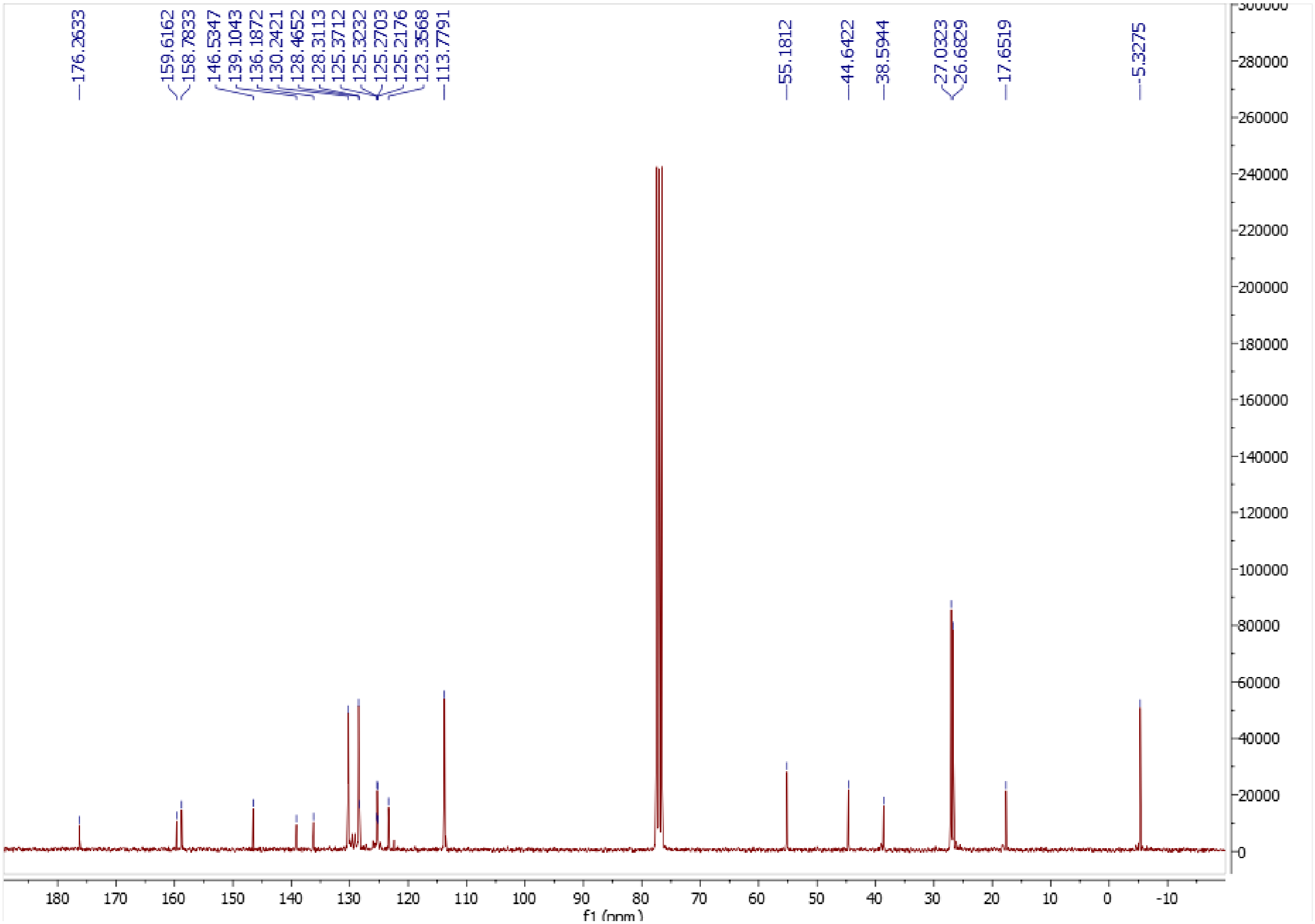

^{19}F NMR spectrum ($CDCl_3$, 282 MHz) of compound **4g**

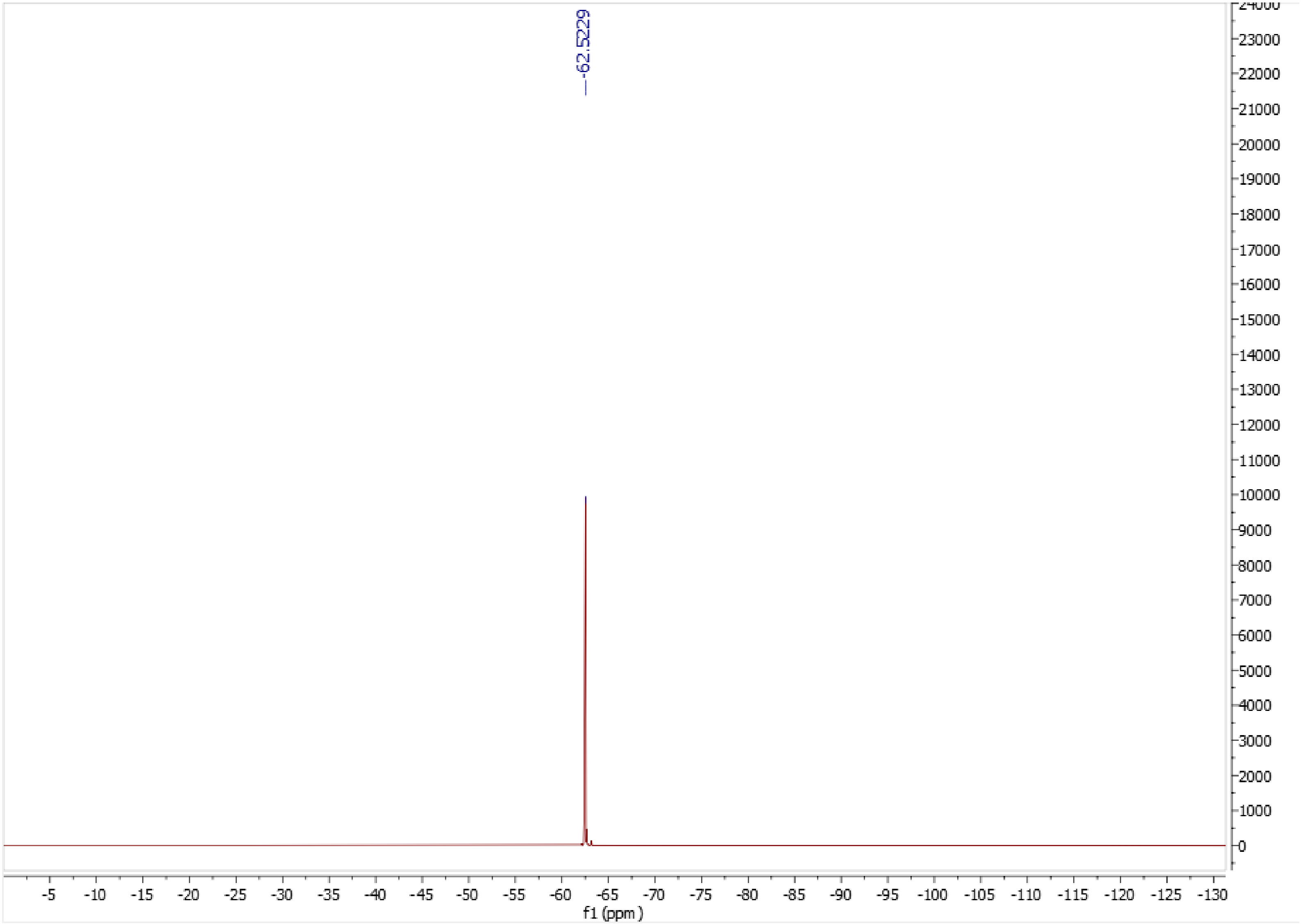

^{1}H NMR spectrum ($CDCl_3$, 300 MHz) of compound **4h** (obtained by Method A)

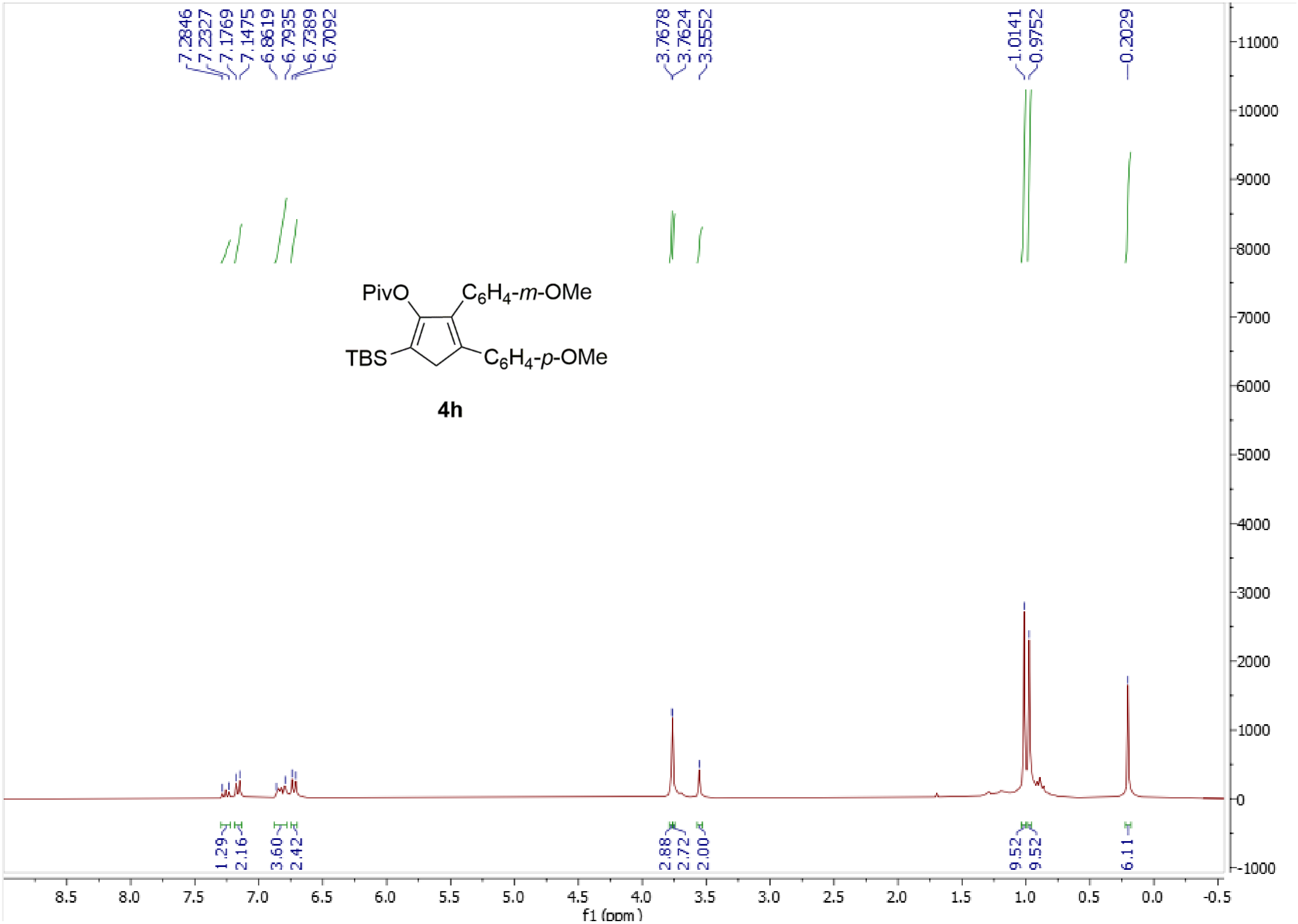

^{13}C NMR spectrum ($CDCl_3$, 75 MHz) of compound **4h** (obtained by Method A)

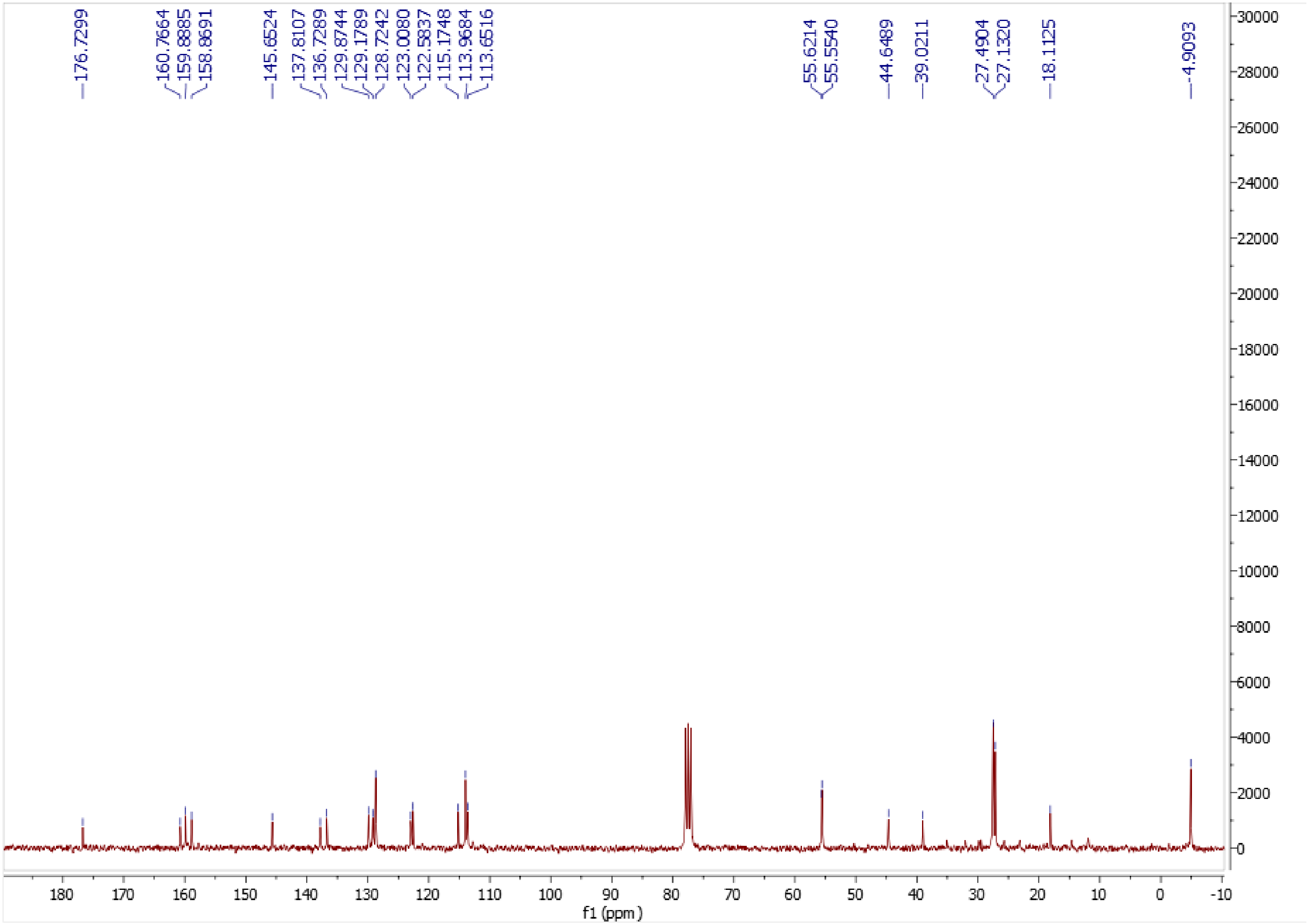

^{1}H NMR spectrum ($CDCl_3$, 300 MHz) of compound **4h** (obtained by Method B)

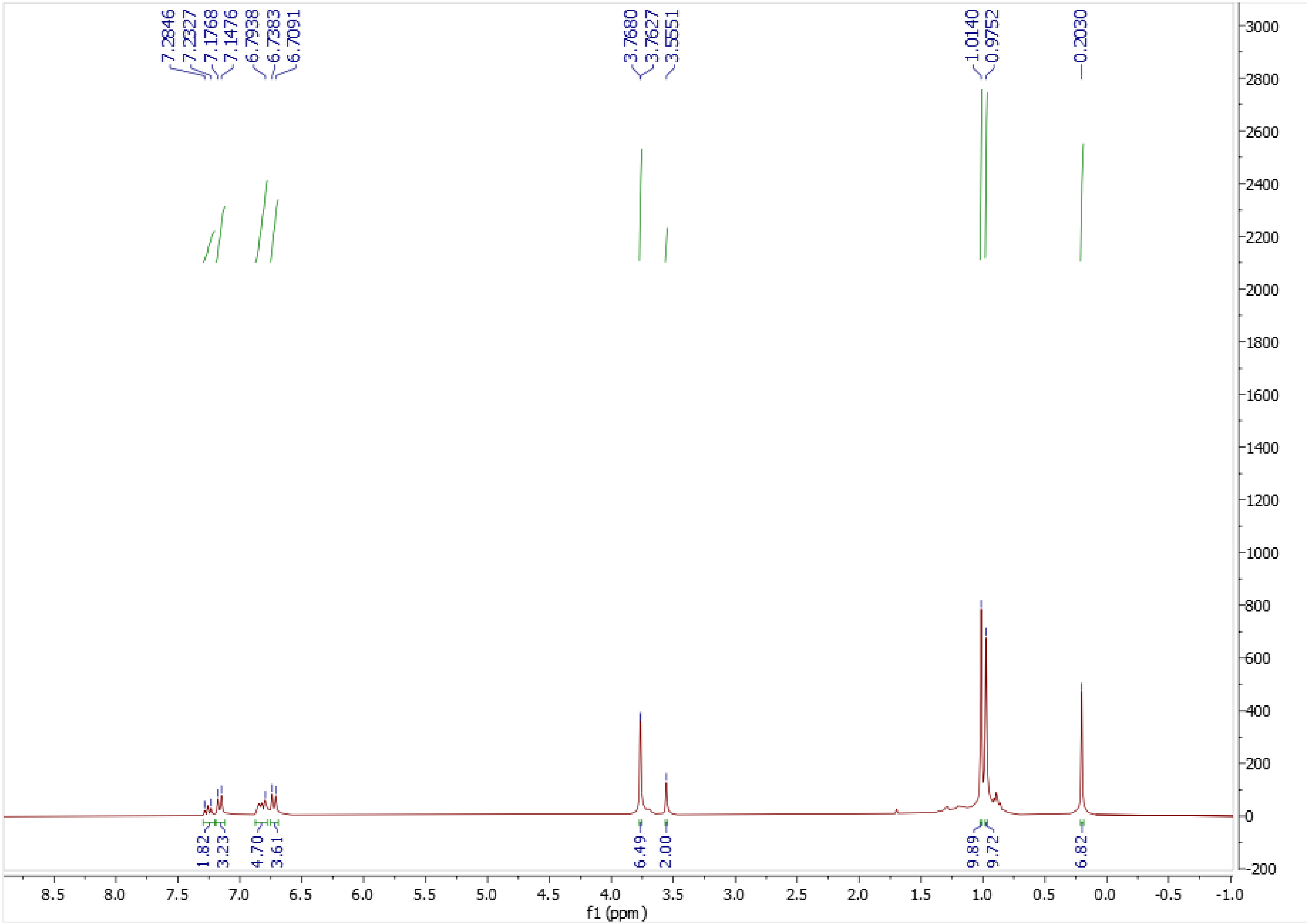

^{1}H NMR spectrum ($CDCl_3$, 300 MHz) of compound **4i**

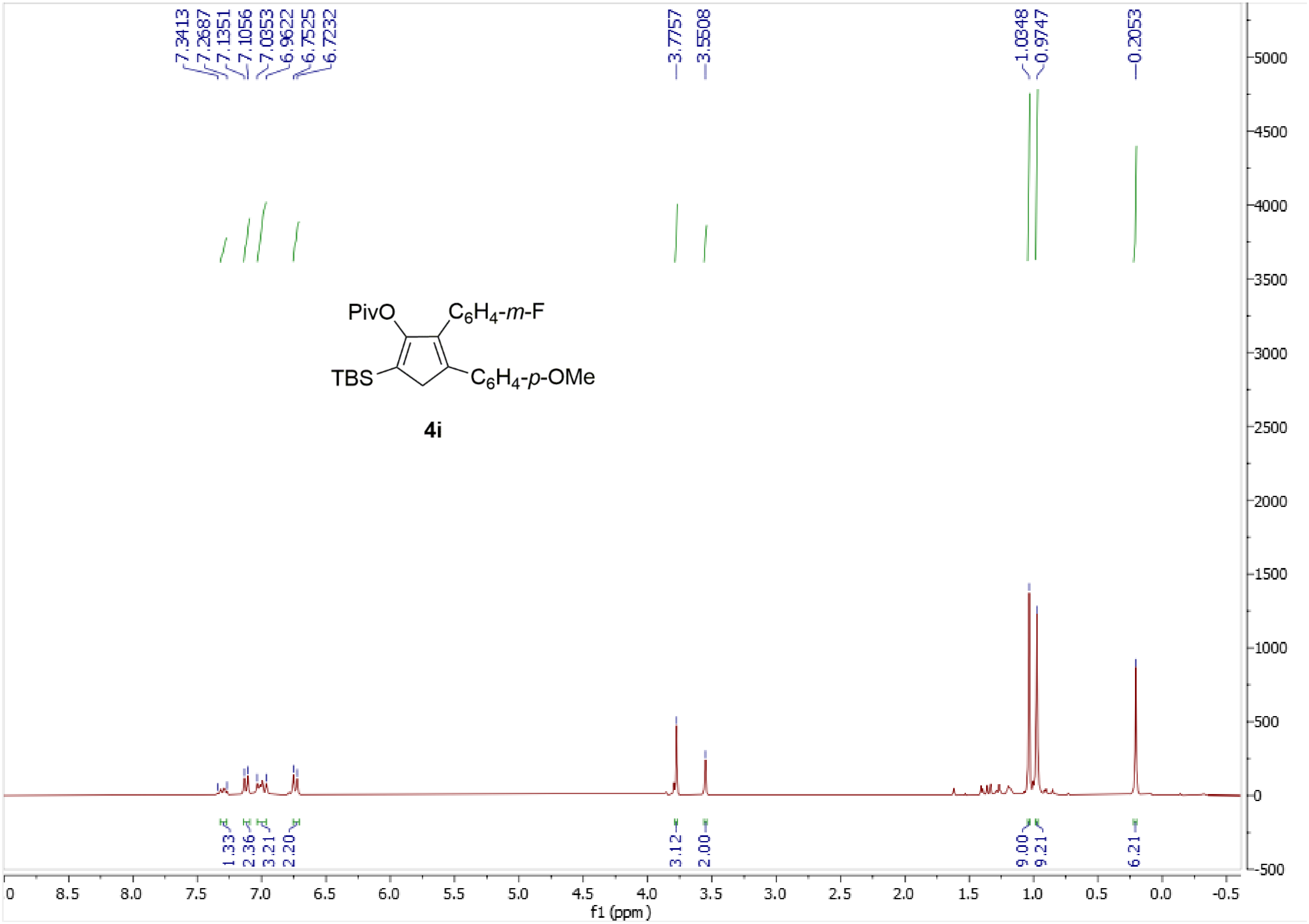

^{13}C NMR spectrum ($CDCl_3$, 75 MHz) of compound **4i**

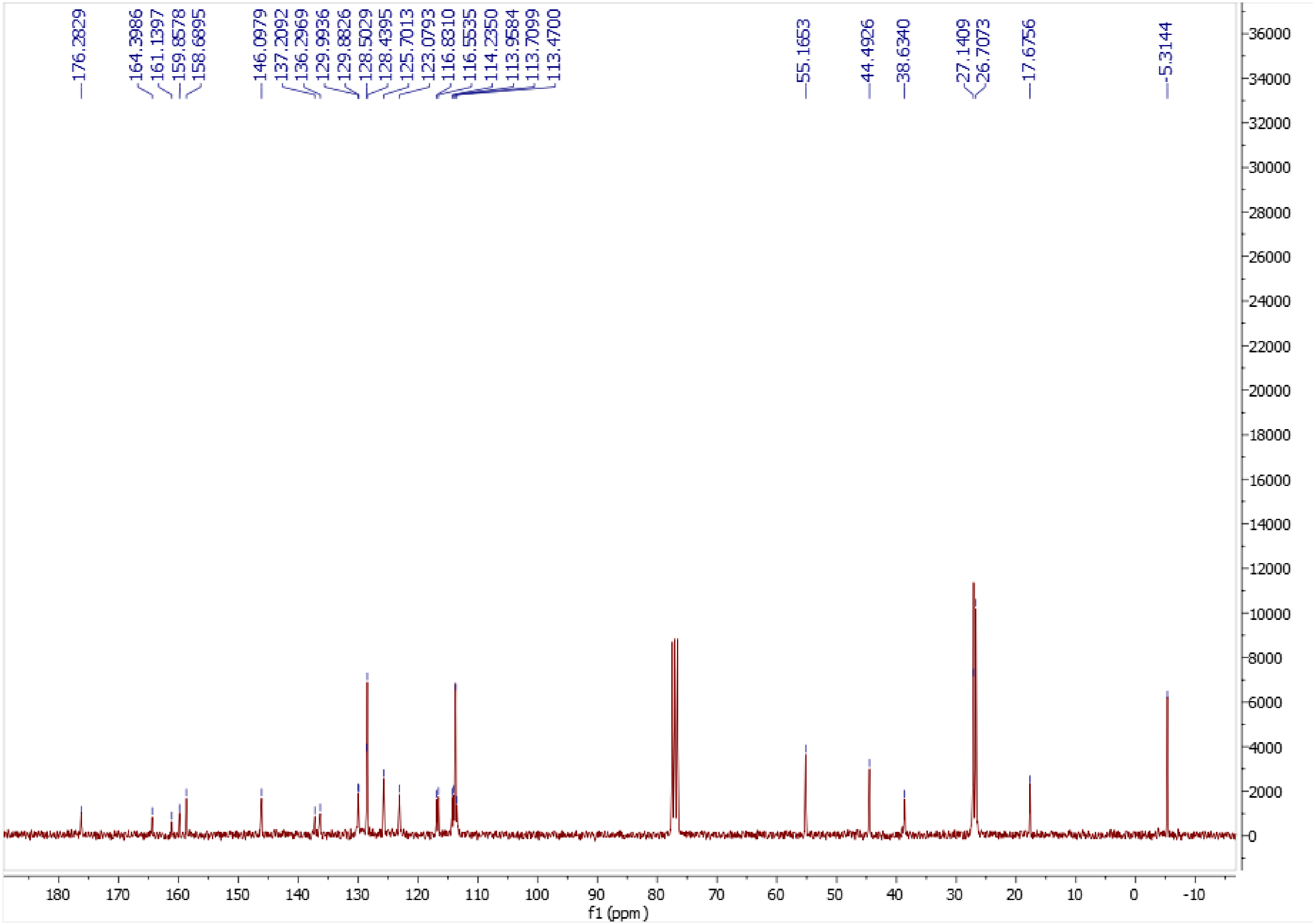

^{19}F NMR spectrum ($CDCl_3$, 282 MHz) of compound **4i**

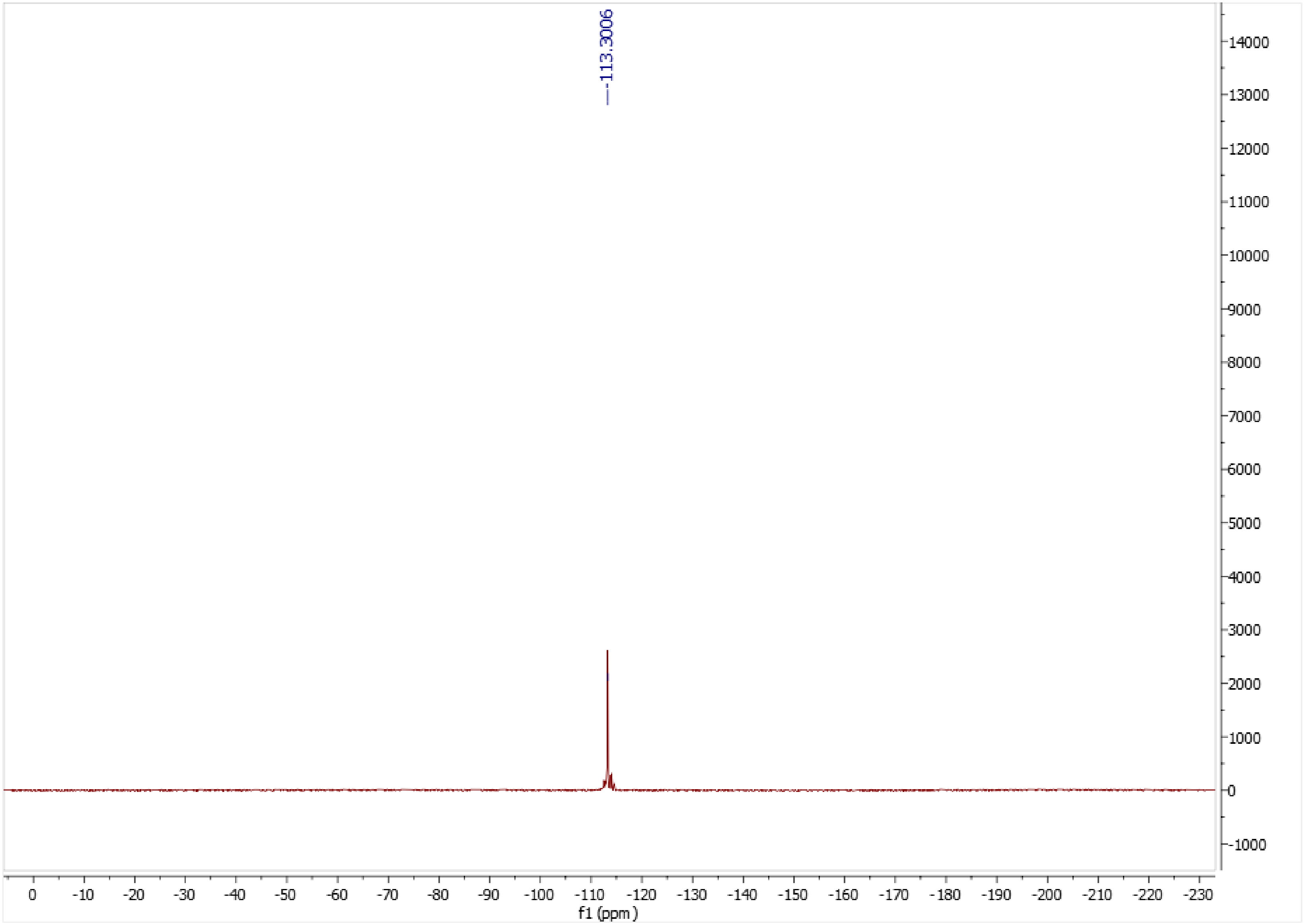

^{1}H NMR spectrum ($CDCl_3$, 300 MHz) of compound **4j** (obtained by Method A)

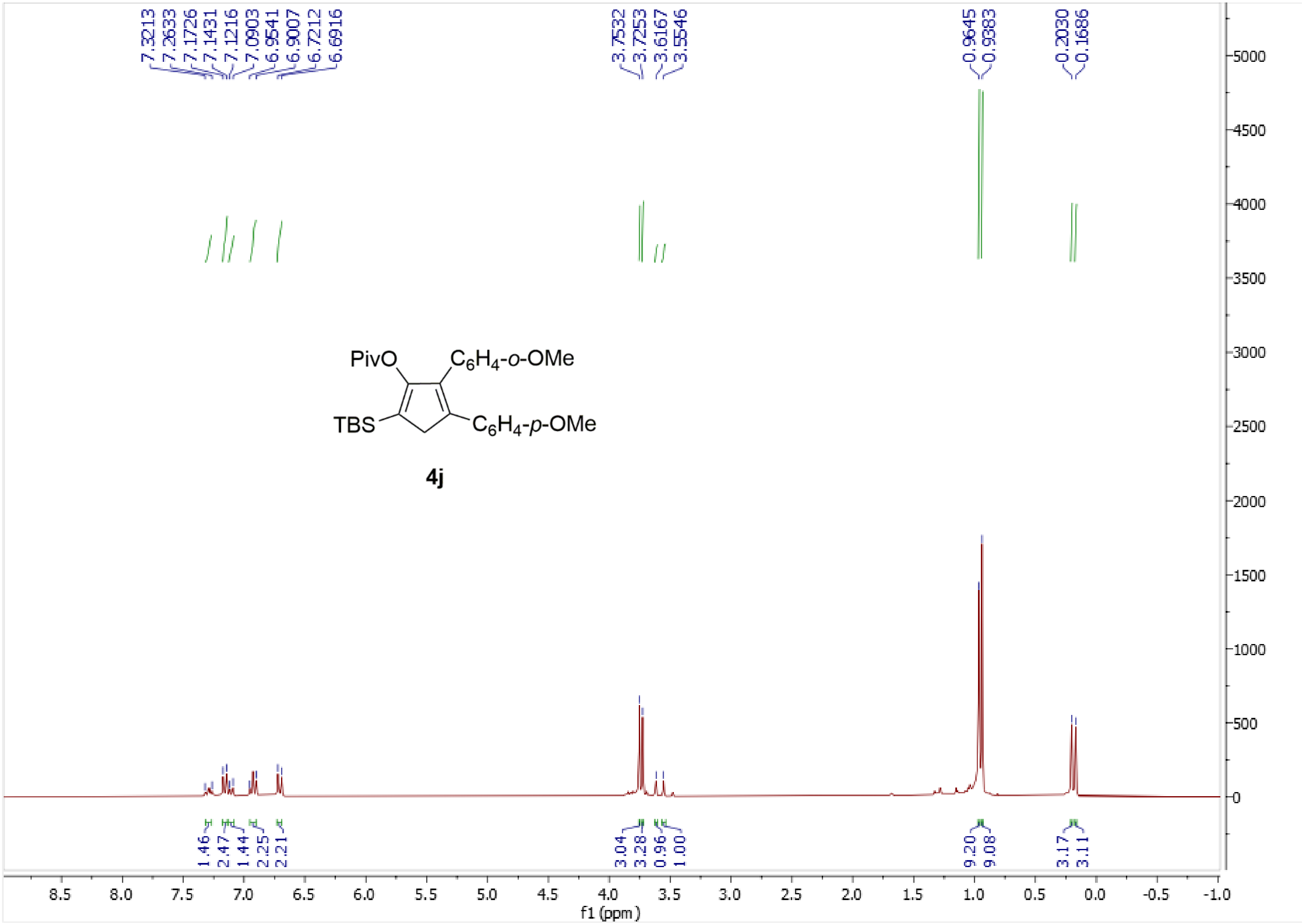

^{13}C NMR spectrum ($CDCl_3$, 75 MHz) of compound **4j** (obtained by Method A)

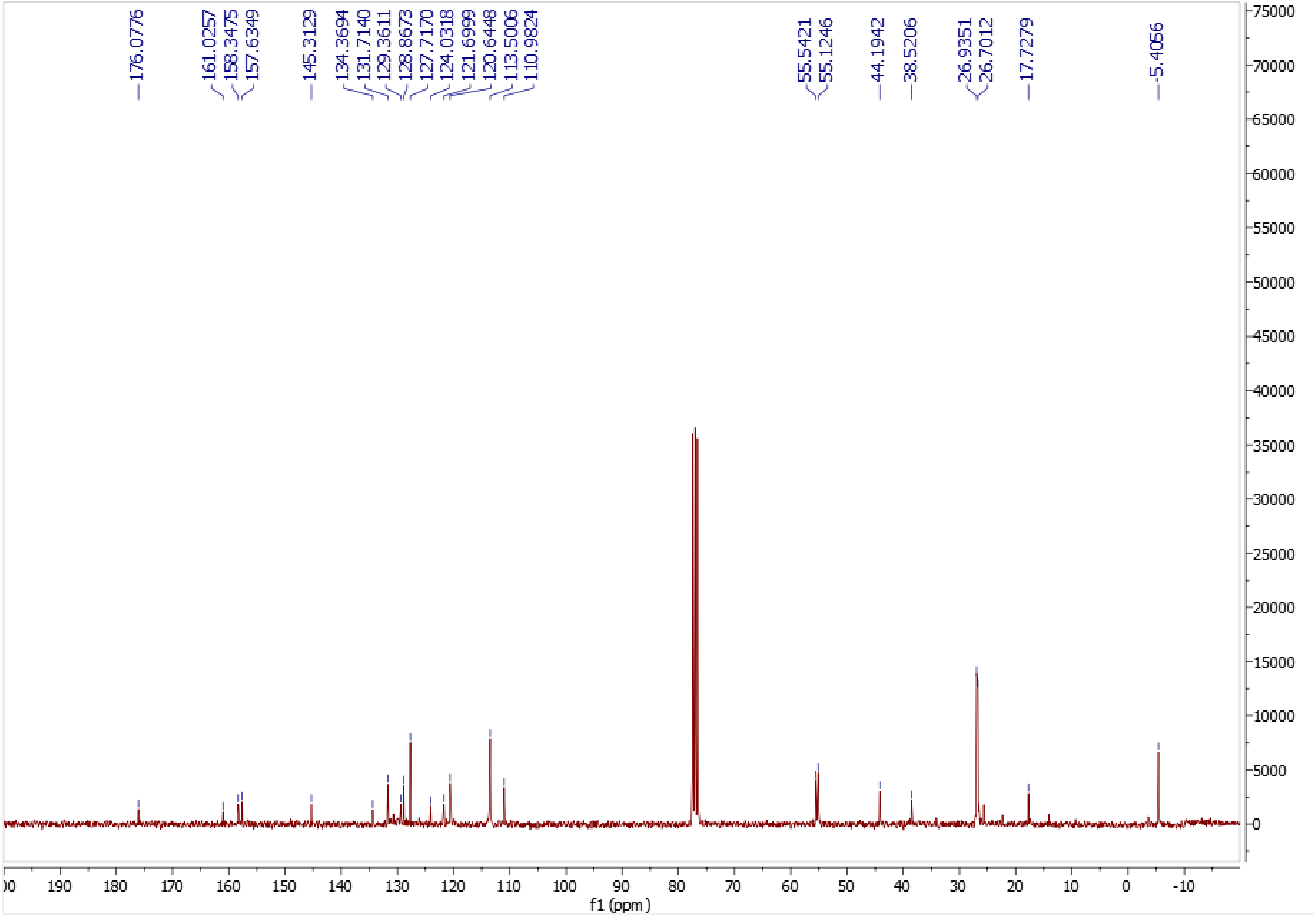

^{1}H NMR spectrum ($CDCl_3$, 300 MHz) of compound **4j** (obtained by Method B)

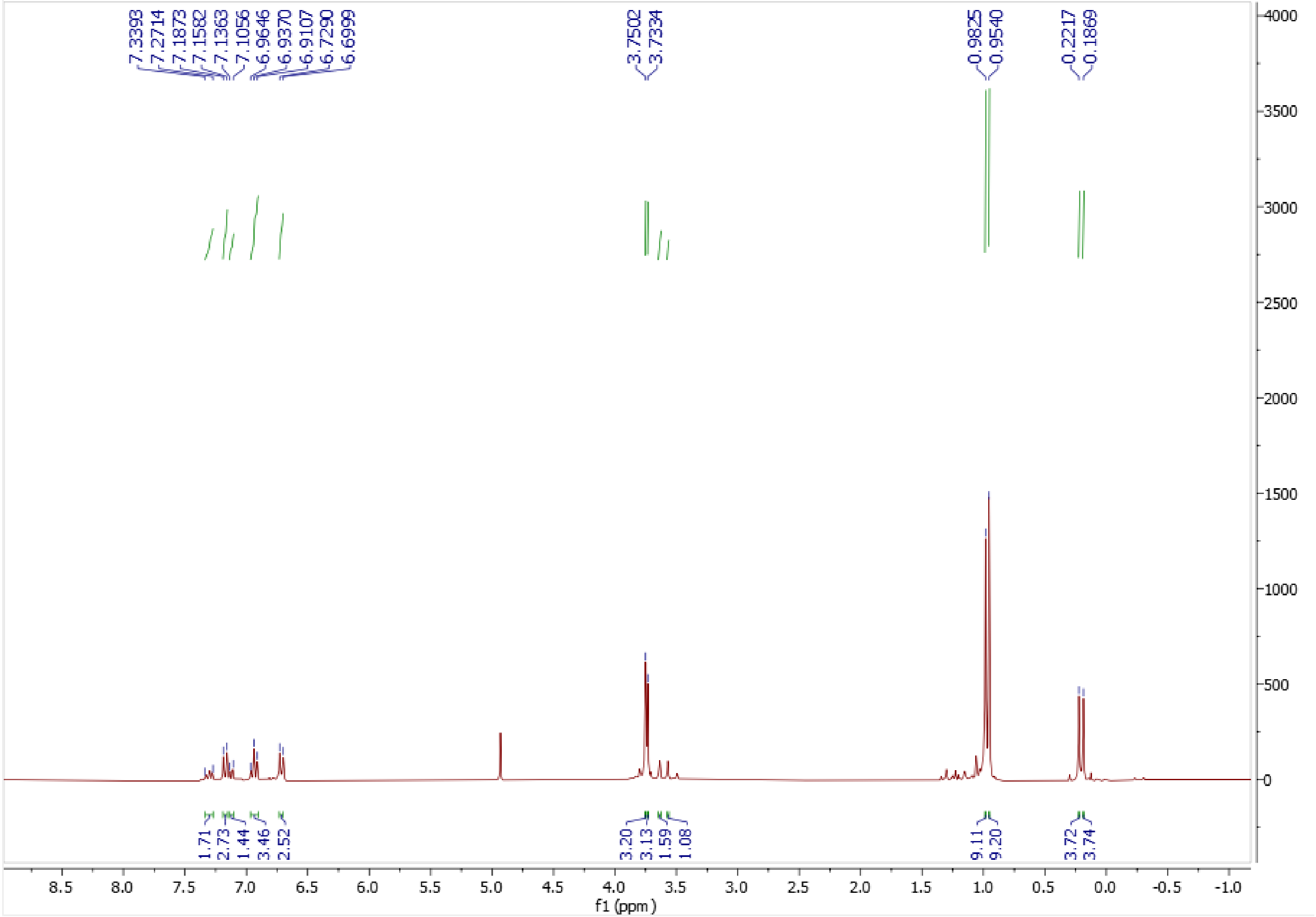

^{1}H NMR spectrum ($CDCl_3$, 300 MHz) of compound **4k** (obtained by Method A)

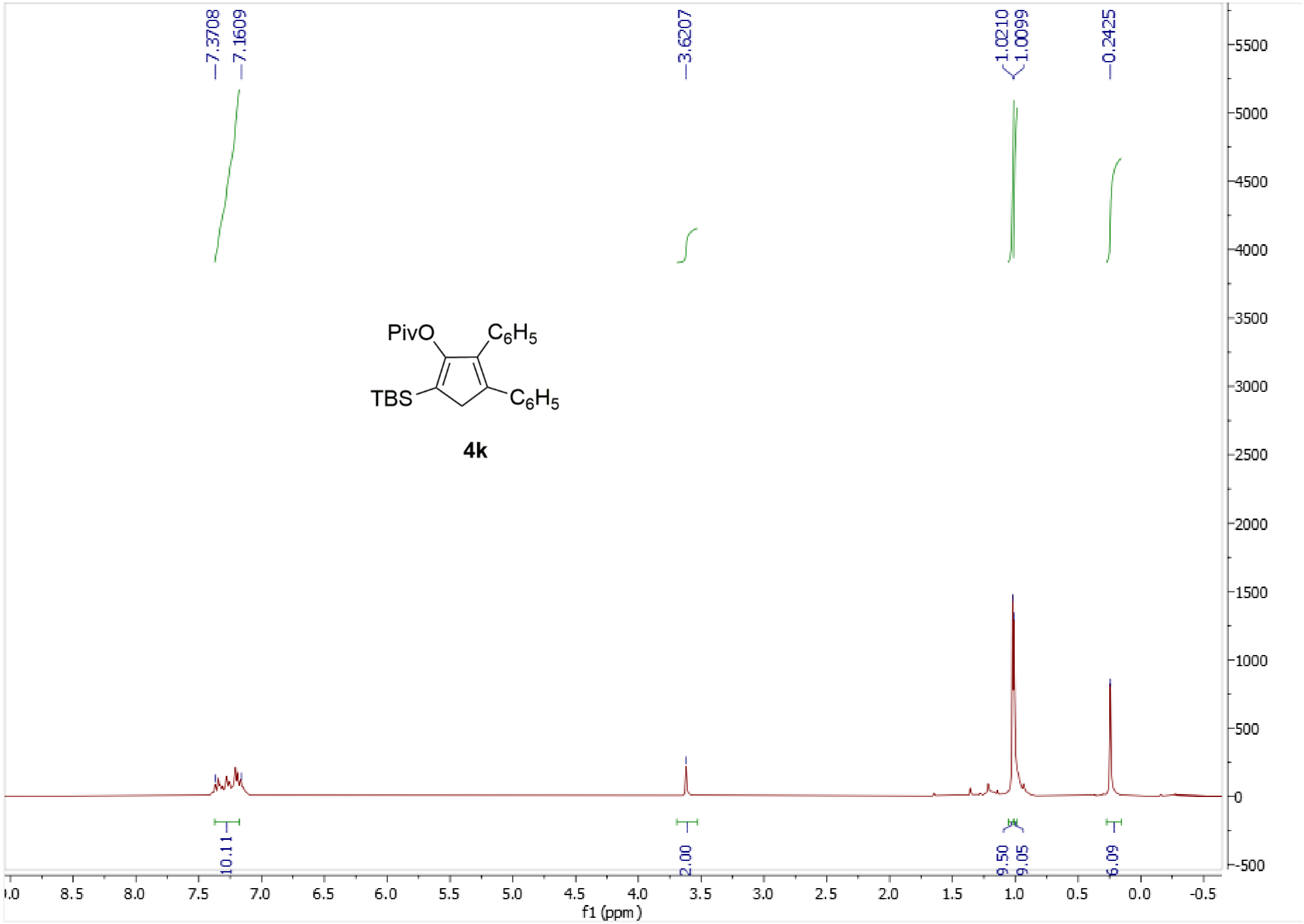

^{13}C NMR spectrum ($CDCl_3$, 75 MHz) of compound **4k** (obtained by Method A)

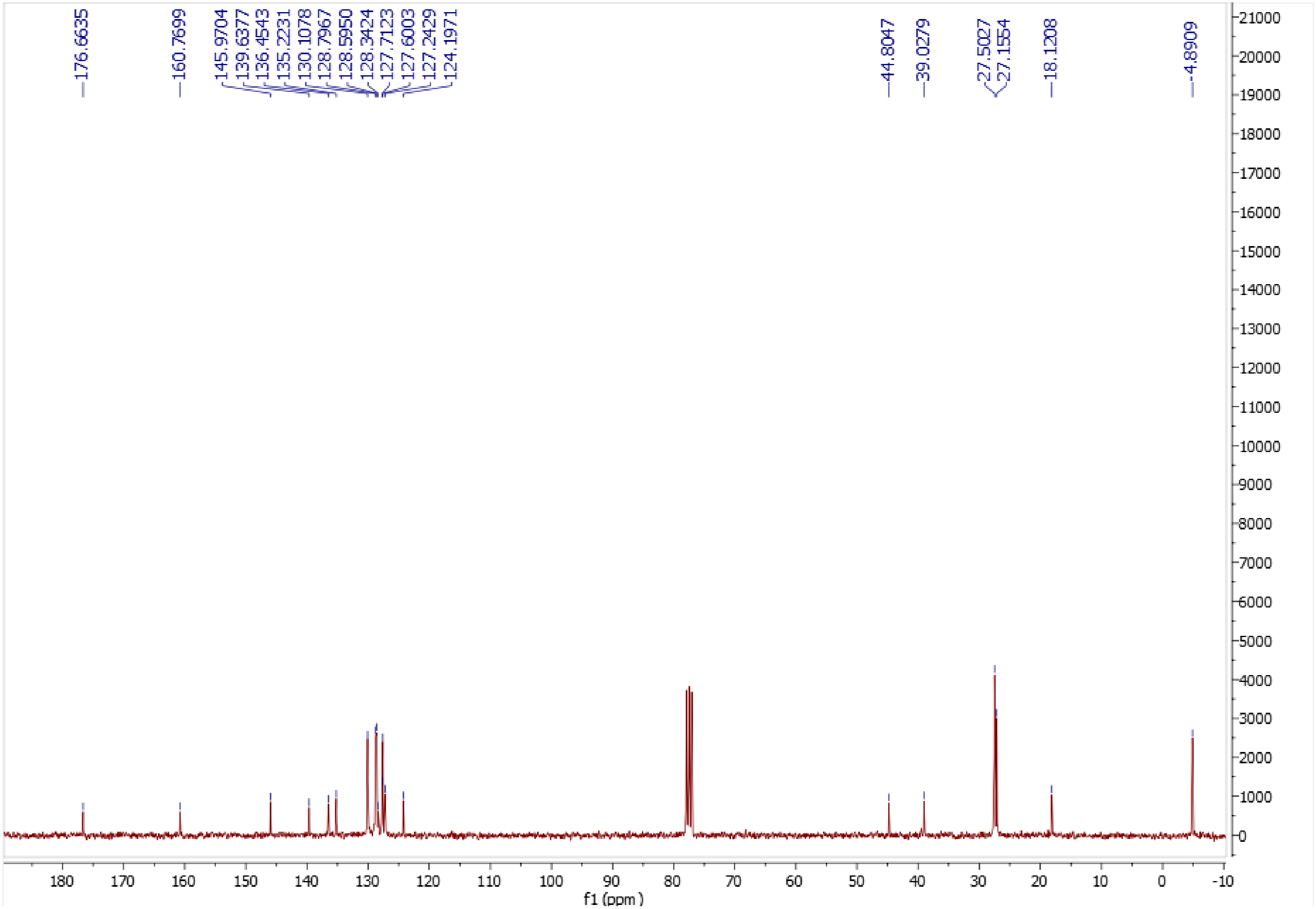

^{1}H NMR spectrum ($CDCl_3$, 300 MHz) of compound **4k** (obtained by Method B)

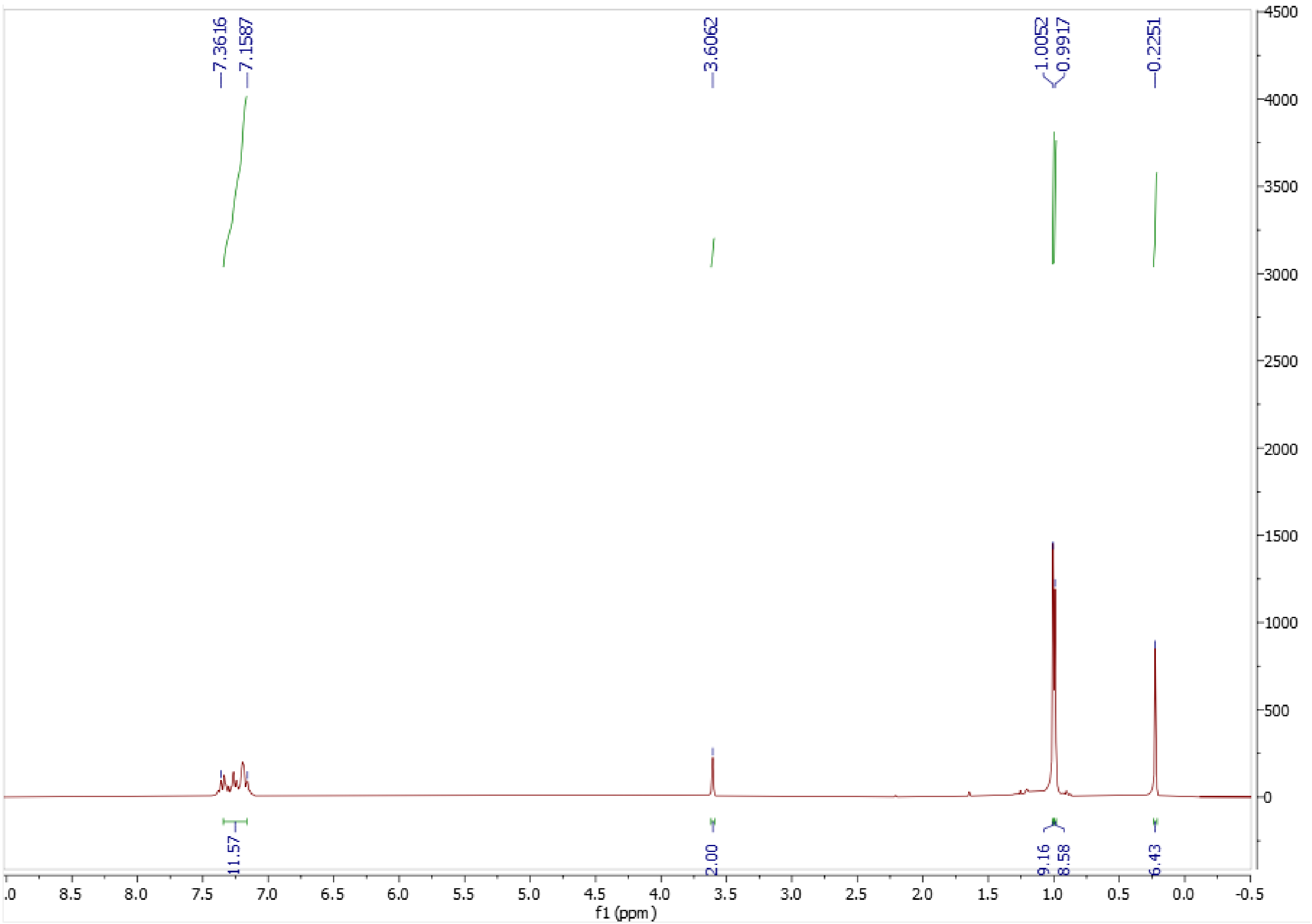

^{1}H NMR spectrum ($CDCl_3$, 300 MHz) of compound **4l** (obtained by Method A)

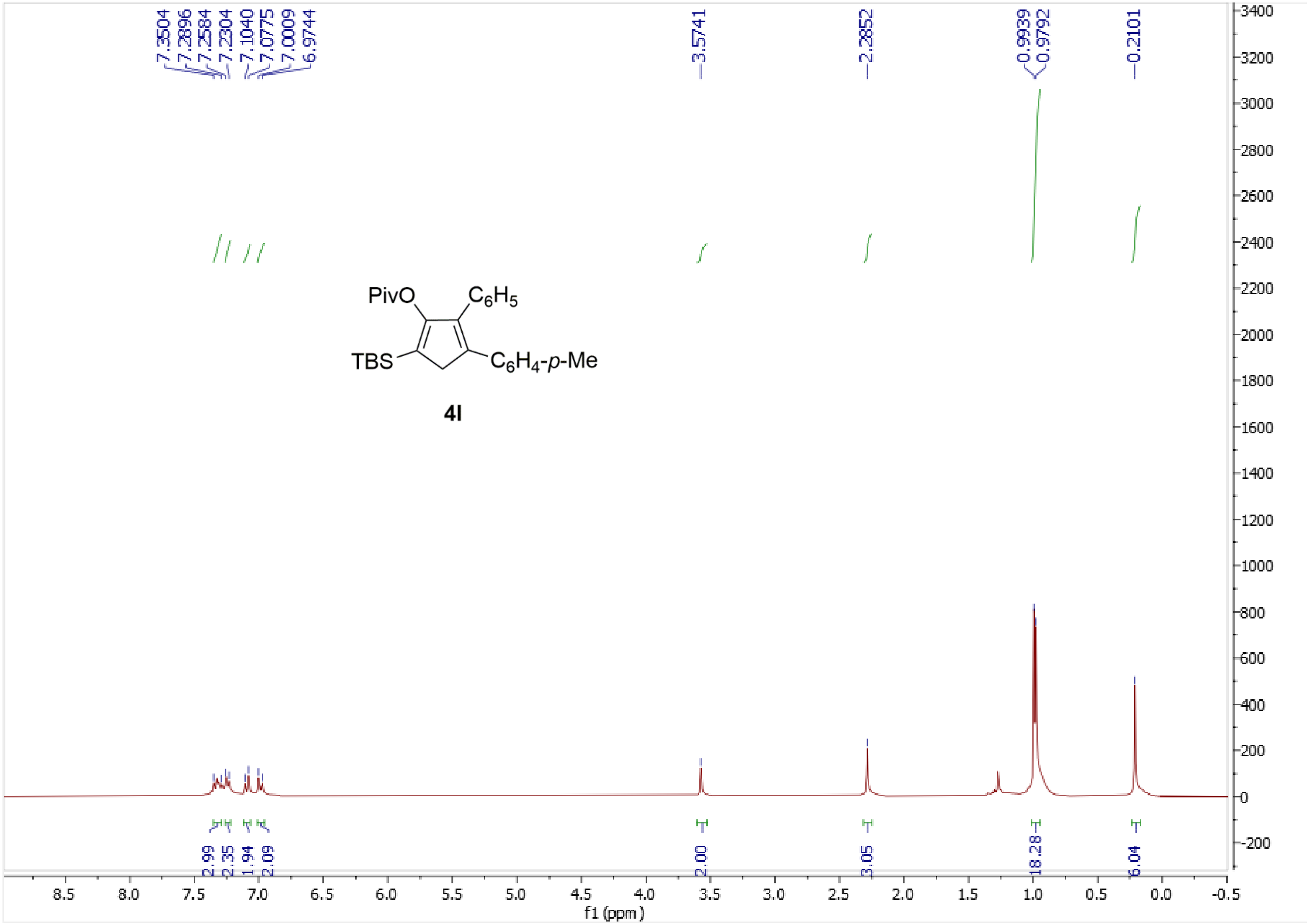

^{13}C NMR spectrum ($CDCl_3$, 75 MHz) of compound **4l** (obtained by Method A)

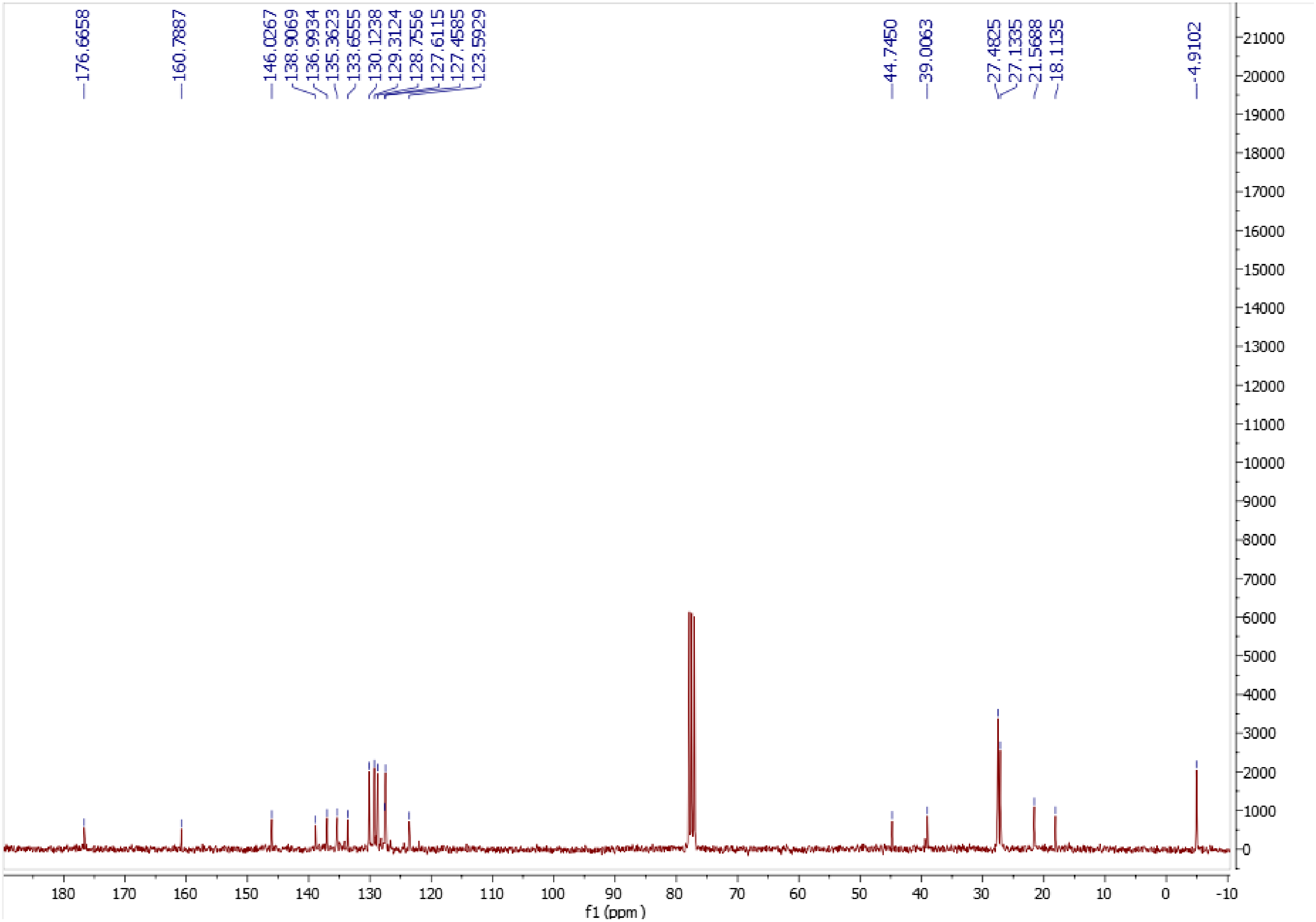

^{1}H NMR spectrum ($CDCl_3$, 300 MHz) of compound **4l** (obtained by Method B)

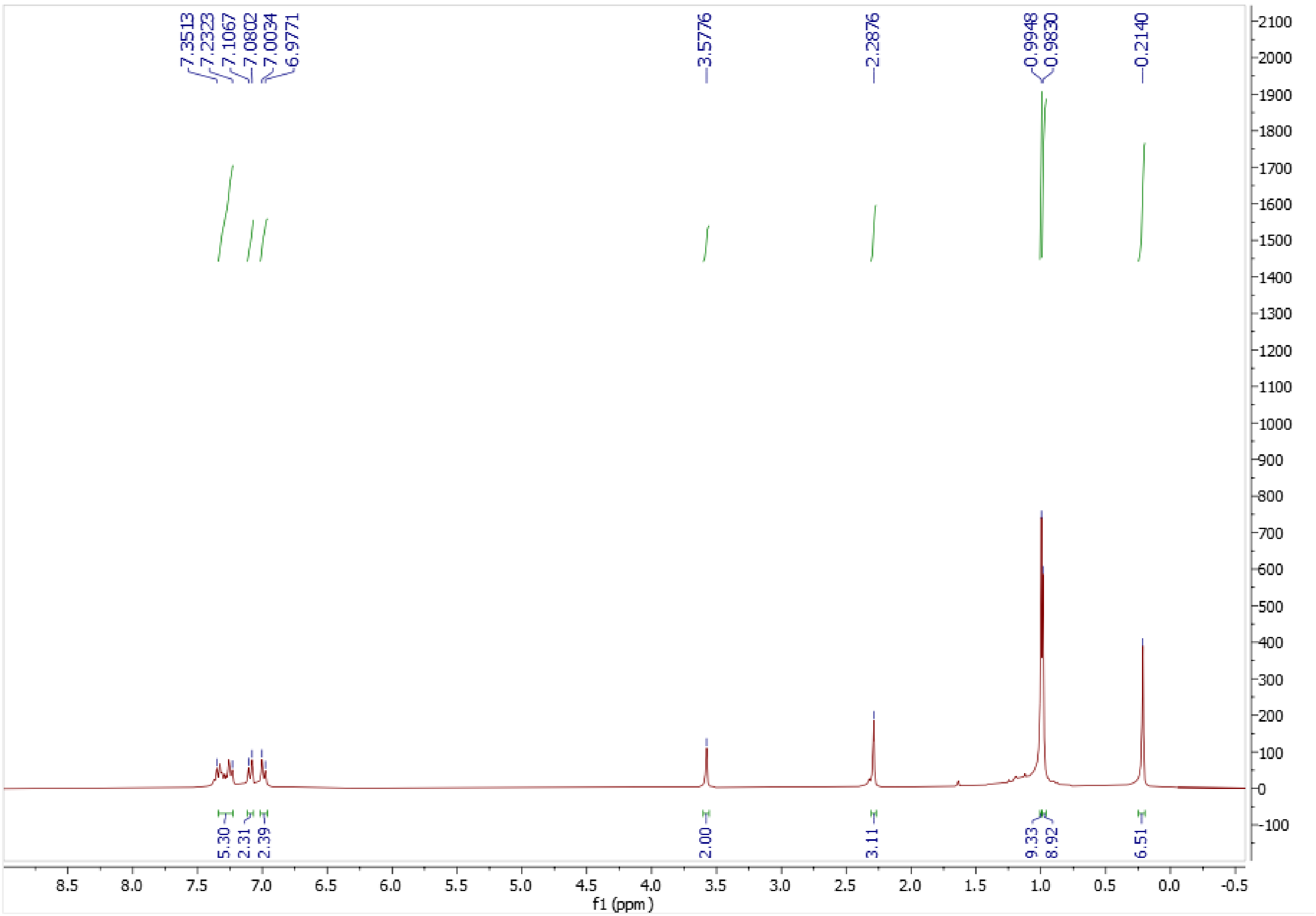

^{1}H NMR spectrum ($CDCl_3$, 300 MHz) of compound **4m**

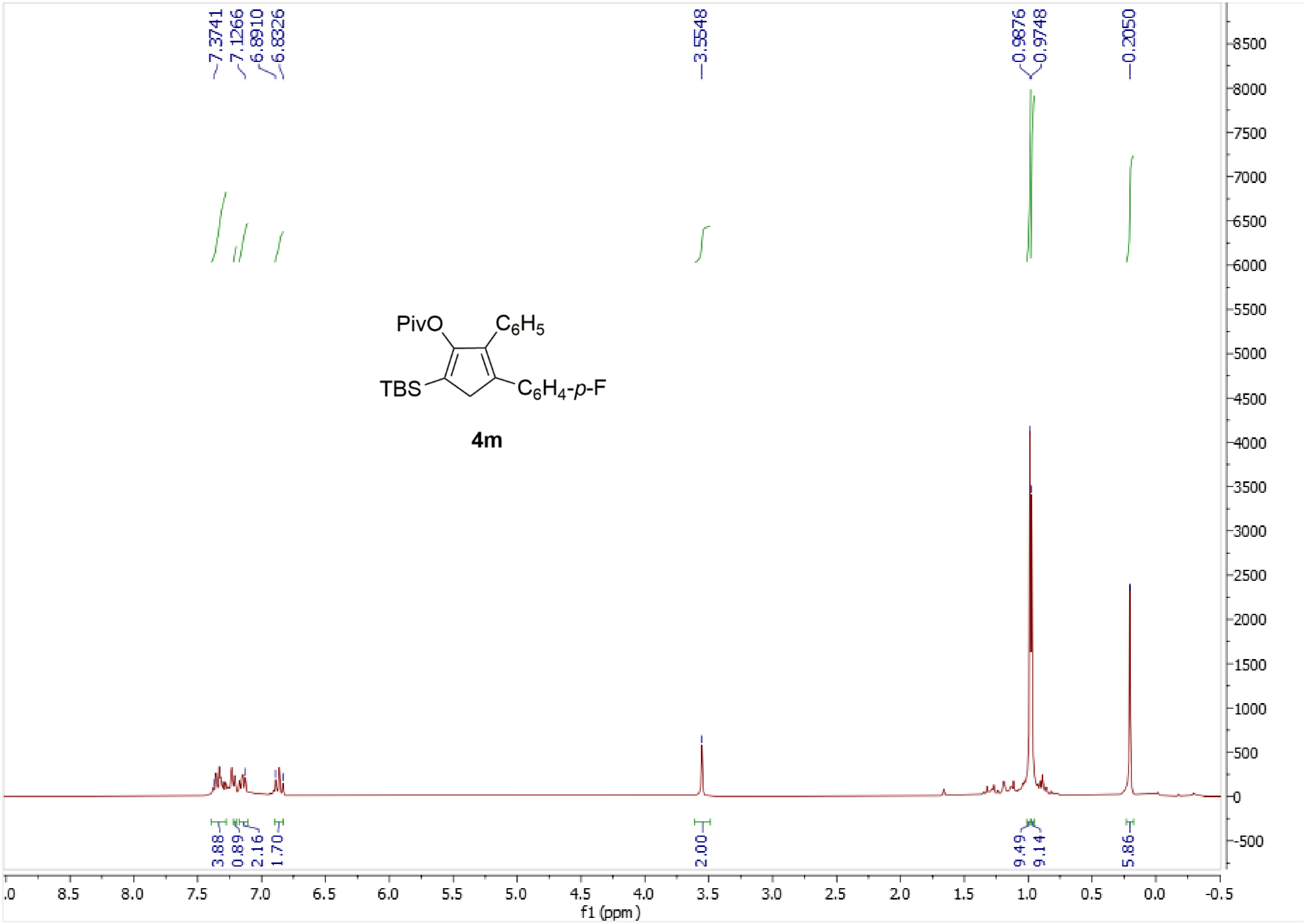

^{13}C NMR spectrum ($CDCl_3$, 75 MHz) of compound **4m**

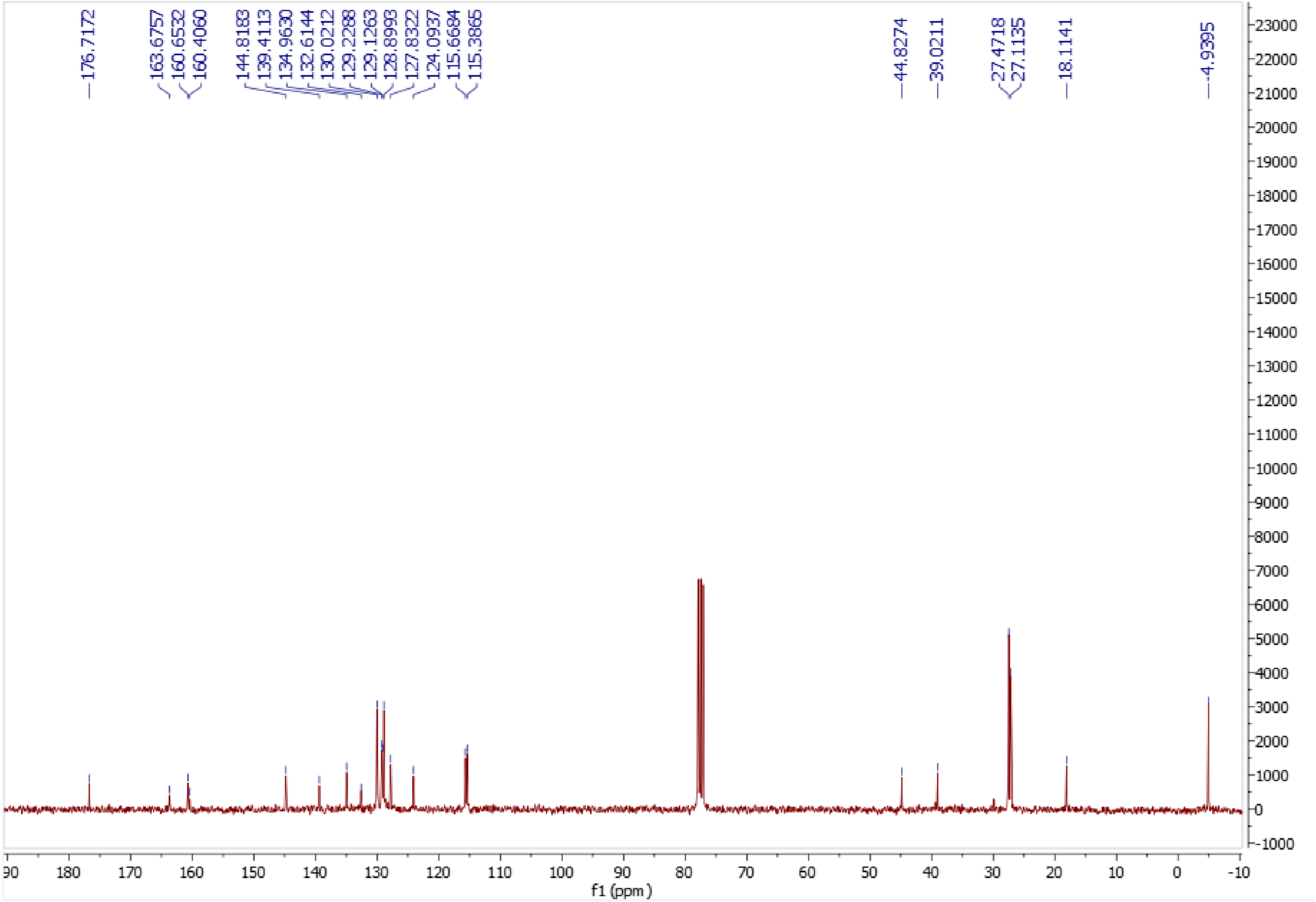

^{19}F NMR spectrum ($CDCl_3$, 282 MHz) of compound **4m**

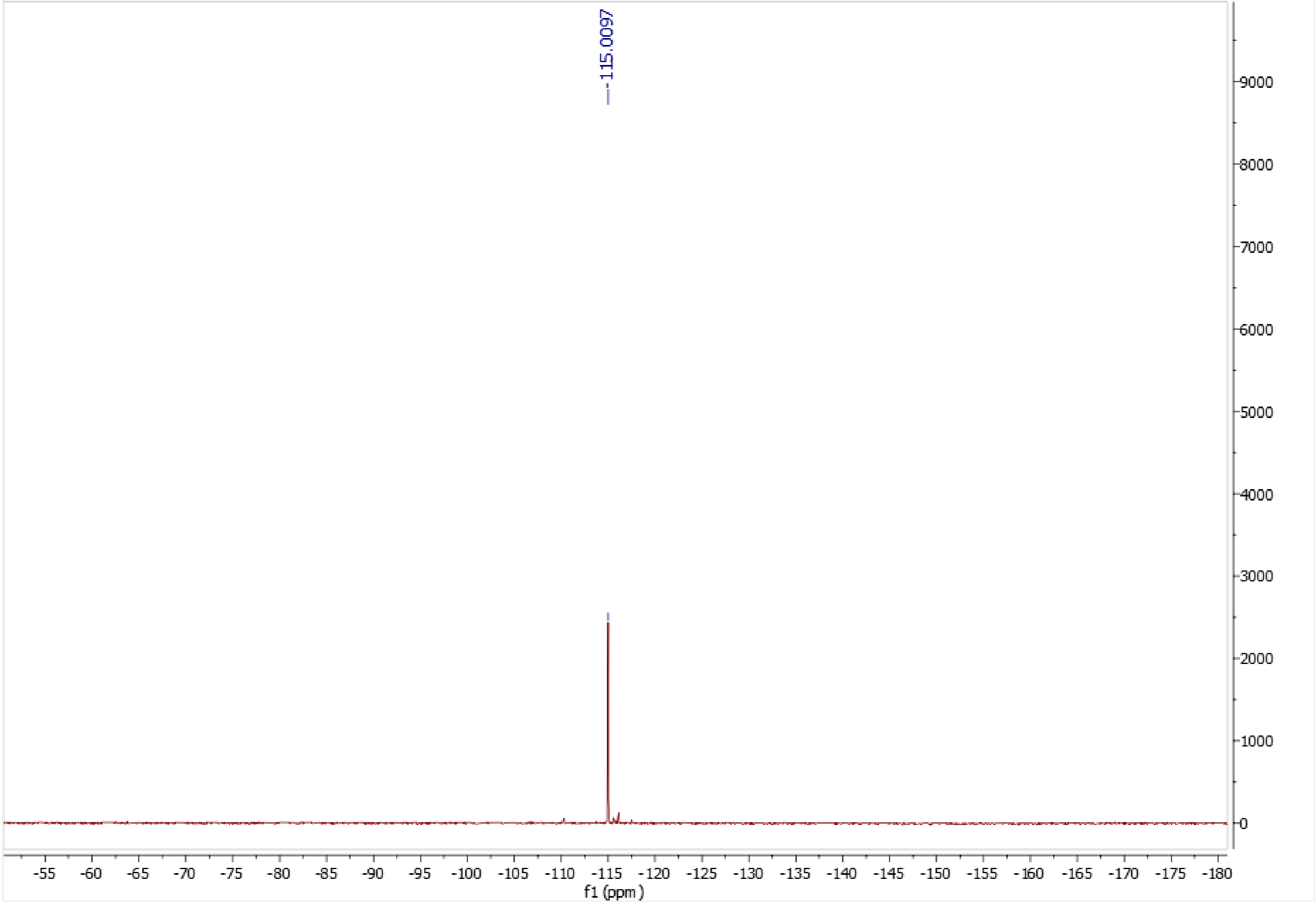

^{1}H NMR spectrum ($CDCl_3$, 300 MHz) of compound **4n**

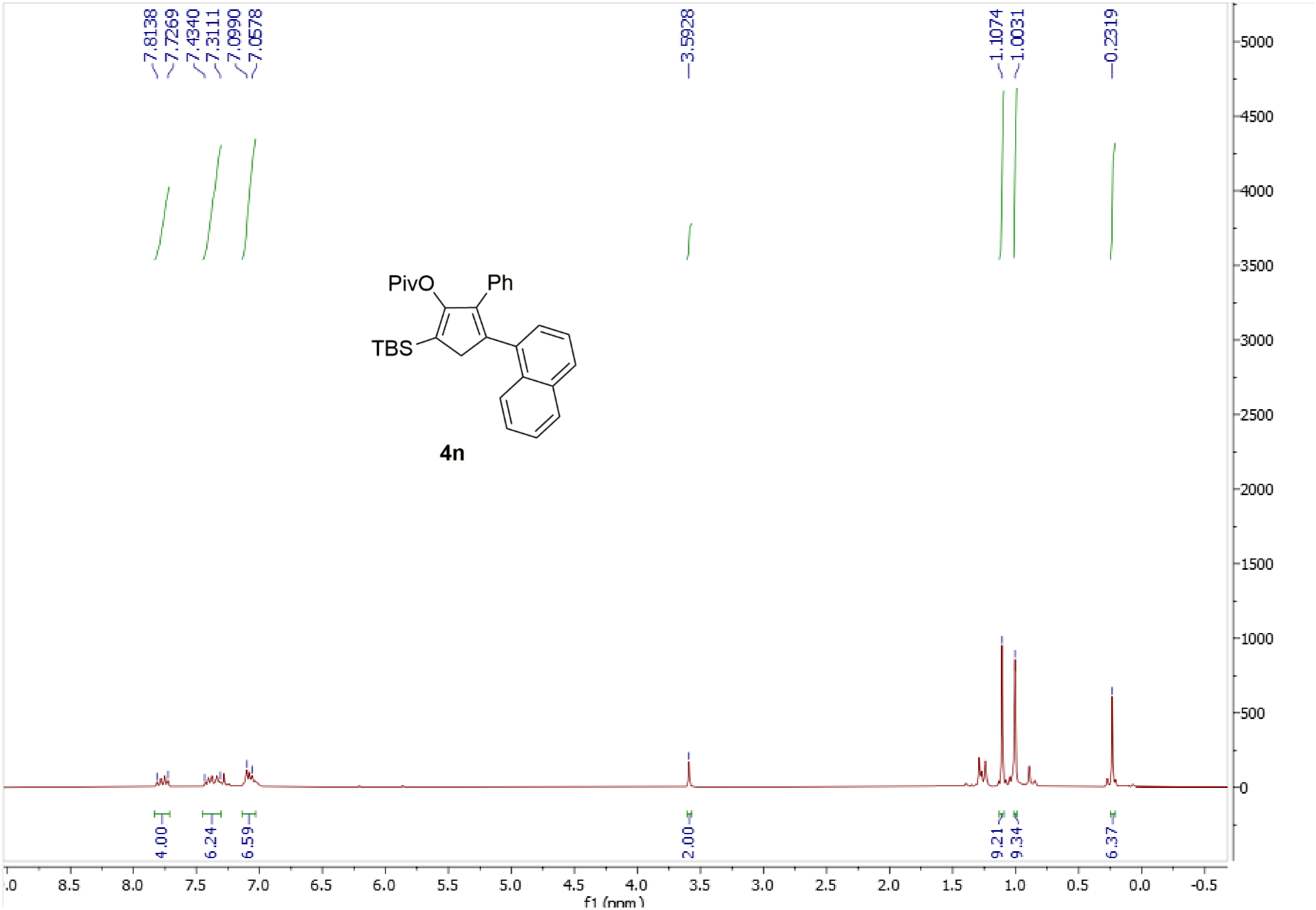

^{13}C NMR spectrum ($CDCl_3$, 75 MHz) of compound **4n**

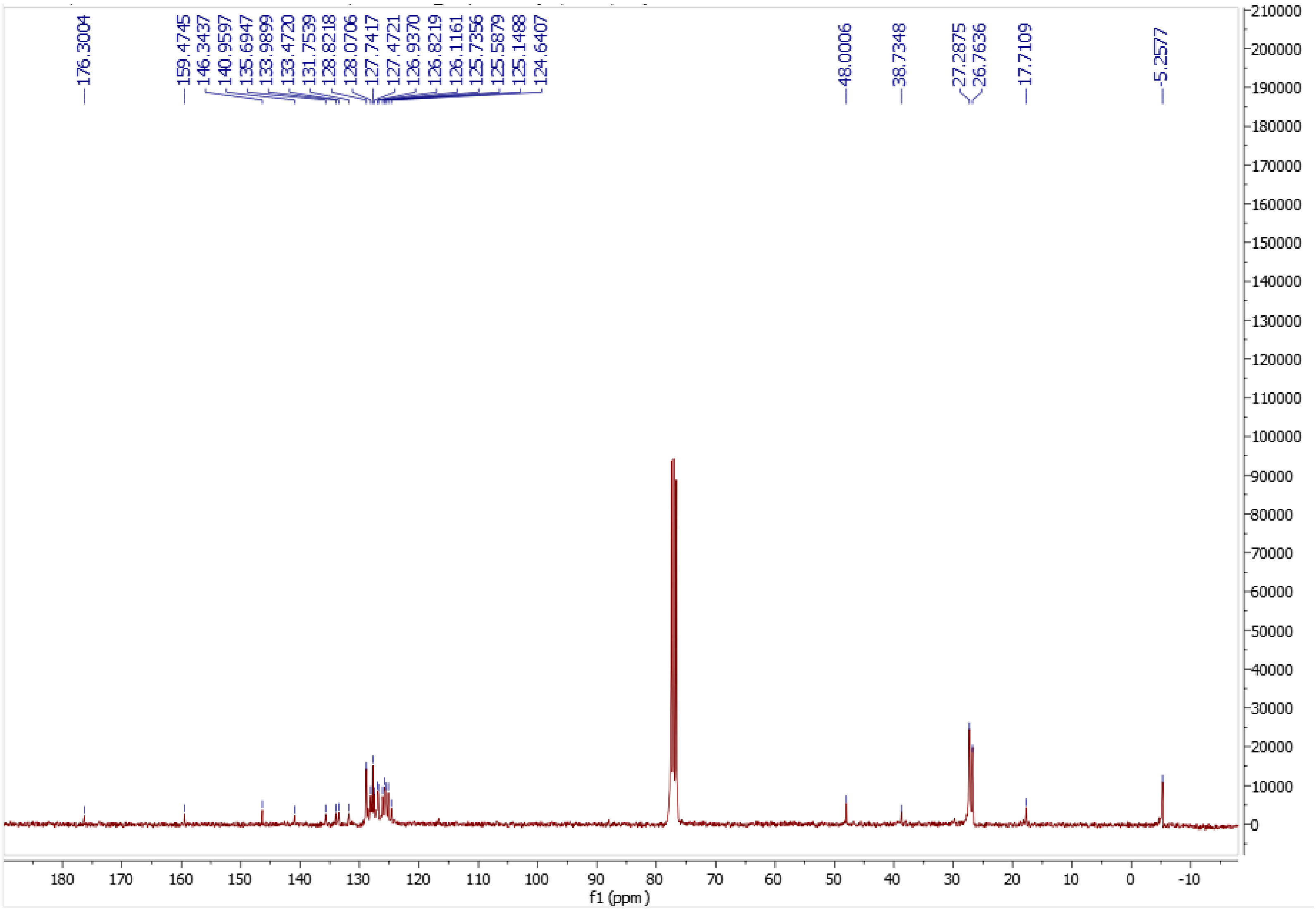

^{1}H NMR spectrum ($CDCl_3$, 300 MHz) of compound **4o**

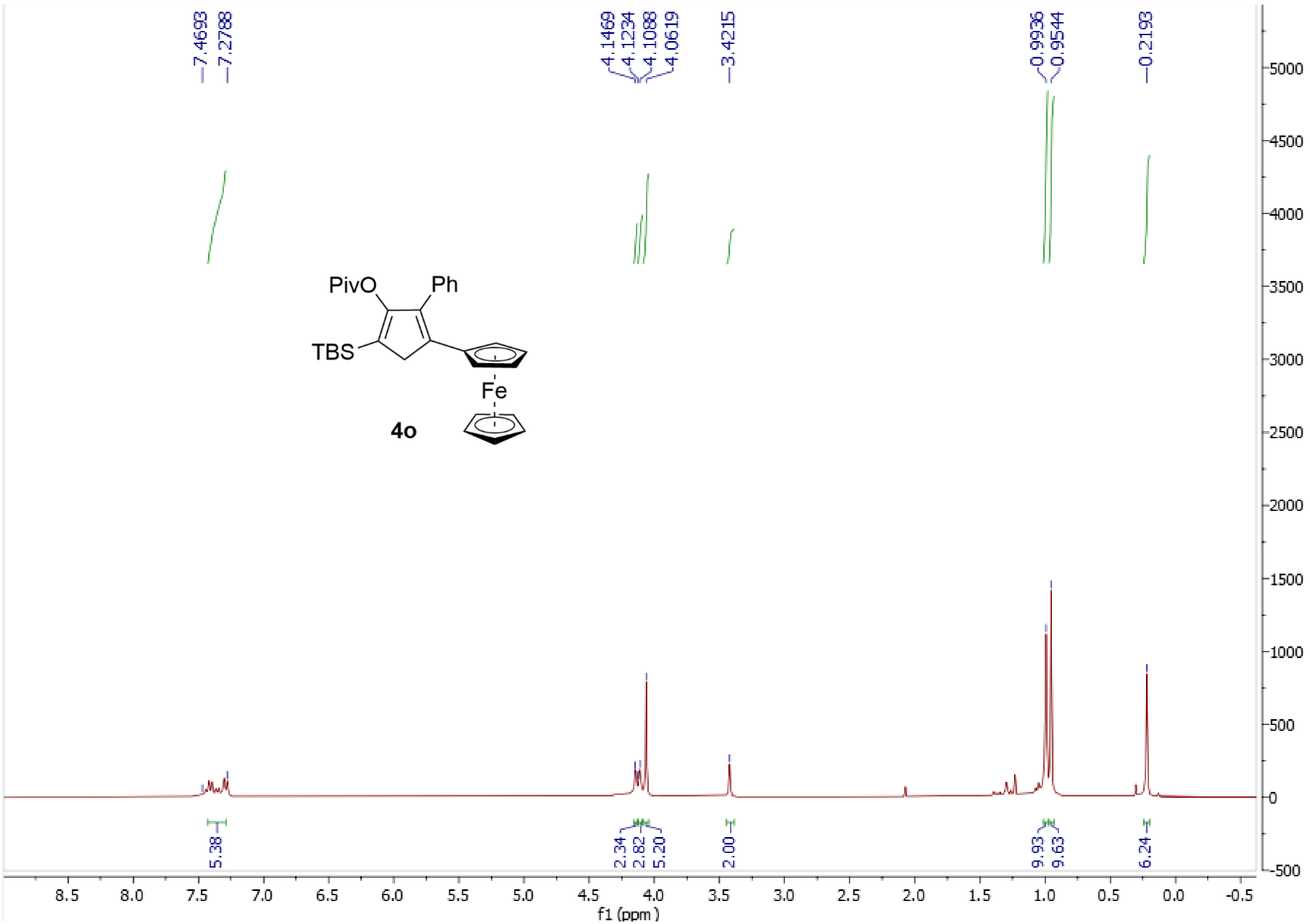

^{13}C NMR spectrum ($CDCl_3$, 75 MHz) of compound **4o**

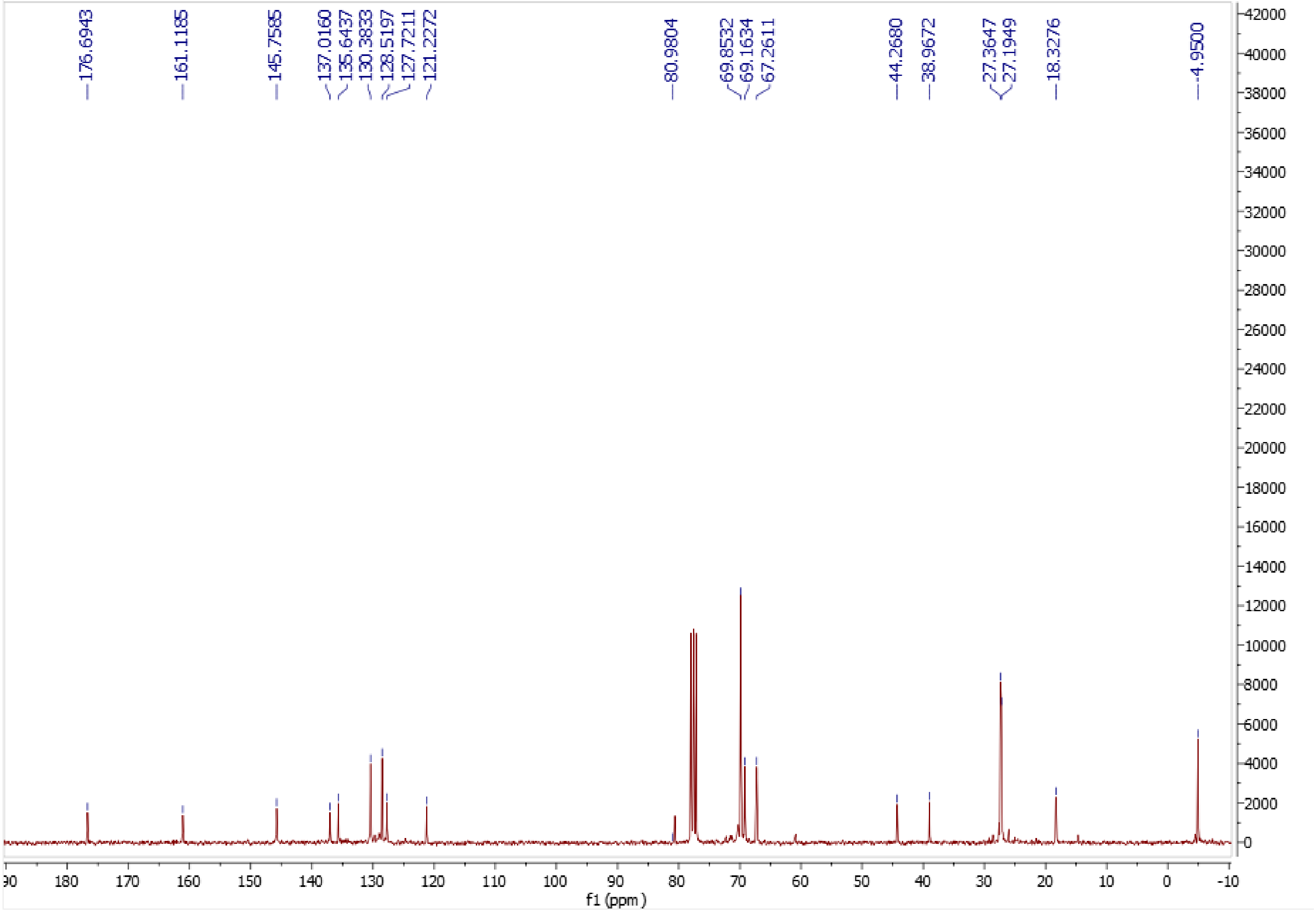

^{1}H NMR spectrum ($CDCl_3$, 300 MHz) of compound **4p** (obtained by Method A)

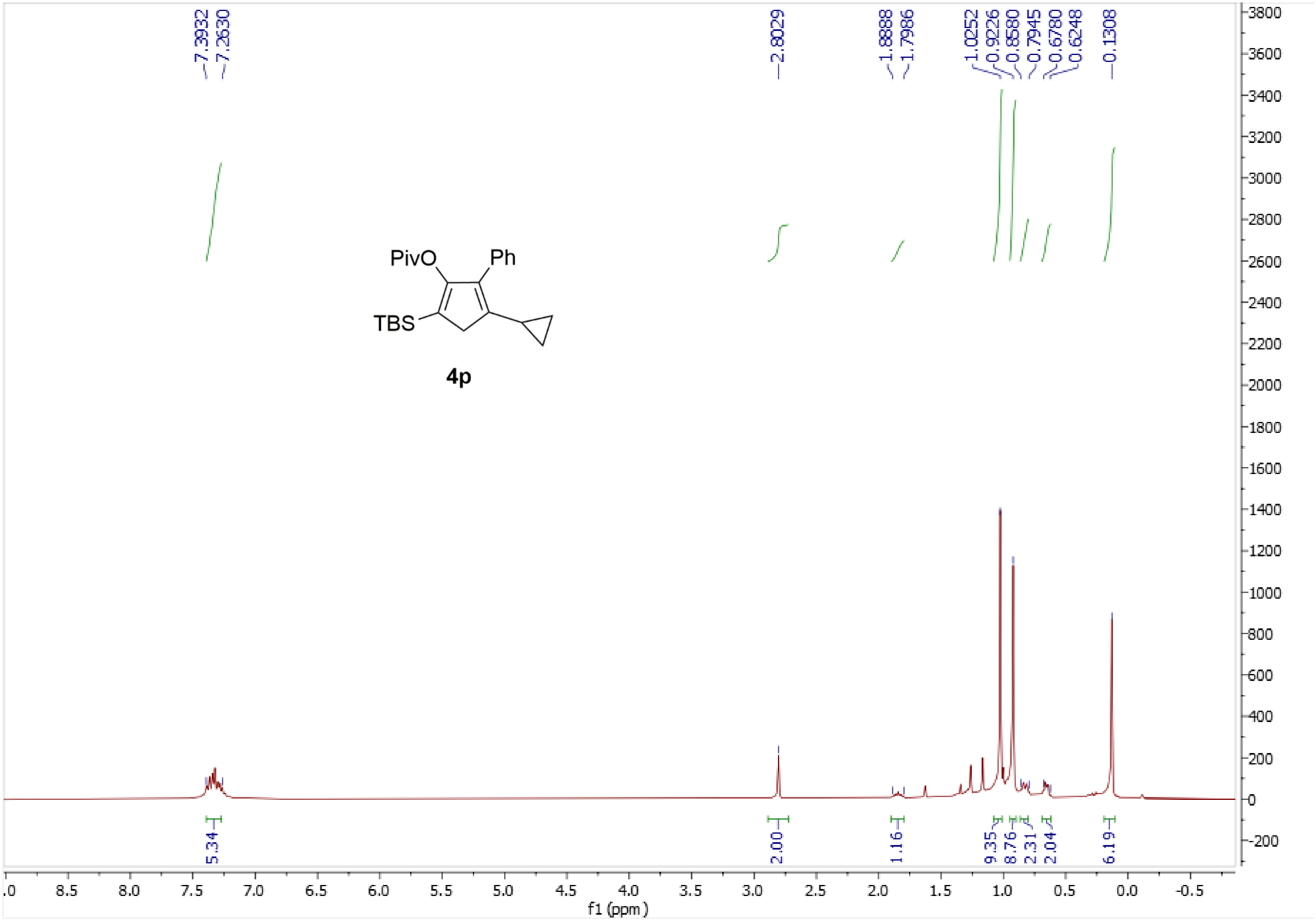

^{13}C NMR spectrum ($CDCl_3$, 75 MHz) of compound **4p** (obtained by Method A)

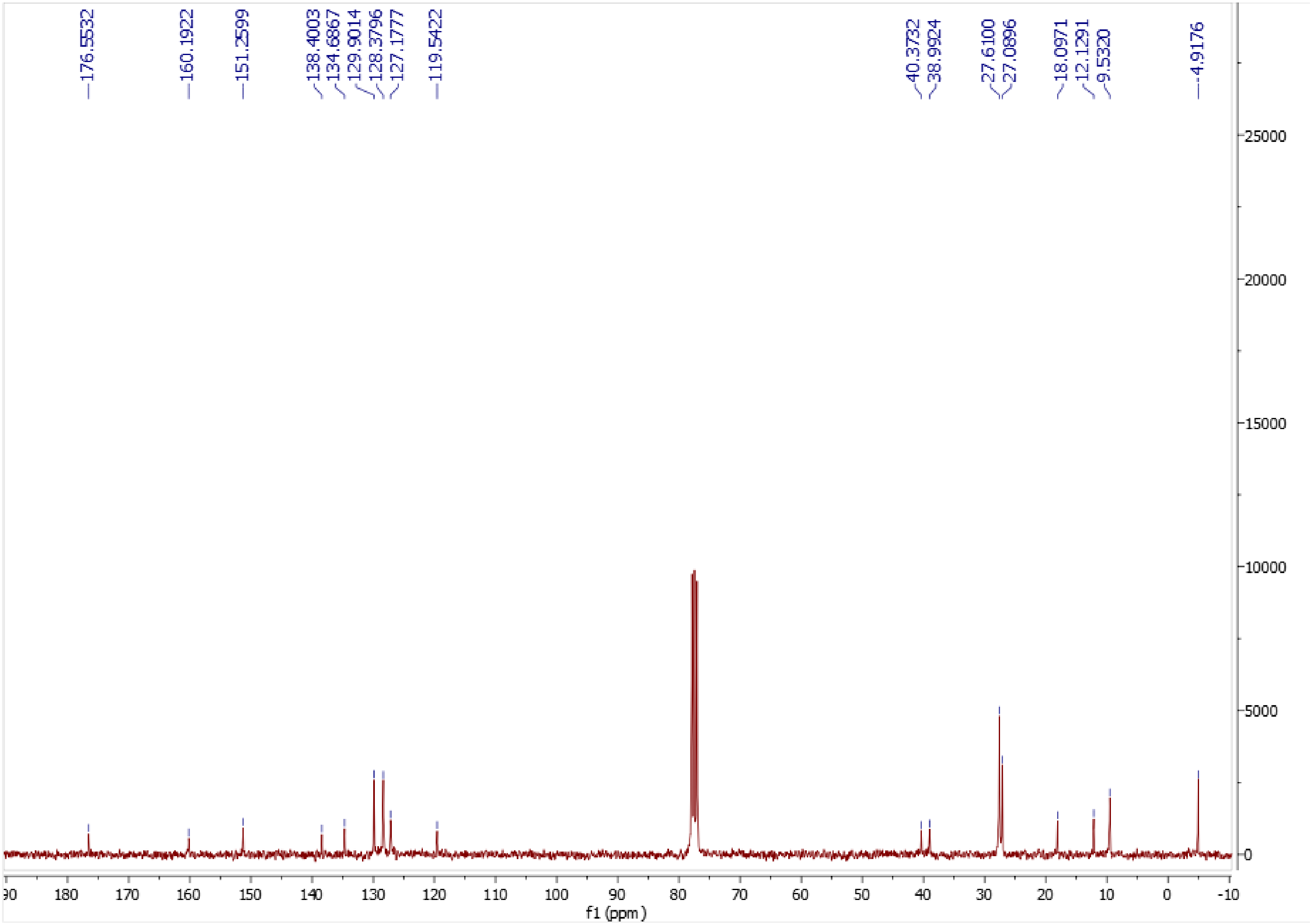

^{1}H NMR spectrum ($CDCl_3$, 300 MHz) of compound **4p** (obtained by Method B)

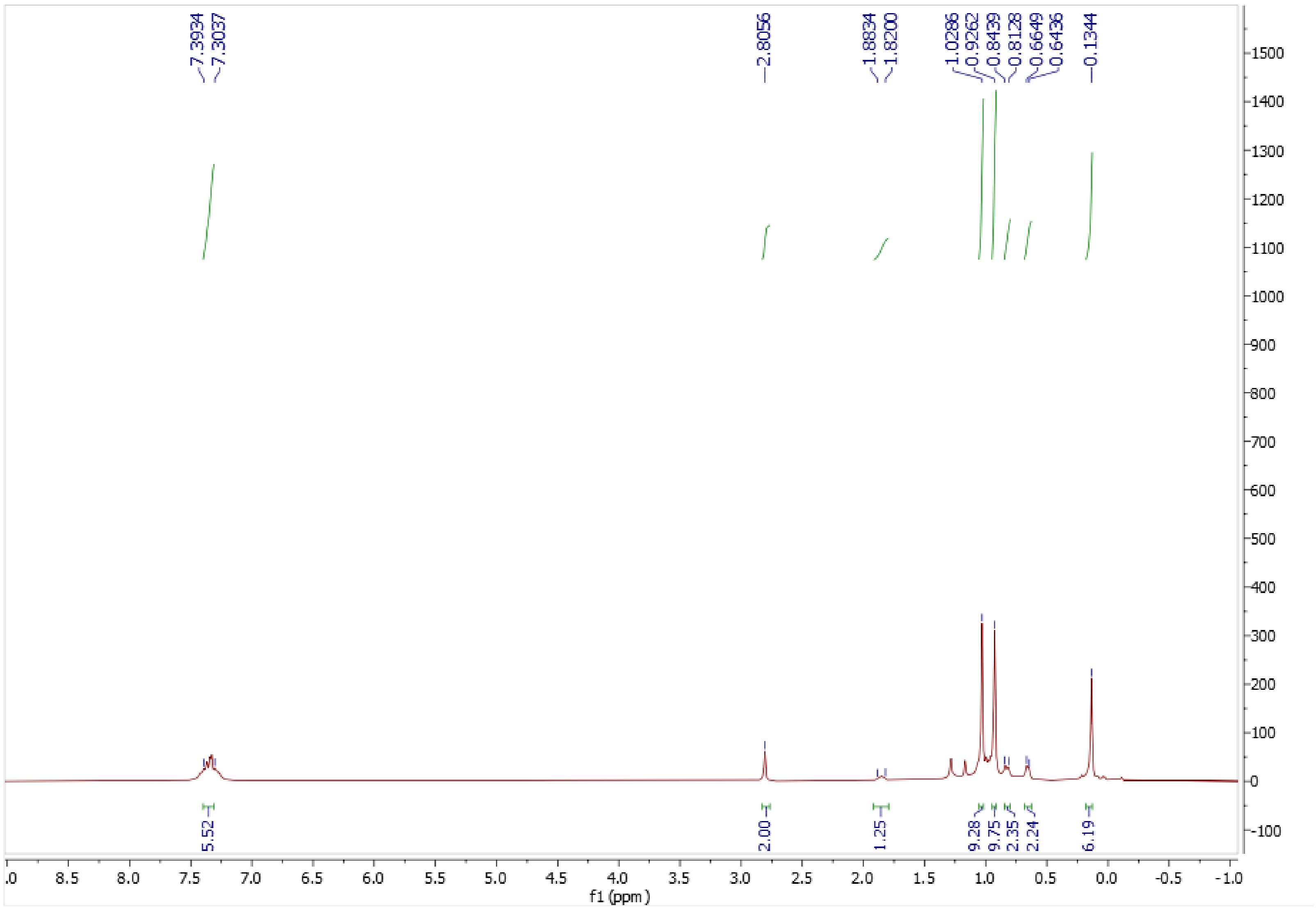

^{1}H NMR spectrum ($CDCl_3$, 300 MHz) of compound **4q** (obtained by Method A)

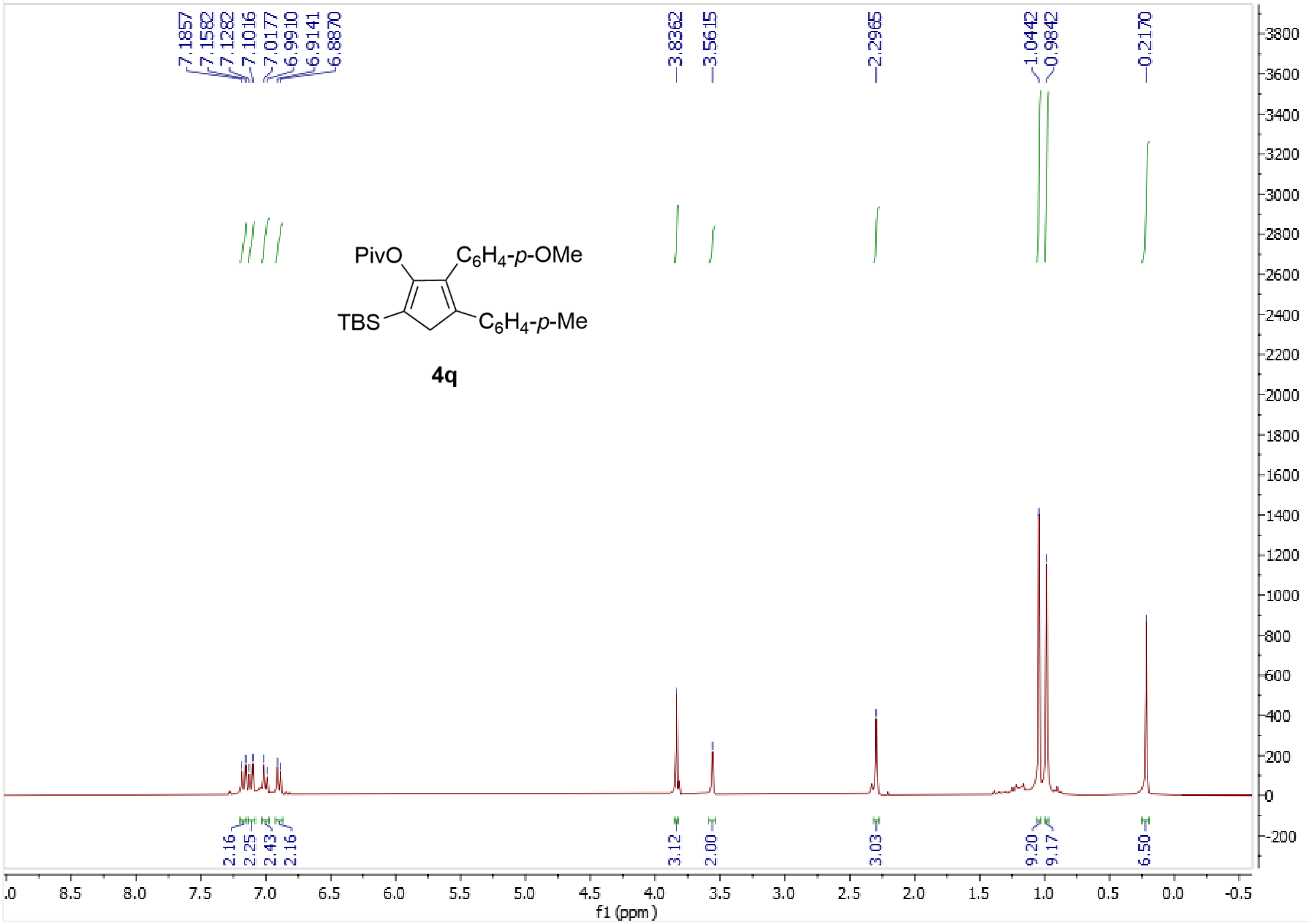

^{13}C NMR spectrum ($CDCl_3$, 75 MHz) of compound **4q** (obtained by Method A)

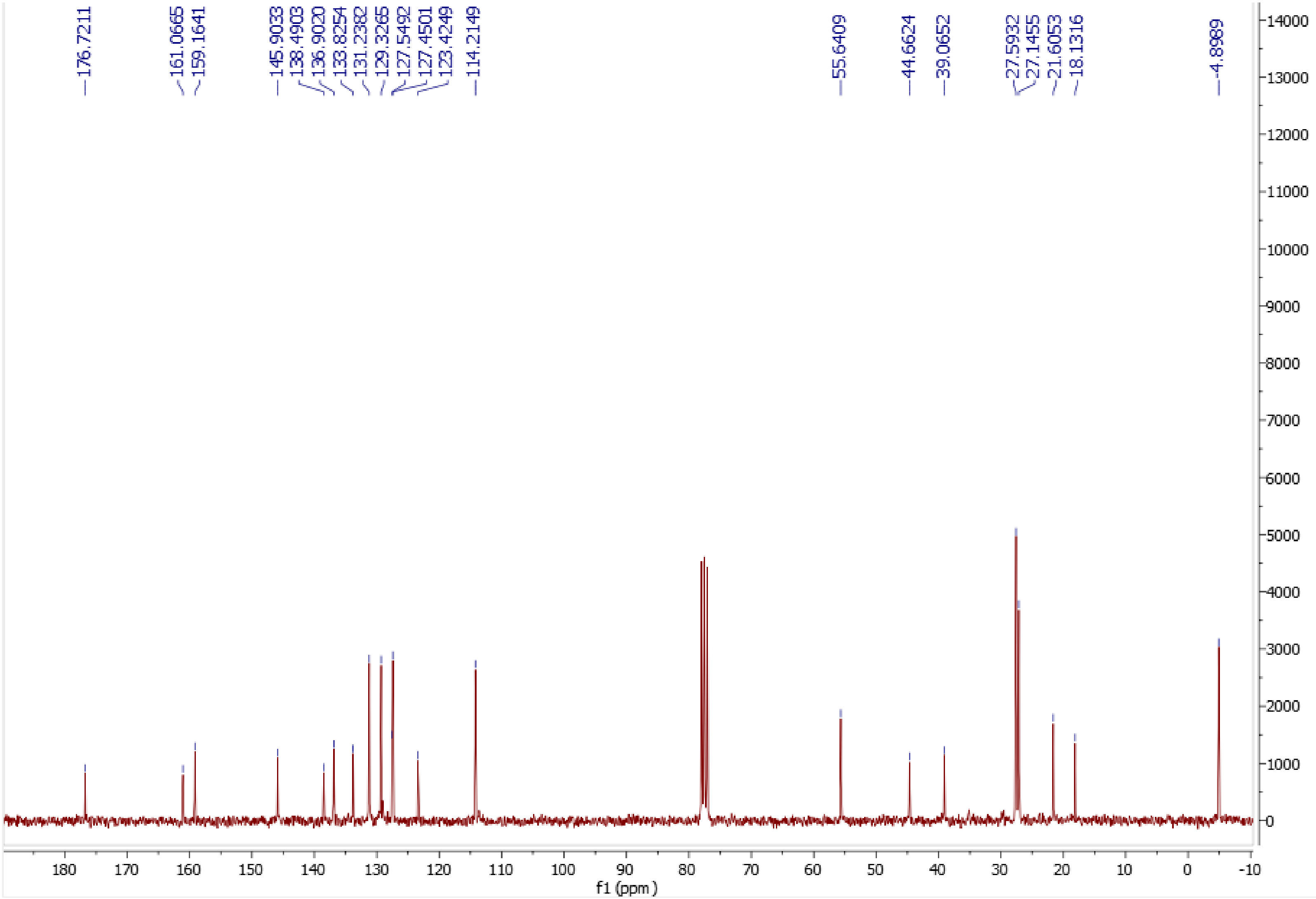

^{1}H NMR spectrum ($CDCl_3$, 300 MHz) of compound **4q** (obtained by Method B)

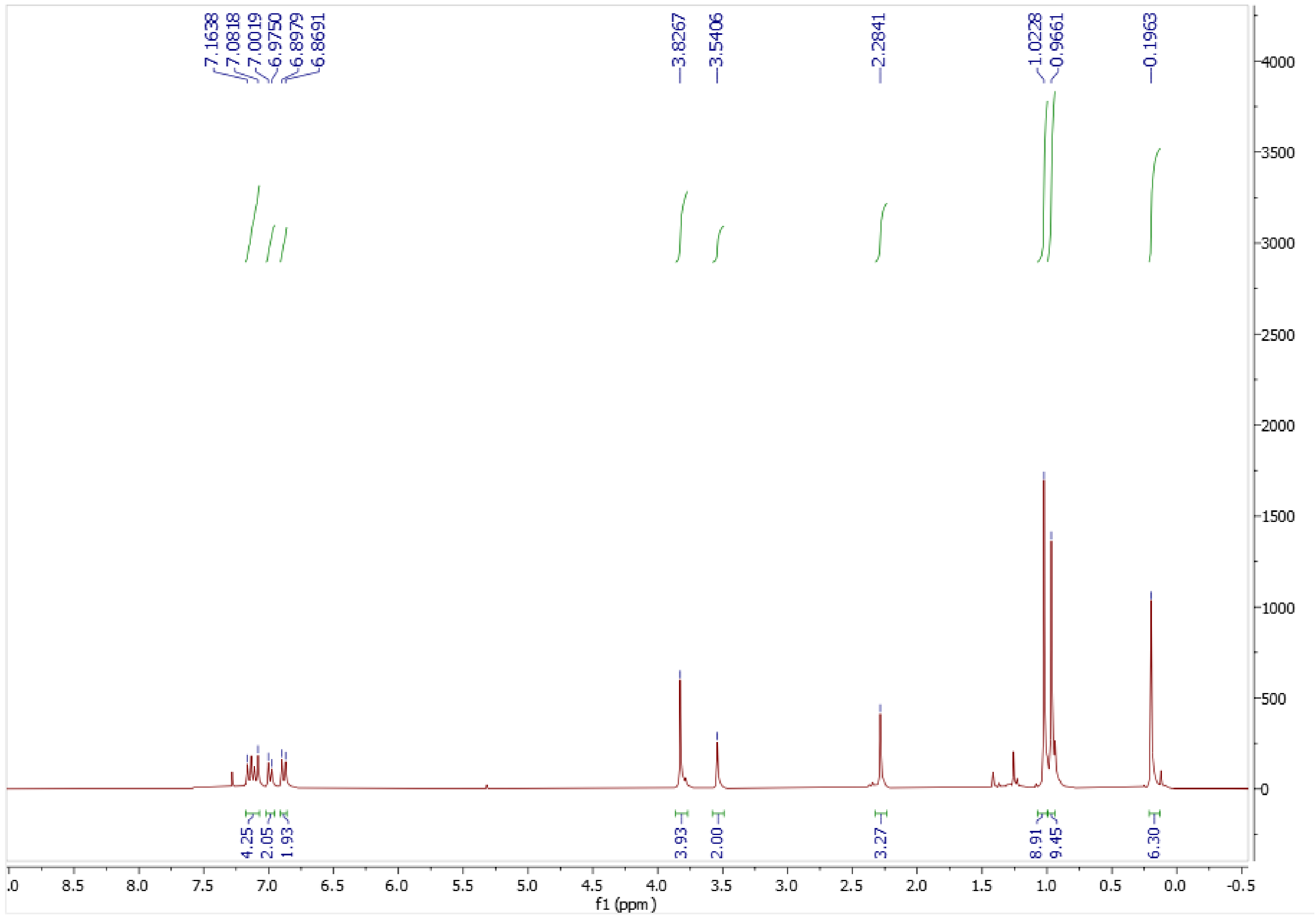

^{1}H NMR spectrum ($CDCl_3$, 300 MHz) of compound **4r** (obtained by Method A)

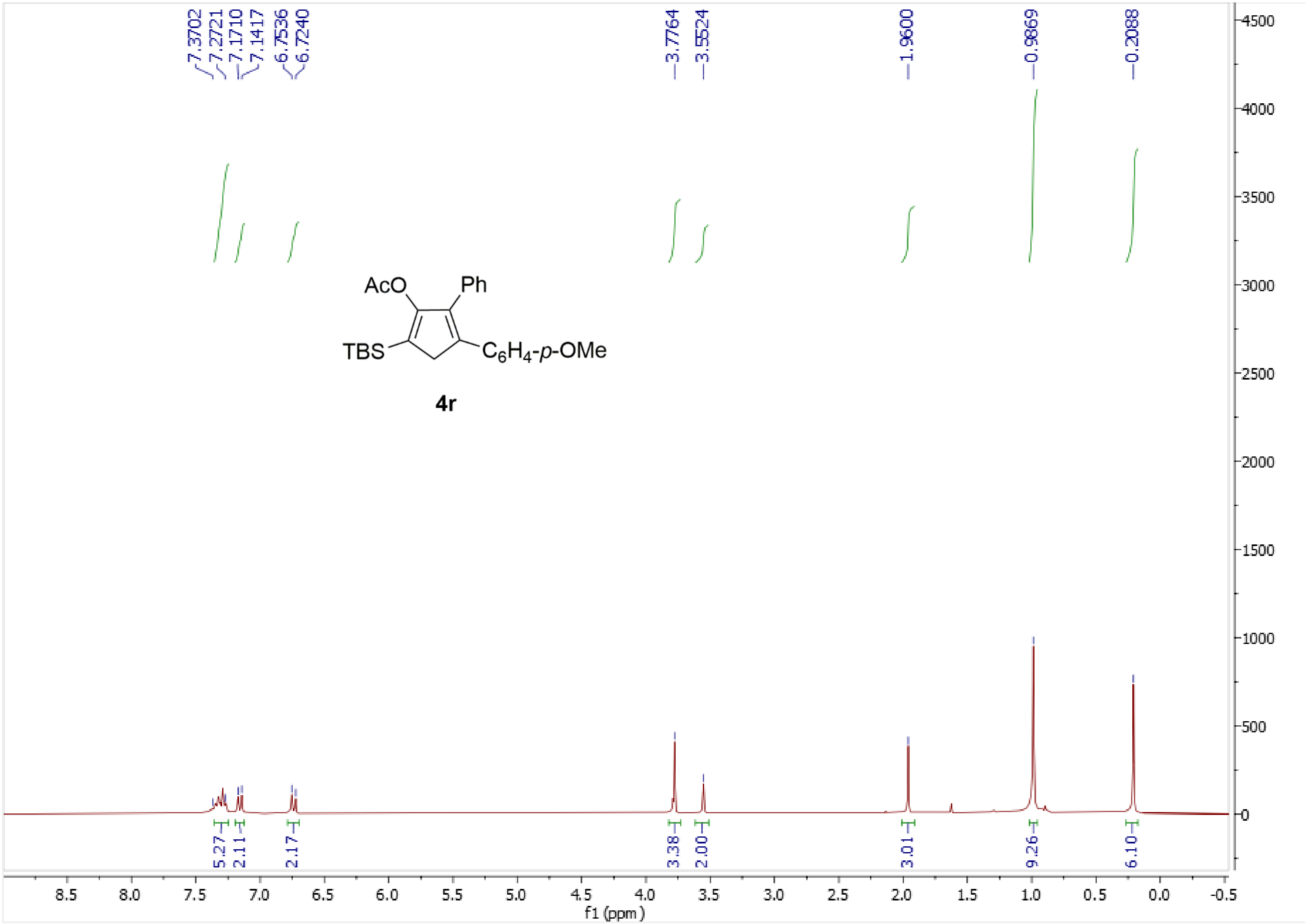

^{13}C NMR spectrum ($CDCl_3$, 75 MHz) of compound **4r** (obtained by Method A)

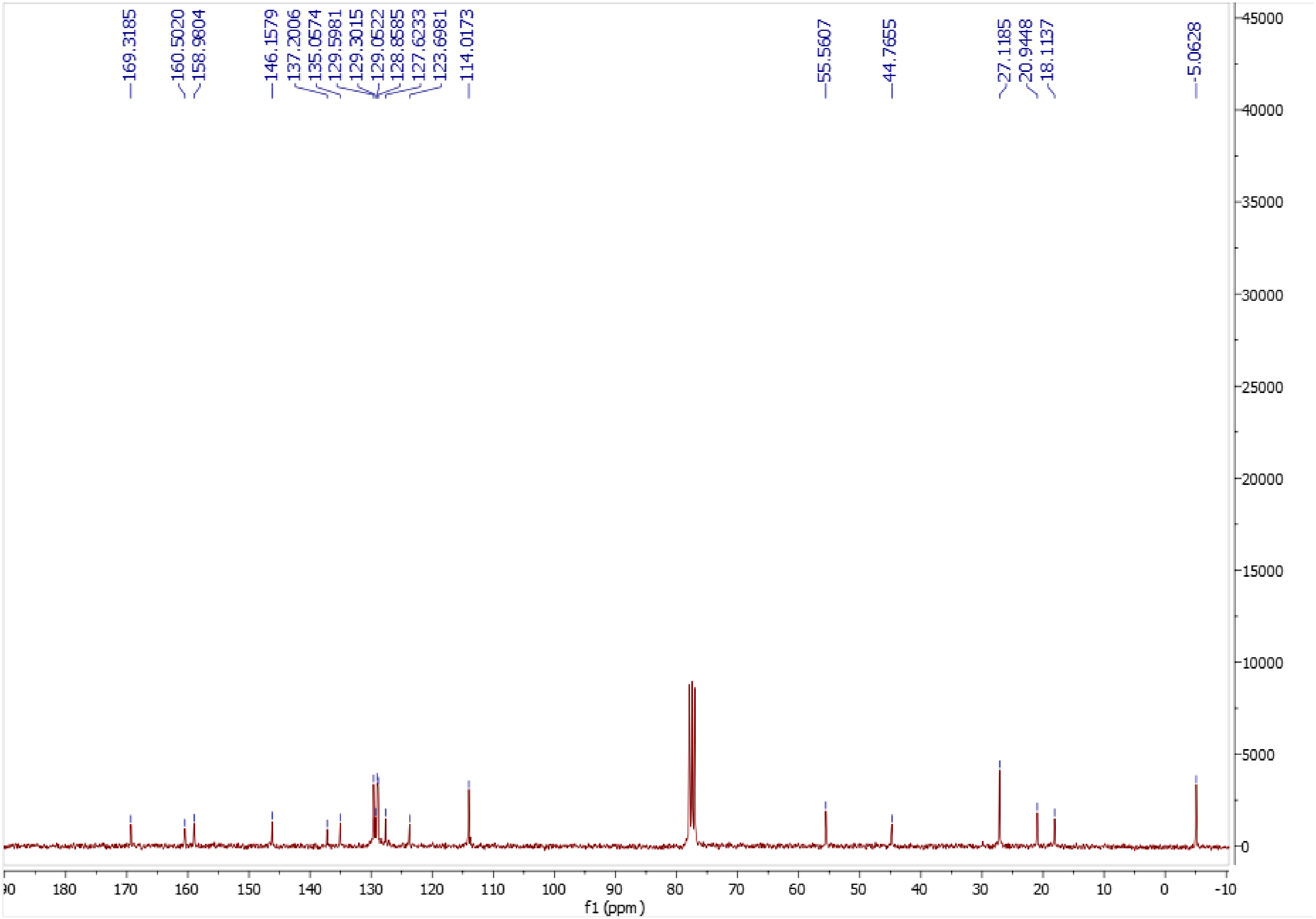

^{1}H NMR spectrum ($CDCl_3$, 300 MHz) of compound **4r** (obtained by Method B)

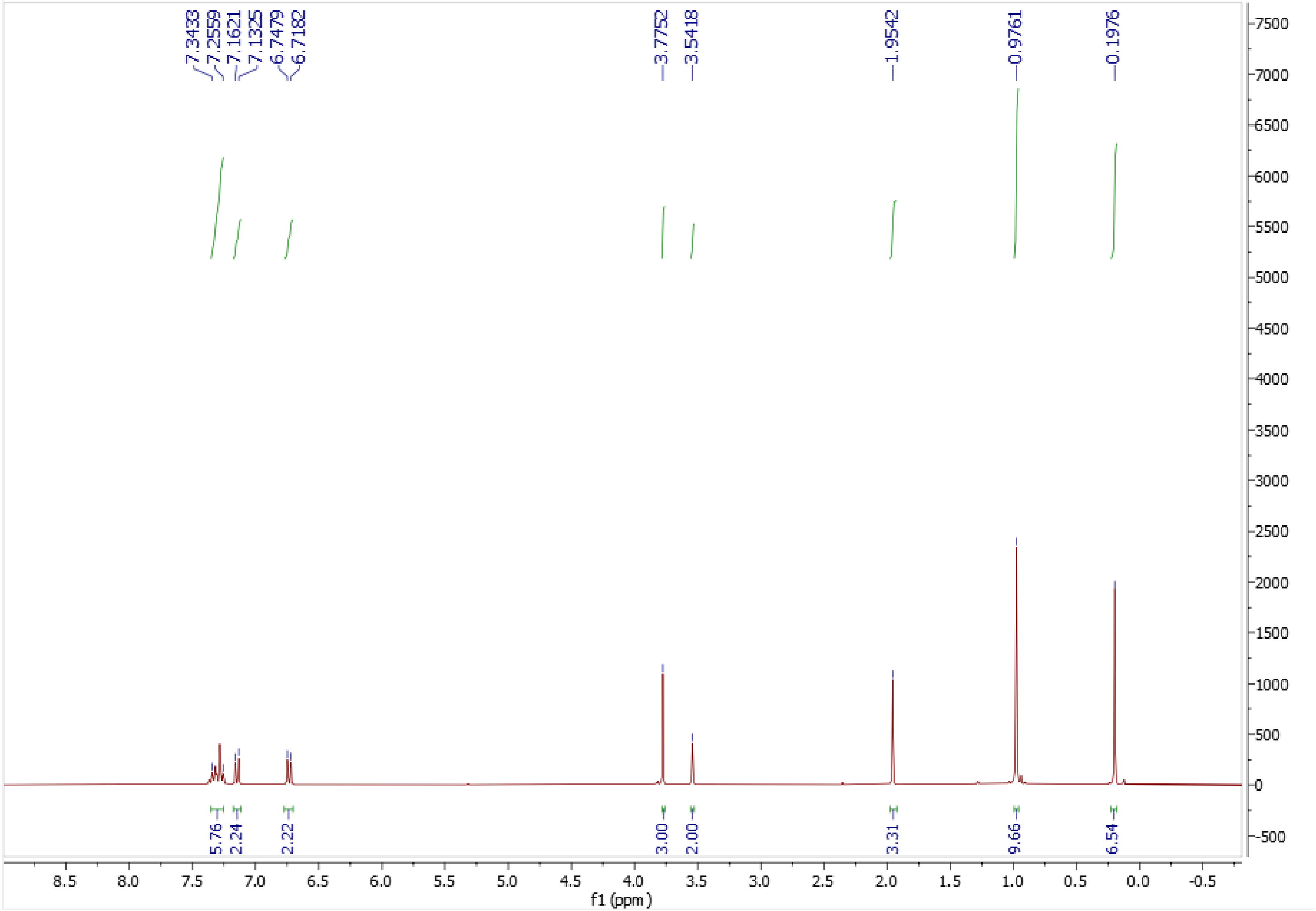

^{1}H NMR spectrum ($CDCl_3$, 300 MHz) of compound **4s**

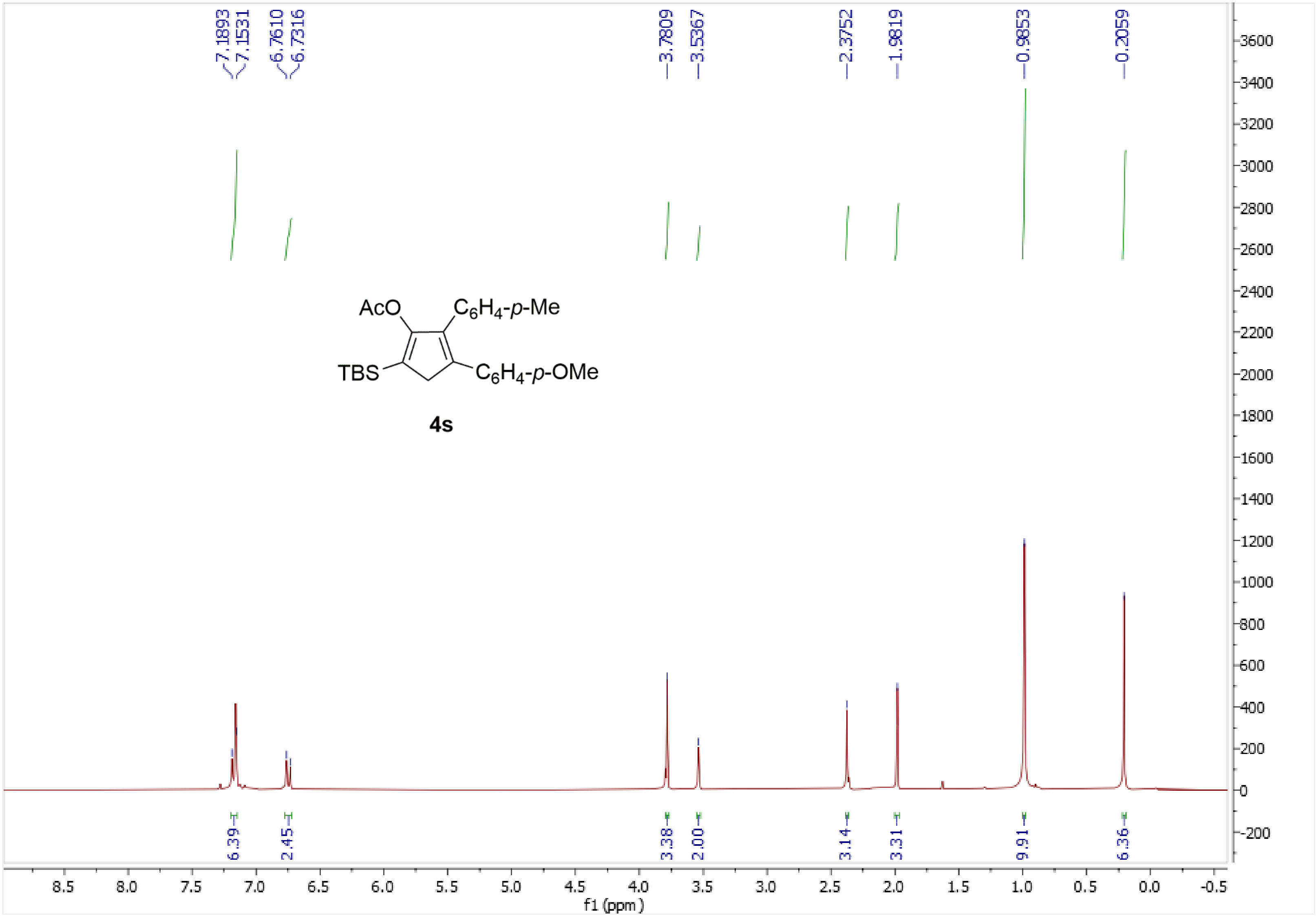

^{13}C NMR spectrum ($CDCl_3$, 75 MHz) of compound **4s**

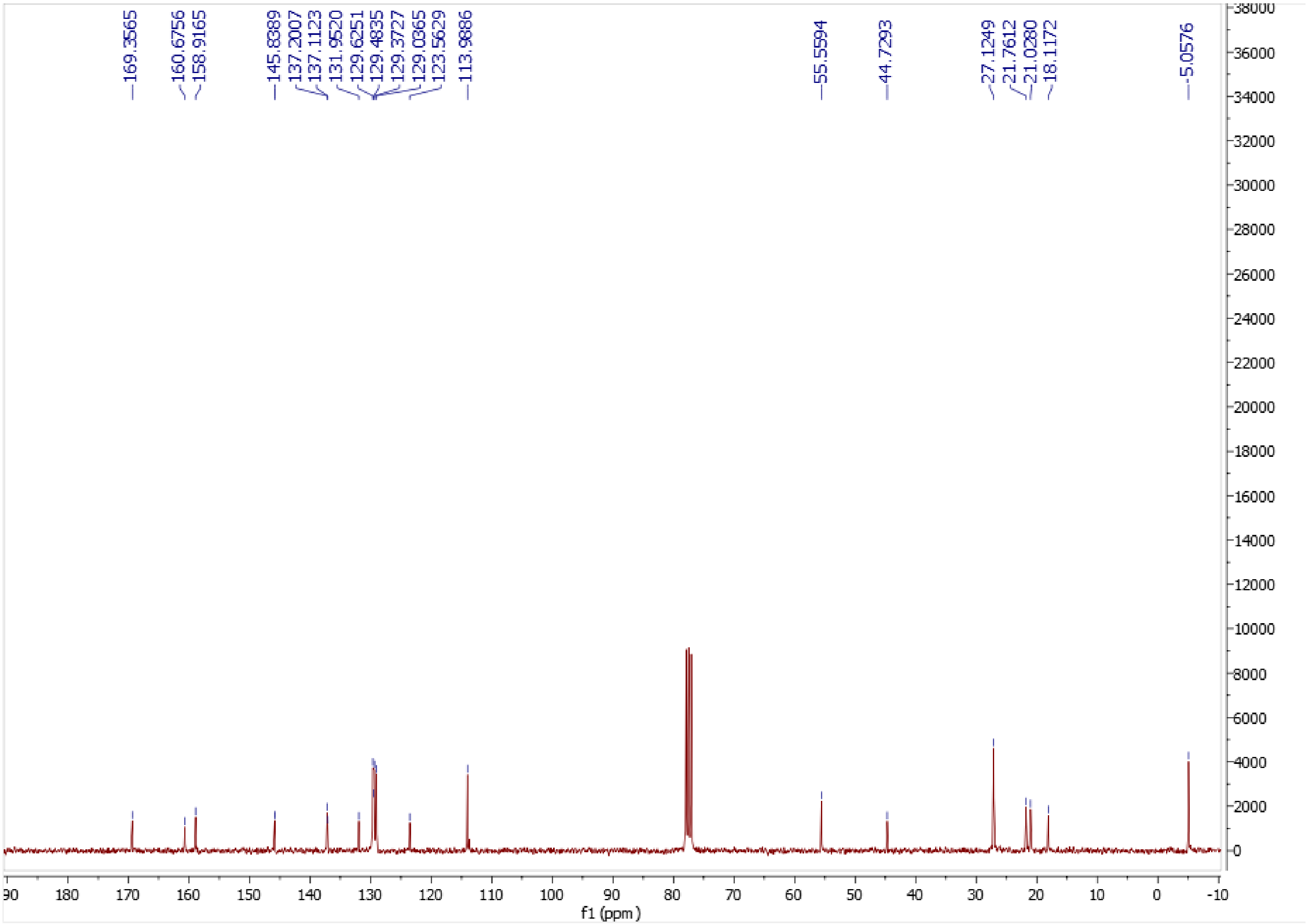

^{1}H NMR spectrum ($CDCl_3$, 300 MHz) of compound **4t**

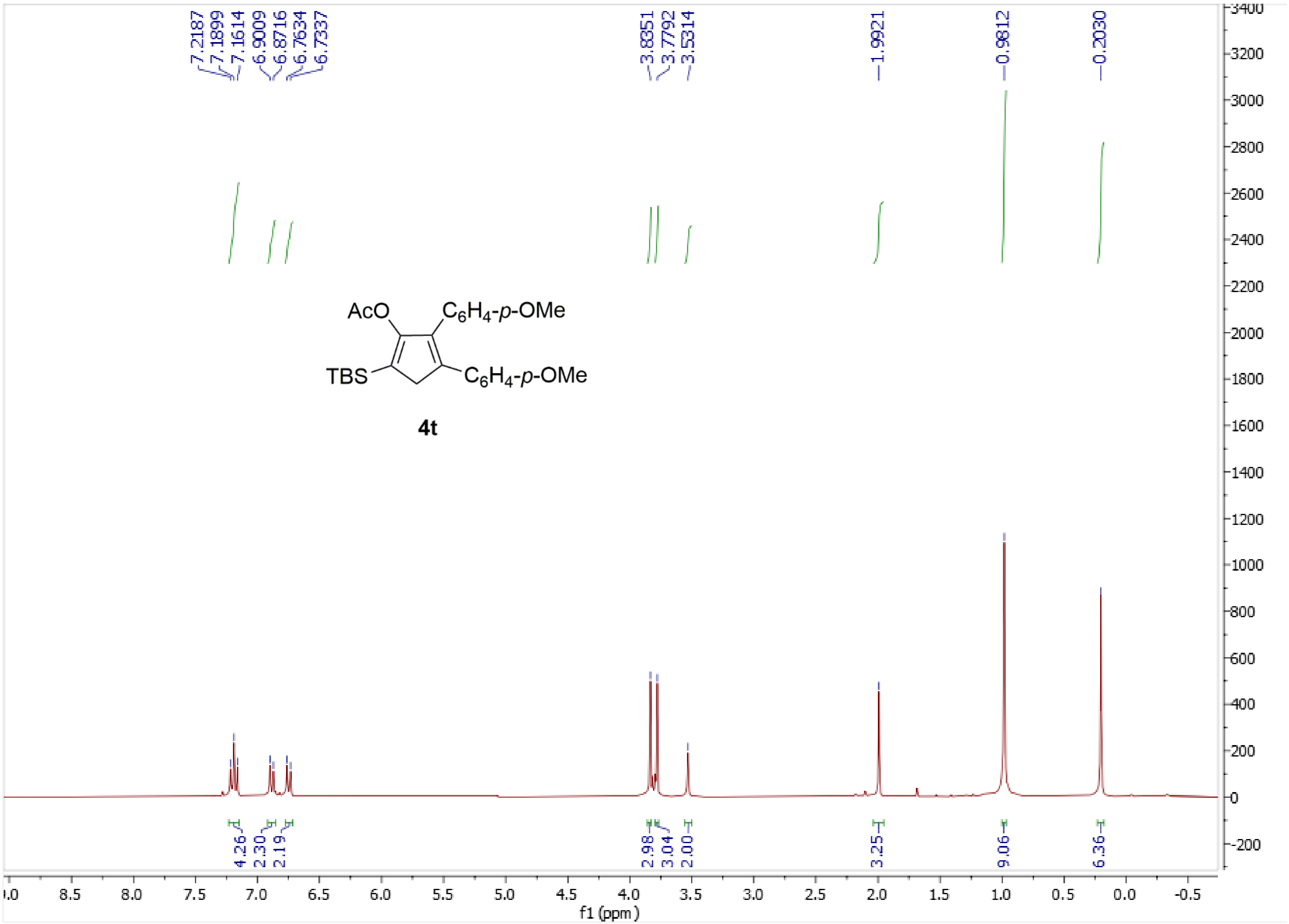

^{13}C NMR spectrum ($CDCl_3$, 75 MHz) of compound **4t**

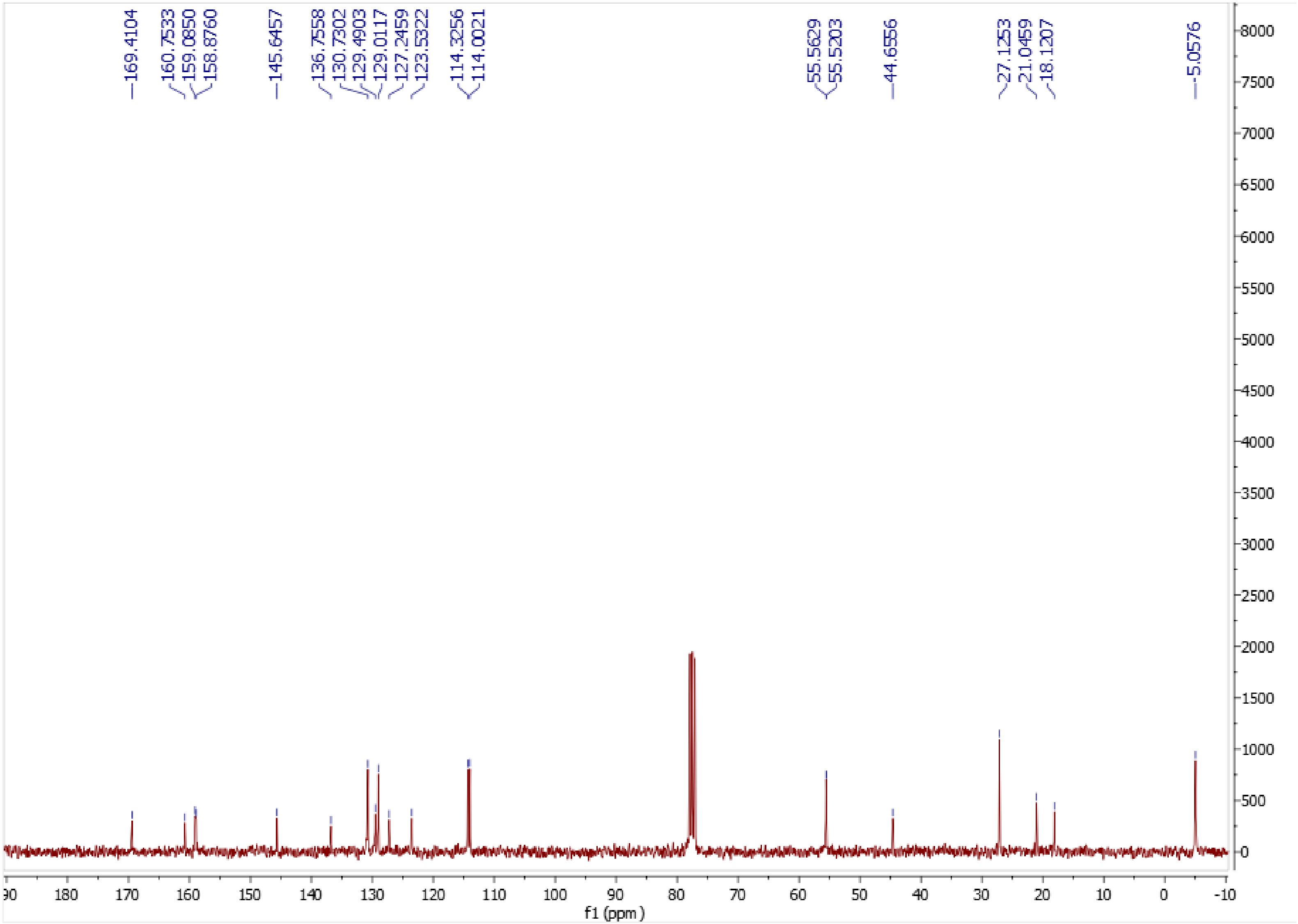

^{1}H NMR spectrum ($CDCl_3$, 300 MHz) of compound **4u**

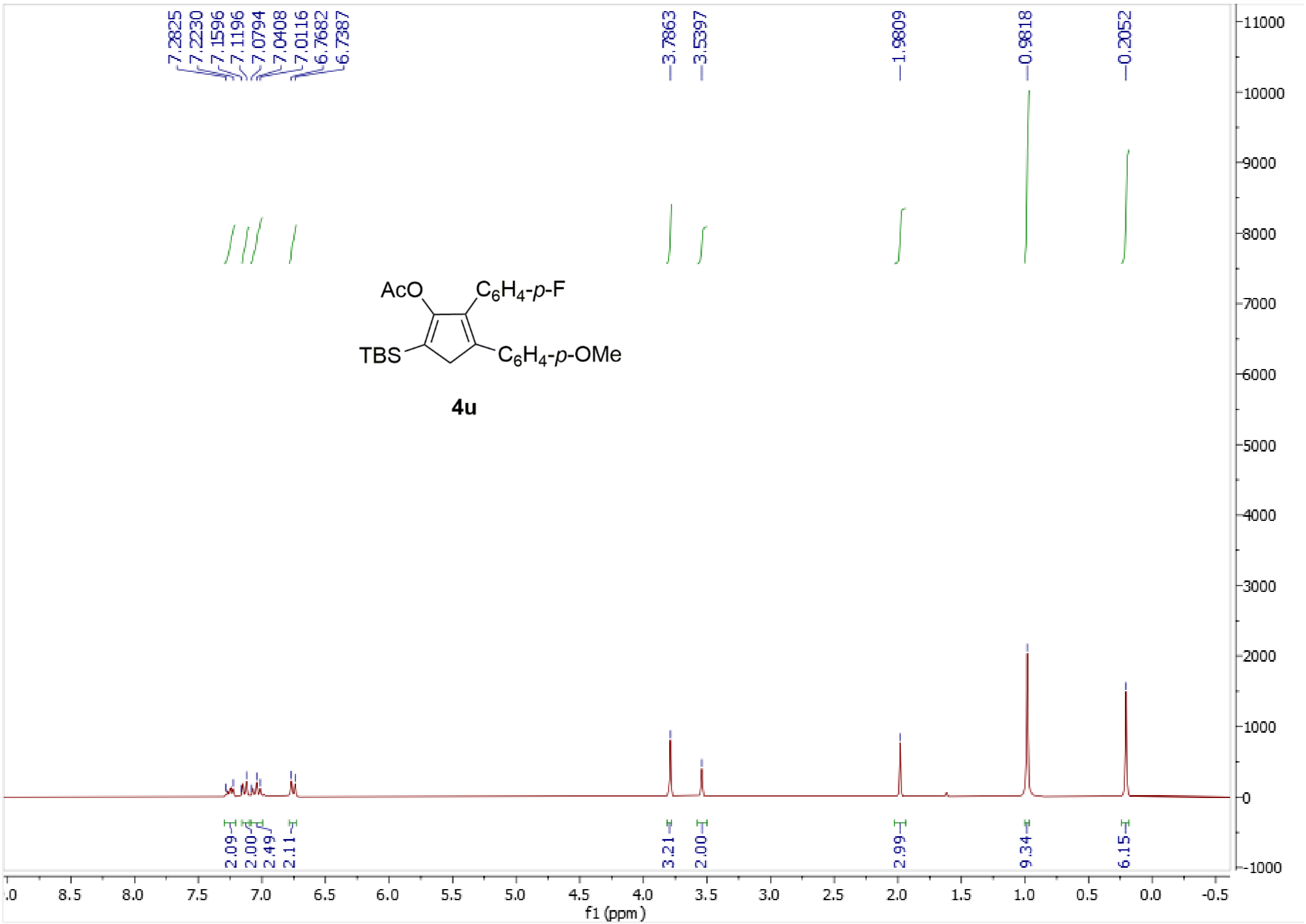

^{13}C NMR spectrum ($CDCl_3$, 75 MHz) of compound **4u**

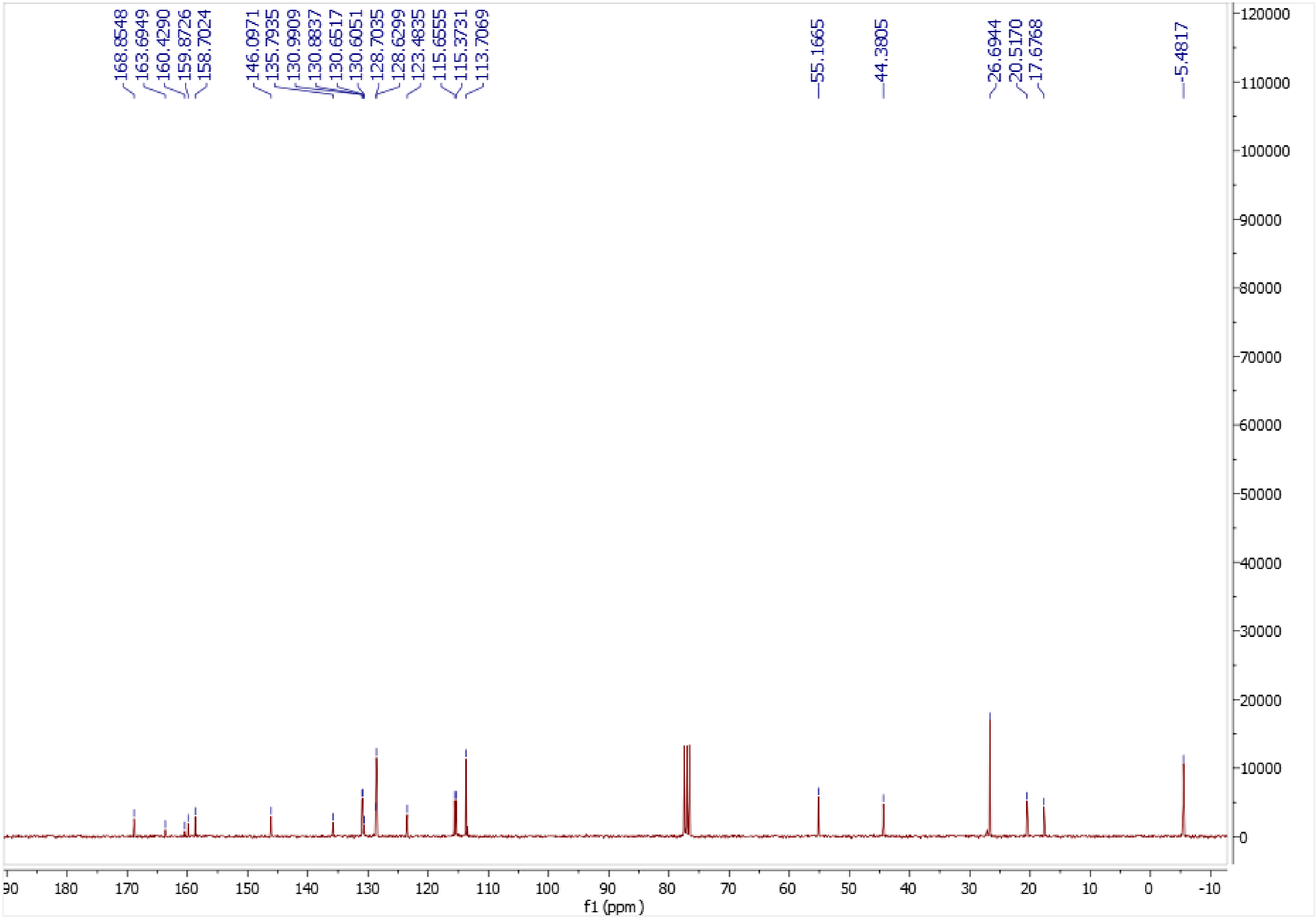

^{19}F NMR spectrum ($CDCl_3$, 282 MHz) of compound **4u**

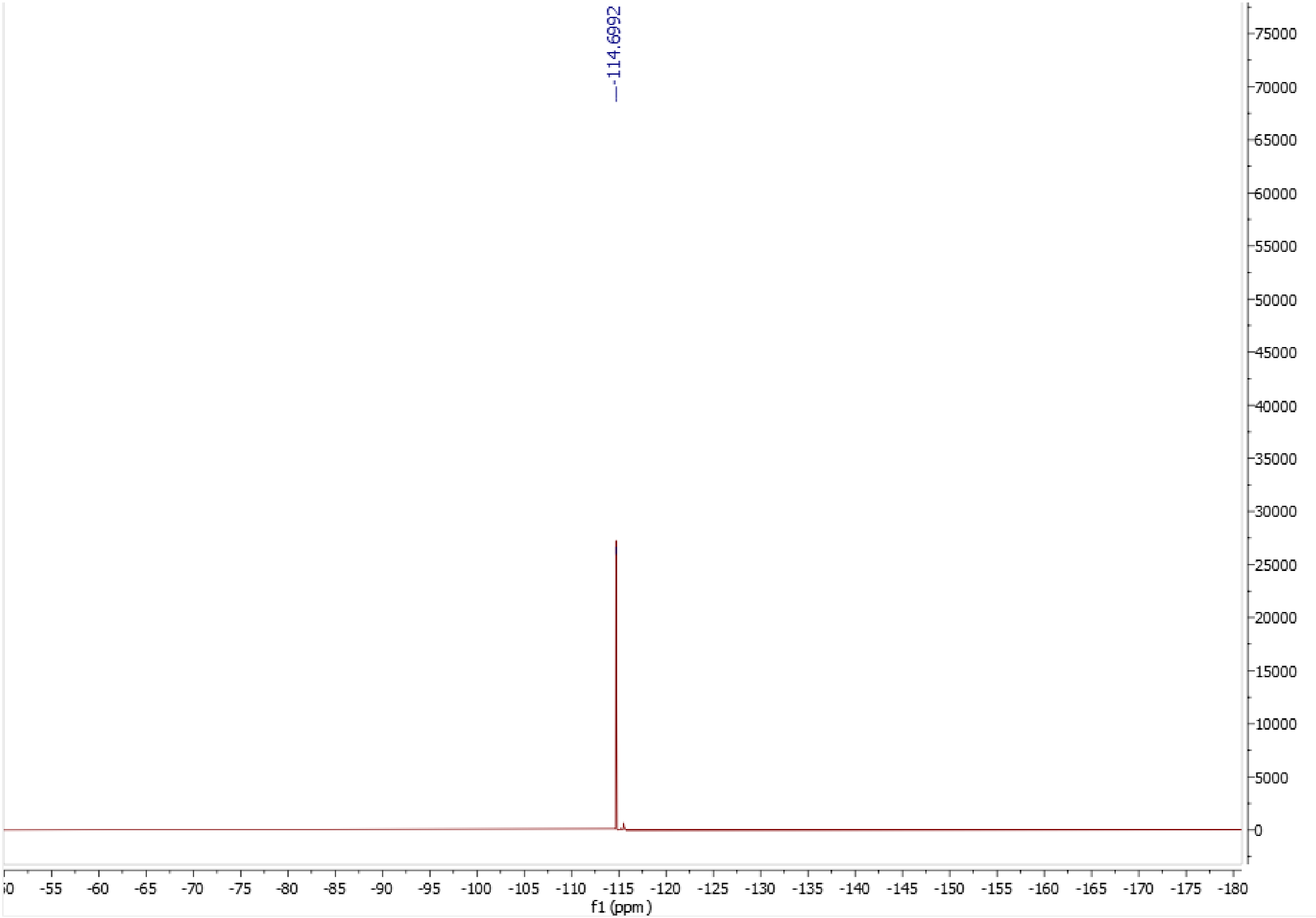

^{1}H NMR spectrum ($CDCl_3$, 300 MHz) of compound **5a**

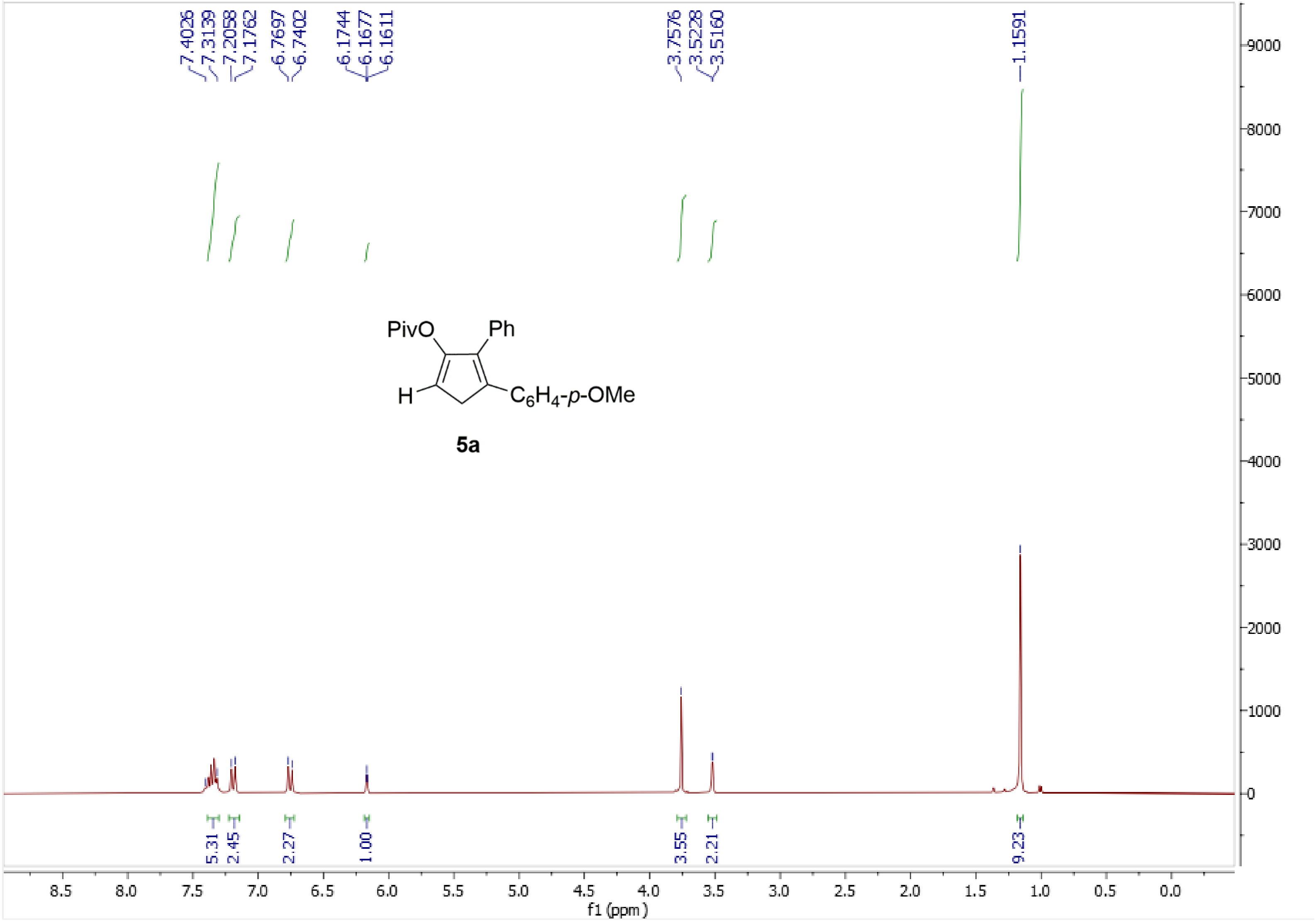

^{13}C NMR spectrum ($CDCl_3$, 75 MHz) of compound **5a**

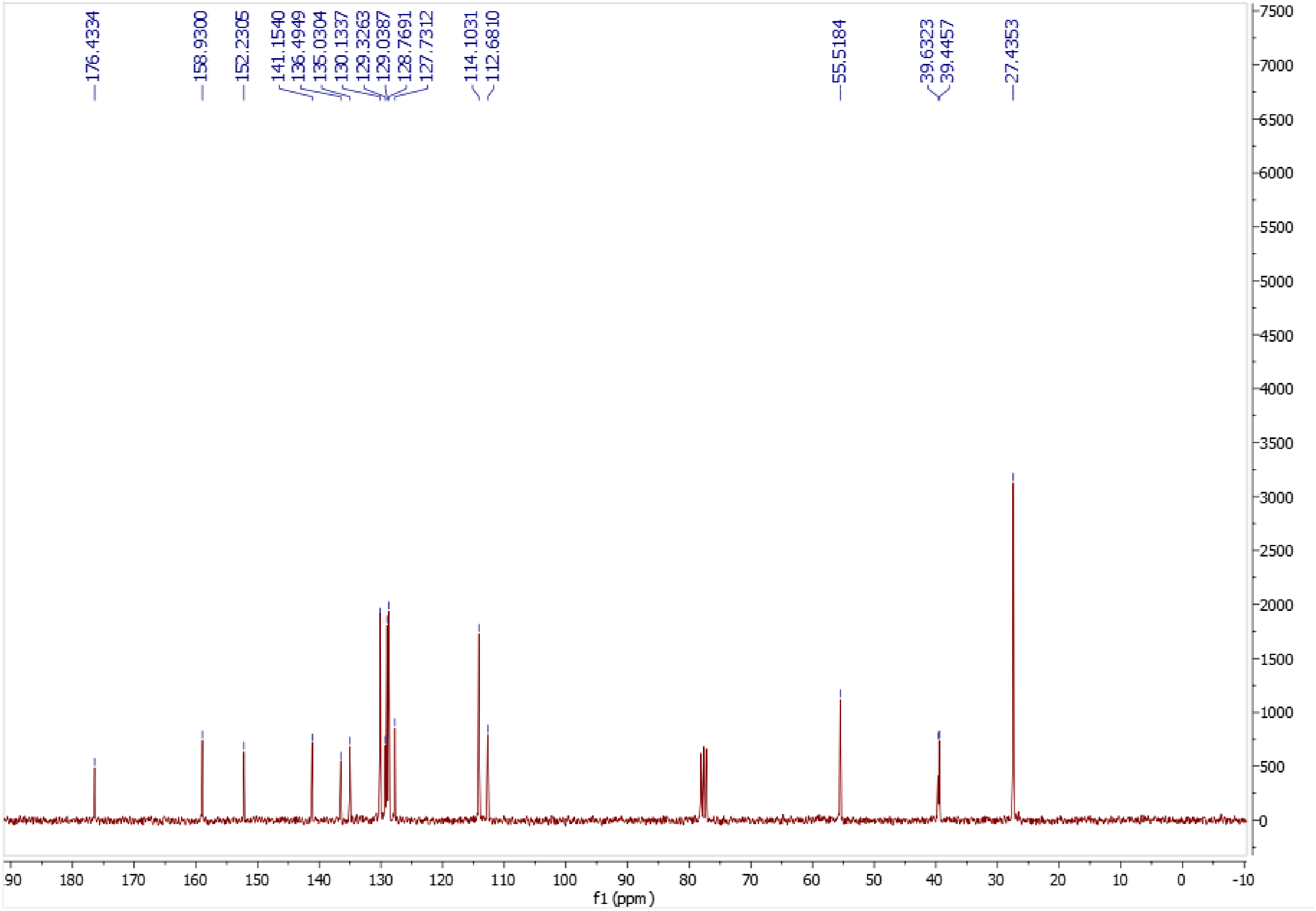

^1H NMR spectrum ($CDCl_3$, 300 MHz) of compound **6a**

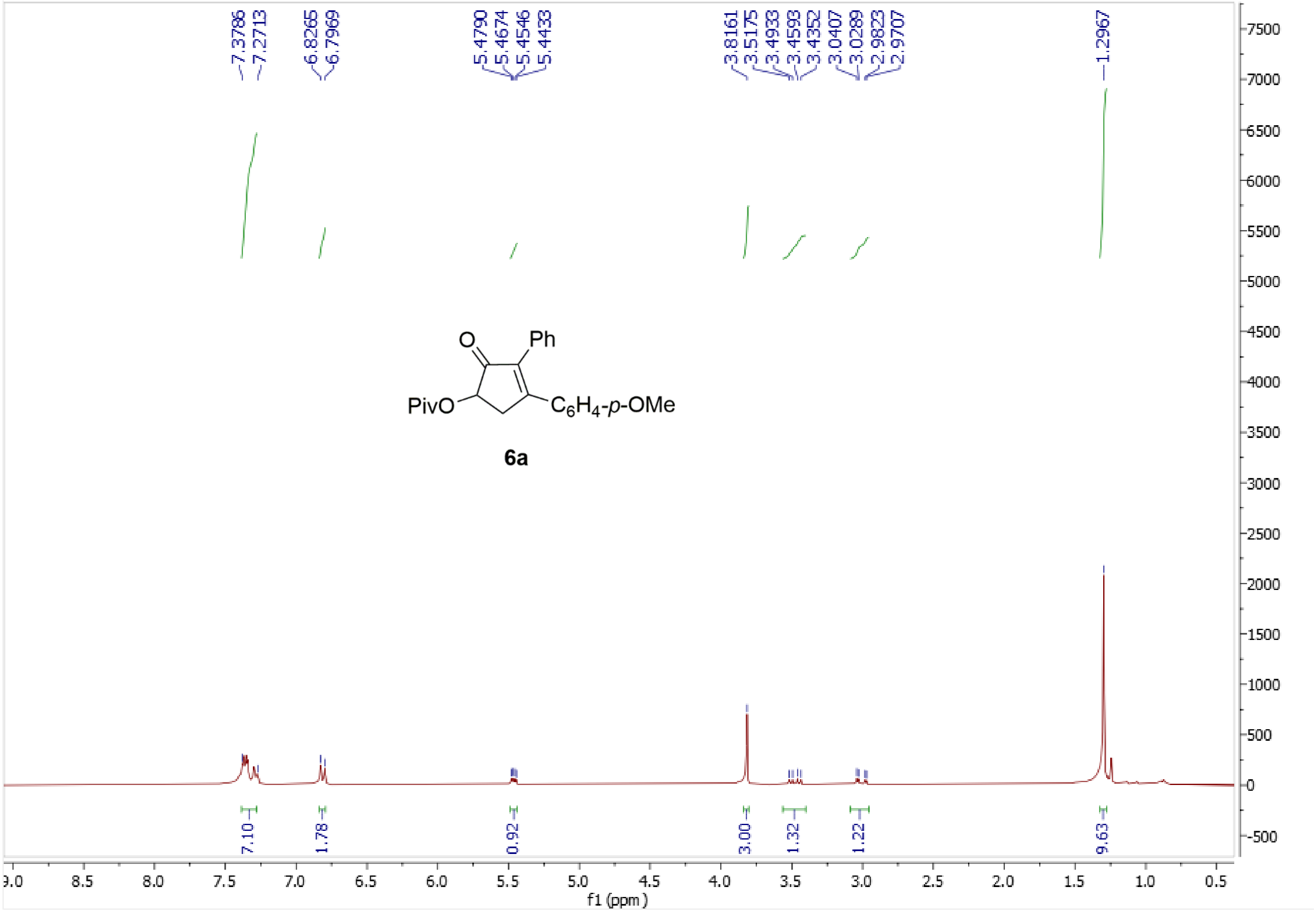

^{13}C NMR spectrum ($CDCl_3$, 75 MHz) of compound **6a**

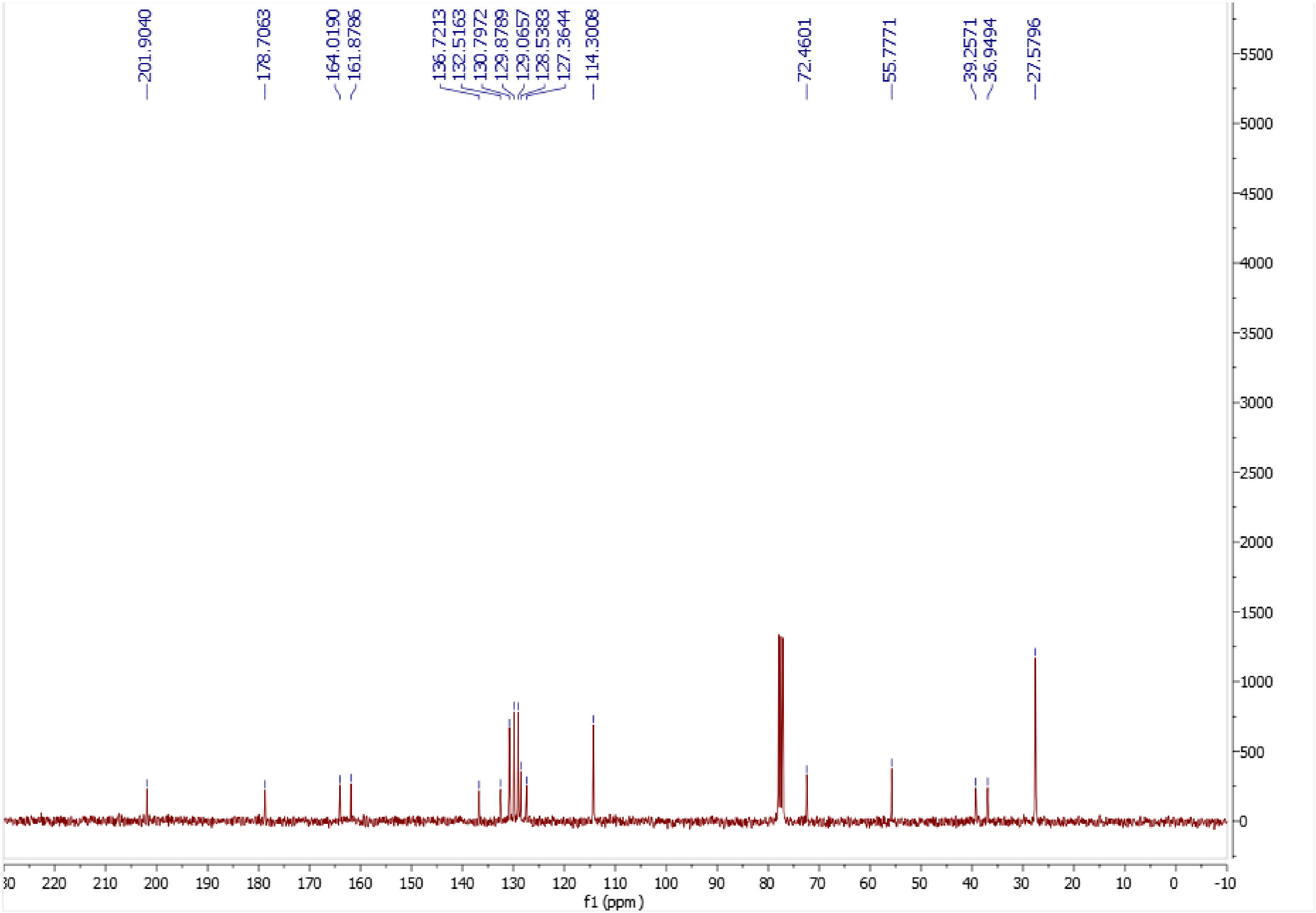

^{1}H NMR spectrum ($CDCl_3$, 300 MHz) of compound **7a**

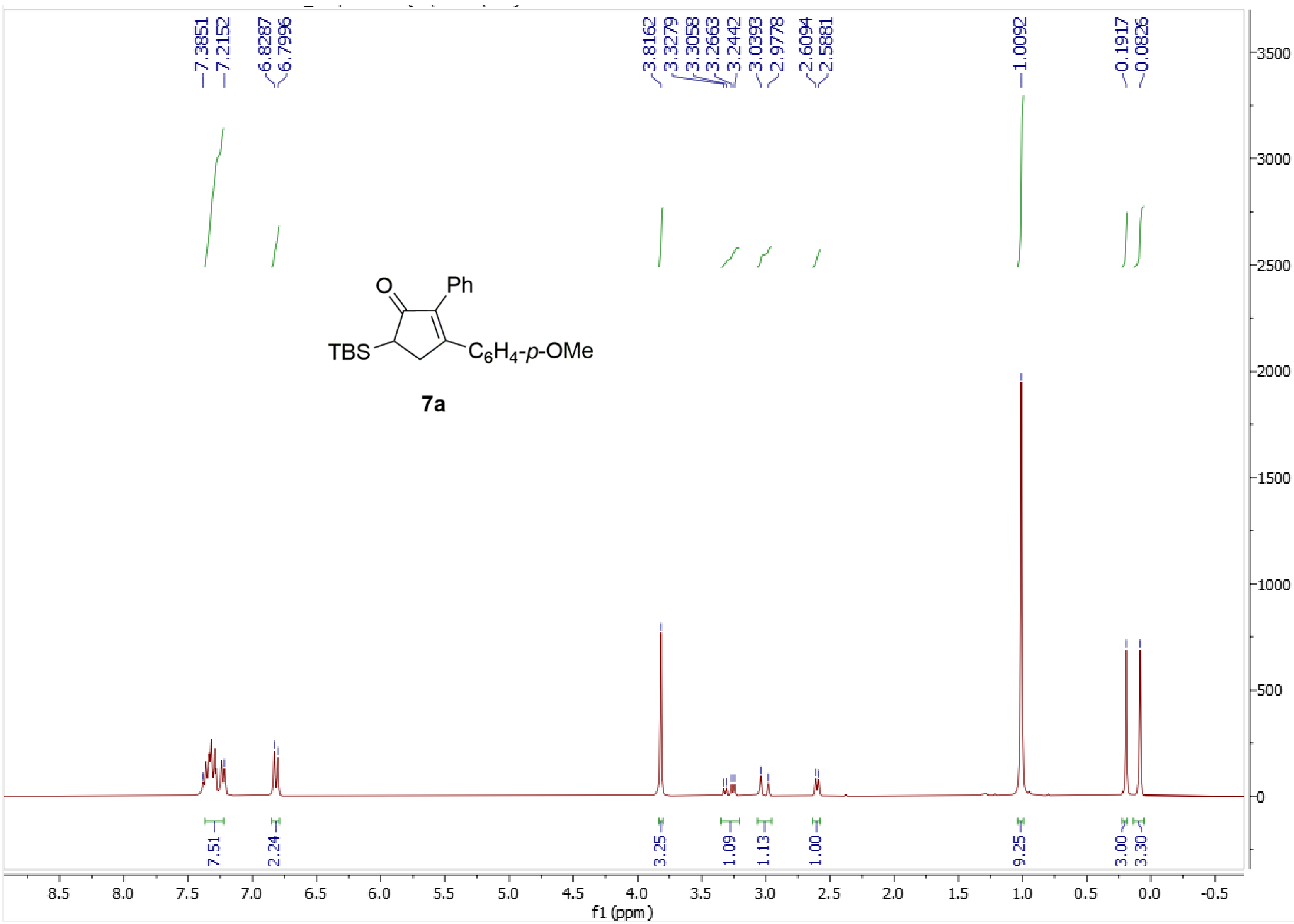

^{13}C NMR spectrum ($CDCl_3$, 75 MHz) of compound **7a**

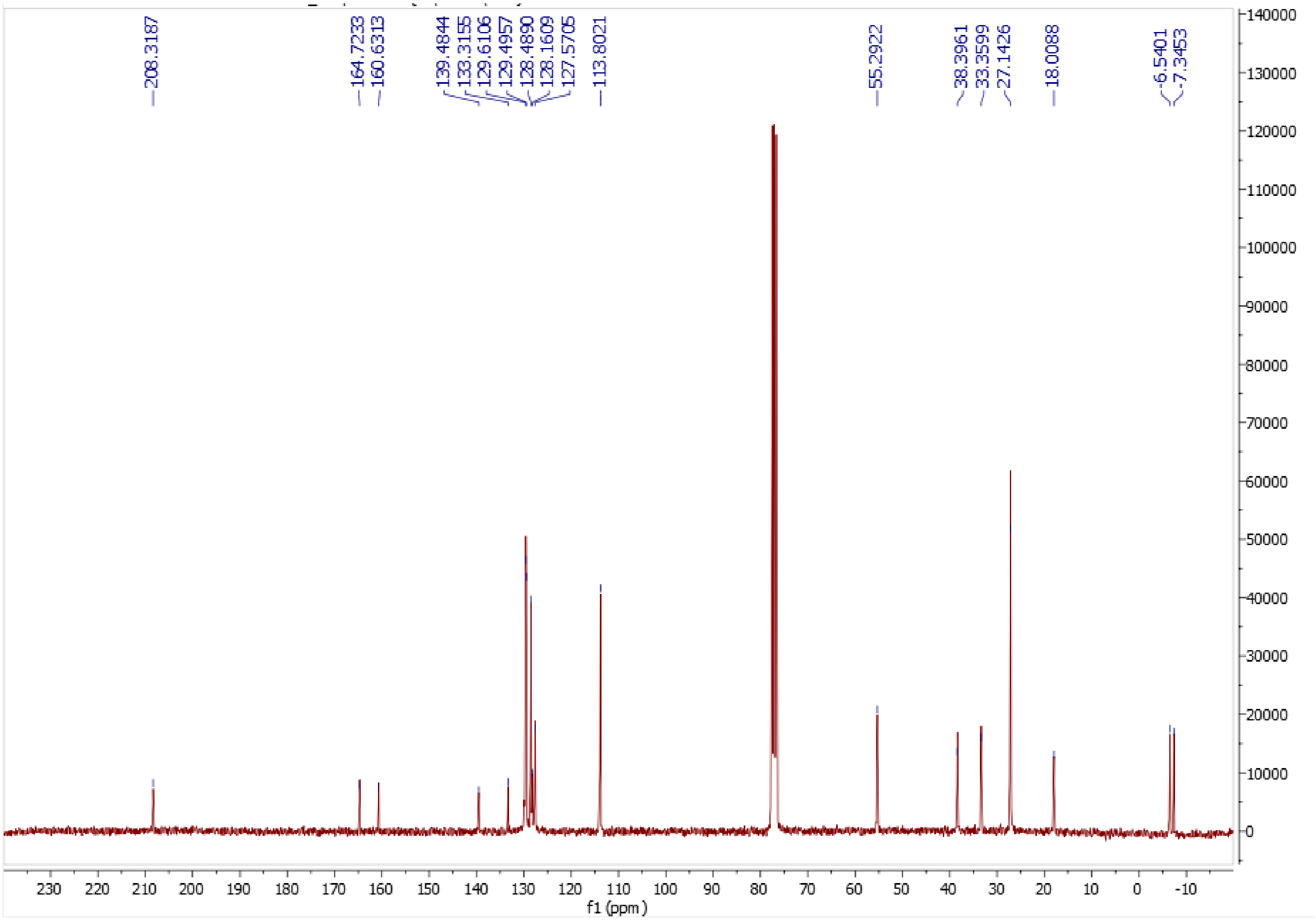

^{1}H NMR spectrum ($CDCl_3$, 300 MHz) of compound **8a**

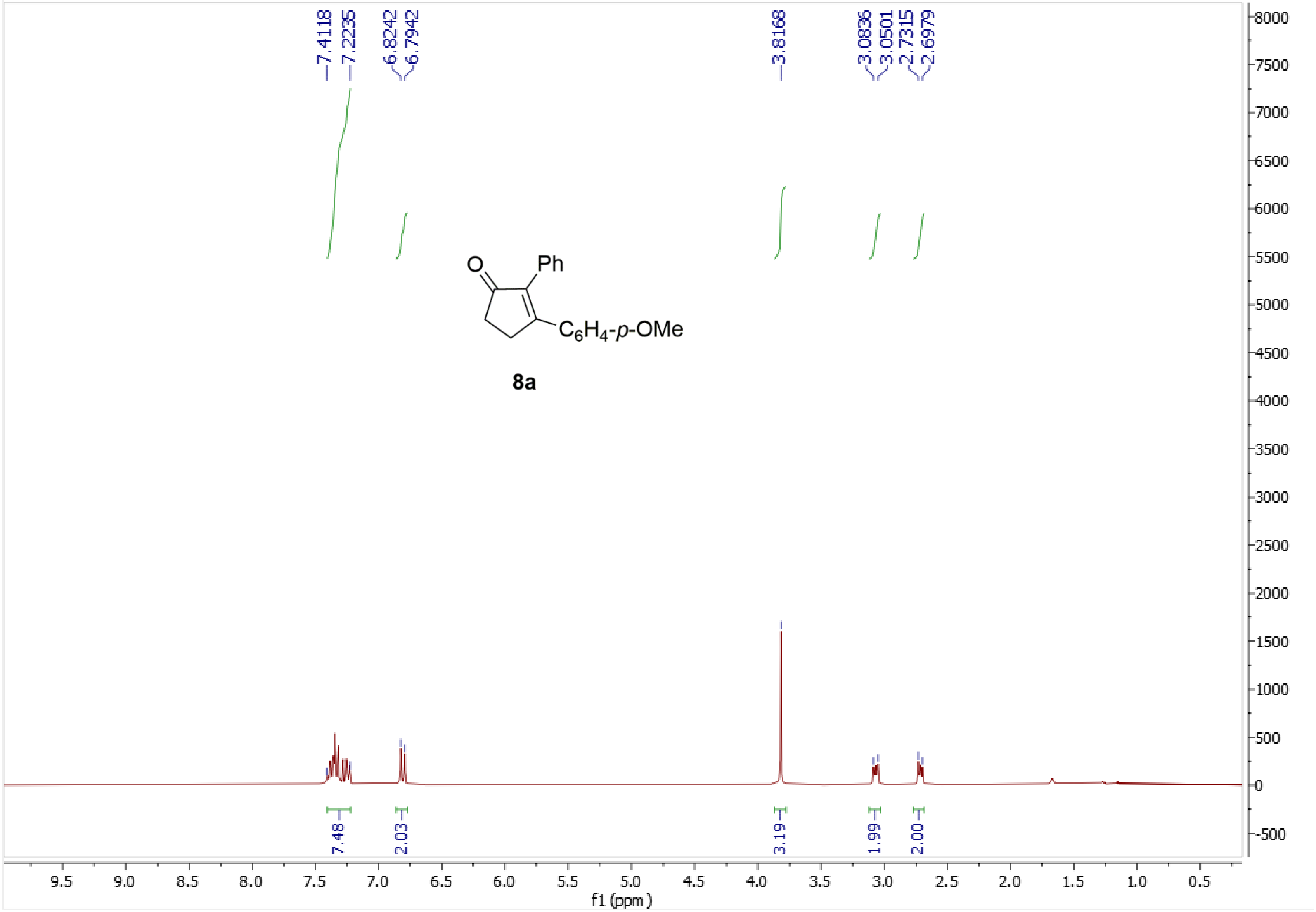

^{13}C NMR spectrum ($CDCl_3$, 75 MHz) of compound **8a**

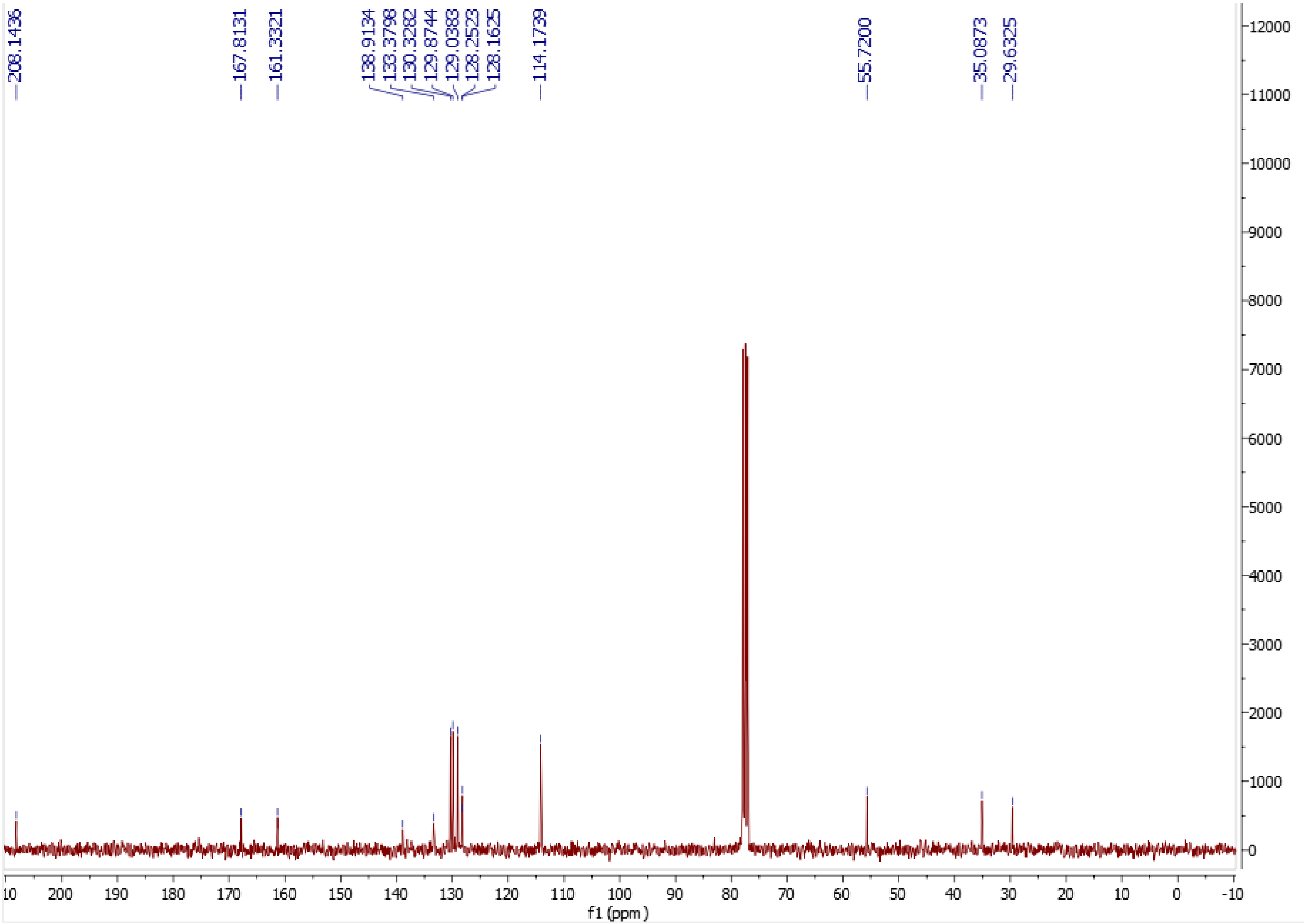

Supplement: Supplementary file 1 — ol2c02035_si_001.pdf [file ol2c02035_si_001.pdf]
